# Supplementary material for: A nuclear protein quality control system for elimination of nucleolus-related inclusions
Source: EMBO J. 2024 Dec 17;44(3):801–23. doi: 10.1038/s44318-024-00333-9 (PMC11791210; doi:10.1038/s44318-024-00333-9)
Supplement: Supplementary file 1 — Appendix [file 44318_2024_333_MOESM1_ESM.pdf]

Appendix for

A nuclear Protein Quality Control system for the elimination of nucleolus-related inclusions

Table of content of the Appendix Figures

Page

Appendix Figure S1……………………………………………………………….2

Appendix Figure S2……………………………………………………………….3

Appendix Figure S3……………………………………………………………….4

Appendix Figure S4……………………………………………………………….5

Appendix Figure S5……………………………………………………………….6

Appendix Figure S6……………………………………………………………….7

Appendix Figure S7………………………………………………………………..8

Appendix Figure S8………………………………………………………………..9

S1

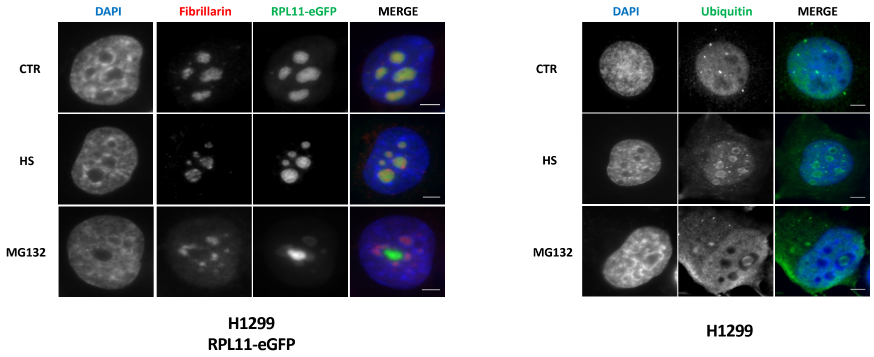

Appendix Figure S1. Effect of HS and MG132 on nucleolar morphology. Related to Fig. 1.
Complete images of the experiment performed in Fig. 1A.

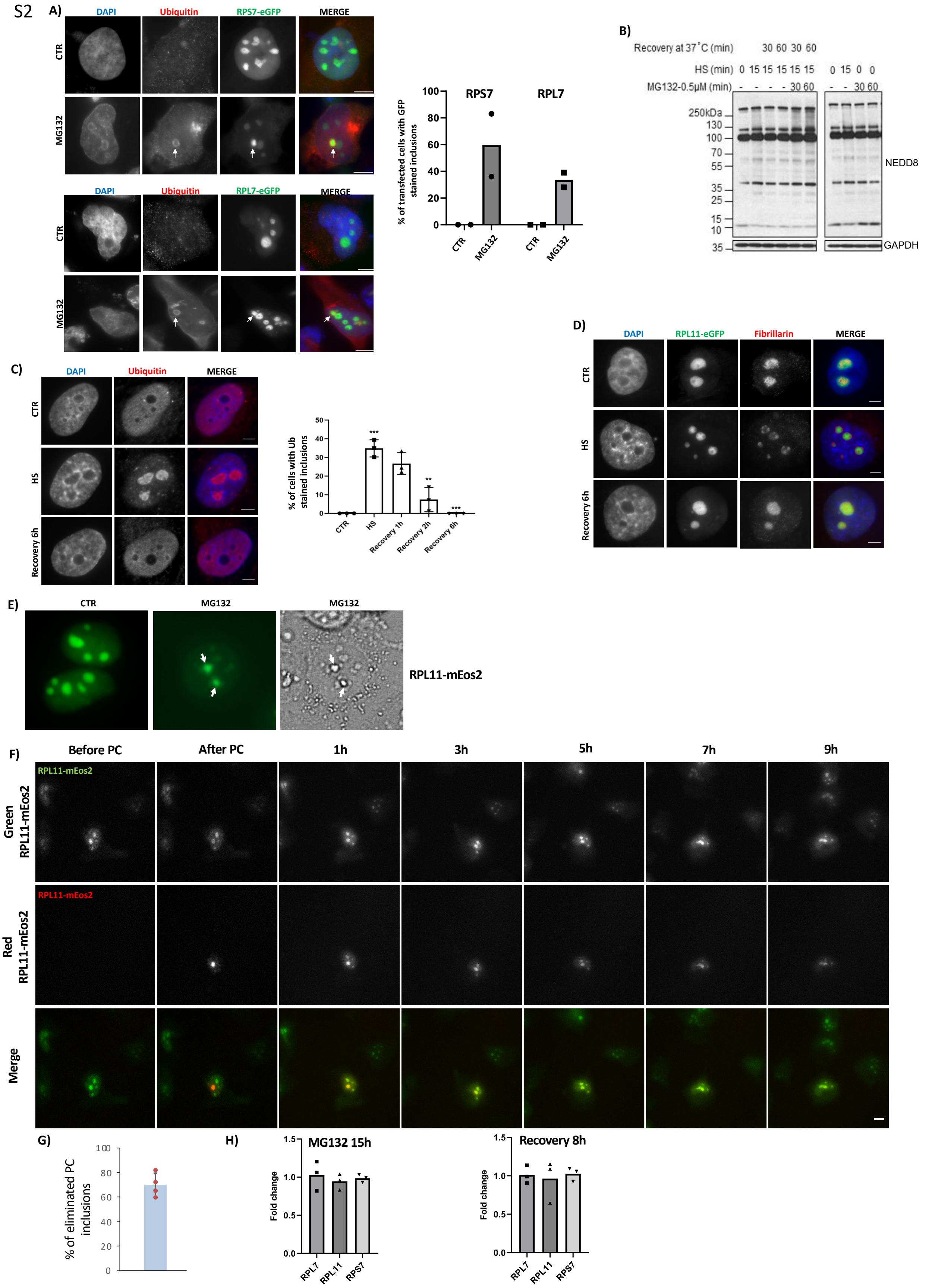

Appendix Figure S2. Nucleolar-related inclusions are eliminated during the recovery period. Related to Fig. 3.

(A). H1299 cells were transfected with constructs expressing either RPS7-eGFP or RPL7-eGFP. 48h post transfection cells were either unstressed or stressed with MG132 (5μM, 15h), stained with Ubiquitin, before imaging. Arrows indicate the formation Ubiquitin-RP inclusions. Graph represents the mean of the % of transfected cells with GFP-RP inclusions. Each dot represents an independent experiment (B). Western blot analysis in extracts from cells exposed to HS and allowed to recover in the absence or presence of low-doses of MG132. (C). H1299 cells were treated as indicated and stained for Ubiquitin. DAP1 was used for nuclear staining. (Right panel). Quantitation of the performed experiment in (C) (n=3). (D). Experiment performed as in (C) in H1299 RPL11-eGFP cells, stained for fibrillarin (red). (E). Expression of RPL11-mEos2 in unstressed (CTR) or MG132 (5μM, 15h) treated H1299 cells. The arrows indicate the localisation of RPL11-mEos2 within the induced inclusions. (F). Photoconversion (PC) of RPL11-mEOs2 in unstressed nucleoli. Upon photoconversion, red RPL11-mEos2 rapidly appears in neighbouring nucleoli, due to the dynamic exchange of functional RPs. Scale bar 10μm (G). Quantitation of the experiment performed in Fig. 3G (n=4). (H). Fold change expression of indicated RP genes upon MG132 treatment (5μM, 15h) and recovery period of 8h. Values indicate the fold change of expression over the unstressed conditions. Each dot represents an independent experiment.

S3

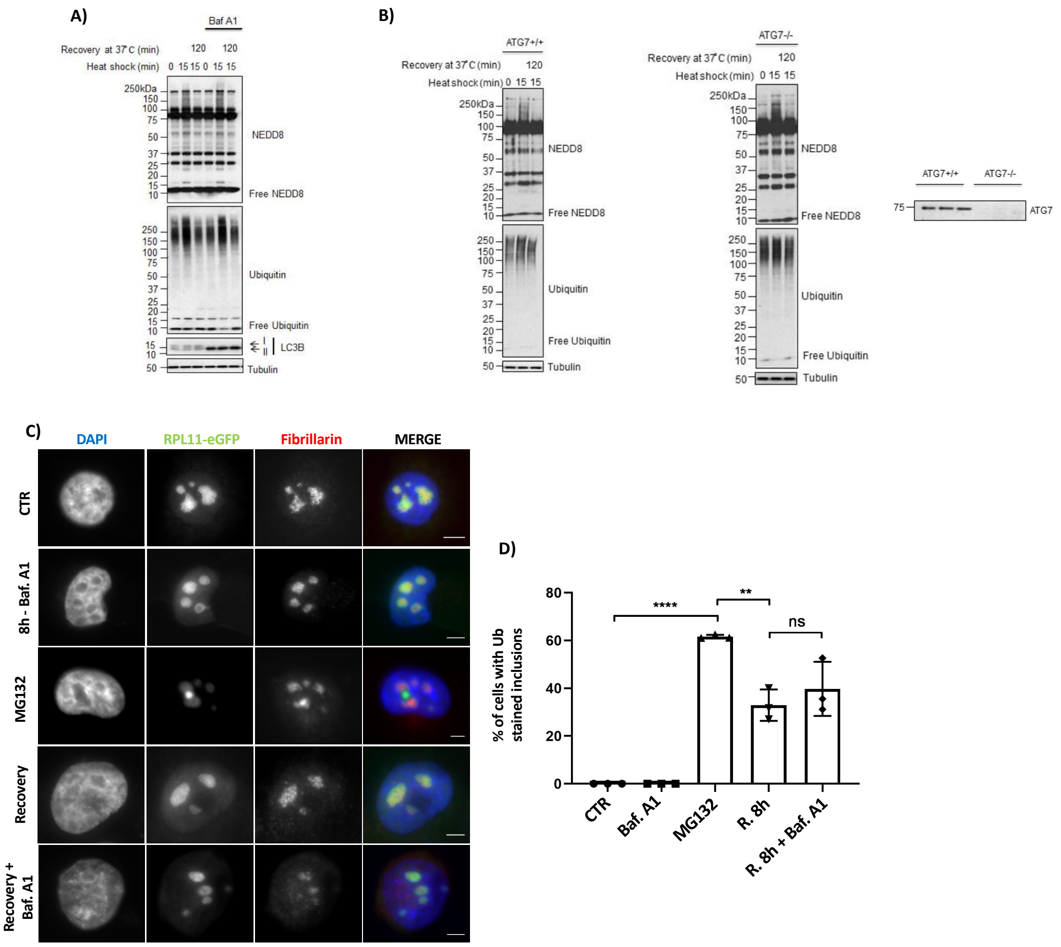

Appendix Figure S3. Nucleolar-related inclusion elimination is independent of the autophagy/lysosome pathway. Related to Fig. 4.
(A). Western blot analysis in extracts from cells exposed to HS and allowed to recover in the absence or presence of the autophagy inhibitor Bafilomycin A1 (100nM, 4hrs). (B). Parental or ATG7 knockout MEFs were heat shocked (43°C) and recovered as indicated, before cell extracts were used for western blotting. (C). H1299 RPL11-eGFP cells were treated as indicated and stained for fibrillarin. DAPI was used for nuclear staining. (D). Quantitation of the performed experiment in (C) (n=3). R. 8h: Recovery 8h.

S4

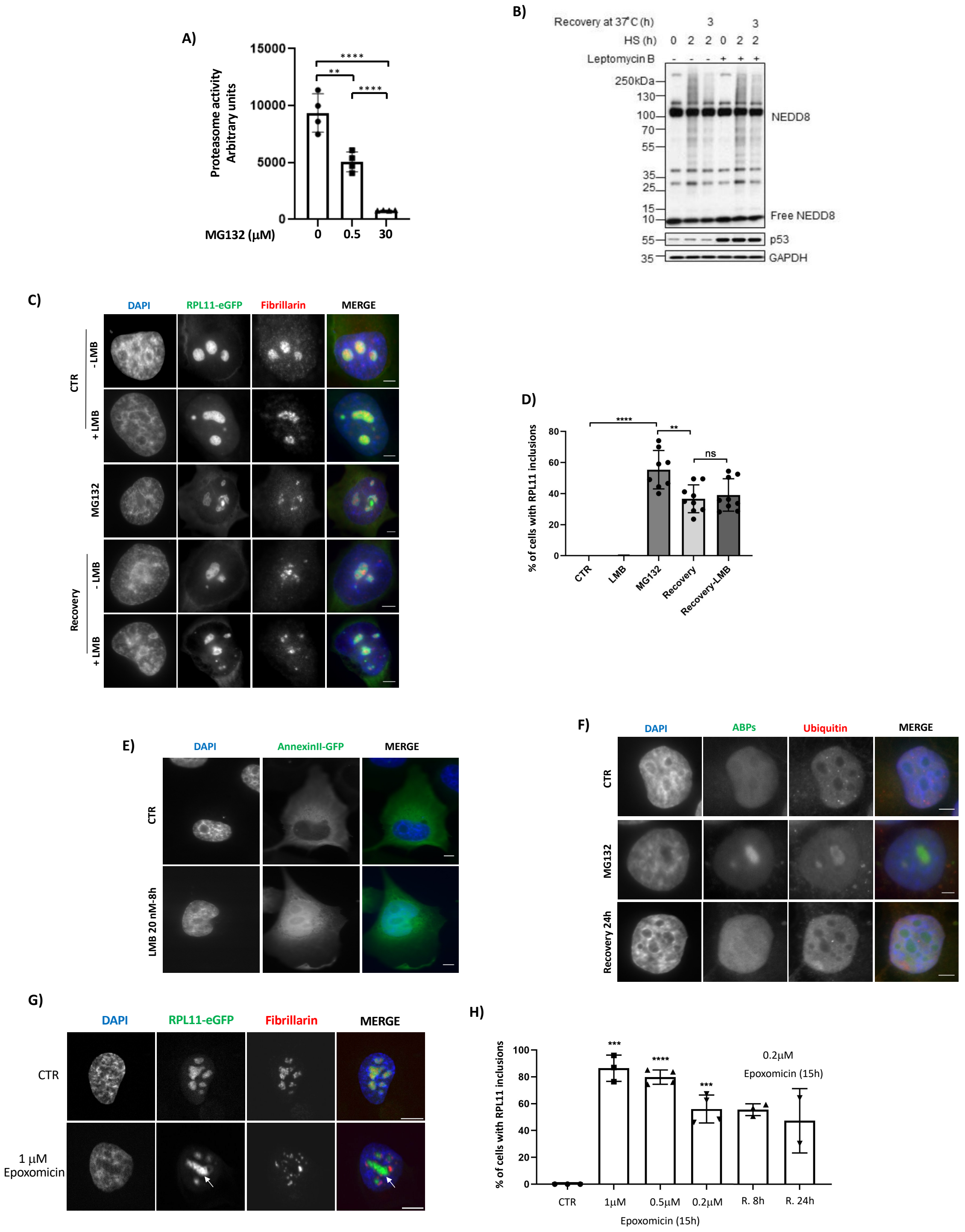

Appendix Figure S4. Nucleolar-related inclusion elimination is independent of the CRM1-mediated nuclear export pathway but depends on active proteasomes. Related to Fig. 4.

(A). H1299 cells were either untreated (CTR, DMSO) or treated for 8h with the indicated doses of MG132 and proteasome activity was measured as described in Methods. The 30μM dose was used as positive control for proteasome inhibition. Values represent the mean +/- SD of 4 independent experiments. (B). Western blot analysis in extracts from cells exposed to HS (43ºC) and allowed to recover in the absence or presence of the CRM1 receptor inhibitor leptomycin B (20nM, 8h prior to stress). Stabilisation of p53 was used as marker of nuclear export inhibition by LMB. (C). H1299 RPL11-eGFP cells were treated as indicated before staining for fibrillarin. DAPI was used for nuclear staining. (D). Quantitation of the experiment in (C) (n=8). (E). Annexin-GFP expressing cells were used as control to test the efficacy of LMB to block nuclear export under the used conditions. (F). H1299 cells were exposed to MG132 (5μM, 15h) before the ABP was applied and cells stained for Ubiquitin. DAPI was used for nuclear staining. (G). H1299 RPL11-eGFP cells were untreated or treated with Epoxomicin for 15h. Arrows indicate the generation of RPL11 inclusions (separation of RPL11 and fibrillarin staining). (H) Graph represents the % of cells with RPL11 inclusions for different Epoxomicin concentrations as the mean +/- SD. Recovery was monitored for 8h or 24h after Epoxomicin treatment (0.2μM, 15h). Each dot indicates an independent experiment.

S5

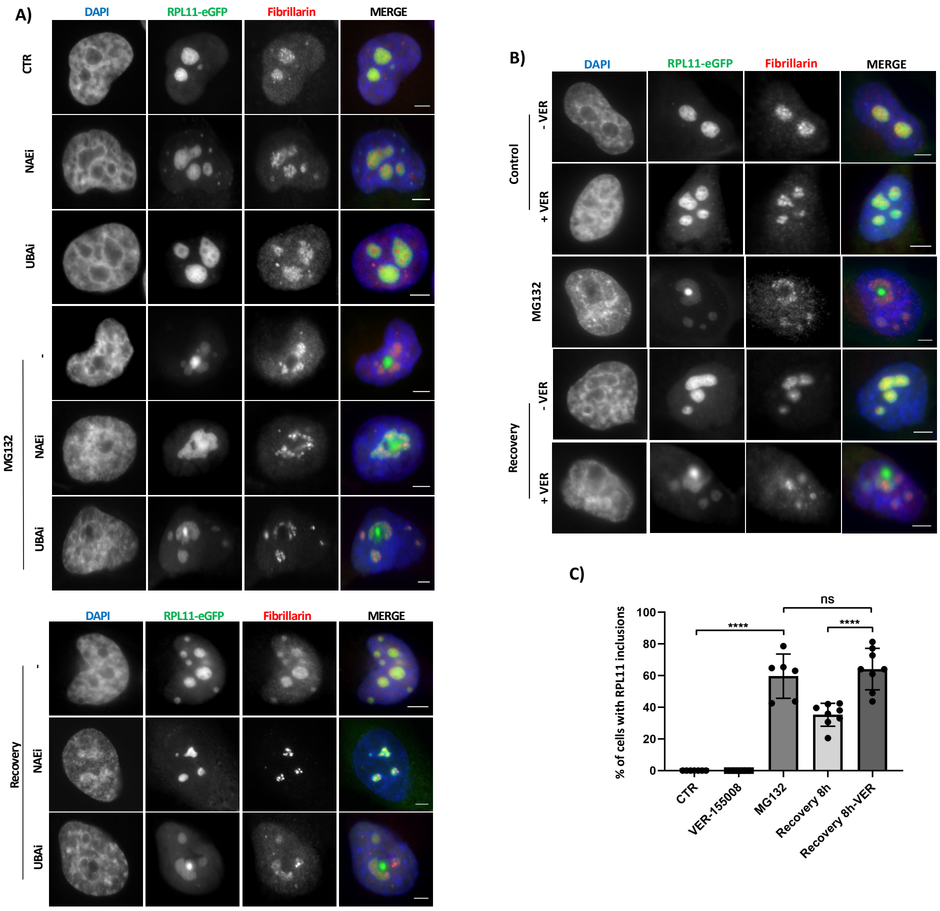

Appendix Figure S5. The role of the Ubiquitin, NEDD8 pathways and HSP70 on the elimination of nucleolus-related inclusions. Related to Fig. 5.

(A). Detailed images of the experiment performed in Fig. 5. (B). H1299 RPL11-eGFP cells were exposed to proteotoxic stress (MG132) and allowed to recover in the absence or presence of the HSP70 inhibitor VER155008, before staining for fibrillarin. DAPI was used for nuclear staining. (C). Quantitation of the experiment performed in (B).

S6

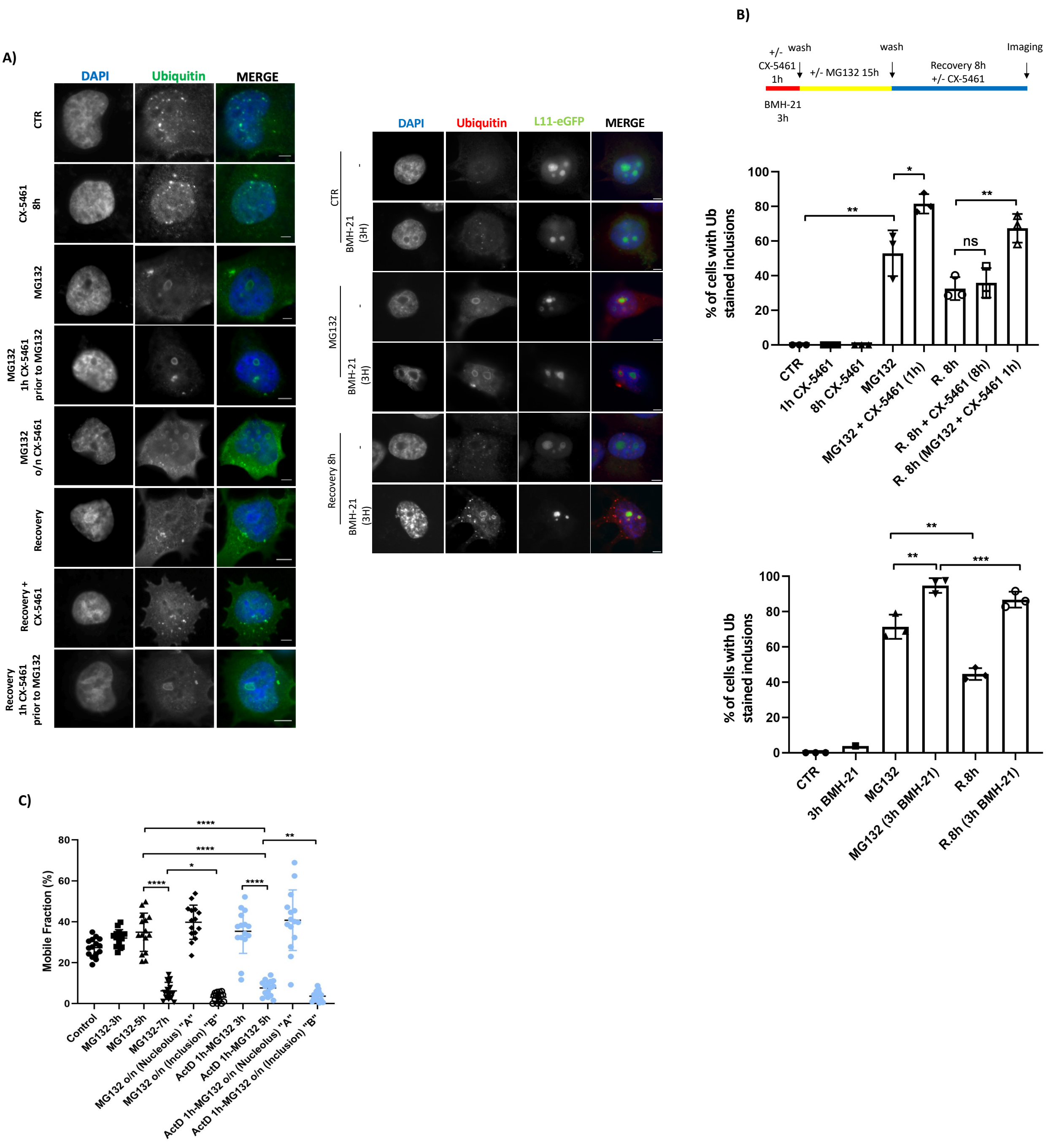

Appendix Figure S6. Nucleolus-related inclusion elimination depends on Pol I activity. Related to Fig. 6.
(A). H1299 or H1299 cells stably expressing RPL11-eGFP were exposed to proteotoxic stress and recovery periods as indicated in the absence or presence of the Pol I inhibitors CX5461 (1μM, 1h/8h or 100nM 15h-o/n) or BMH-21 (0.5μM, 3h). Cells were stained for Ubiquitin and DAPI was used for nuclear staining. (B). Schematic representation of the performed experiment. Bottom panel: Quantitation of the experiment performed in A. R: Recovery, MG132+CX-5461(1h): CX-5461 was added 1hr prior to MG132 treatment, R.8h+CX-5461(8h): CX-5461 was added only during the recovery period. Similar labelling for BMH-21. (C). Kinetic analysis of the effect of ActD treatment on the mobile fraction of RPL11 by FRAP. Effect of Pol I inhibition on RPL11 mobile fraction during MG132-induced proteotoxic stress.

S7

A)

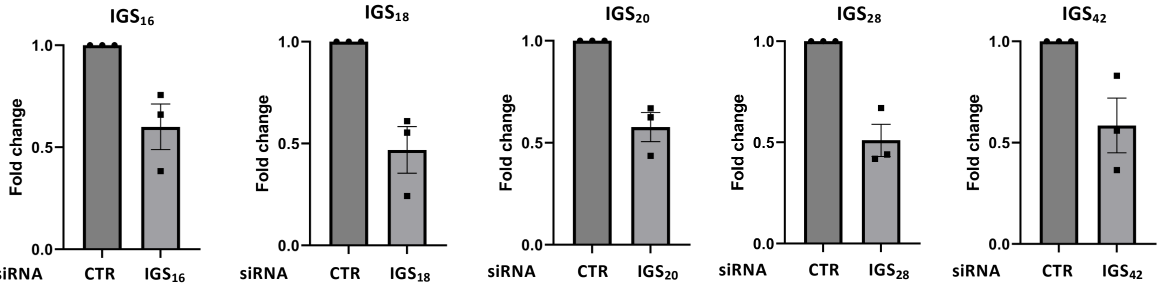

B)

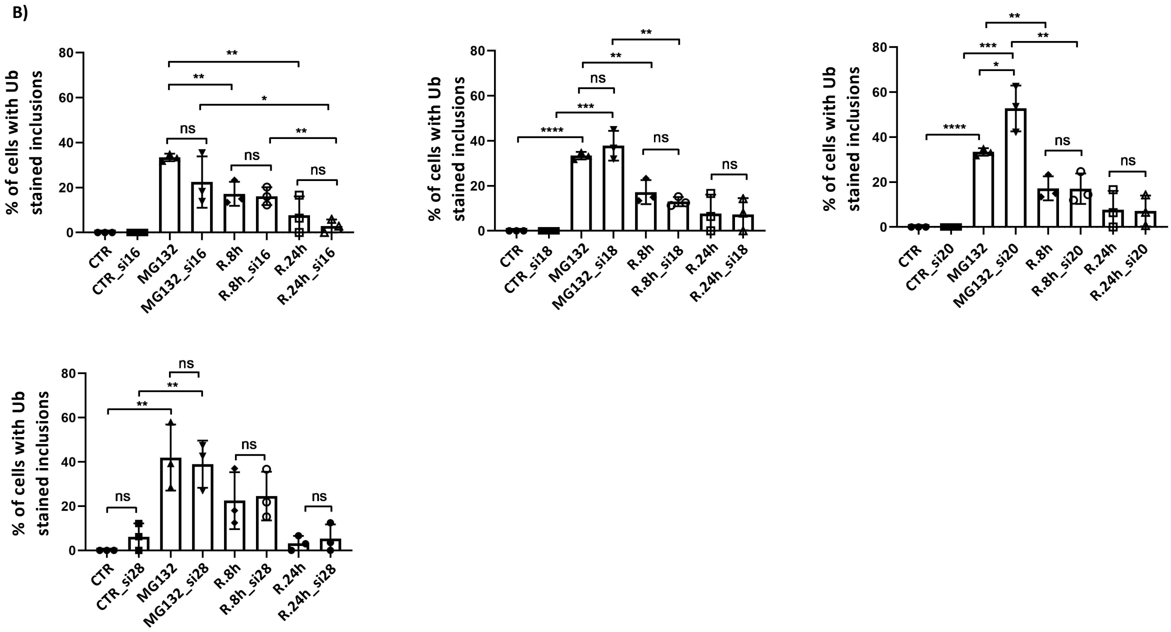

Appendix Figure S7. Knockdown of IGS ncRNAs and the effect on MG132-induced nuclear inclusion response. Related to Fig. 8.

(A). H1299 cells were transfected with siRNAs, either control (CTR) or targeting the indicated IGS lncRNAs. 48h post-transfection cells were treated with MG132 (5μM, 15h). RNA was isolated and qPCR analysis was performed as described in Methods using the CTR condition as reference. Values represent the average +/- SD of 3 independent experiments. (B). Experiment was performed as in (A) using the indicated siRNAs, including a recovery period for 8h and 24h and nuclear inclusions were scored with Ubiquitin staining. Values represent the mean +/- SD of 3 independent experiments. The experiments for siRNA 16, 18 and 20 were performed at the same time and the same control values were used in each graph.

S8

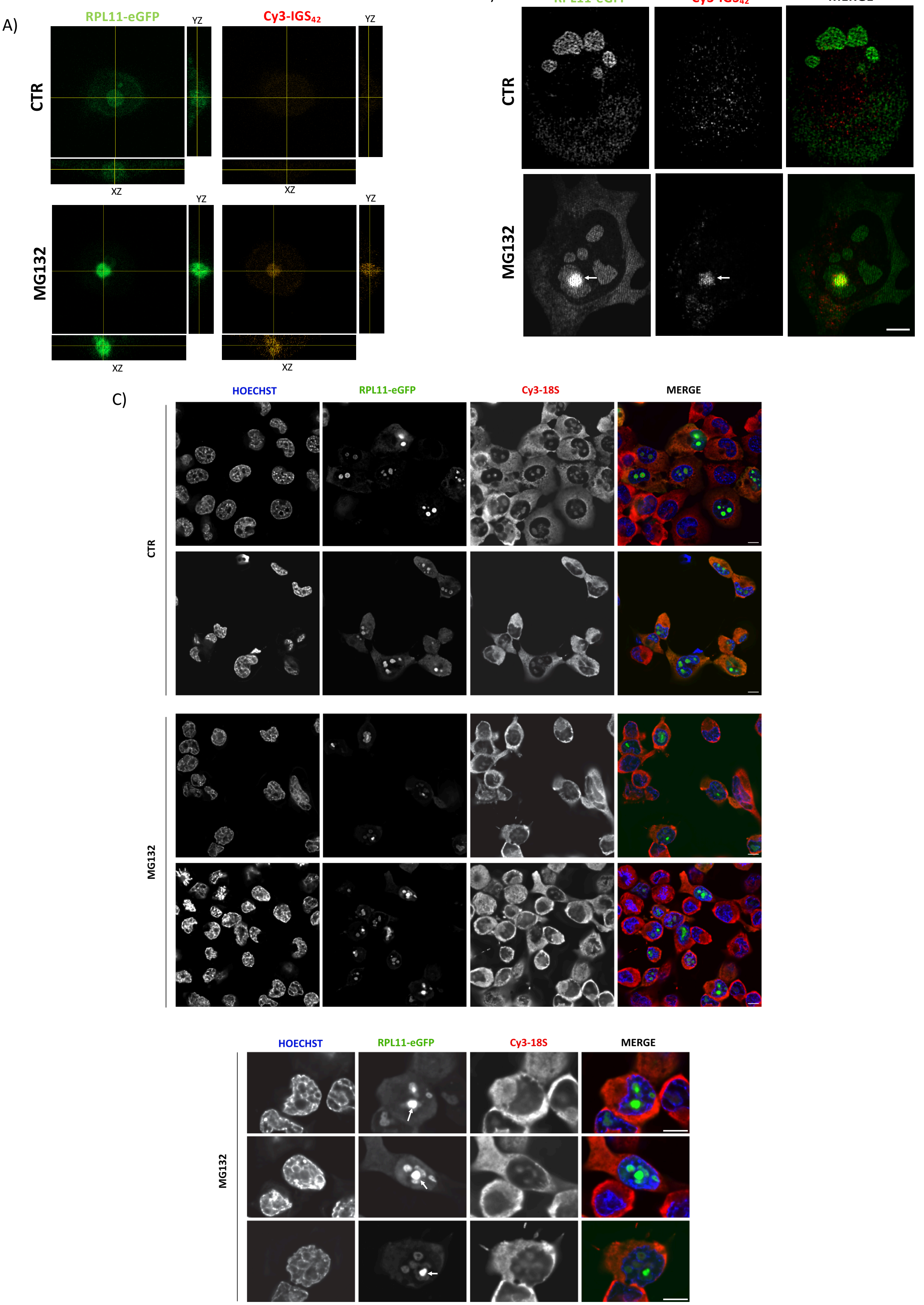

Appendix Figure S8. 18S rRNA is excluded from MG132-induced inclusions. Related to Fig. 9.

(A). Orthogonal projections of the images displayed in Fig. 9B. (B). H1299 cells stably expressing RPL11-eGFP were either untreated (CTR) or treated with MG132 (5μM, 15h). High resolution (RIM) FISH analysis using fluorescent probes against IGS_{42} was performed as described in Methods. Arrows indicate the position of the induced inclusions. Scale bar 10μm. (C). Similar experiment as in (B) using fluorescent probes against 18S. Bottom panel enlarged images of the performed experiment. Arrows indicate the position of the induced inclusions (RPL11-eGFP). Scale bar 10μm.
