# Supplementary material for: A nuclear protein quality control system for elimination of nucleolus-related inclusions
Source: EMBO J. 2024 Dec 17;44(3):801–23. doi: 10.1038/s44318-024-00333-9 (PMC11791210; doi:10.1038/s44318-024-00333-9)
Supplement: Supplementary file 2 — Table EV1 [file 44318_2024_333_MOESM2_ESM.docx]

**Table EV1: FISH probes, primers, and siRNAs used**

**FISH probes**

The probes, conjugated in 5’ with Cy3 or Cy5, were as follows:

ITS1 probe: 5’-CCTCGCCCTCCGGGCTCCGTTAATGATC

ITS2 probe: 5’-GCGATTGATCGGCAAGCGACGCTC

IGS_42_ probe: 5’-TCTGGTCAACCCAAGGACAC

18S probe 5’ACTTCCTCTAGATAGTCAAGTTCGACC

**Primers - For IGS - adapted from**(Audas *et al*, 2012)

47S: 5’-TGTCAGGCGTTCTCGTCTC, 5’-GAGAGCACGACGTCACCAC

IGS_16_: 5’-TGTGTCATTGTCACGTTCATCG, 5’-CGGCAGAGACAGAGGAAGAC

IGS_18_: 5’-CGGTGTGAACGTTTCTCTT, 5’-CACCGAAAACCCACTCAGCC

IGS_20_: 5’-ACAGAGCCTTATTCCCTTCC, 5’-AACACACCCCCTAATCCTC

IGS_21.5_: 5’-CAGTGGCTCACGTCTGTCAT, 5’-CGCCTGACTCCATTTCGTAT

IGS_24_: 5’-CCTGACCACAAATGATCCAC, 5’-TCATTCTGAGCGTAAGGGC

IGS_27.5_: 5’-CGATTTCGGGAGGTCGAGG, 5’-CCACACGTGACCGAGAGAAA

IGS_28_: 5’-GAAAGTCGACGTGACACGGA, 5’-GAGGTTCCCTAGGCGAGGTT

IGS_40_: 5’-TTCCTTGTCTTTCTTCGTGTC, 5’-CTACCTGCTTTCACTACATCTG

IGS_42_: 5’-GGAAGAGCTTCTCGACTCAC, 5’-AGAGCACGATCTCAAAGCG

GAPDH: 5' -TATGACAACAGCCTCAAGAT, 5' -GAGTCCTTCCACGATACC

RPL7: 5’ -TGGCAAGAAAAGCTGGCAAC, 5’ -TTGACGAAGGCGAAGAAGCT

RPL11: 5’ -GGGGAGAGTGGAGACAGACT, 5’ -TGTGCAGTGGACAGCAATCT

RPS7: 5’ -CCAAGCGAAATTGTGGGCAA, 5’ -CCTTGCCCGTGAGCTTCTTA

**siRNAs**

Control siRNA is from Dharmacon and the following sequences were used as a pool:

HUWE1:

1. 5’-AAUUGCUAUGUCUCUGGGACA

2. 5’-CUGUGAGAGUGAUCGGGAA

For IGS lncRNA siRNA we adapted sequences from(Audas *et al*, 2012)

IGS lncRNA_42_:

1. 5’-GCUCUGGCGUGCAGGUUUA

2. 5’-CCGCGUGUGUCCUUGGGUU

3. 5’-CGACCGAGUCCUUGUGUGU

IGS lncRNA_16_:

5’-ACGCUGCCGUGUAUGAACAUA

IGS lncRNA_18_:

5’-CCCACCGGCUACCUGCCACCU

IGS lncRNA_20_:

5’-GCACUGUAUUGCUACUG

IGS lncRNA_28:_

5’-CAAGGCCCGCCUGUCUAGA
