# Supplementary material for: A nuclear protein quality control system for elimination of nucleolus-related inclusions
Source: EMBO J. 2024 Dec 17;44(3):801–23. doi: 10.1038/s44318-024-00333-9 (PMC11791210; doi:10.1038/s44318-024-00333-9)
Supplement: Supplementary file 5 — Movie EV1 [file 44318_2024_333_MOESM5_ESM.zip › Movie EV1/Text_EV1.rtf]

Movie EV1Formation of RPL11 inclusions upon MG132 treatment. H1299 cells stably expressing RPL11-eGFP were transfected with iRFP-nucleolin plasmid. 36h post transfection cells were treated with MG132 (5uM) and the formation of RPL11 inclusions (separation of GFP and RFP fluorescence) was monitored by live imaging over a period of 15h. Arrow indicates the formation of RPL11 inclusions. Scale bar 5um.
