# Supplementary material for: A nuclear protein quality control system for elimination of nucleolus-related inclusions
Source: EMBO J. 2024 Dec 17;44(3):801–23. doi: 10.1038/s44318-024-00333-9 (PMC11791210; doi:10.1038/s44318-024-00333-9)
Supplement: Supplementary file 6 — Movie EV2 [file 44318_2024_333_MOESM6_ESM.zip › Movie EV2/Text_EV2.rtf]

Movie EV2Elimination of RPL11 inclusions. H1299 cells stably expressing RPL11-eGFP were transfected with iRFP-nucleolin plasmid. 36h post transfection cells were treated with MG132 (5uM) for 15h. Cells were washed and the recovery of RPL11 formed inclusions was monitored by live imaging over a period of 15h. Scale bar 5um.
