# Supplementary material for: A nuclear protein quality control system for elimination of nucleolus-related inclusions
Source: EMBO J. 2024 Dec 17;44(3):801–23. doi: 10.1038/s44318-024-00333-9 (PMC11791210; doi:10.1038/s44318-024-00333-9)
Supplement: Supplementary file 7 — Movie EV3 [file 44318_2024_333_MOESM7_ESM.zip › Movie EV4/Text_EV4.rtf]

Movie EV4H1299 cells were transfected with RPL11-mEOS2 construct and 36h post transfection were treated with MG132 (5uM) for 15h  before photoconversion of RPL11 within the formed inclusions. The fate of the photoconverted RPL11 (red) was followed by live imaging for 10h. Scale bar 10um.  
