# Supplementary material for: A nuclear protein quality control system for elimination of nucleolus-related inclusions
Source: EMBO J. 2024 Dec 17;44(3):801–23. doi: 10.1038/s44318-024-00333-9 (PMC11791210; doi:10.1038/s44318-024-00333-9)
Supplement: Supplementary file 8 — Movie EV4 [file 44318_2024_333_MOESM8_ESM.zip › Movie EV3/Text_EV3.rtf]

Movie EV3
H1299 cells were transfected with RPL11-mEOS2 construct and 36h post transfection photoconversion was performed in nucleoli as described in Methods. The green and red version of RPL11 was followed by live imaging for 10h. Scale bar 10um.
