# Supplementary figures and images for: A nuclear protein quality control system for elimination of nucleolus-related inclusions (part 1 of 4)
Source: EMBO J. 2024 Dec 17;44(3):801–23. doi: 10.1038/s44318-024-00333-9 (PMC11791210; doi:10.1038/s44318-024-00333-9)

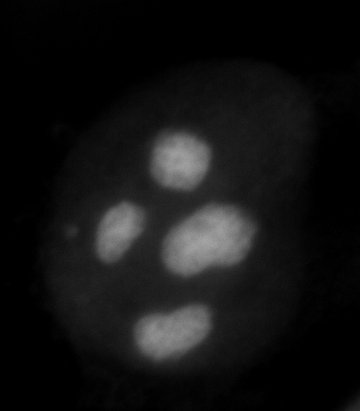

Supplement: Supplementary file 9 — Source data Fig. 1 [file 44318_2024_333_MOESM9_ESM.zip › Figure 1/Figure 1A/H1299_L11_GFP_Fibrillarin/CTR/080421_coat L11 GFP_UNT_7_w1GFP.jpg]

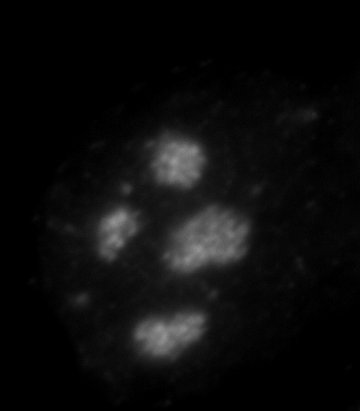

Supplement: Supplementary file 9 — Source data Fig. 1 [file 44318_2024_333_MOESM9_ESM.zip › Figure 1/Figure 1A/H1299_L11_GFP_Fibrillarin/CTR/080421_coat L11 GFP_UNT_7_w2Texas Red.jpg]

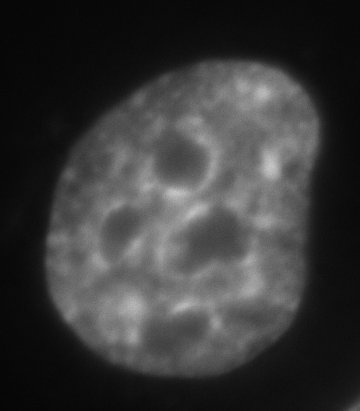

Supplement: Supplementary file 9 — Source data Fig. 1 [file 44318_2024_333_MOESM9_ESM.zip › Figure 1/Figure 1A/H1299_L11_GFP_Fibrillarin/CTR/080421_coat L11 GFP_UNT_7_w3Hoechst.jpg]

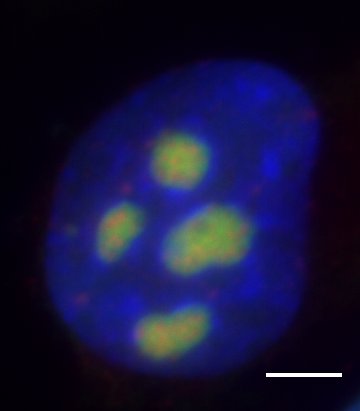

Supplement: Supplementary file 9 — Source data Fig. 1 [file 44318_2024_333_MOESM9_ESM.zip › Figure 1/Figure 1A/H1299_L11_GFP_Fibrillarin/CTR/Composite CTR HS.jpg]

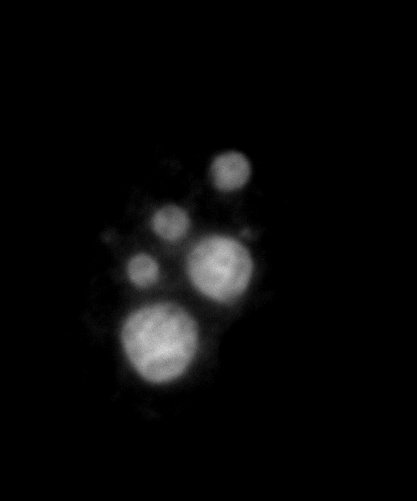

Supplement: Supplementary file 9 — Source data Fig. 1 [file 44318_2024_333_MOESM9_ESM.zip › Figure 1/Figure 1A/H1299_L11_GFP_Fibrillarin/HS/080421_coat L11 GFP_HS_5_w1GFP.jpg]

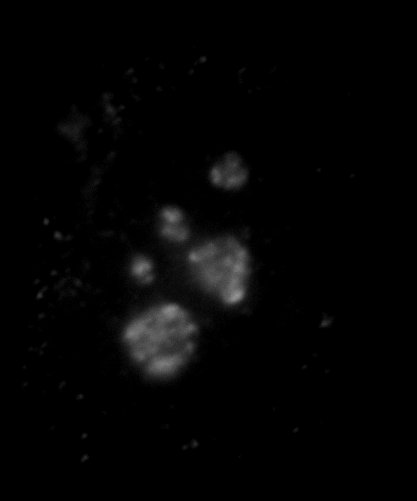

Supplement: Supplementary file 9 — Source data Fig. 1 [file 44318_2024_333_MOESM9_ESM.zip › Figure 1/Figure 1A/H1299_L11_GFP_Fibrillarin/HS/080421_coat L11 GFP_HS_5_w2Texas Red.jpg]

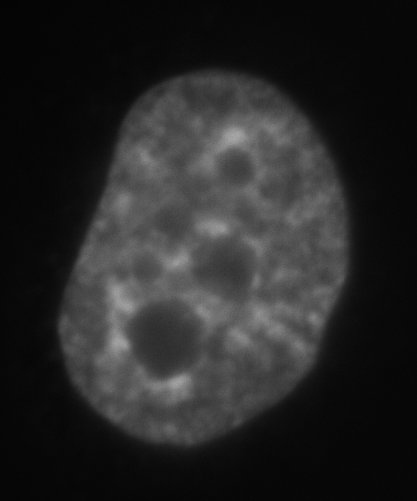

Supplement: Supplementary file 9 — Source data Fig. 1 [file 44318_2024_333_MOESM9_ESM.zip › Figure 1/Figure 1A/H1299_L11_GFP_Fibrillarin/HS/080421_coat L11 GFP_HS_5_w3Hoechst.jpg]

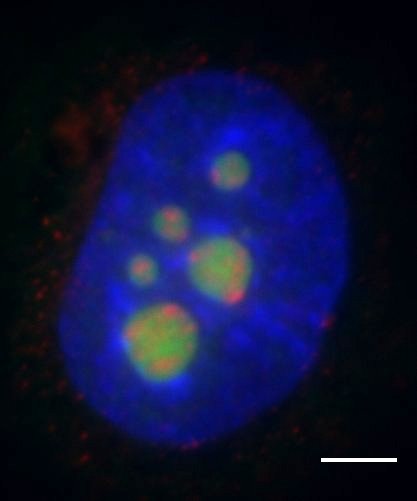

Supplement: Supplementary file 9 — Source data Fig. 1 [file 44318_2024_333_MOESM9_ESM.zip › Figure 1/Figure 1A/H1299_L11_GFP_Fibrillarin/HS/Composite.jpg]

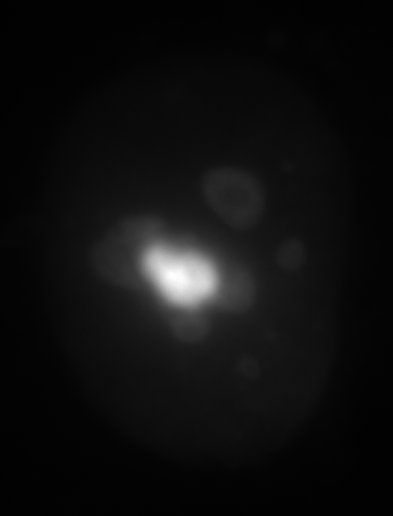

Supplement: Supplementary file 9 — Source data Fig. 1 [file 44318_2024_333_MOESM9_ESM.zip › Figure 1/Figure 1A/H1299_L11_GFP_Fibrillarin/MG132/300919_MG_4_w1GFP.jpg]

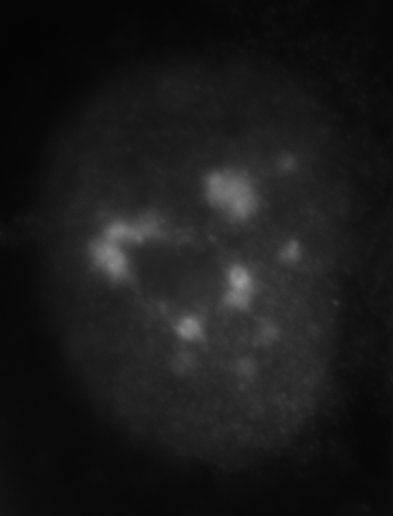

Supplement: Supplementary file 9 — Source data Fig. 1 [file 44318_2024_333_MOESM9_ESM.zip › Figure 1/Figure 1A/H1299_L11_GFP_Fibrillarin/MG132/300919_MG_4_w2TexasRed.jpg]

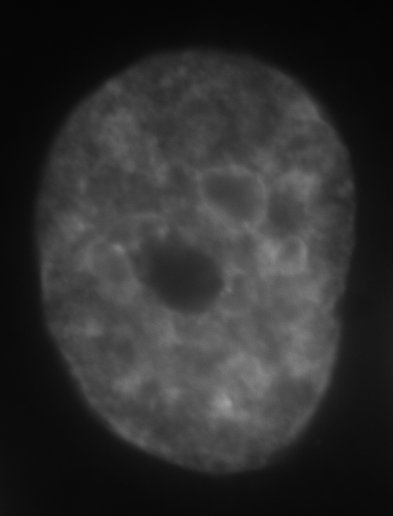

Supplement: Supplementary file 9 — Source data Fig. 1 [file 44318_2024_333_MOESM9_ESM.zip › Figure 1/Figure 1A/H1299_L11_GFP_Fibrillarin/MG132/300919_MG_4_w3DAPI.jpg]

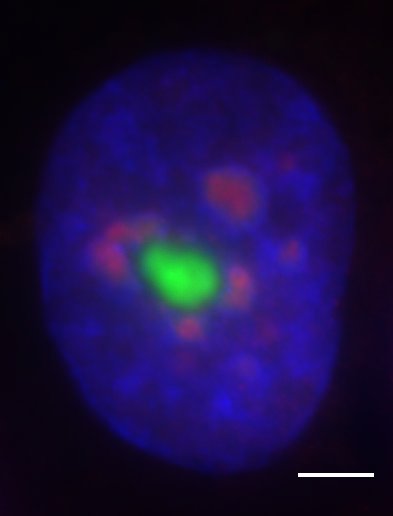

Supplement: Supplementary file 9 — Source data Fig. 1 [file 44318_2024_333_MOESM9_ESM.zip › Figure 1/Figure 1A/H1299_L11_GFP_Fibrillarin/MG132/Composite MG.jpg]

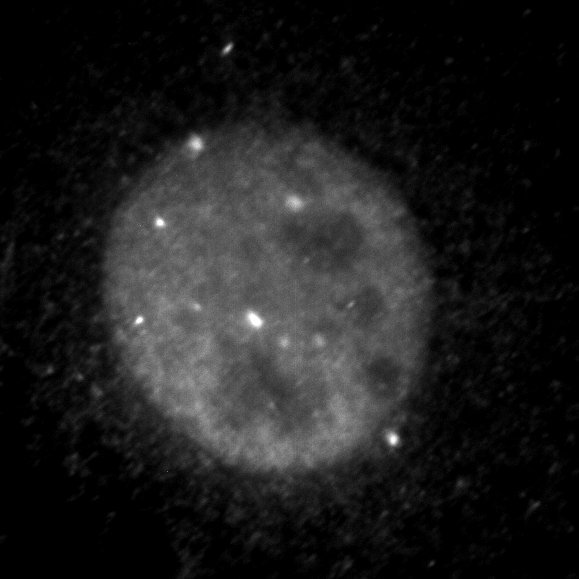

Supplement: Supplementary file 9 — Source data Fig. 1 [file 44318_2024_333_MOESM9_ESM.zip › Figure 1/Figure 1A/H1299_Ub/CTR/30.05.20_ubiquitin_unt_1_w1GFP.jpg]

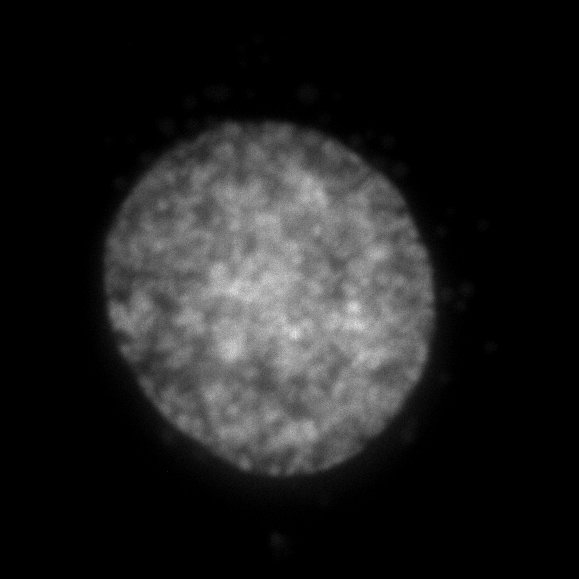

Supplement: Supplementary file 9 — Source data Fig. 1 [file 44318_2024_333_MOESM9_ESM.zip › Figure 1/Figure 1A/H1299_Ub/CTR/30.05.20_ubiquitin_unt_1_w2Hoechst.jpg]

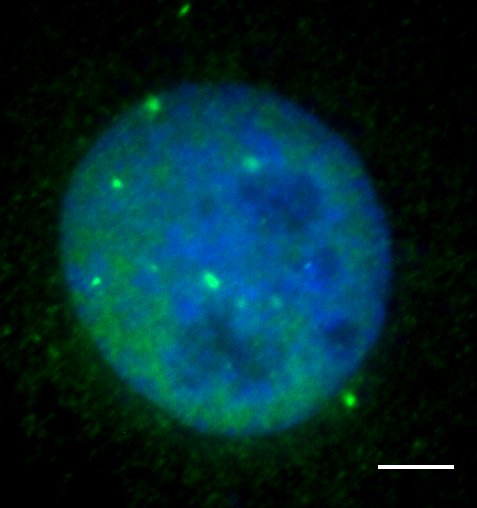

Supplement: Supplementary file 9 — Source data Fig. 1 [file 44318_2024_333_MOESM9_ESM.zip › Figure 1/Figure 1A/H1299_Ub/CTR/Composite CTR.jpg]

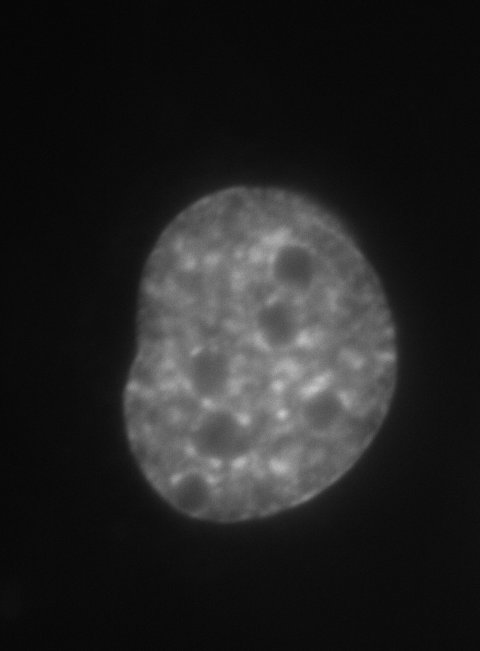

Supplement: Supplementary file 9 — Source data Fig. 1 [file 44318_2024_333_MOESM9_ESM.zip › Figure 1/Figure 1A/H1299_Ub/HS/131120__HS_w1Hoechst.jpg]

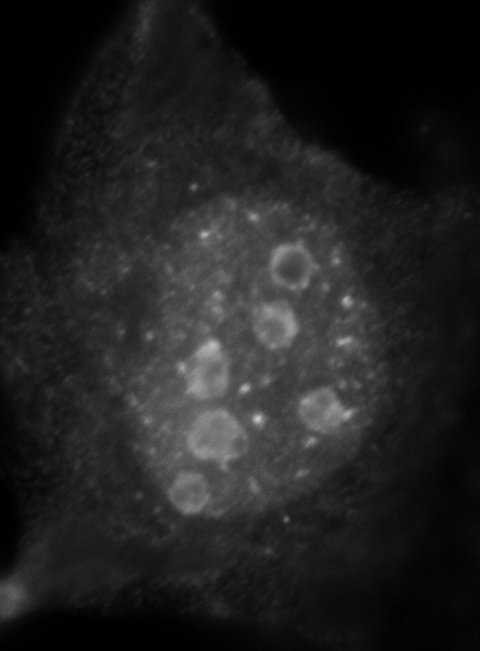

Supplement: Supplementary file 9 — Source data Fig. 1 [file 44318_2024_333_MOESM9_ESM.zip › Figure 1/Figure 1A/H1299_Ub/HS/131120_HS_w2GFP.jpg]

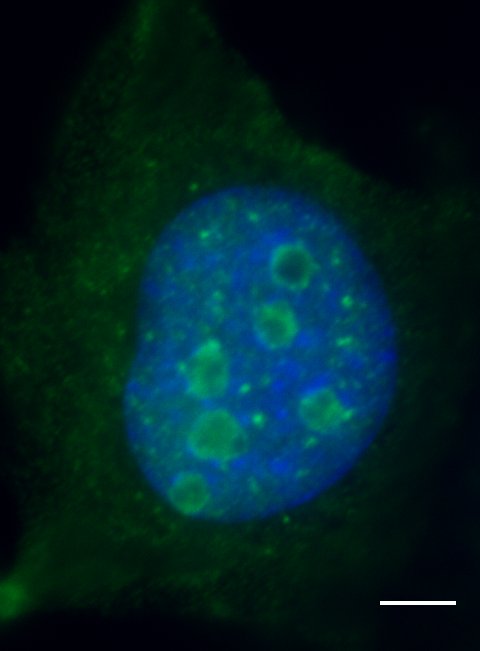

Supplement: Supplementary file 9 — Source data Fig. 1 [file 44318_2024_333_MOESM9_ESM.zip › Figure 1/Figure 1A/H1299_Ub/HS/Composite HS HS.jpg]

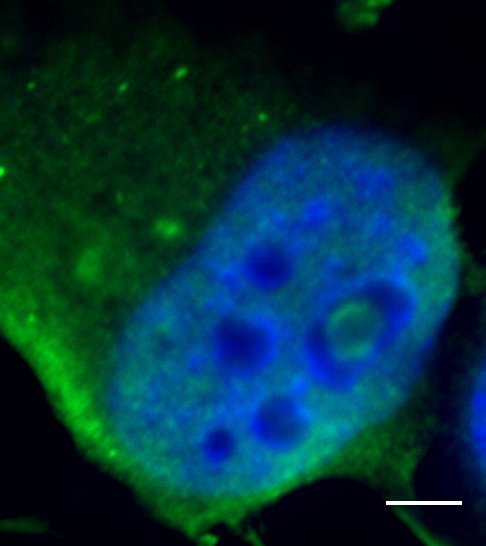

Supplement: Supplementary file 9 — Source data Fig. 1 [file 44318_2024_333_MOESM9_ESM.zip › Figure 1/Figure 1A/H1299_Ub/MG132/Composite MG ub scale.jpg]

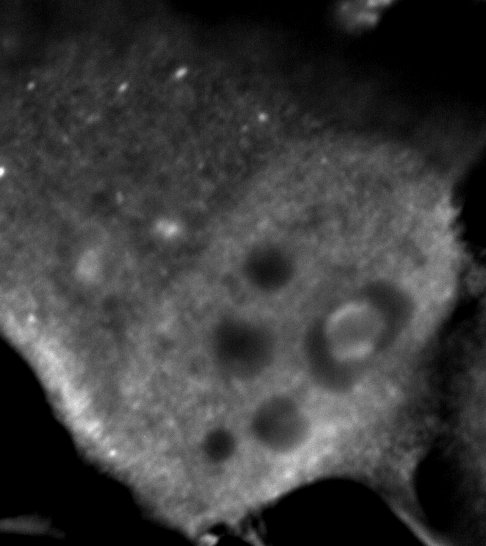

Supplement: Supplementary file 9 — Source data Fig. 1 [file 44318_2024_333_MOESM9_ESM.zip › Figure 1/Figure 1A/H1299_Ub/MG132/ubiquitin_04062020_mg_15_w1GFP.jpg]

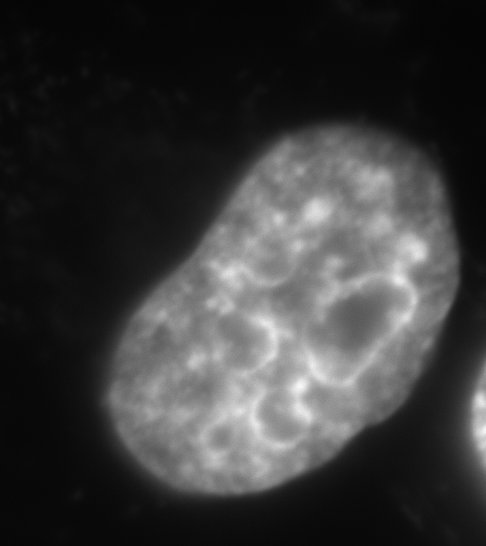

Supplement: Supplementary file 9 — Source data Fig. 1 [file 44318_2024_333_MOESM9_ESM.zip › Figure 1/Figure 1A/H1299_Ub/MG132/ubiquitin_04062020_mg_15_w2Hoechst.jpg]

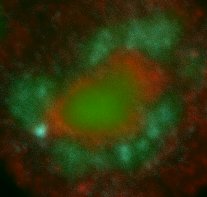

Supplement: Supplementary file 9 — Source data Fig. 1 [file 44318_2024_333_MOESM9_ESM.zip › Figure 1/Figure 1C/Composite - zoom.jpg]

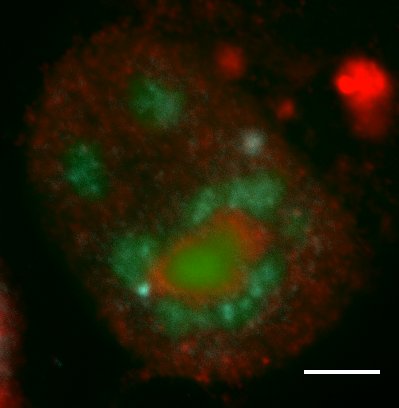

Supplement: Supplementary file 9 — Source data Fig. 1 [file 44318_2024_333_MOESM9_ESM.zip › Figure 1/Figure 1C/Composite.jpg]

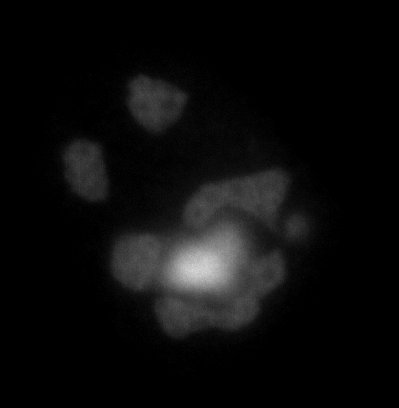

Supplement: Supplementary file 9 — Source data Fig. 1 [file 44318_2024_333_MOESM9_ESM.zip › Figure 1/Figure 1C/ub figure_3_w1GFP.jpg]

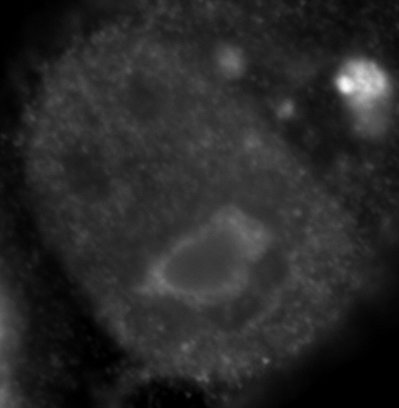

Supplement: Supplementary file 9 — Source data Fig. 1 [file 44318_2024_333_MOESM9_ESM.zip › Figure 1/Figure 1C/ub figure_3_w2TexasRed.jpg]

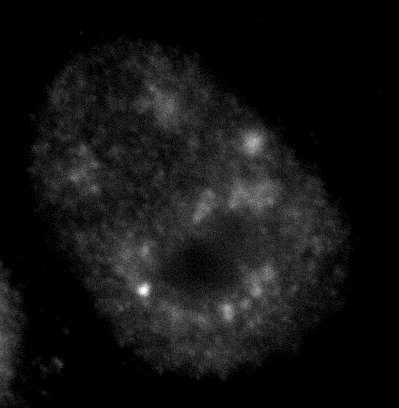

Supplement: Supplementary file 9 — Source data Fig. 1 [file 44318_2024_333_MOESM9_ESM.zip › Figure 1/Figure 1C/ub figure_3_w3Cy5.jpg]

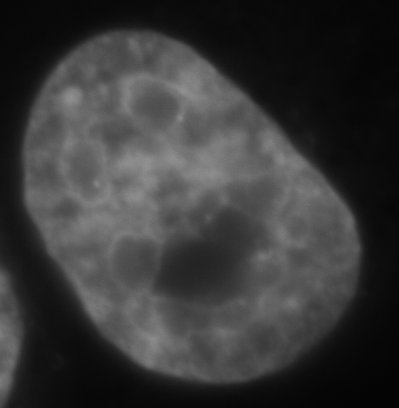

Supplement: Supplementary file 9 — Source data Fig. 1 [file 44318_2024_333_MOESM9_ESM.zip › Figure 1/Figure 1C/ub figure_3_w4DAPI.jpg]

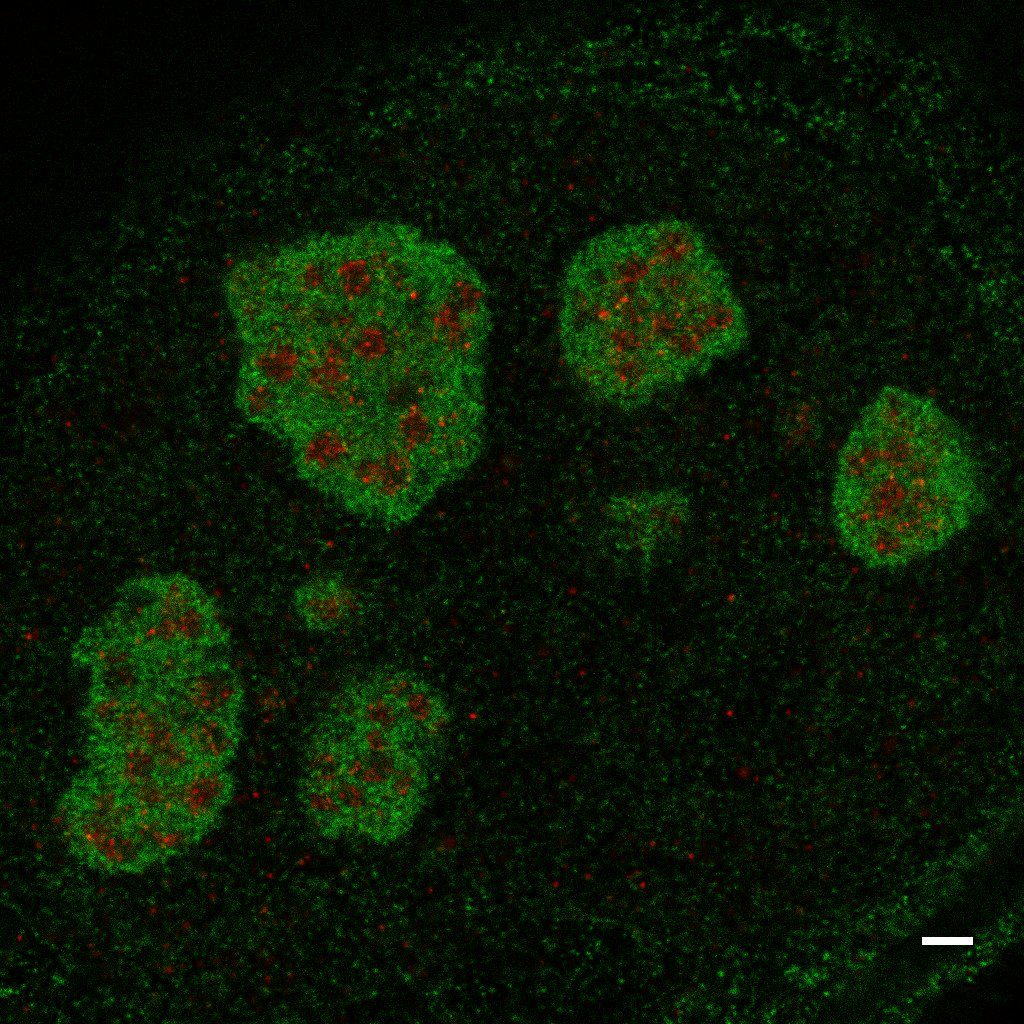

Supplement: Supplementary file 9 — Source data Fig. 1 [file 44318_2024_333_MOESM9_ESM.zip › Figure 1/Figure 1D/CTR/post expansion/Composite.jpg]

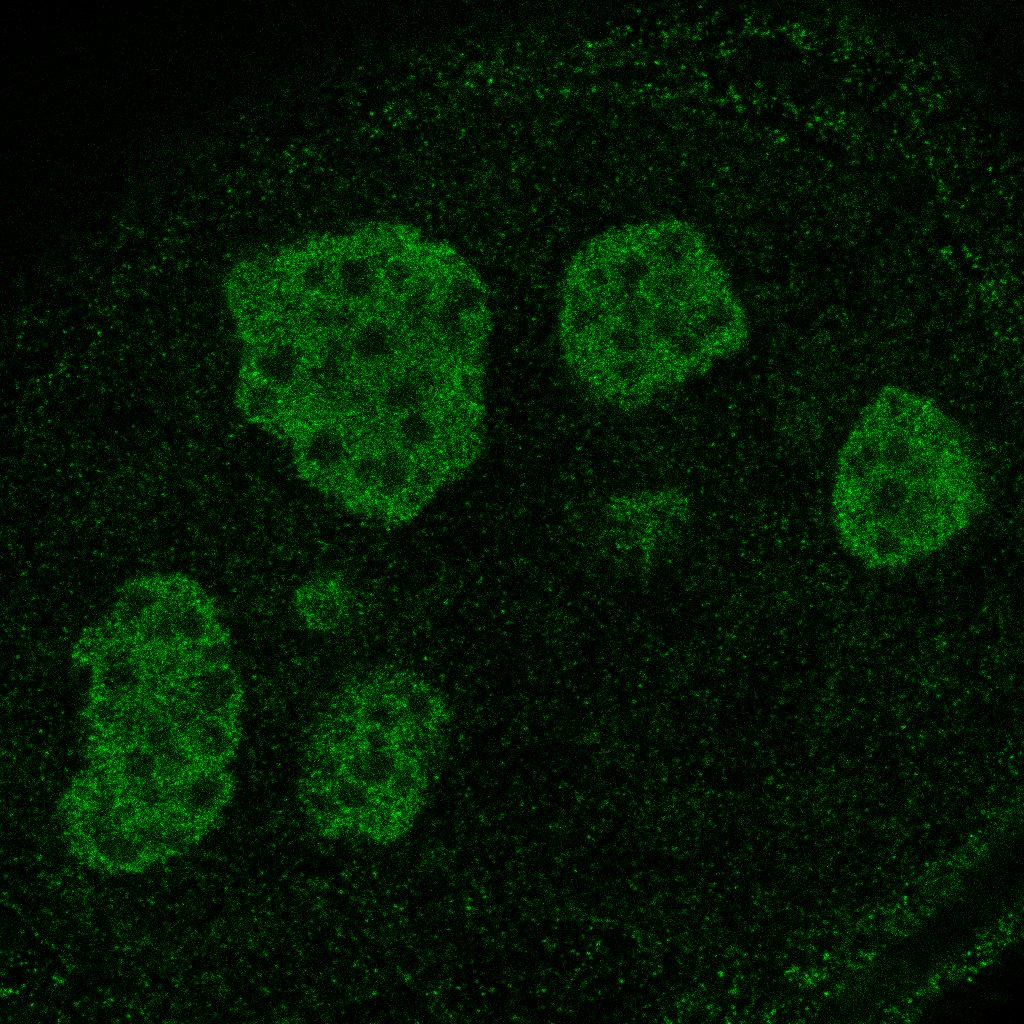

Supplement: Supplementary file 9 — Source data Fig. 1 [file 44318_2024_333_MOESM9_ESM.zip › Figure 1/Figure 1D/CTR/post expansion/Expansion_nucleole_02102019.lif - Expansion_ctrl_63X_zoom2.5_zstack_aver3_cell3.jpg]

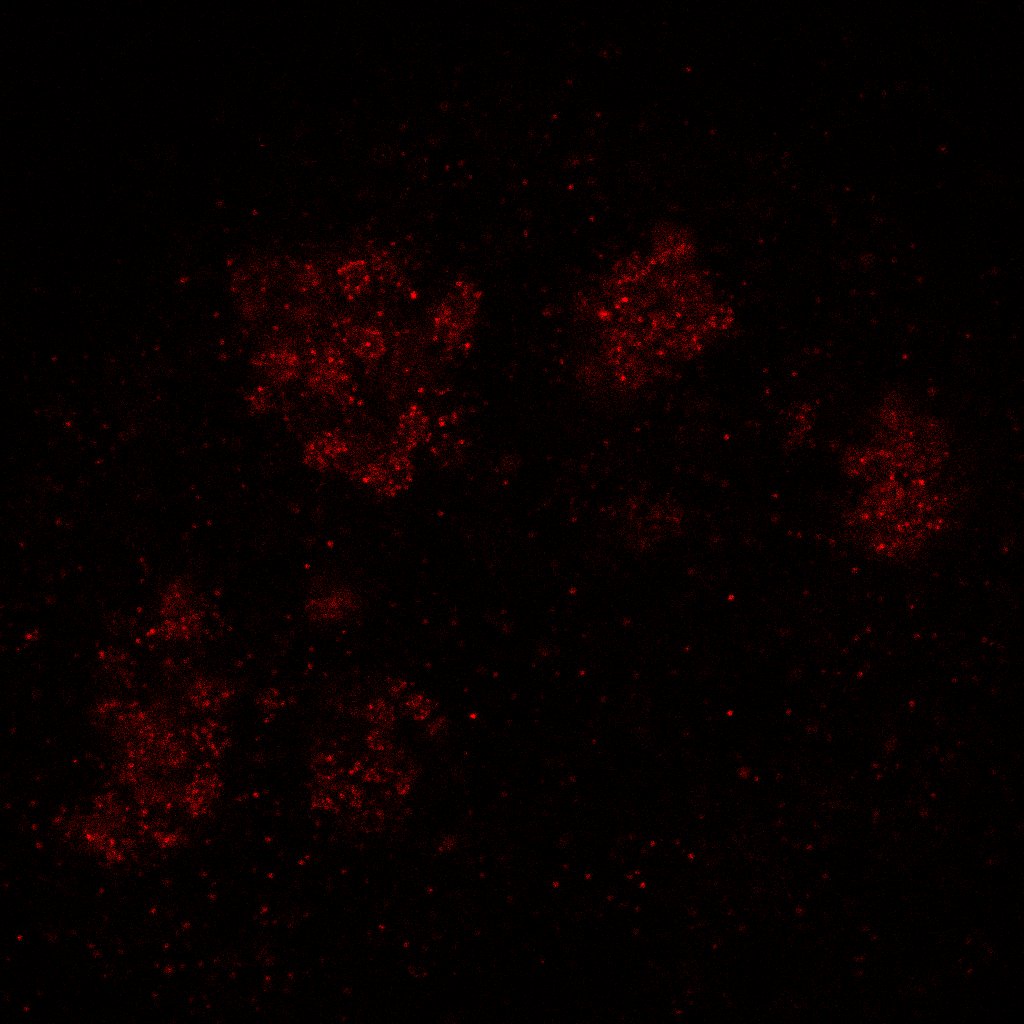

Supplement: Supplementary file 9 — Source data Fig. 1 [file 44318_2024_333_MOESM9_ESM.zip › Figure 1/Figure 1D/CTR/post expansion/Expansion_nucleole_02102019.lif - Expansion_ctrl_63X_zoom2.5_zstack_aver3_cell3_red.jpg]

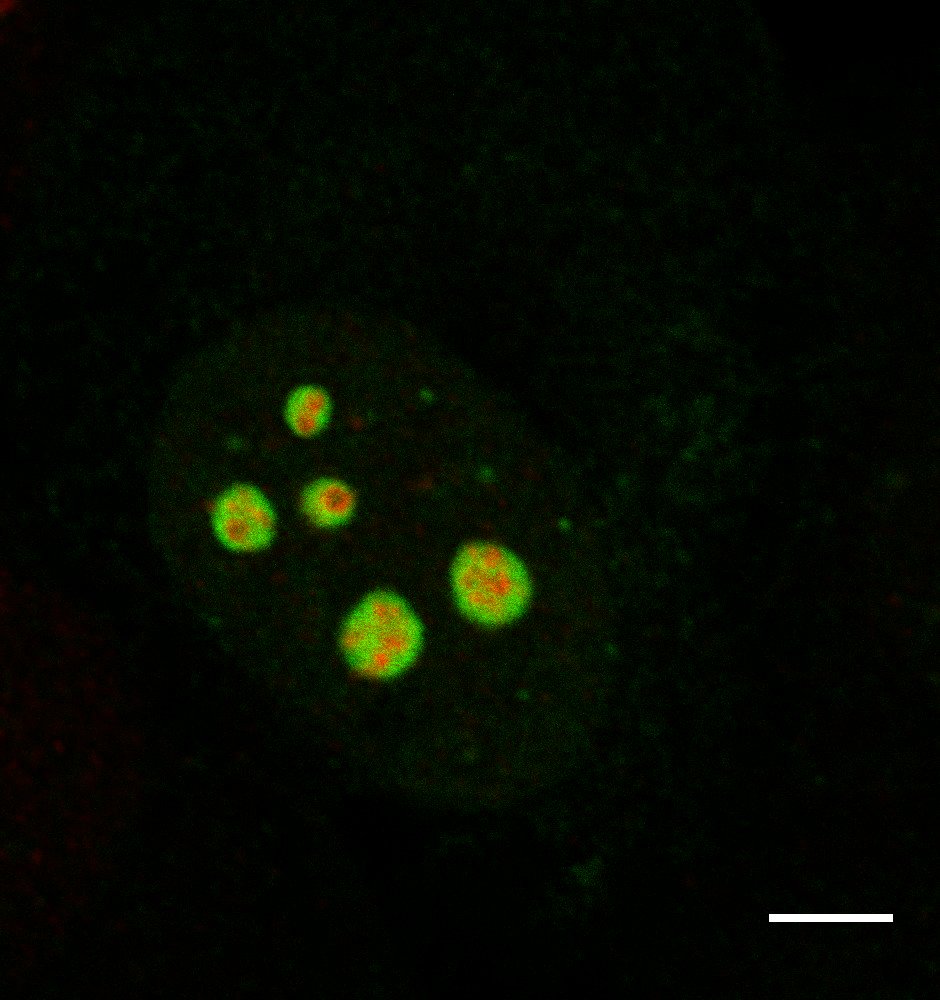

Supplement: Supplementary file 9 — Source data Fig. 1 [file 44318_2024_333_MOESM9_ESM.zip › Figure 1/Figure 1D/CTR/pre expansion/Composite.jpg]

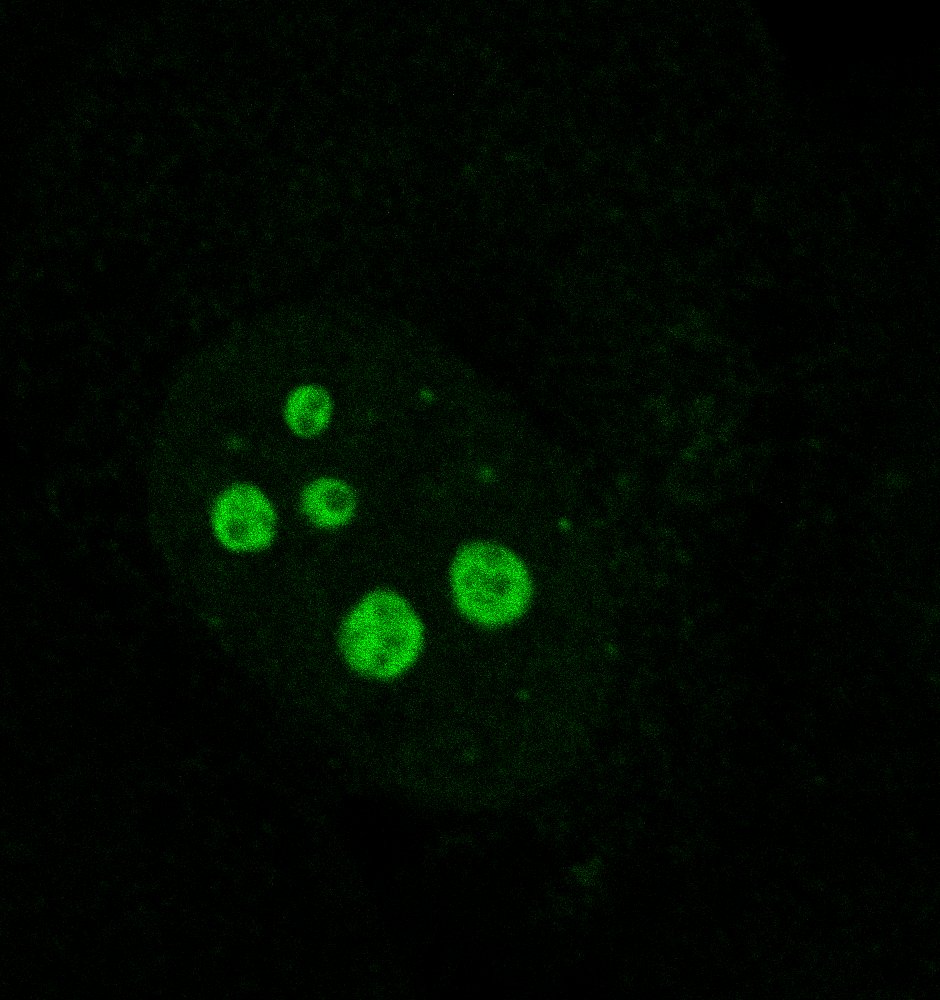

Supplement: Supplementary file 9 — Source data Fig. 1 [file 44318_2024_333_MOESM9_ESM.zip › Figure 1/Figure 1D/CTR/pre expansion/NonExpansion_nucleole_02102019.lif - NonExpansion_Ctrl_63X_zoom6_1plan_aver3_cell1.jpg]

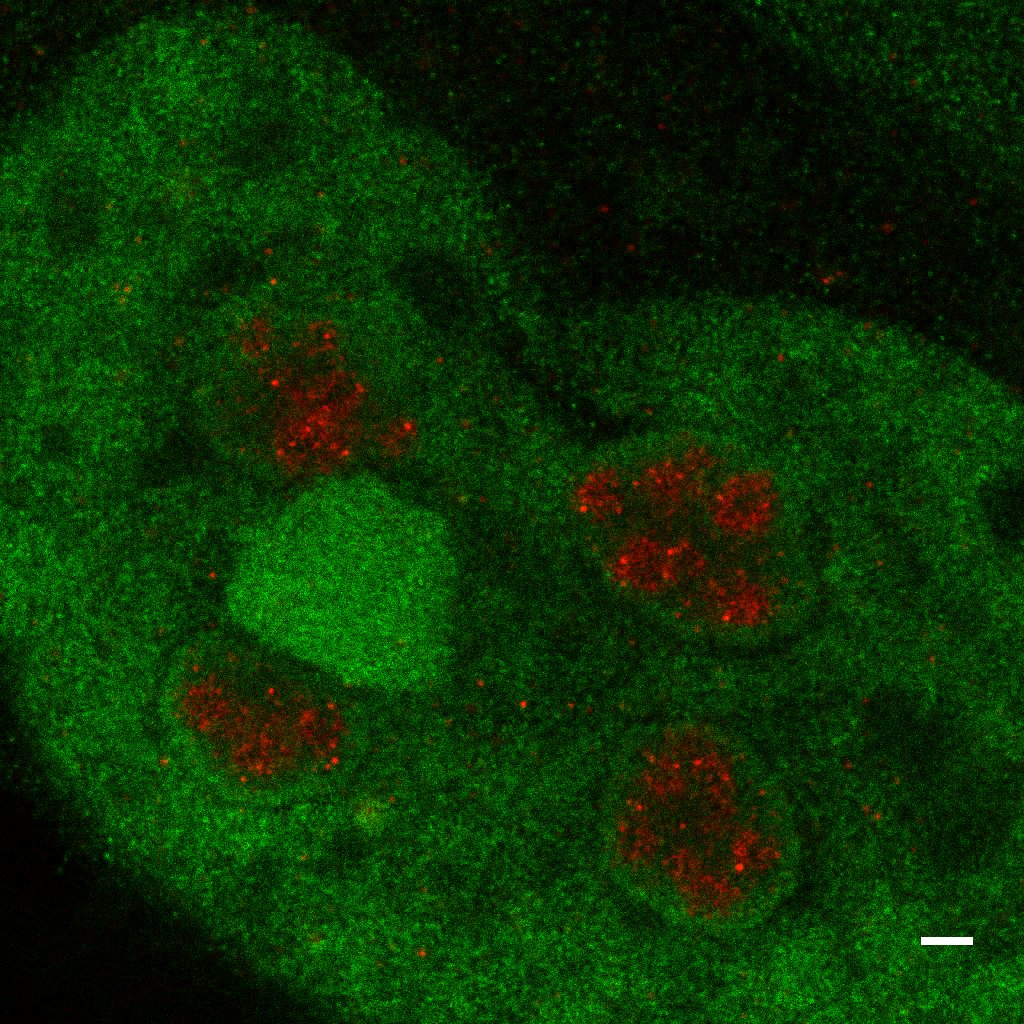

Supplement: Supplementary file 9 — Source data Fig. 1 [file 44318_2024_333_MOESM9_ESM.zip › Figure 1/Figure 1D/MG132/Post Expansion/Composite.jpg]

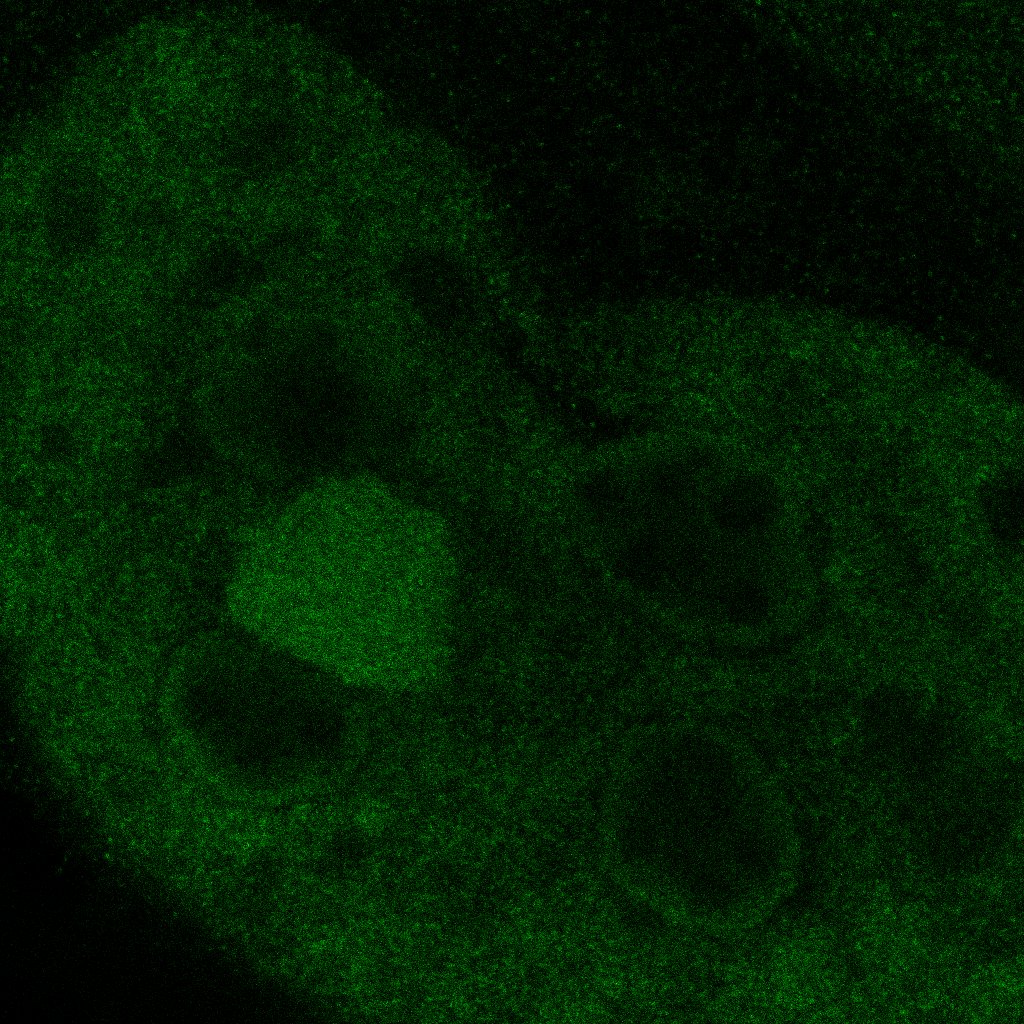

Supplement: Supplementary file 9 — Source data Fig. 1 [file 44318_2024_333_MOESM9_ESM.zip › Figure 1/Figure 1D/MG132/Post Expansion/Expansion_nucleole_02102019.lif - Expansion_MG132_63X_zoom2.5_1plan_aver3_cell5.jpg]

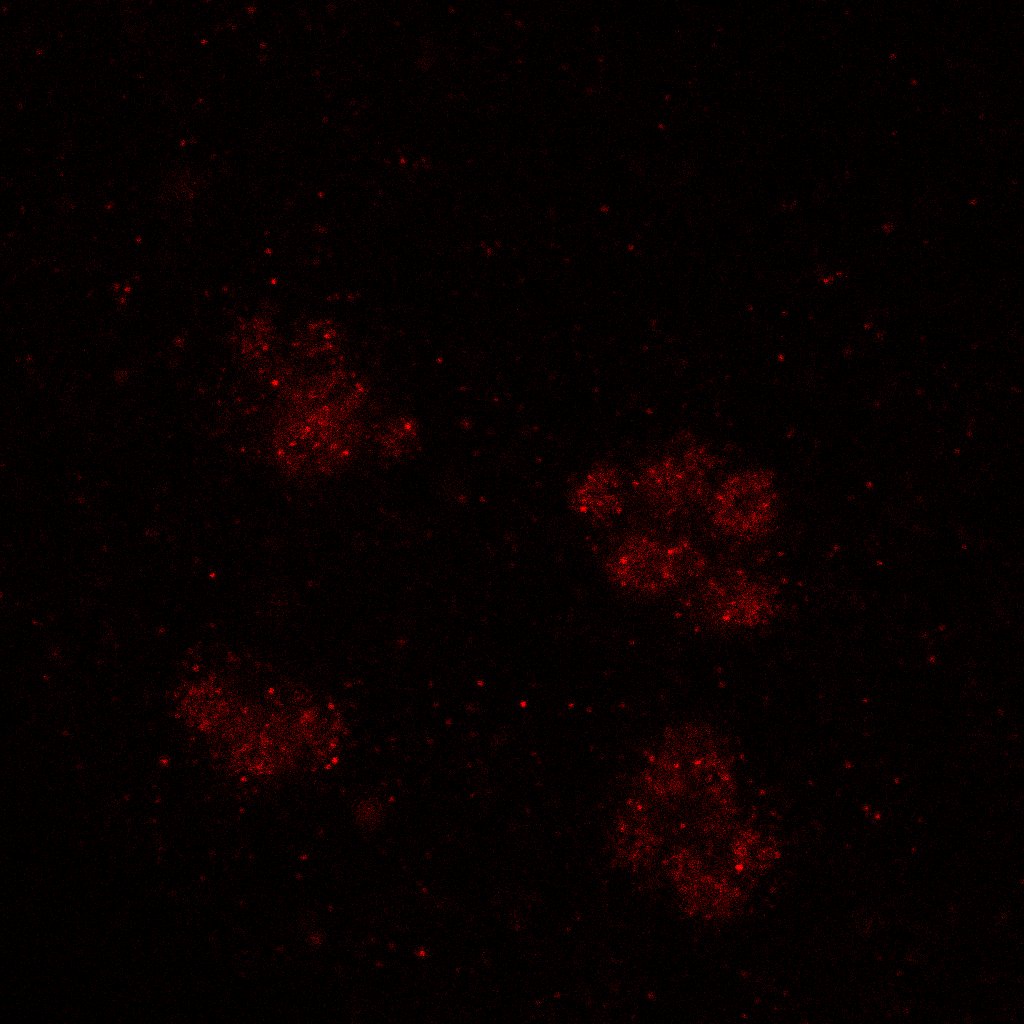

Supplement: Supplementary file 9 — Source data Fig. 1 [file 44318_2024_333_MOESM9_ESM.zip › Figure 1/Figure 1D/MG132/Post Expansion/Expansion_nucleole_02102019.lif - Expansion_MG132_63X_zoom2.5_1plan_aver3_cell5_red.jpg]

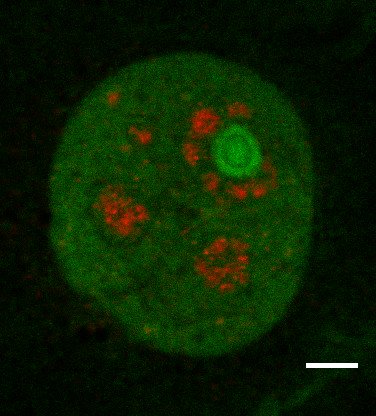

Supplement: Supplementary file 9 — Source data Fig. 1 [file 44318_2024_333_MOESM9_ESM.zip › Figure 1/Figure 1D/MG132/Pre Expansion/Composite.jpg]

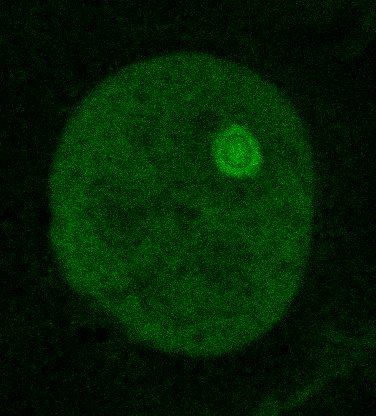

Supplement: Supplementary file 9 — Source data Fig. 1 [file 44318_2024_333_MOESM9_ESM.zip › Figure 1/Figure 1D/MG132/Pre Expansion/NONEXP~1.JPG]

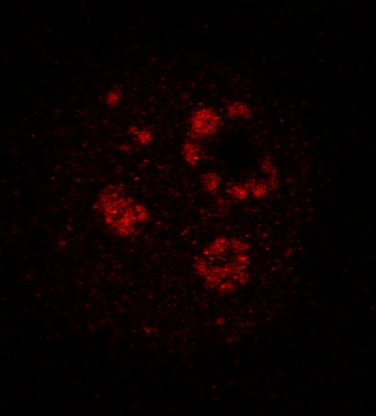

Supplement: Supplementary file 9 — Source data Fig. 1 [file 44318_2024_333_MOESM9_ESM.zip › Figure 1/Figure 1D/MG132/Pre Expansion/NONEXP~2.JPG]

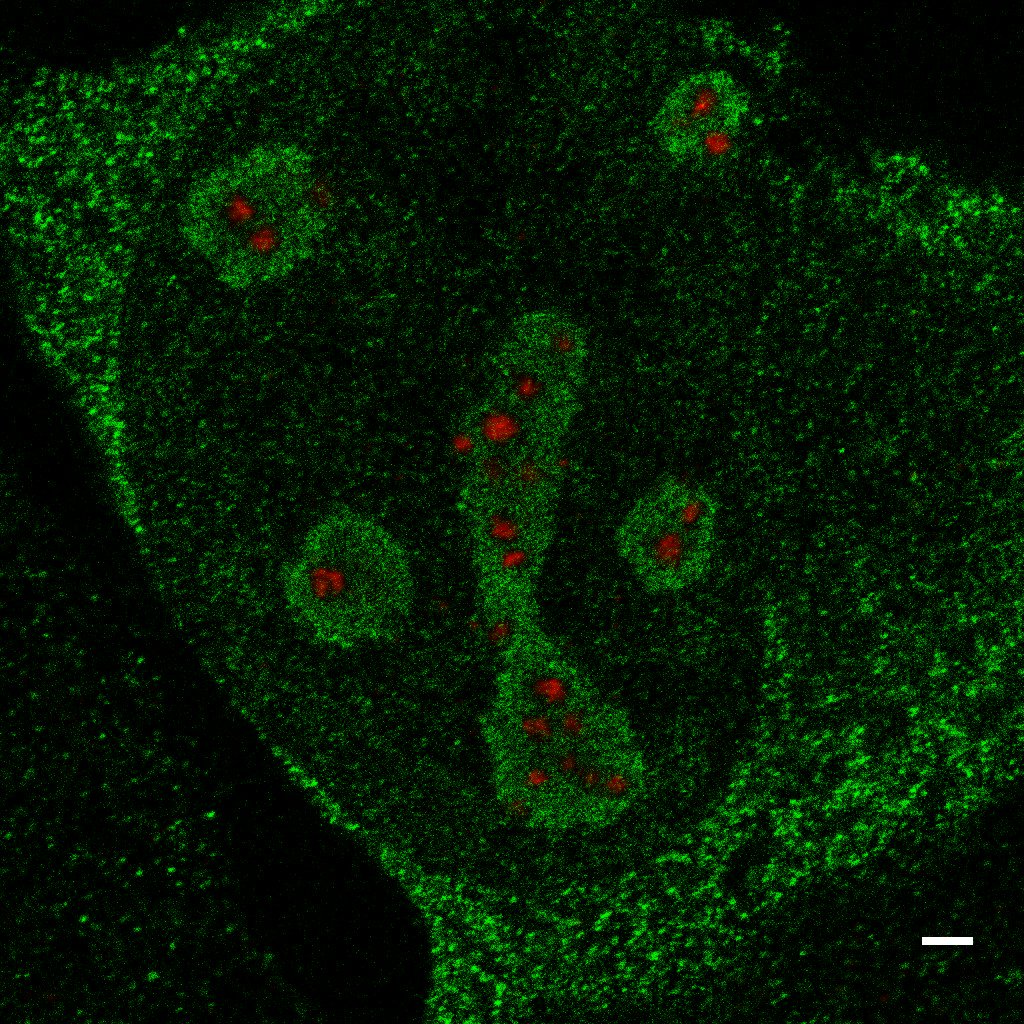

Supplement: Supplementary file 9 — Source data Fig. 1 [file 44318_2024_333_MOESM9_ESM.zip › Figure 1/Figure 1E/CTR/Post expansion/Composite.jpg]

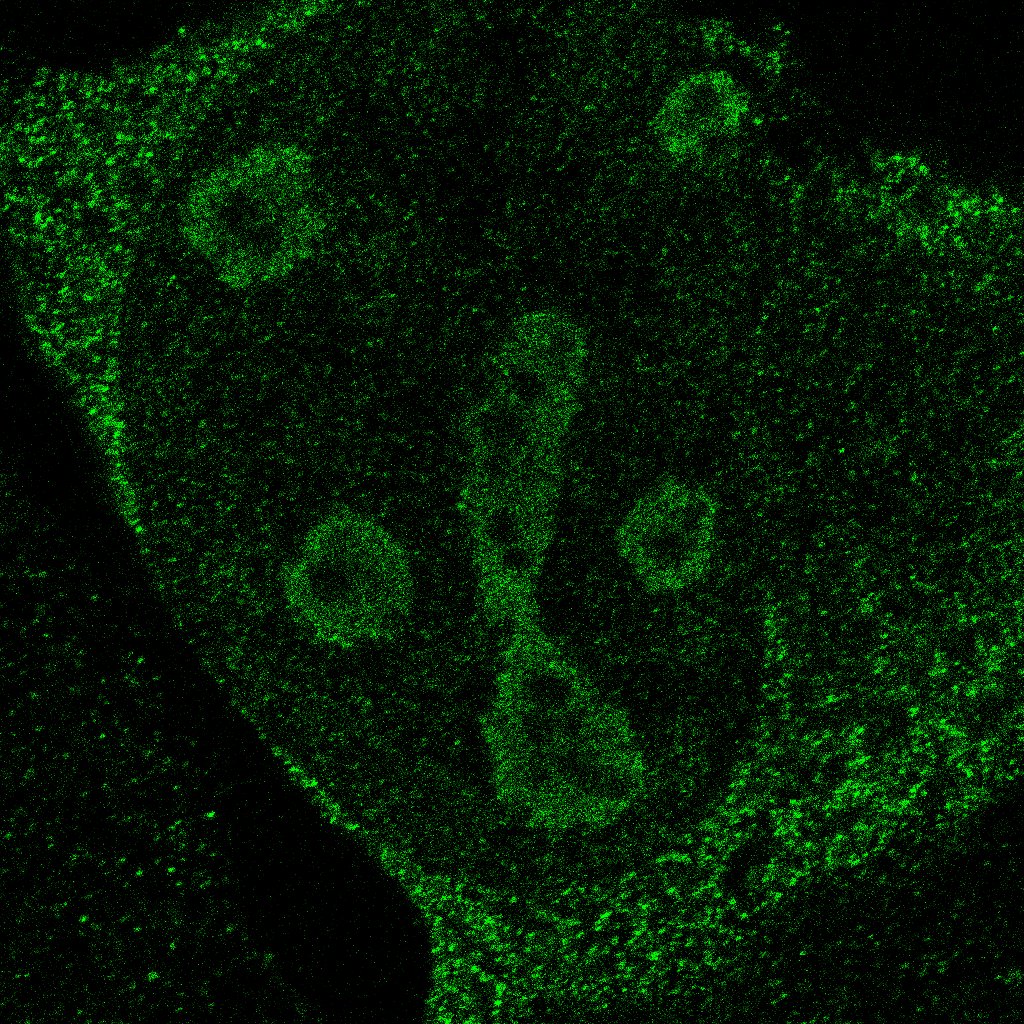

Supplement: Supplementary file 9 — Source data Fig. 1 [file 44318_2024_333_MOESM9_ESM.zip › Figure 1/Figure 1E/CTR/Post expansion/Expansion_nucleole_20200530.lif - Nonstressed_zoom2.5_cell12_green.jpg]

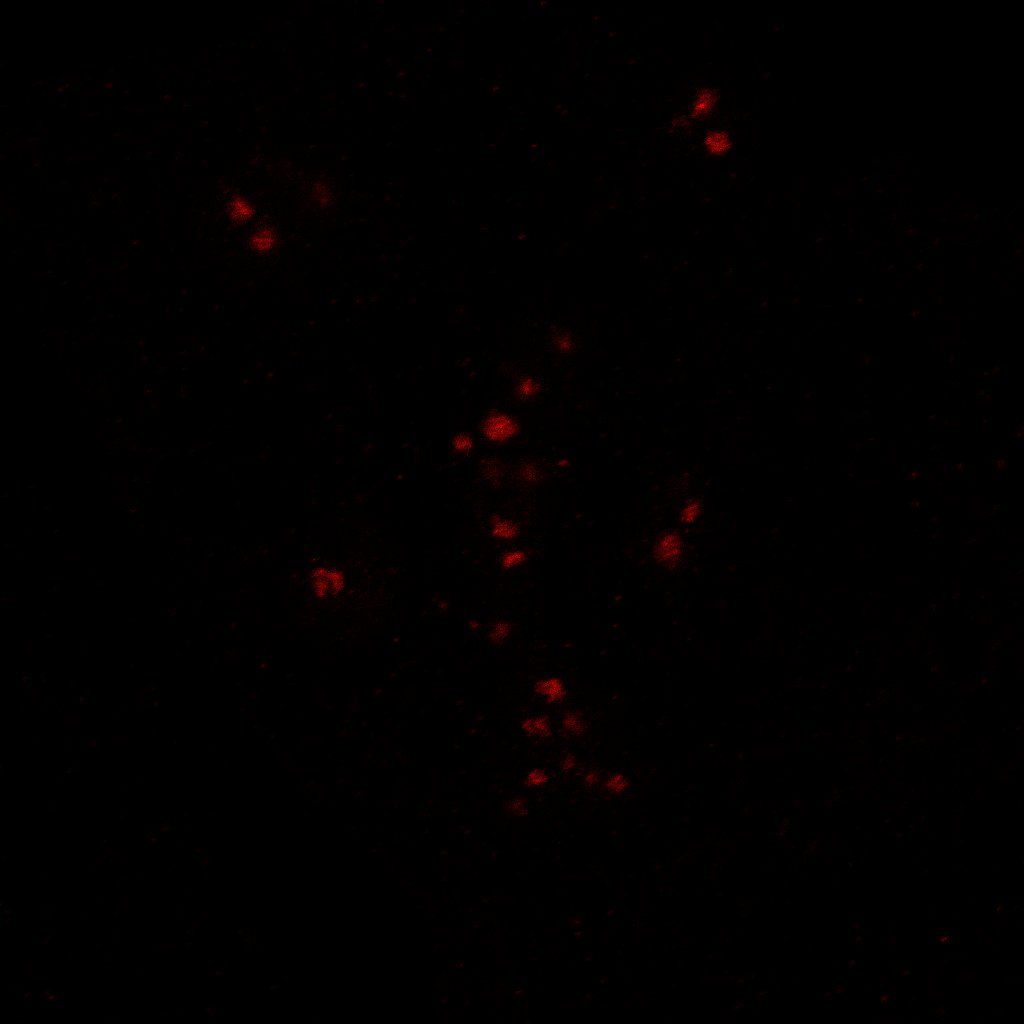

Supplement: Supplementary file 9 — Source data Fig. 1 [file 44318_2024_333_MOESM9_ESM.zip › Figure 1/Figure 1E/CTR/Post expansion/Expansion_nucleole_20200530.lif - Nonstressed_zoom2.5_cell12_red.jpg]

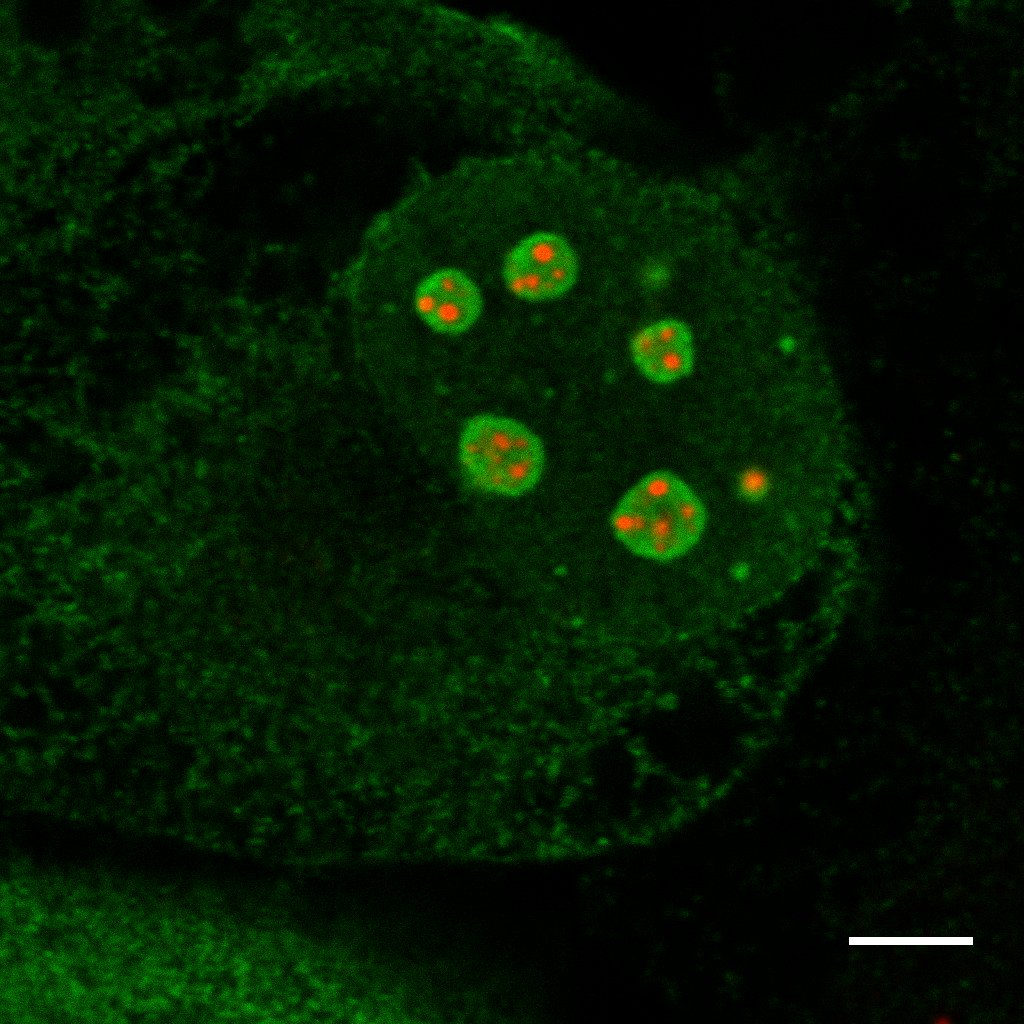

Supplement: Supplementary file 9 — Source data Fig. 1 [file 44318_2024_333_MOESM9_ESM.zip › Figure 1/Figure 1E/CTR/Pre expansion/Composite.jpg]

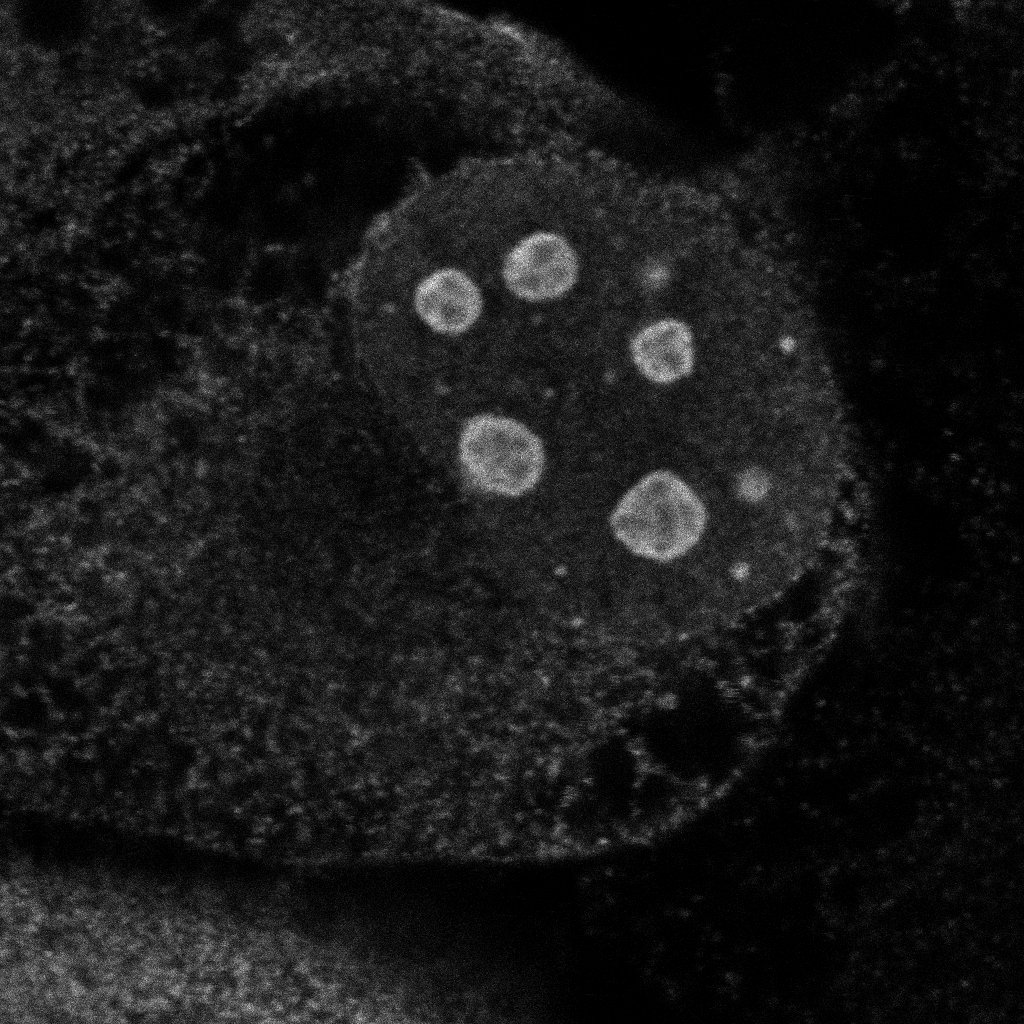

Supplement: Supplementary file 9 — Source data Fig. 1 [file 44318_2024_333_MOESM9_ESM.zip › Figure 1/Figure 1E/CTR/Pre expansion/NonExpansion_nucleole_20200530.lif - non-stressed-zoom6_cell3 - C=0.jpg]

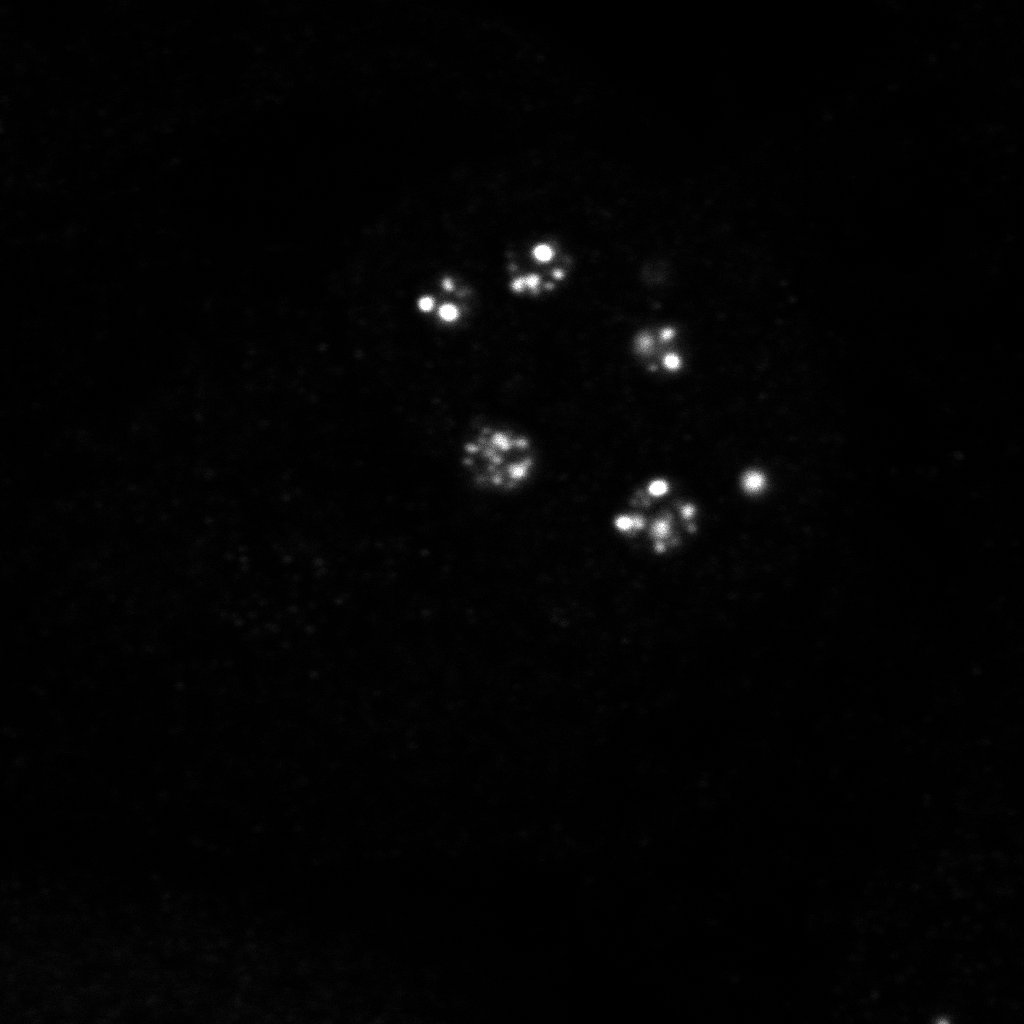

Supplement: Supplementary file 9 — Source data Fig. 1 [file 44318_2024_333_MOESM9_ESM.zip › Figure 1/Figure 1E/CTR/Pre expansion/NonExpansion_nucleole_20200530.lif - non-stressed-zoom6_cell3 - C=1.jpg]

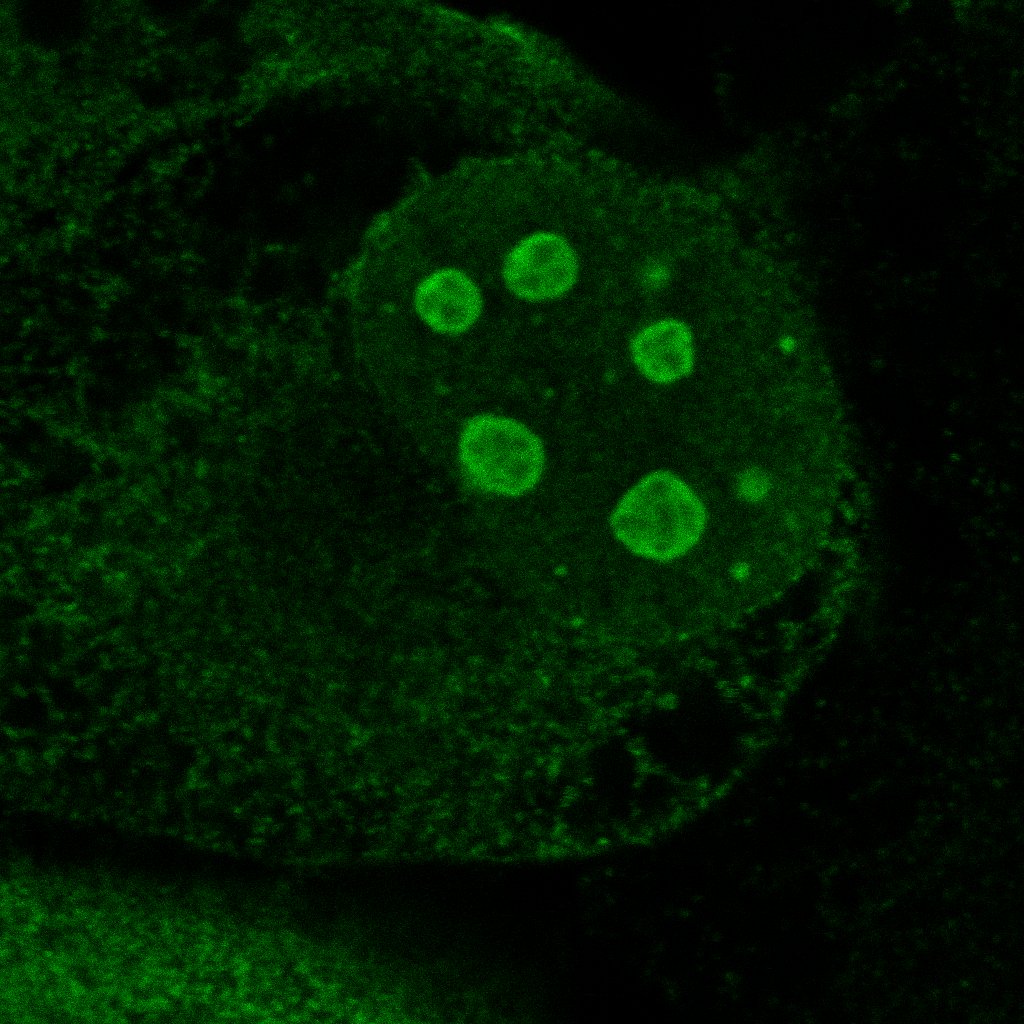

Supplement: Supplementary file 9 — Source data Fig. 1 [file 44318_2024_333_MOESM9_ESM.zip › Figure 1/Figure 1E/CTR/Pre expansion/NonExpansion_nucleole_20200530.lif - non-stressed-zoom6_cell3.jpg]

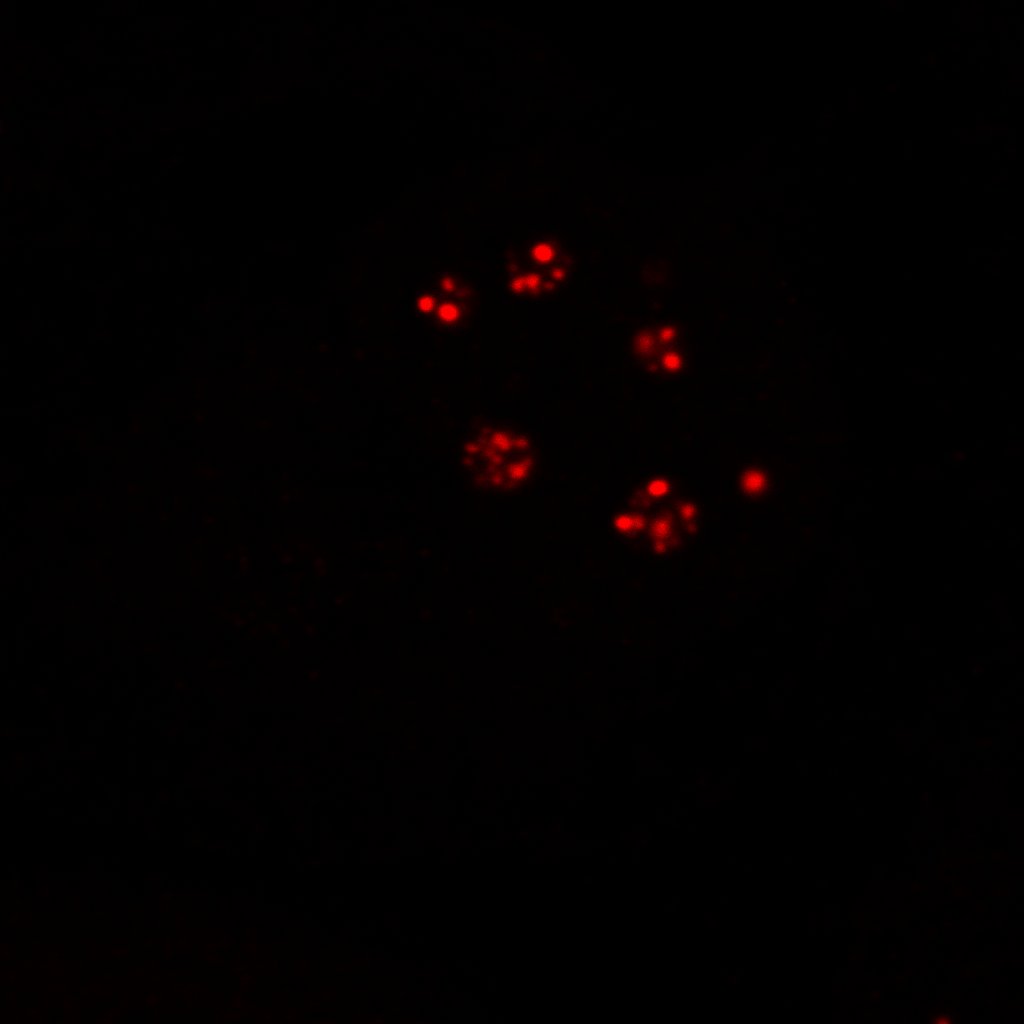

Supplement: Supplementary file 9 — Source data Fig. 1 [file 44318_2024_333_MOESM9_ESM.zip › Figure 1/Figure 1E/CTR/Pre expansion/NonExpansion_nucleole_20200530.lif - non-stressed-zoom6_cell3_red.jpg]

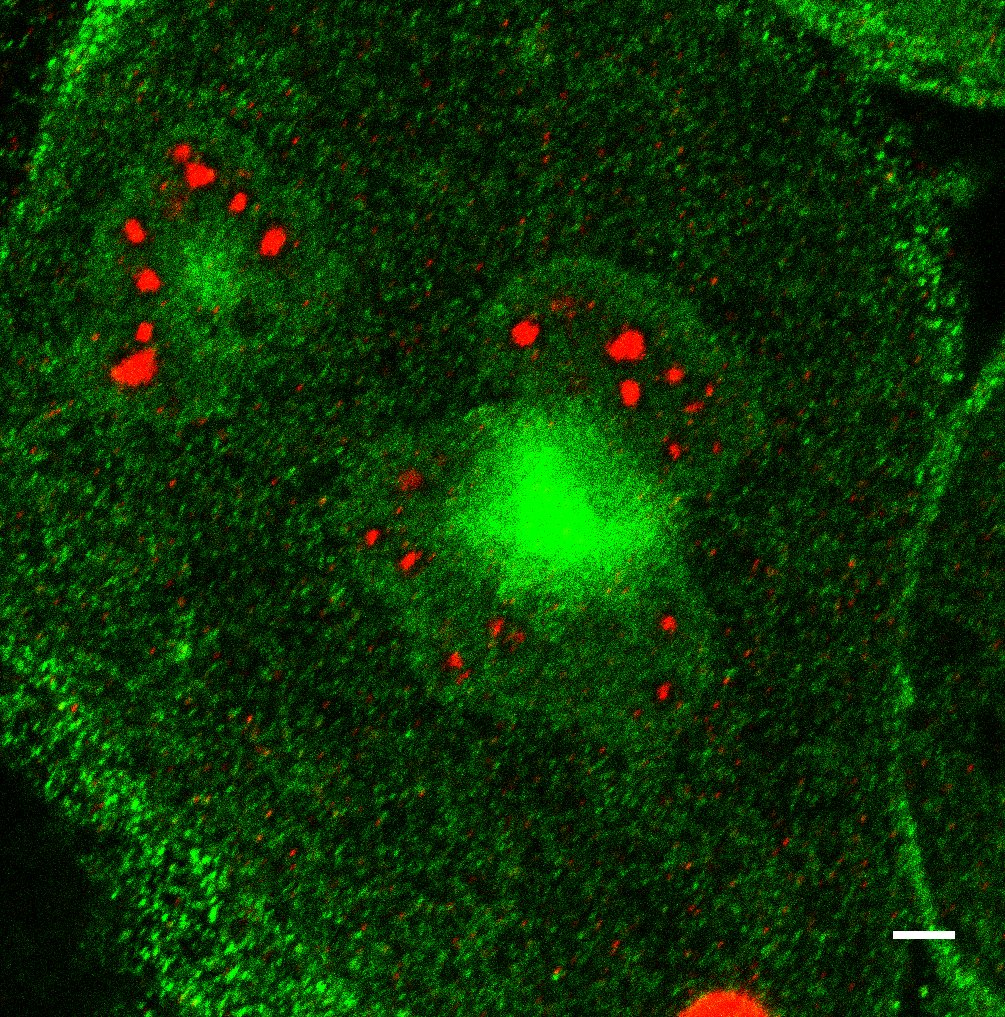

Supplement: Supplementary file 9 — Source data Fig. 1 [file 44318_2024_333_MOESM9_ESM.zip › Figure 1/Figure 1E/MG132/Post-expansion/Composite.jpg]

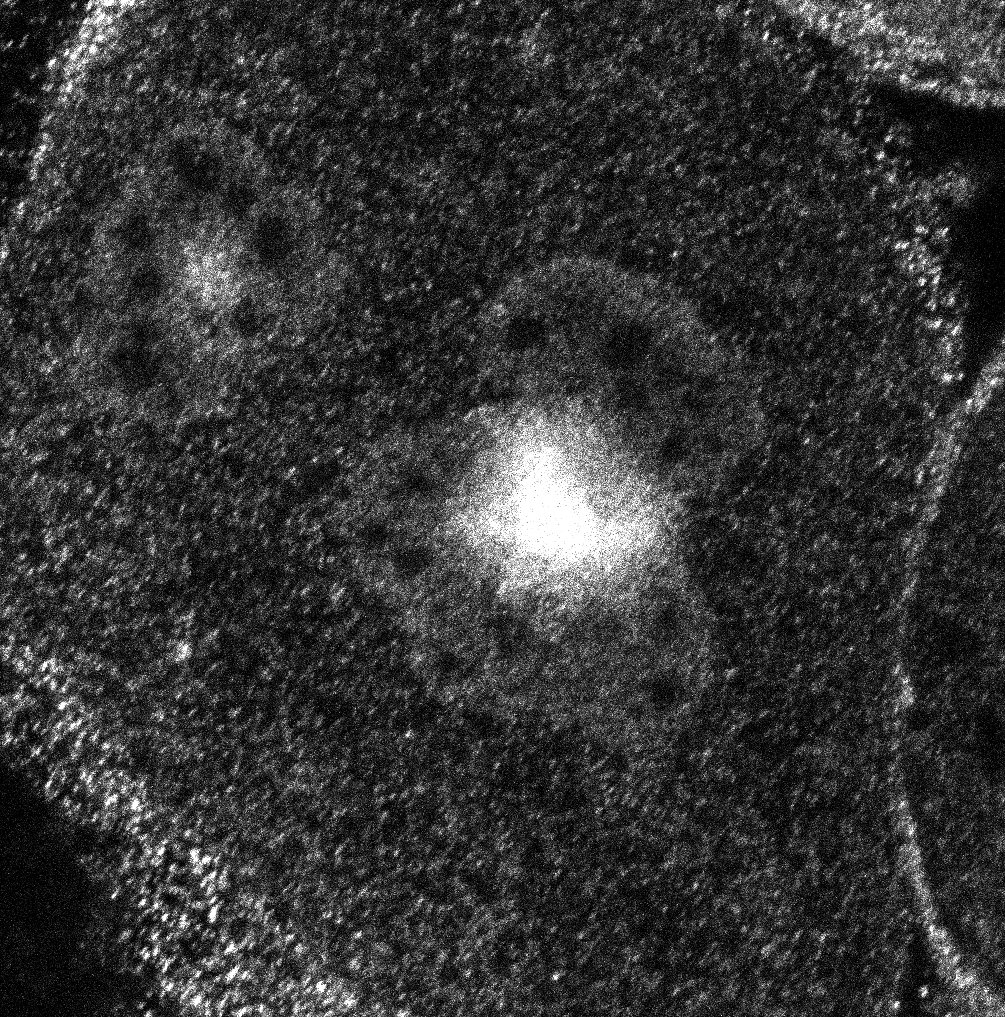

Supplement: Supplementary file 9 — Source data Fig. 1 [file 44318_2024_333_MOESM9_ESM.zip › Figure 1/Figure 1E/MG132/Post-expansion/Expansion_nucleole_stressed_20200530.lif - C=0.jpg]

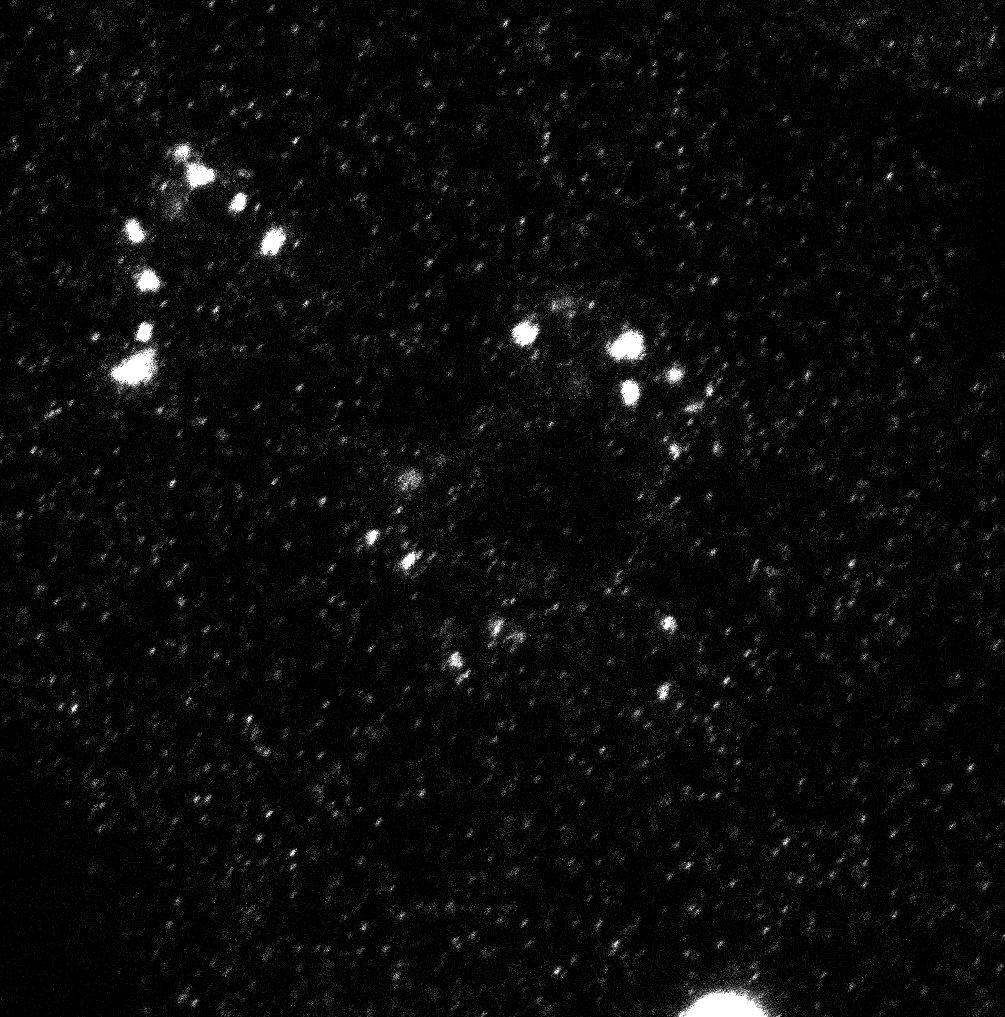

Supplement: Supplementary file 9 — Source data Fig. 1 [file 44318_2024_333_MOESM9_ESM.zip › Figure 1/Figure 1E/MG132/Post-expansion/Expansion_nucleole_stressed_20200530.lif - C=1.jpg]

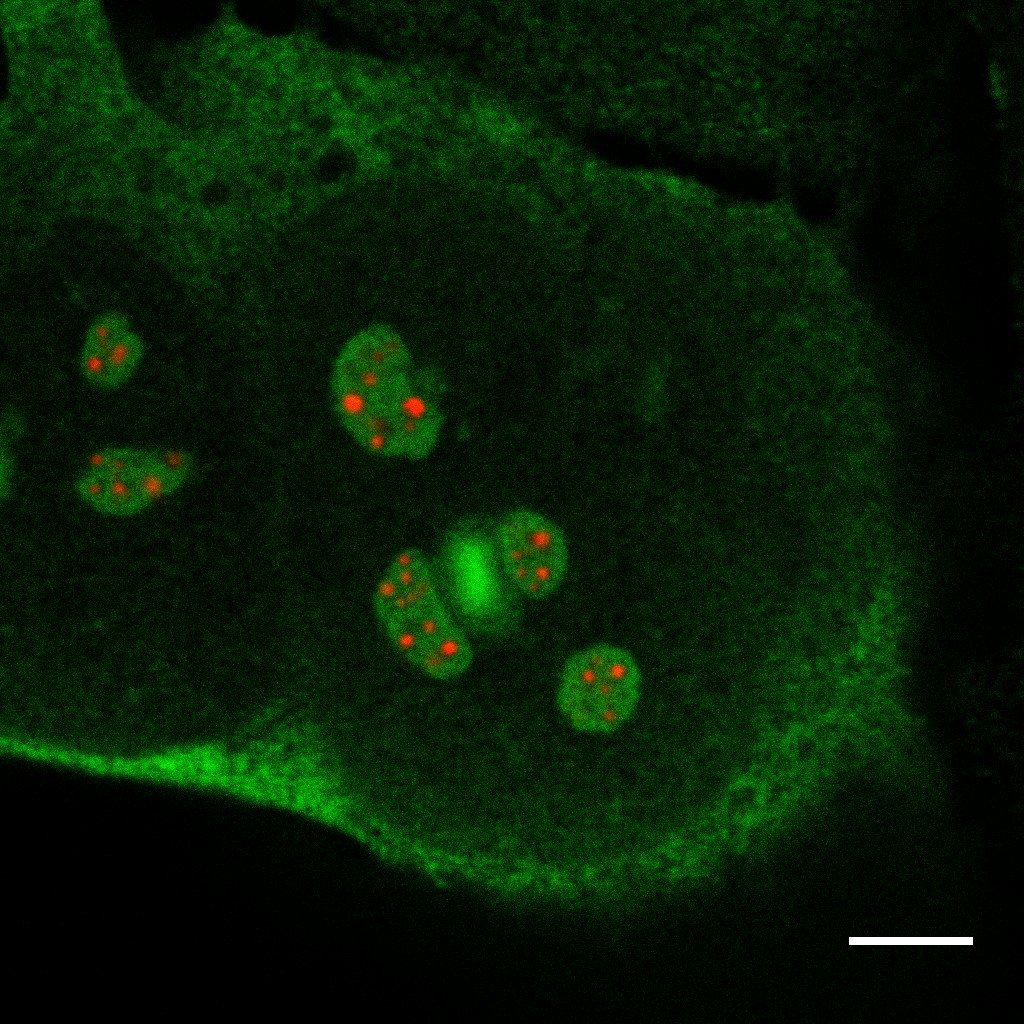

Supplement: Supplementary file 9 — Source data Fig. 1 [file 44318_2024_333_MOESM9_ESM.zip › Figure 1/Figure 1E/MG132/Pre-expansion/Composite.jpg]

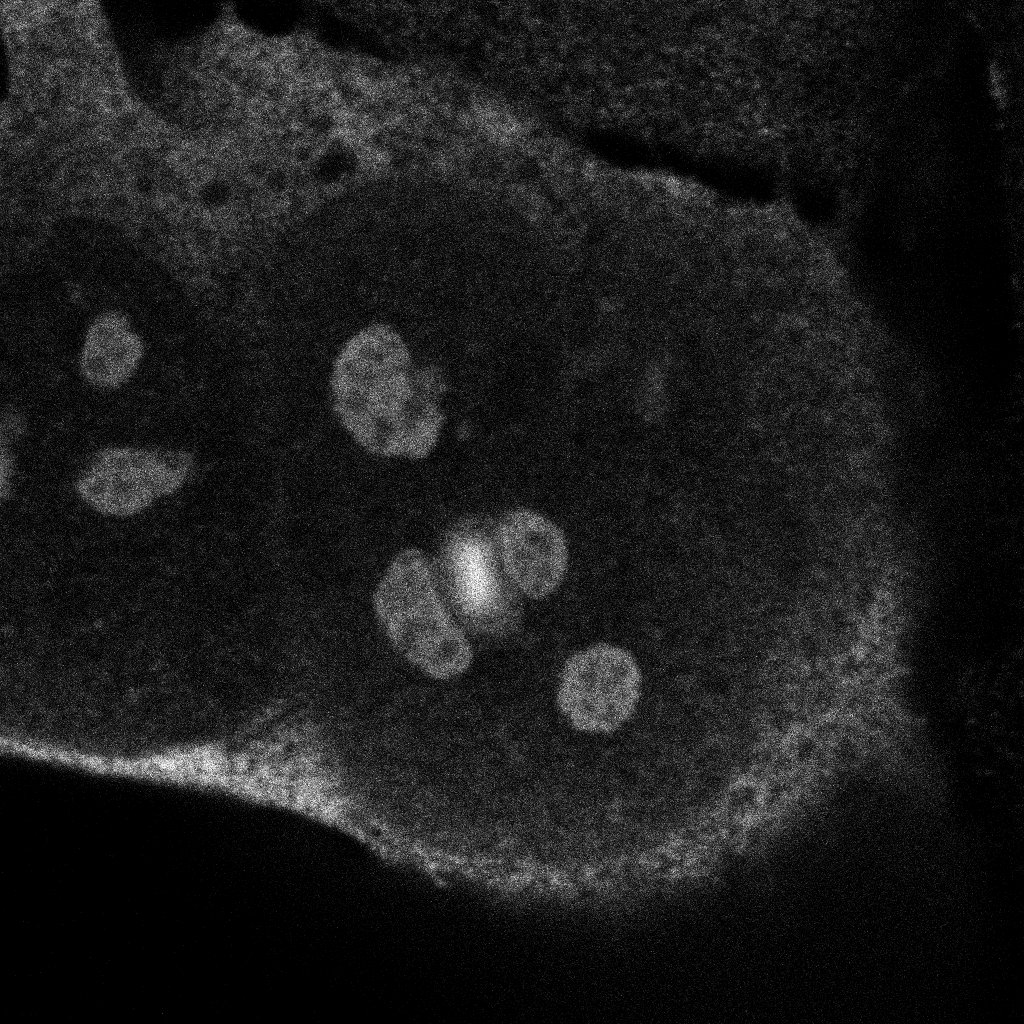

Supplement: Supplementary file 9 — Source data Fig. 1 [file 44318_2024_333_MOESM9_ESM.zip › Figure 1/Figure 1E/MG132/Pre-expansion/NonExpansion_nucleole_20200530.lif - stressed_zoom6_cell1_zstack - C=0.jpg]

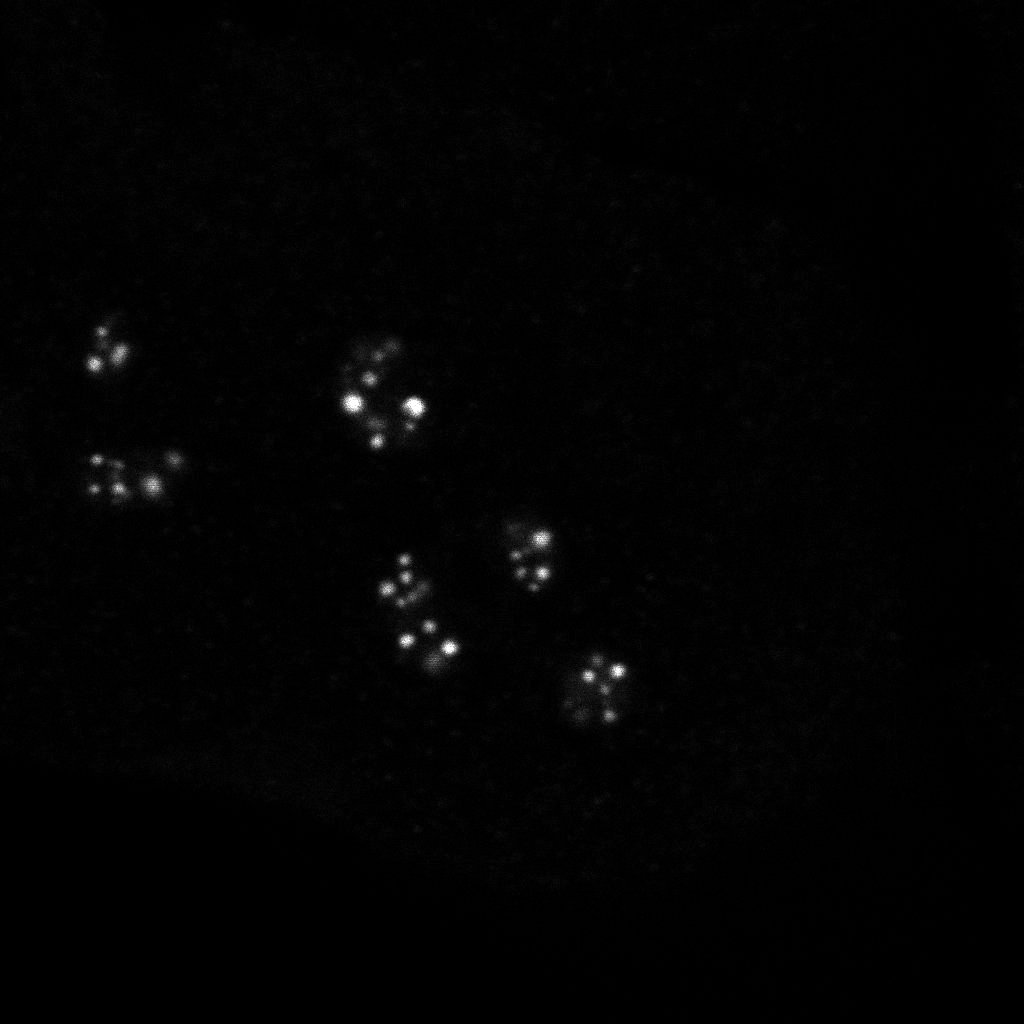

Supplement: Supplementary file 9 — Source data Fig. 1 [file 44318_2024_333_MOESM9_ESM.zip › Figure 1/Figure 1E/MG132/Pre-expansion/NonExpansion_nucleole_20200530.lif - stressed_zoom6_cell1_zstack - C=1.jpg]

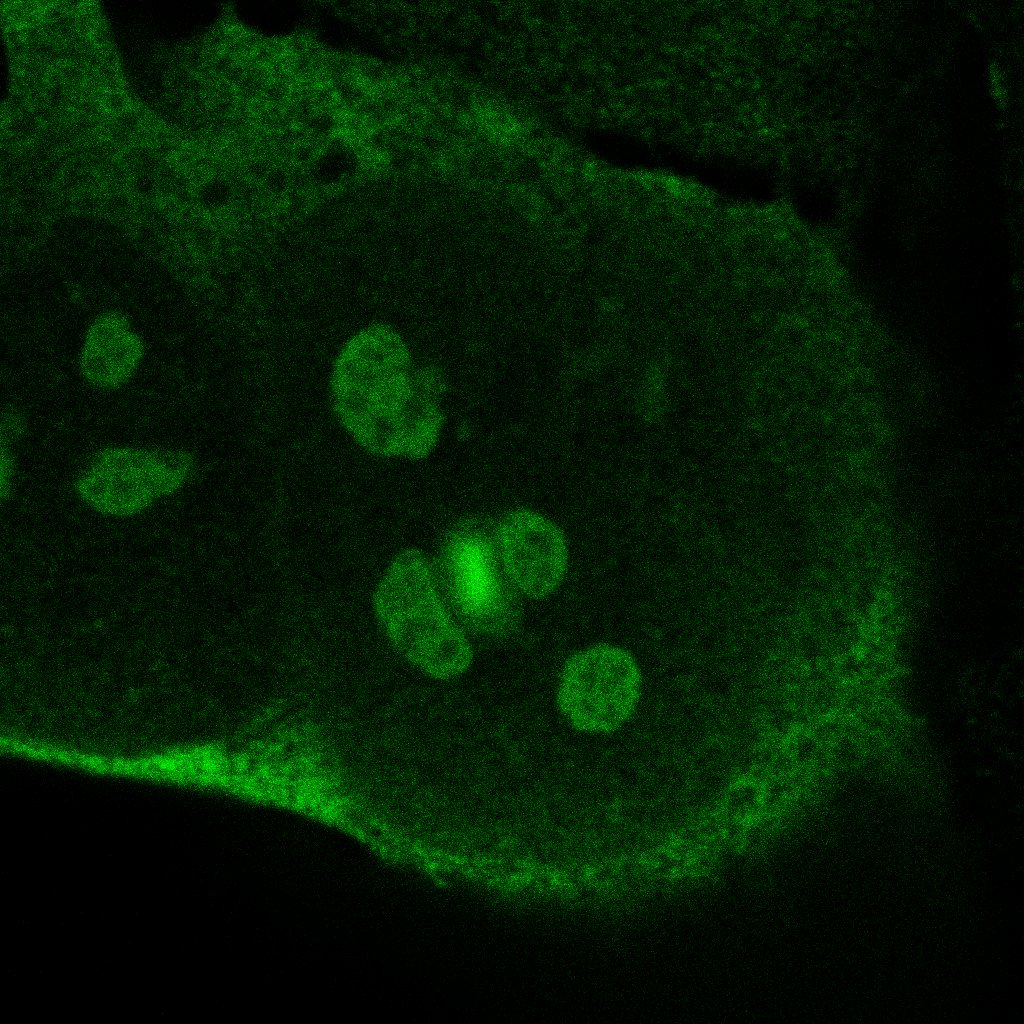

Supplement: Supplementary file 9 — Source data Fig. 1 [file 44318_2024_333_MOESM9_ESM.zip › Figure 1/Figure 1E/MG132/Pre-expansion/NonExpansion_nucleole_20200530.lif - stressed_zoom6_cell1_zstack.jpg]

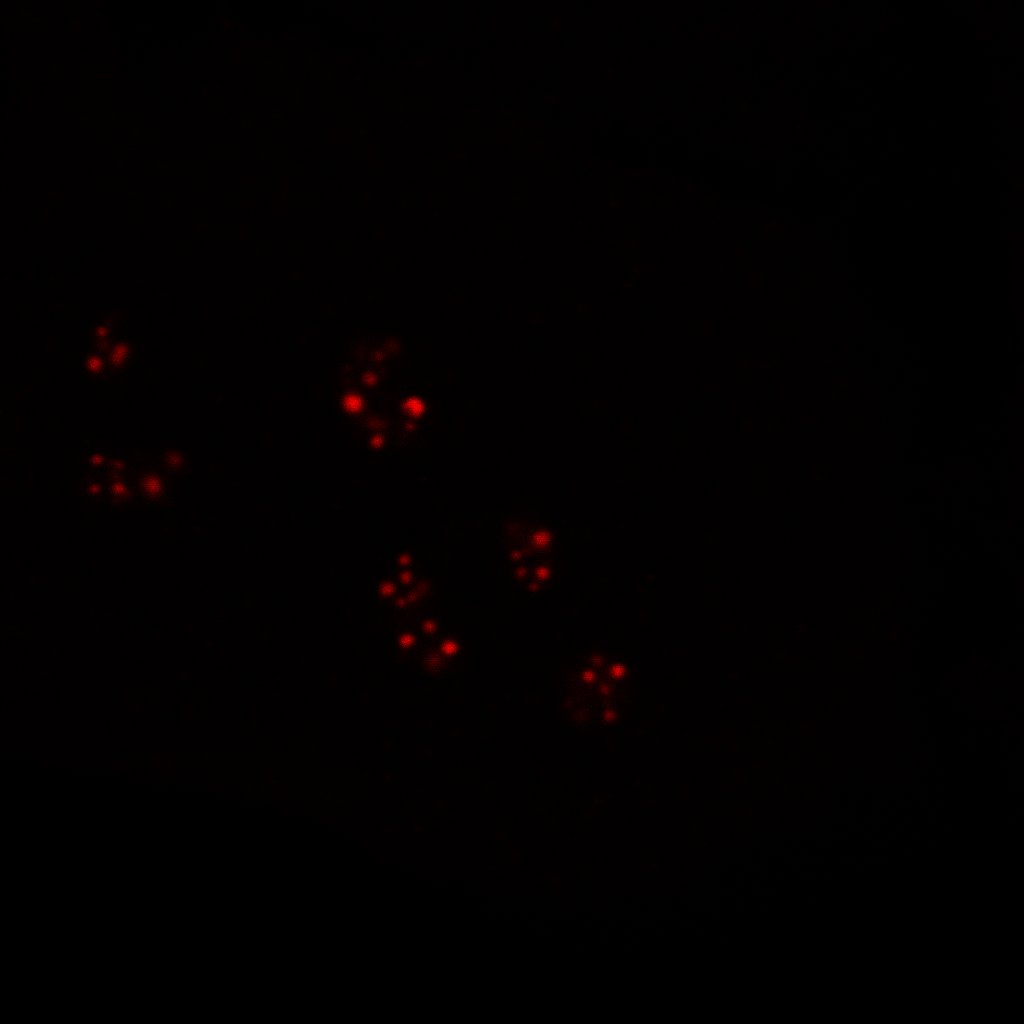

Supplement: Supplementary file 9 — Source data Fig. 1 [file 44318_2024_333_MOESM9_ESM.zip › Figure 1/Figure 1E/MG132/Pre-expansion/NonExpansion_nucleole_20200530.lif - stressed_zoom6_cell1_zstack_red.jpg]

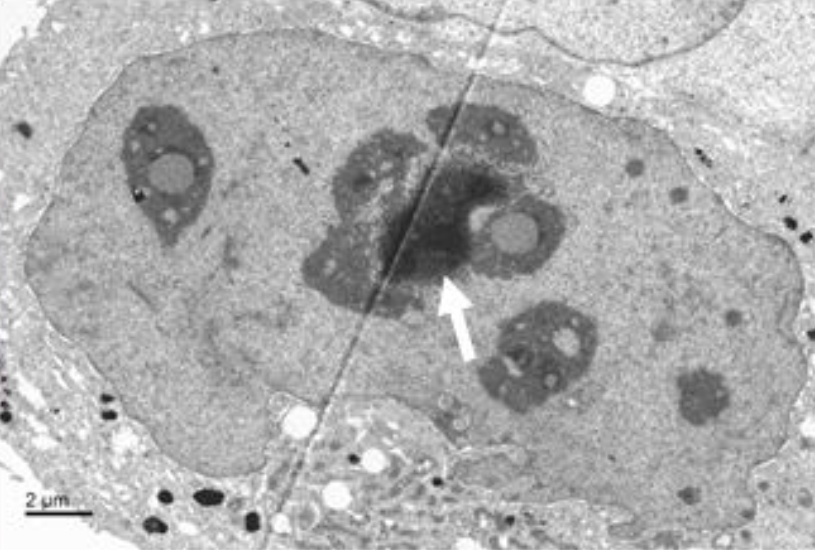

Supplement: Supplementary file 9 — Source data Fig. 1 [file 44318_2024_333_MOESM9_ESM.zip › Figure 1/Figure 1G/Fig.1G_MG132_B.tif]

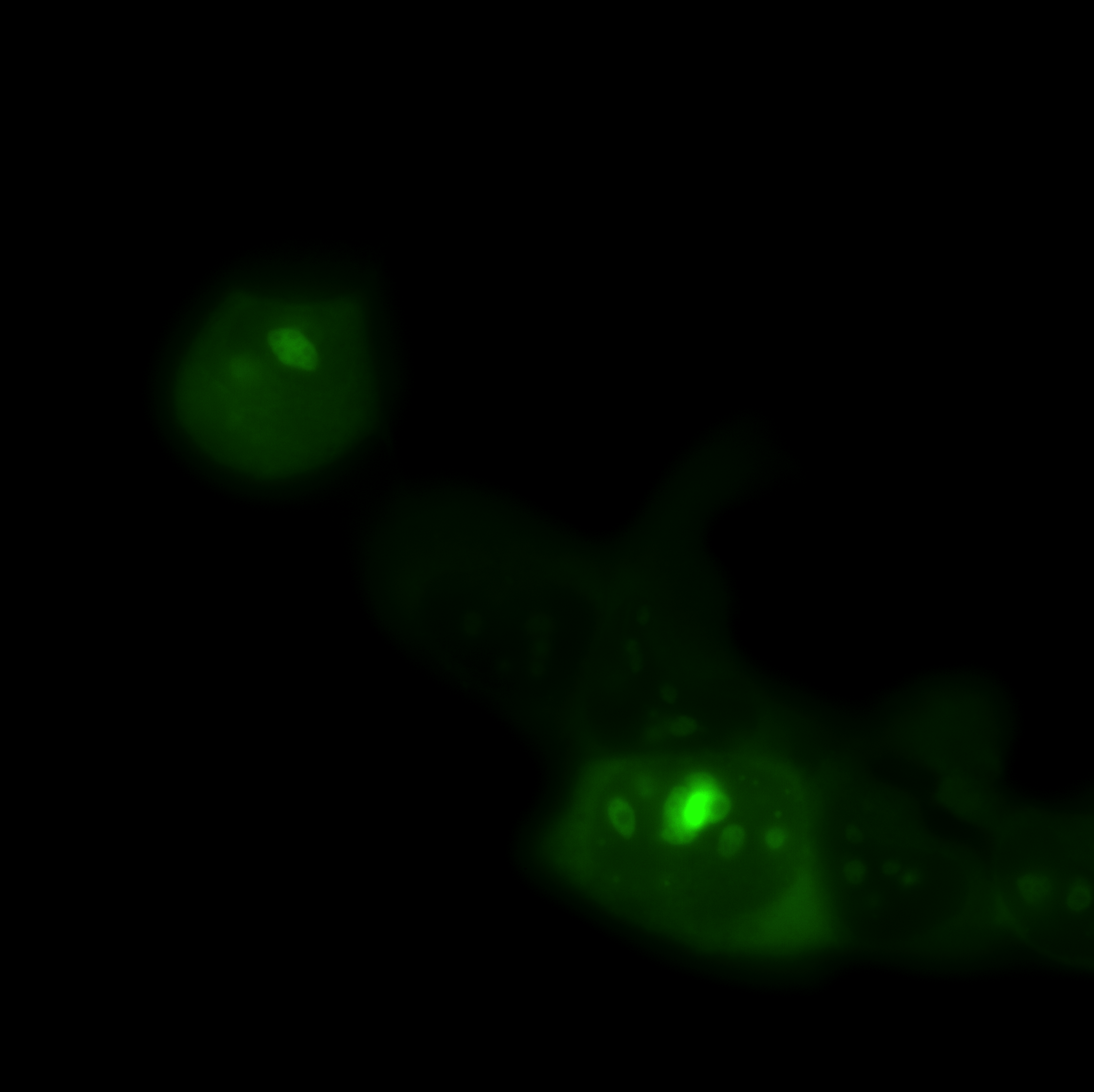

Supplement: Supplementary file 9 — Source data Fig. 1 [file 44318_2024_333_MOESM9_ESM.zip › Figure 1/Figure 1G/Fig.1G_RPL11_MG132_B.tif]

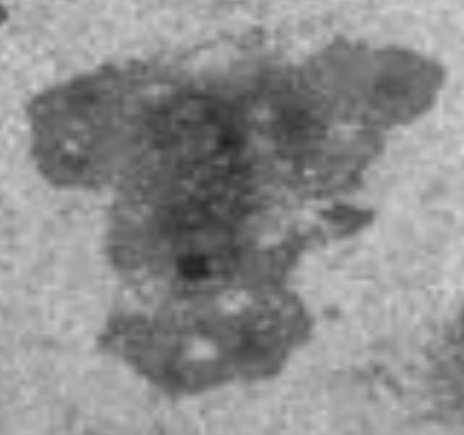

Supplement: Supplementary file 9 — Source data Fig. 1 [file 44318_2024_333_MOESM9_ESM.zip › Figure 1/Figure 1H/Fig.1H_MG132_A.tif]

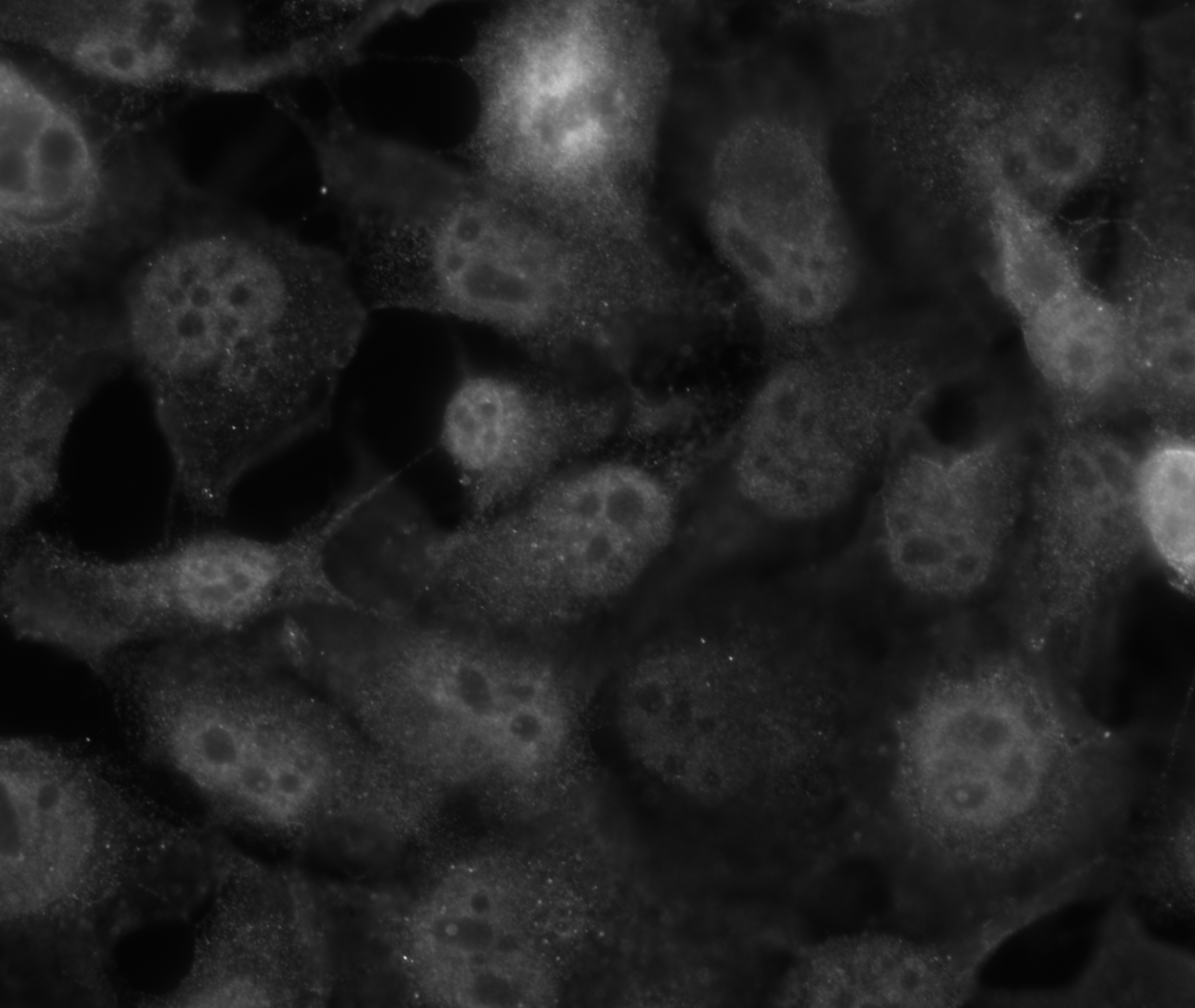

Supplement: Supplementary file 11 — Source data Fig. 3 [file 44318_2024_333_MOESM11_ESM.zip › Figure 3/Figure 3B/CTR/H12 No stress 2.6_w1TexasRed.TIF]

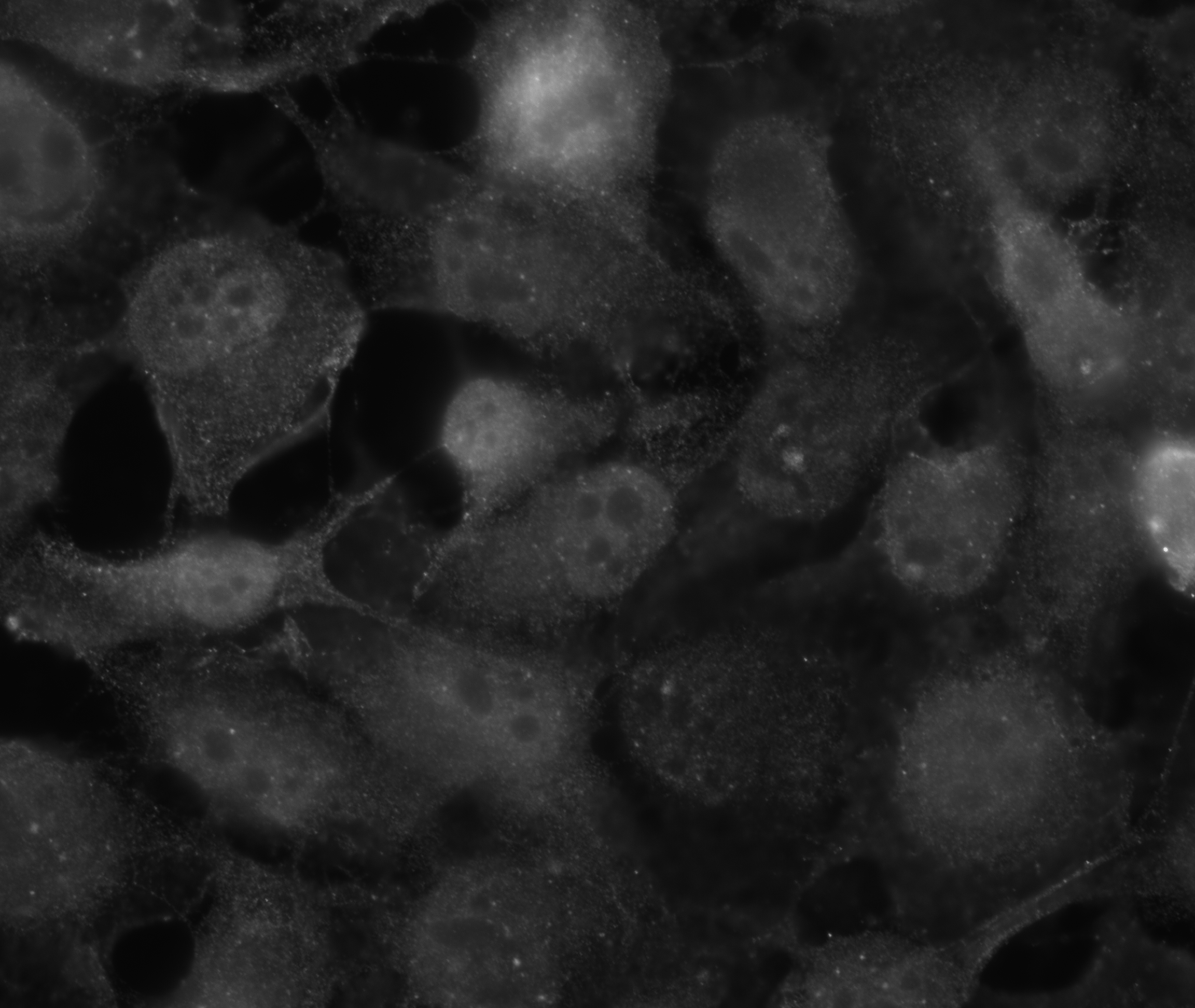

Supplement: Supplementary file 11 — Source data Fig. 3 [file 44318_2024_333_MOESM11_ESM.zip › Figure 3/Figure 3B/CTR/H12 No stress 2.6_w2GFP.TIF]

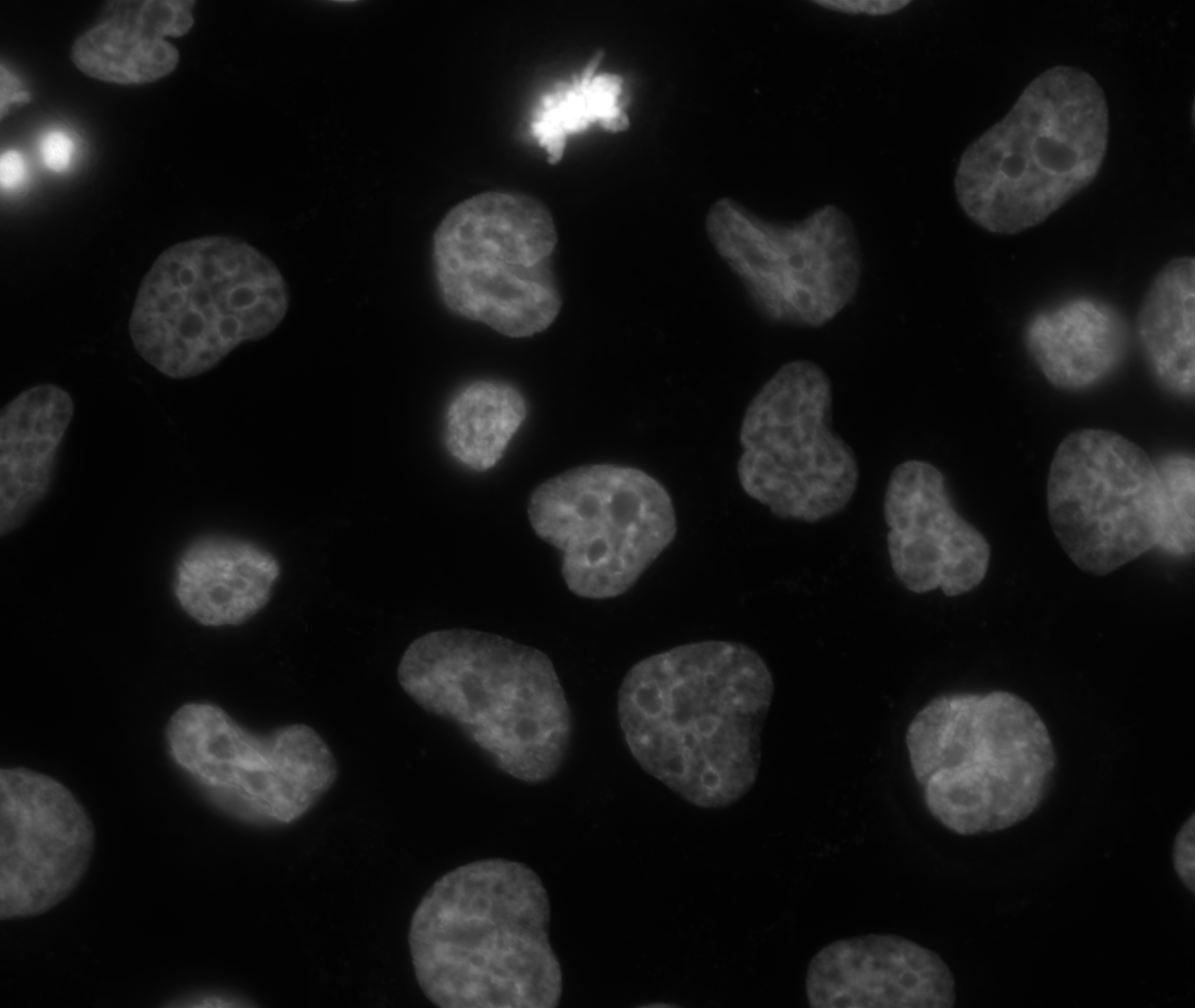

Supplement: Supplementary file 11 — Source data Fig. 3 [file 44318_2024_333_MOESM11_ESM.zip › Figure 3/Figure 3B/CTR/H12 No stress 2.6_w3DAPI.TIF]

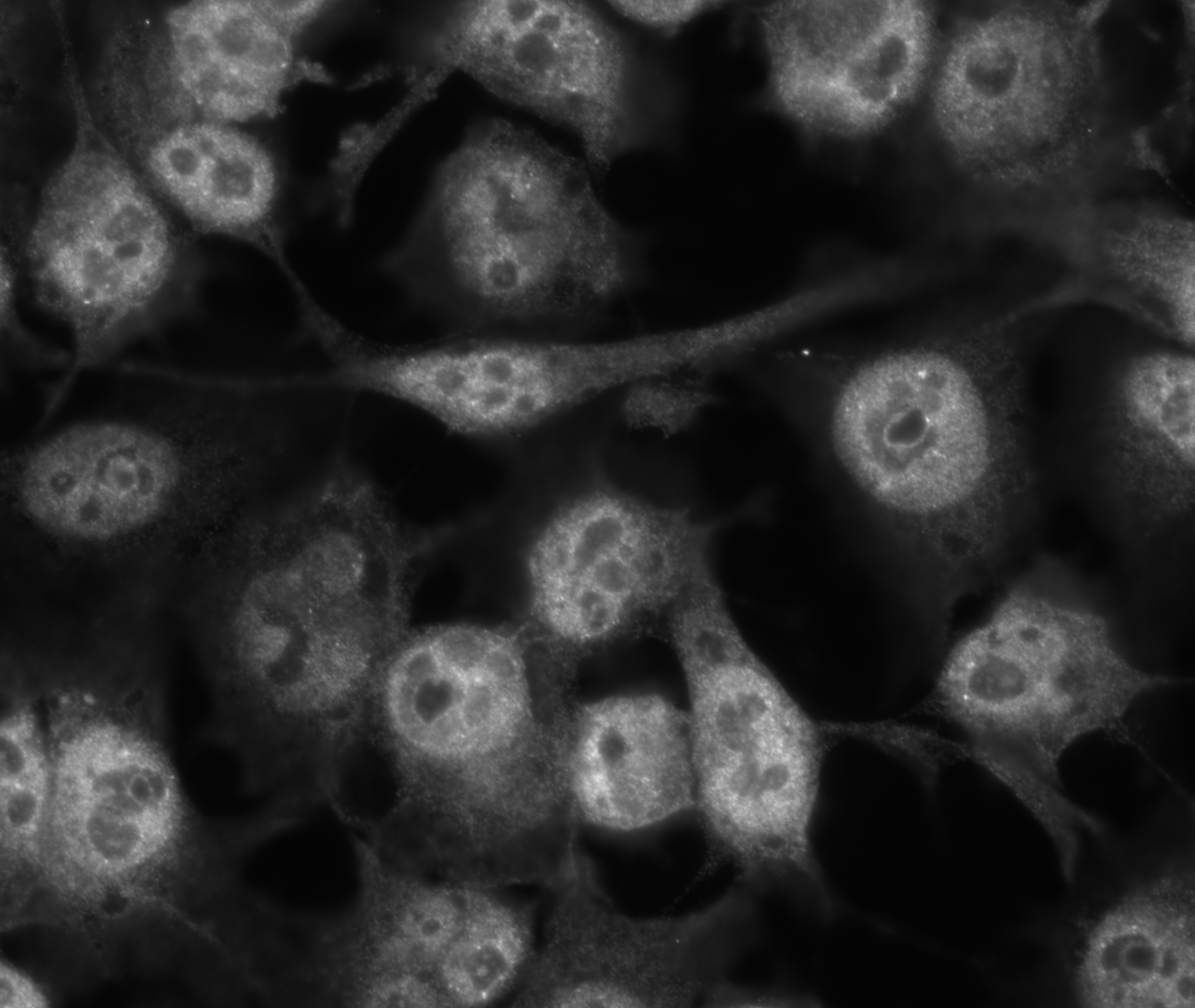

Supplement: Supplementary file 11 — Source data Fig. 3 [file 44318_2024_333_MOESM11_ESM.zip › Figure 3/Figure 3B/MG132/H12 No reccovery 2.7_w1TexasRed.TIF]

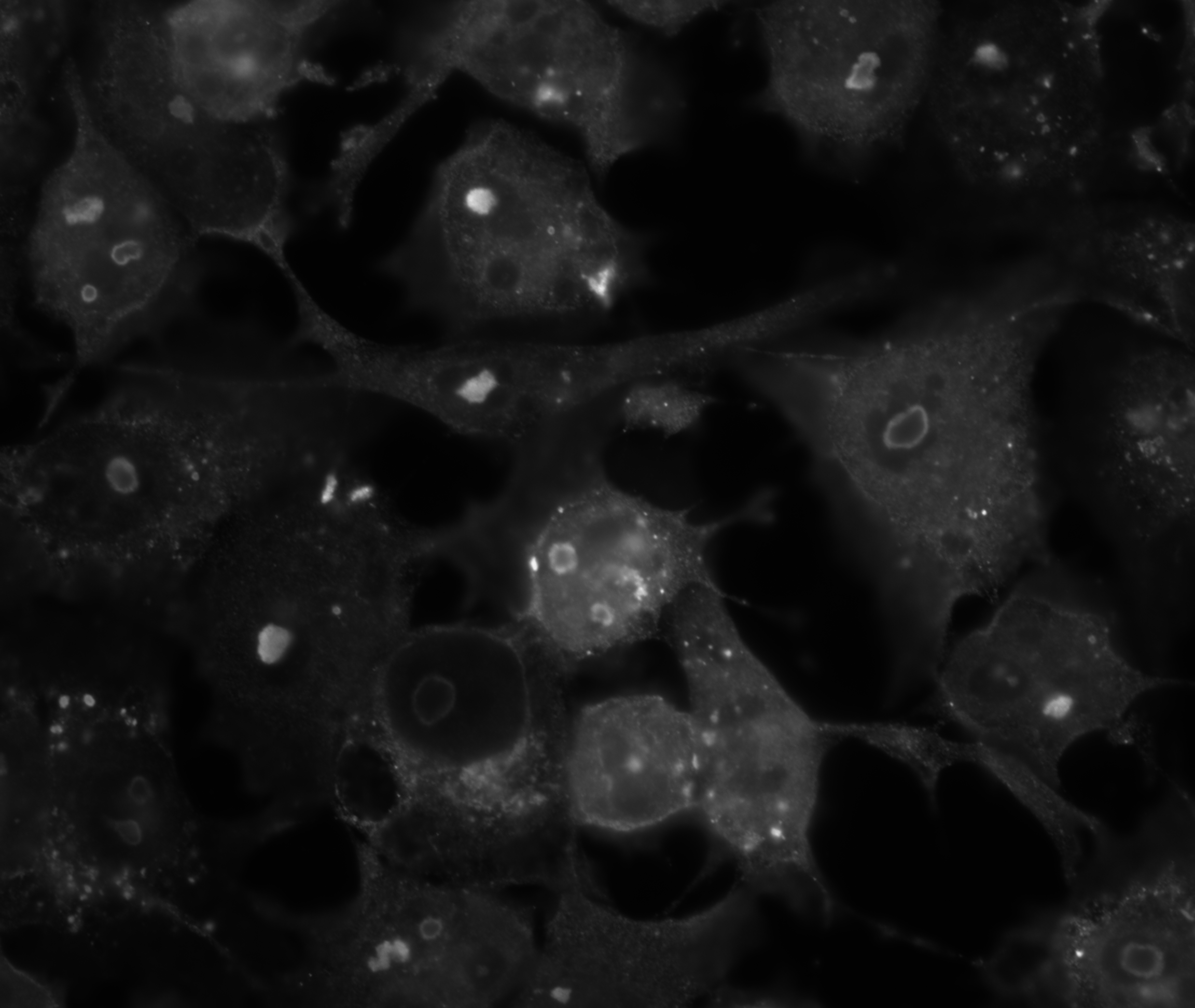

Supplement: Supplementary file 11 — Source data Fig. 3 [file 44318_2024_333_MOESM11_ESM.zip › Figure 3/Figure 3B/MG132/H12 No reccovery 2.7_w2GFP.TIF]

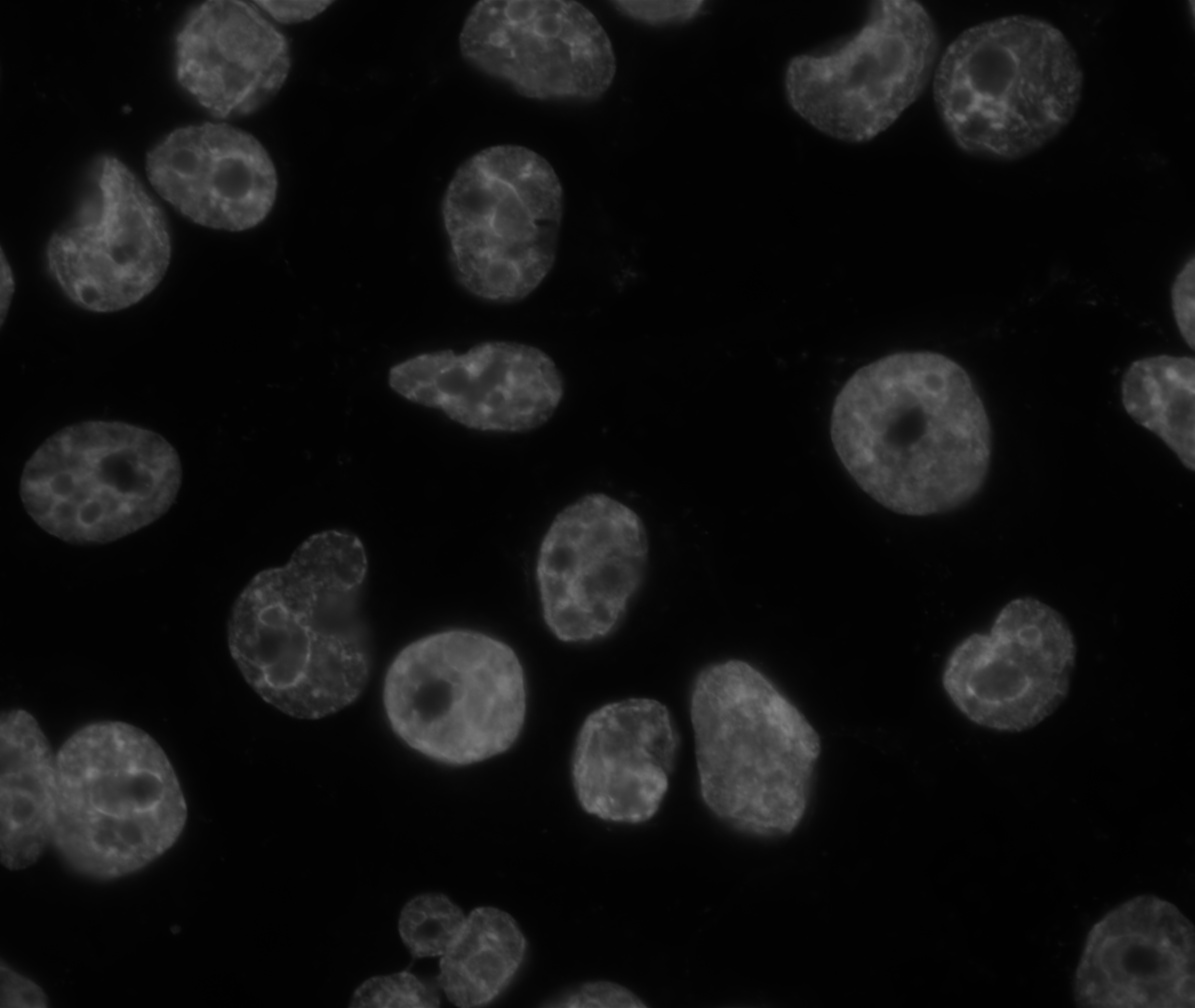

Supplement: Supplementary file 11 — Source data Fig. 3 [file 44318_2024_333_MOESM11_ESM.zip › Figure 3/Figure 3B/MG132/H12 No reccovery 2.7_w3DAPI.TIF]

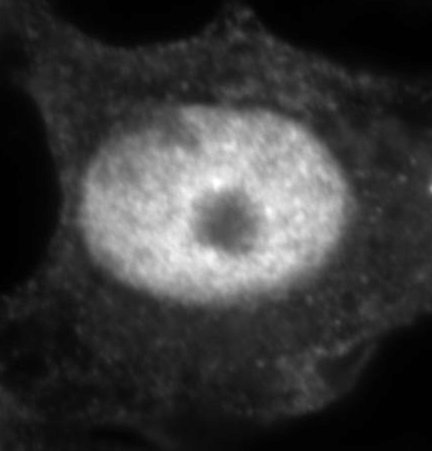

Supplement: Supplementary file 11 — Source data Fig. 3 [file 44318_2024_333_MOESM11_ESM.zip › Figure 3/Figure 3B/Recovery/H12 recovery 24h 2.0_w1TexasRed.jpg]

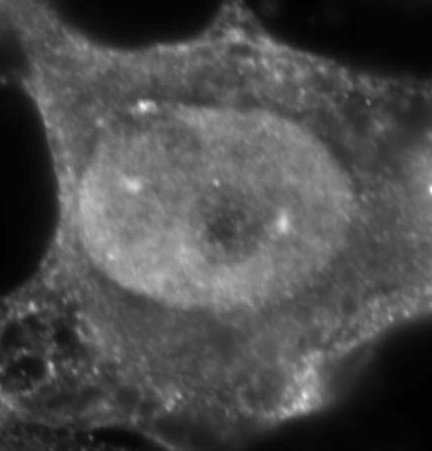

Supplement: Supplementary file 11 — Source data Fig. 3 [file 44318_2024_333_MOESM11_ESM.zip › Figure 3/Figure 3B/Recovery/H12 recovery 24h 2.0_w2GFP.jpg]

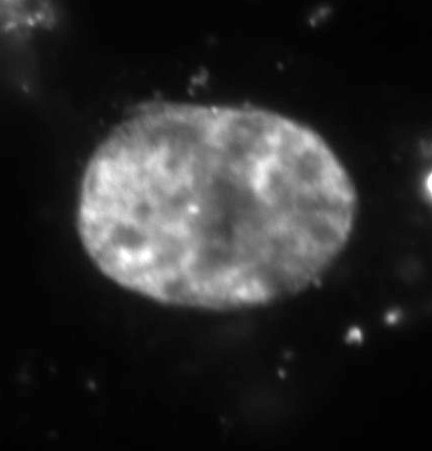

Supplement: Supplementary file 11 — Source data Fig. 3 [file 44318_2024_333_MOESM11_ESM.zip › Figure 3/Figure 3B/Recovery/H12 recovery 24h 2.0_w3DAPI.jpg]

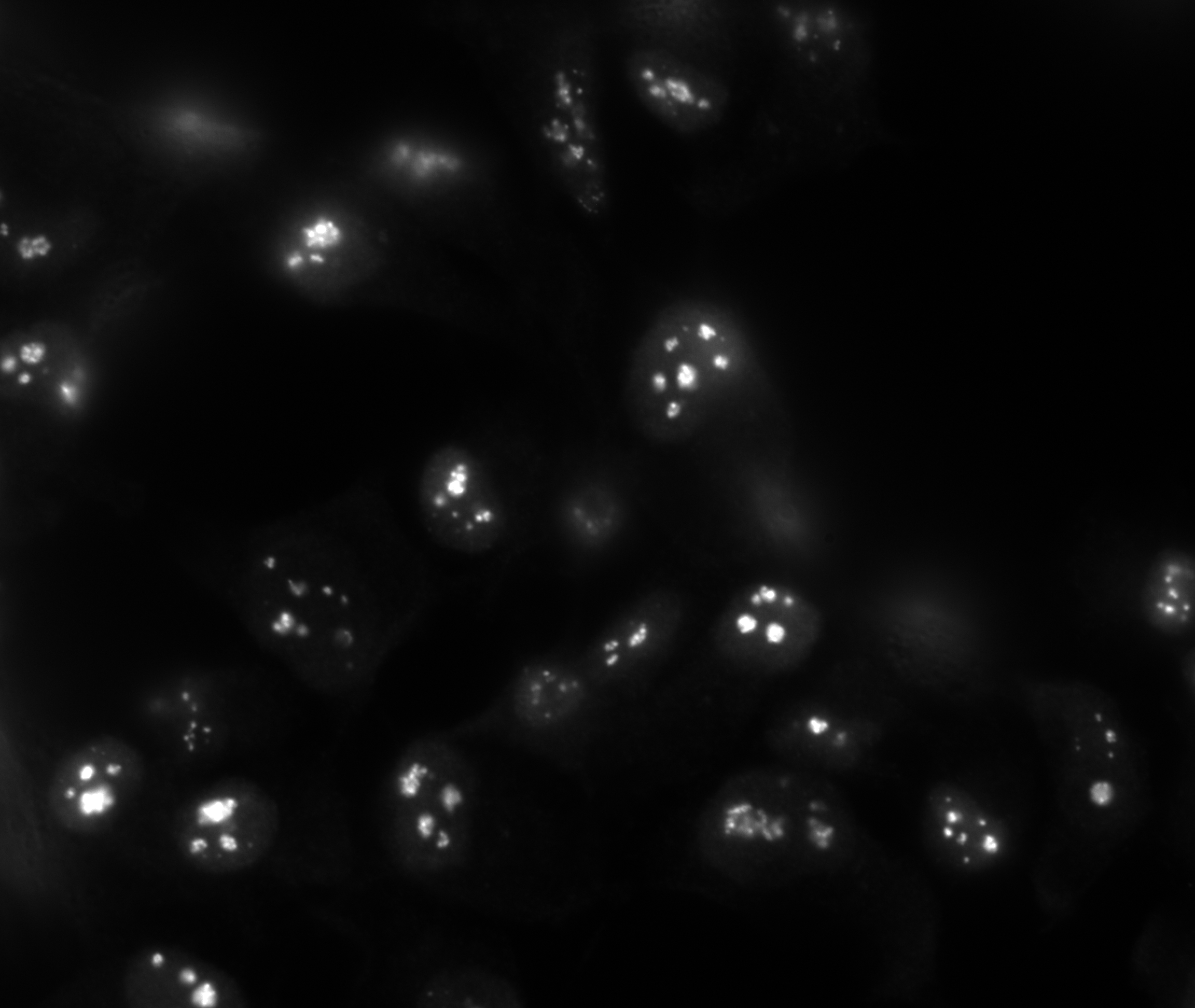

Supplement: Supplementary file 11 — Source data Fig. 3 [file 44318_2024_333_MOESM11_ESM.zip › Figure 3/Figure 3D/CTR/H12 GFP RPL11 no stress 0.19_w1Cy5.TIF]

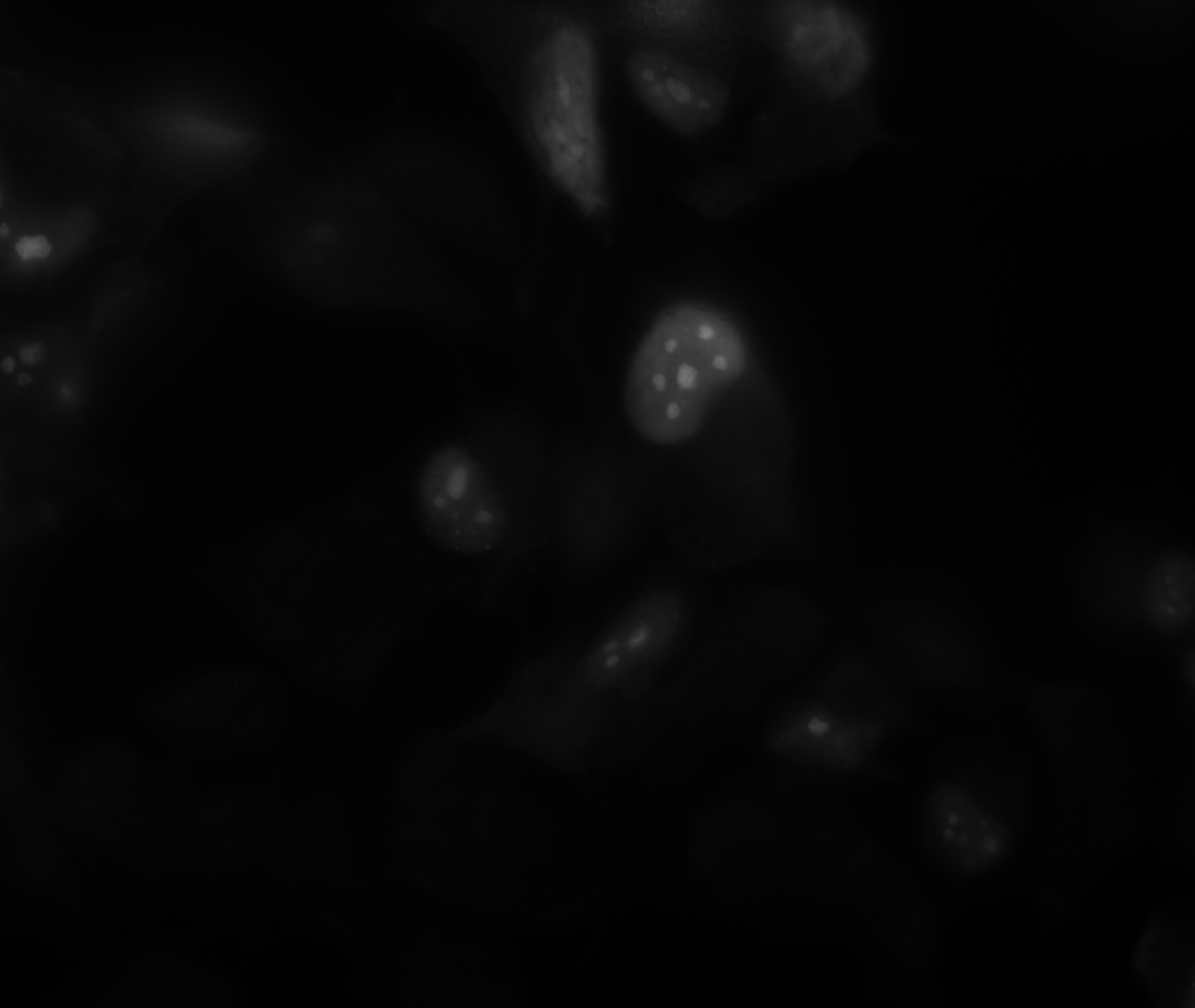

Supplement: Supplementary file 11 — Source data Fig. 3 [file 44318_2024_333_MOESM11_ESM.zip › Figure 3/Figure 3D/CTR/H12 GFP RPL11 no stress 0.19_w2GFP.TIF]

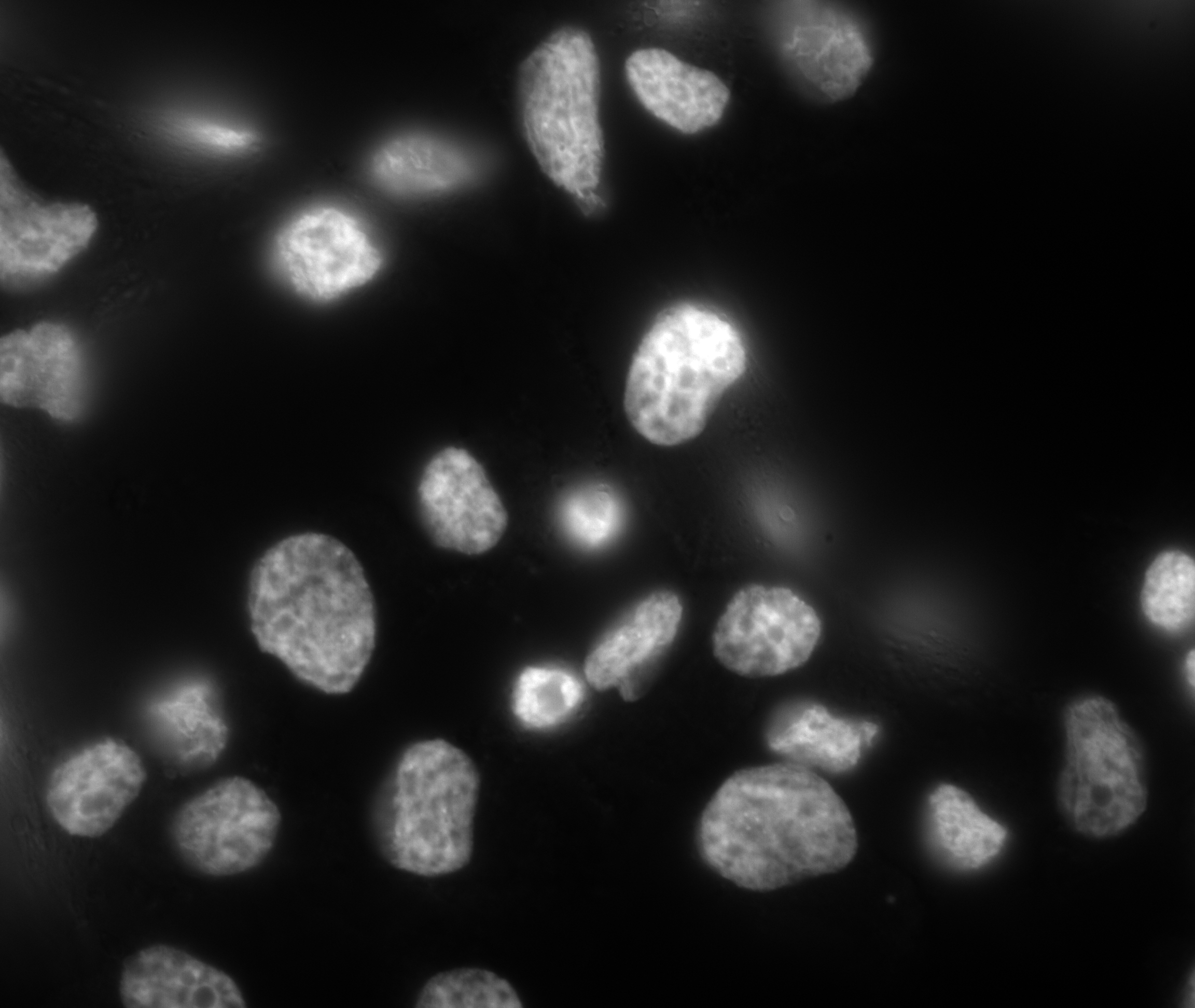

Supplement: Supplementary file 11 — Source data Fig. 3 [file 44318_2024_333_MOESM11_ESM.zip › Figure 3/Figure 3D/CTR/H12 GFP RPL11 no stress 0.19_w3DAPI.TIF]

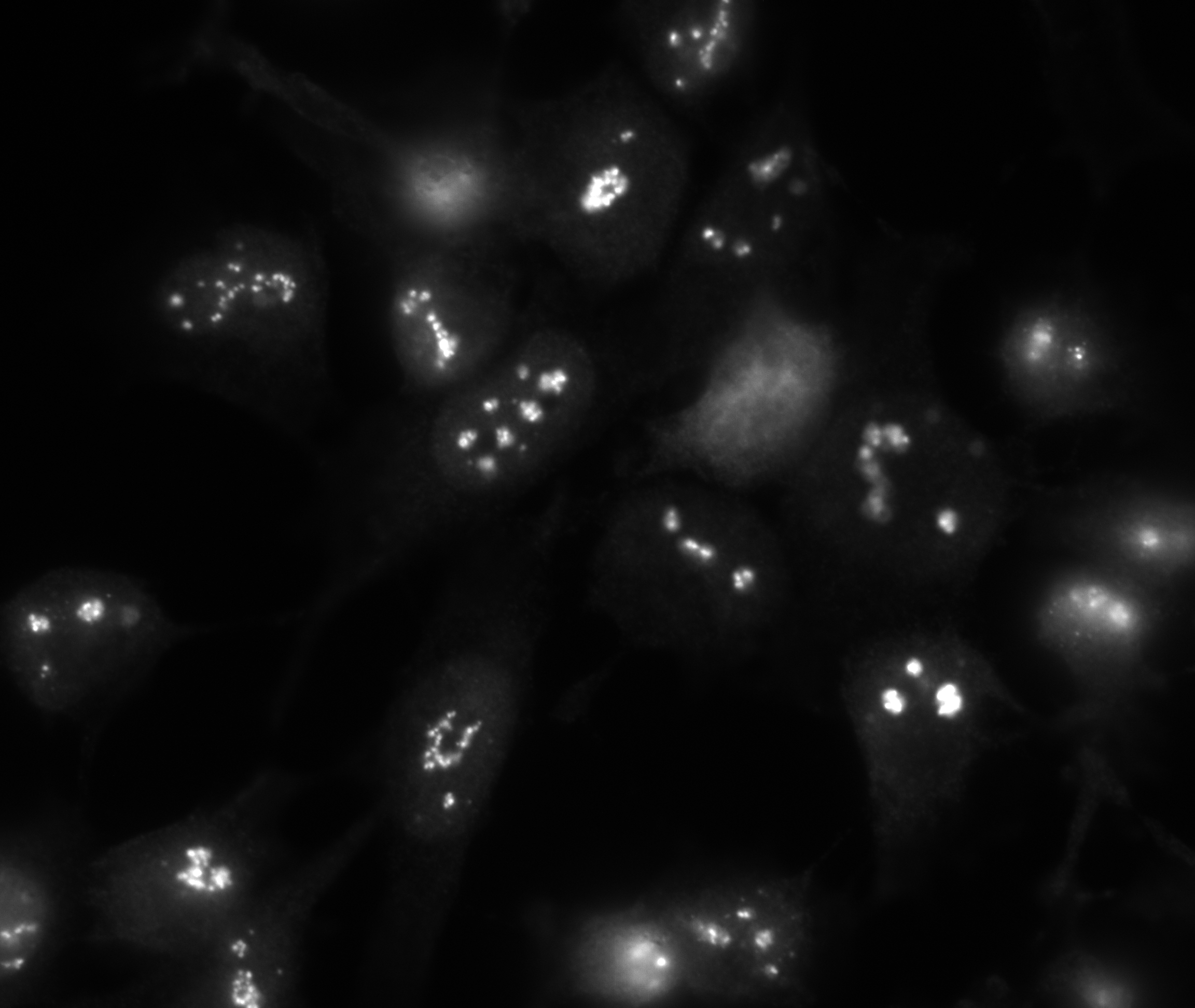

Supplement: Supplementary file 11 — Source data Fig. 3 [file 44318_2024_333_MOESM11_ESM.zip › Figure 3/Figure 3D/MG132/H12 GFP RPL11 no recovery 0.5_w1Cy5.TIF]

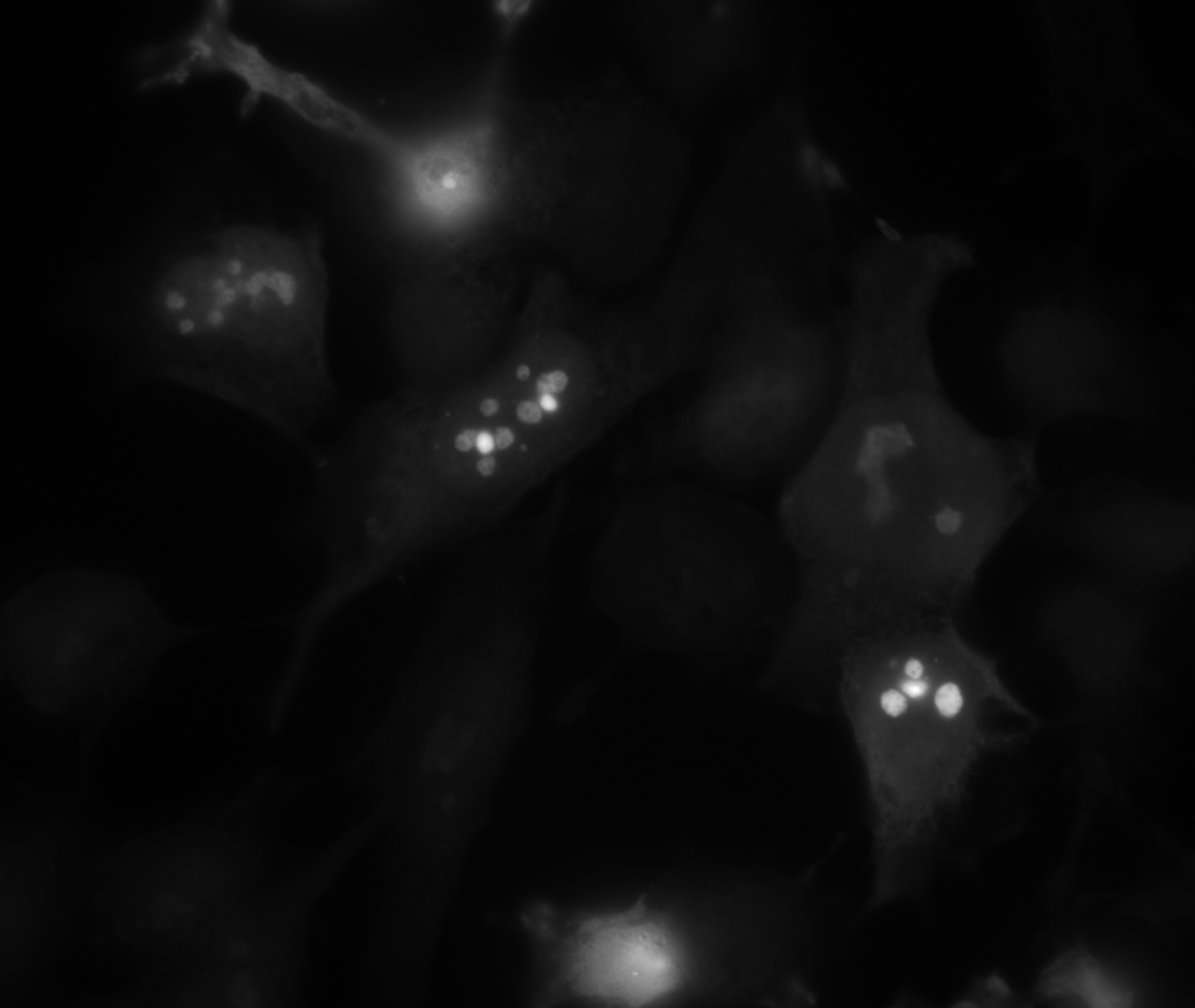

Supplement: Supplementary file 11 — Source data Fig. 3 [file 44318_2024_333_MOESM11_ESM.zip › Figure 3/Figure 3D/MG132/H12 GFP RPL11 no recovery 0.5_w2GFP.TIF]

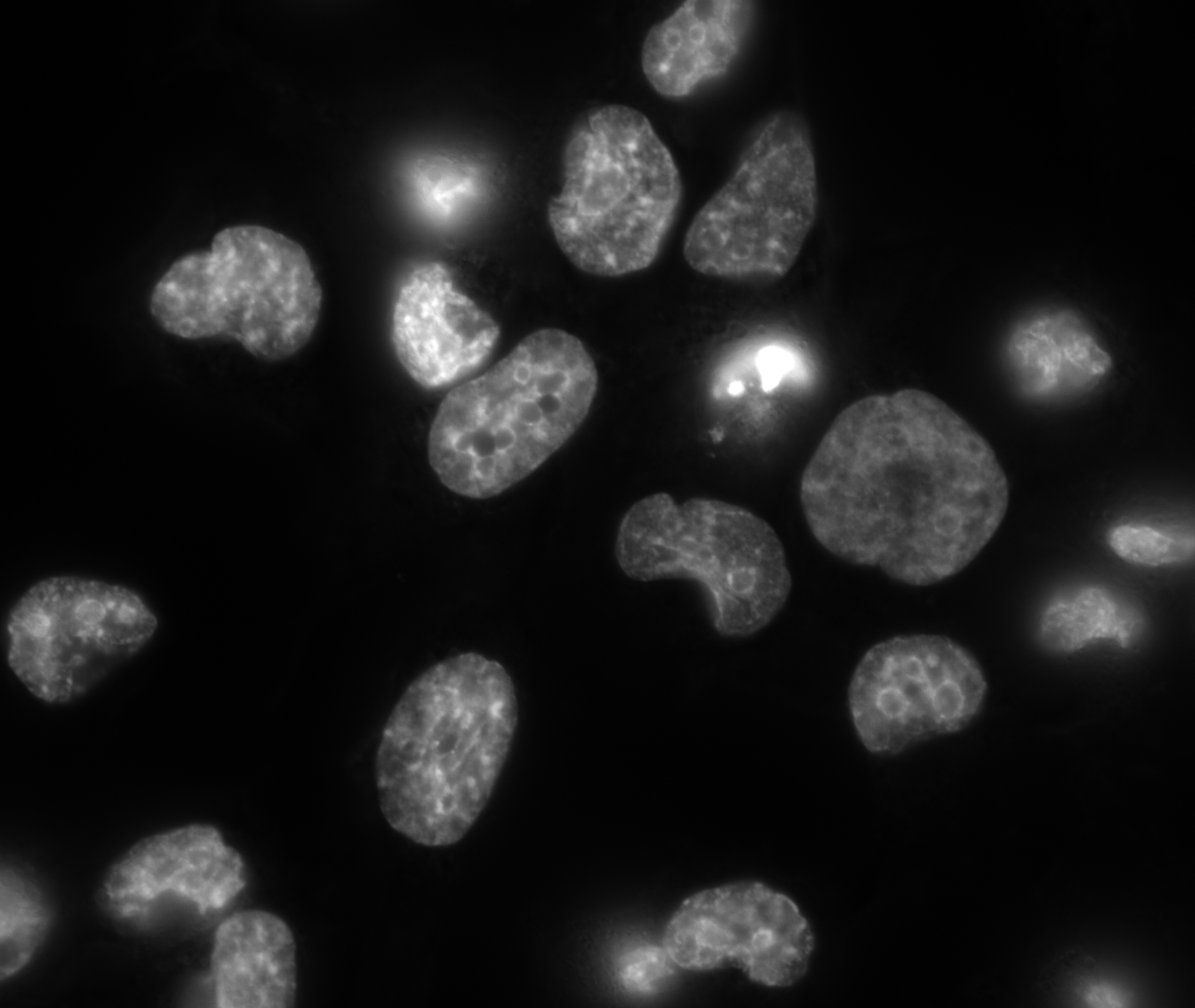

Supplement: Supplementary file 11 — Source data Fig. 3 [file 44318_2024_333_MOESM11_ESM.zip › Figure 3/Figure 3D/MG132/H12 GFP RPL11 no recovery 0.5_w3DAPI.TIF]

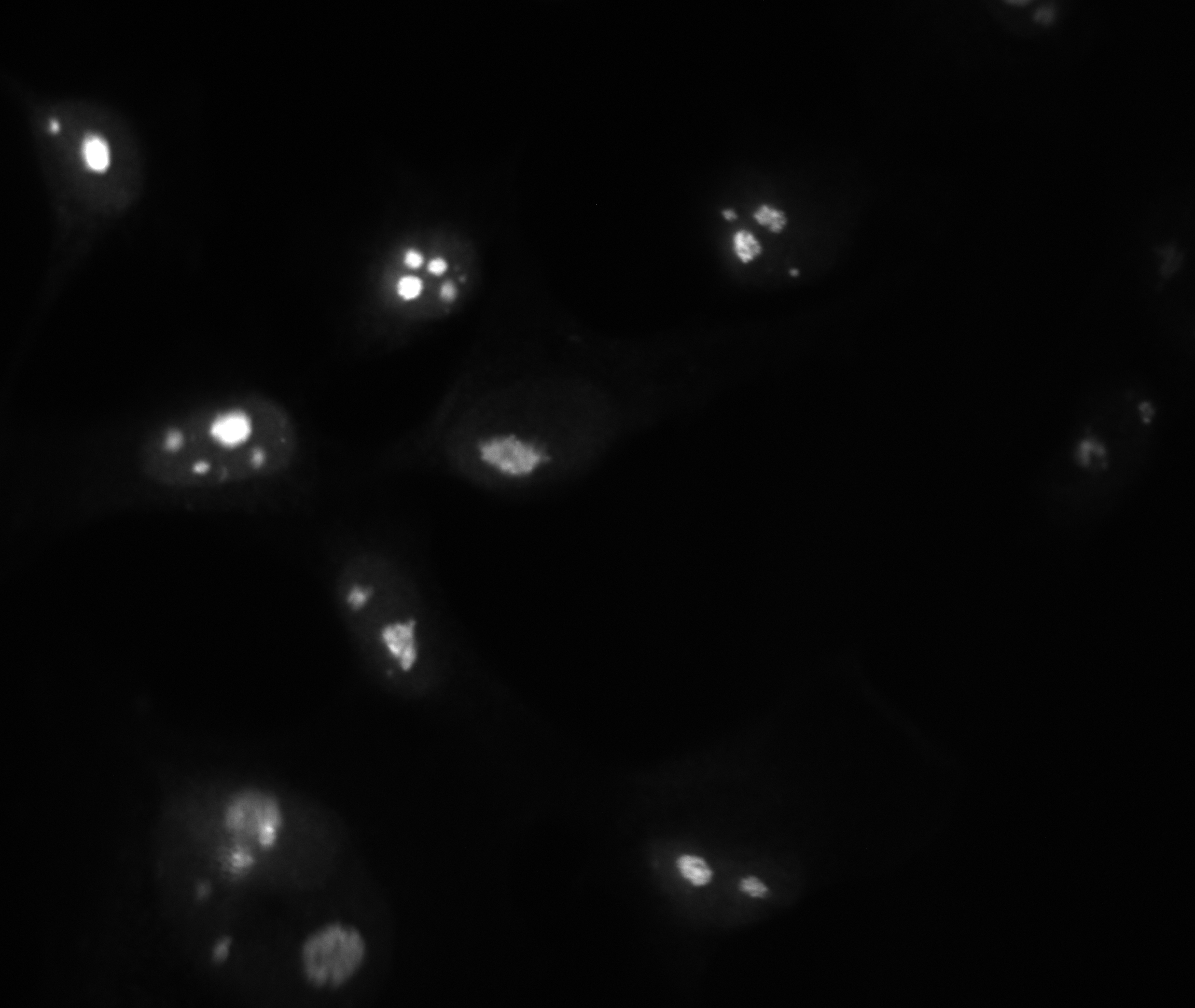

Supplement: Supplementary file 11 — Source data Fig. 3 [file 44318_2024_333_MOESM11_ESM.zip › Figure 3/Figure 3D/Recovery/H12 GFP RPL11 recovery 24h 0.8_w1Cy5.TIF]

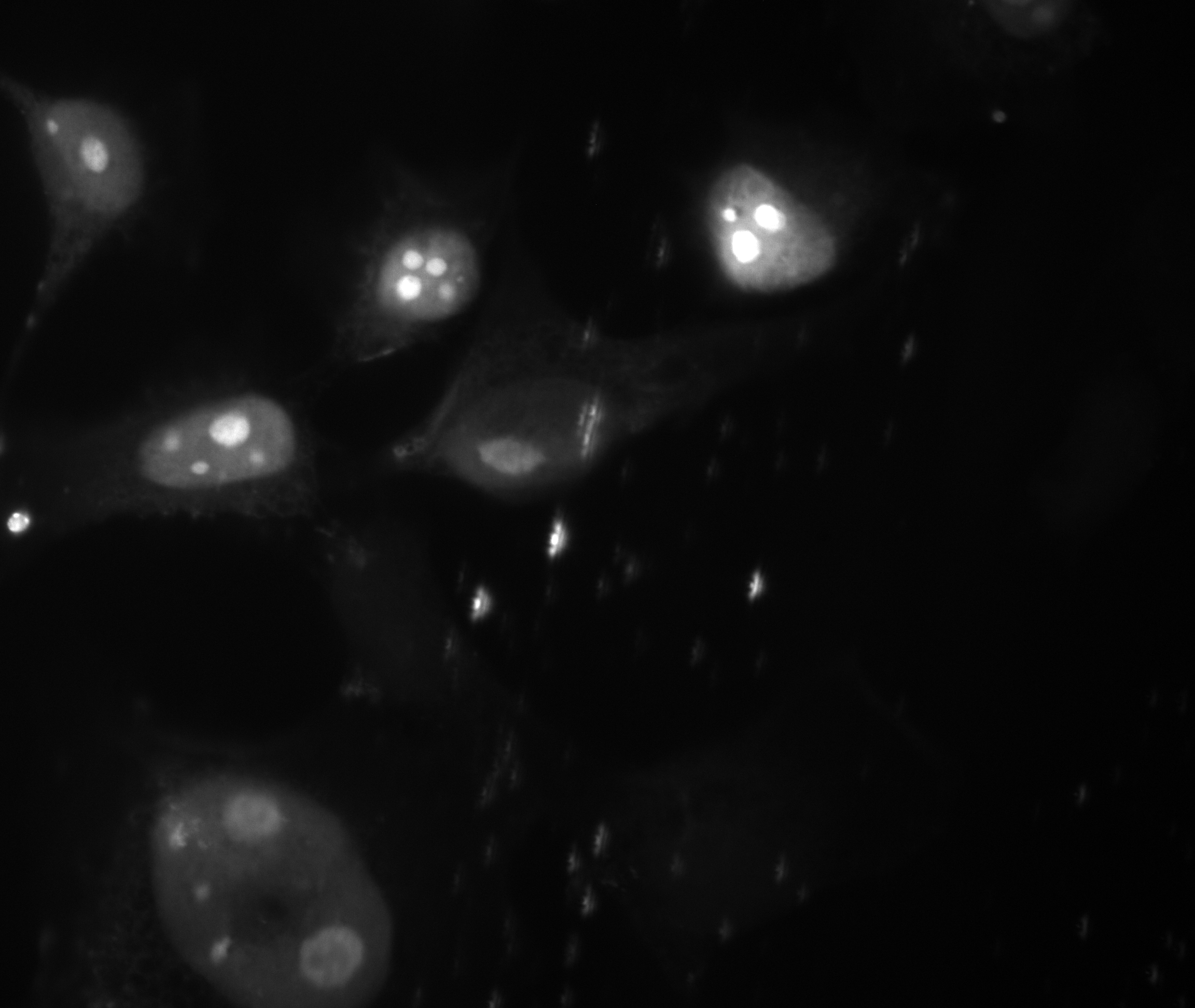

Supplement: Supplementary file 11 — Source data Fig. 3 [file 44318_2024_333_MOESM11_ESM.zip › Figure 3/Figure 3D/Recovery/H12 GFP RPL11 recovery 24h 0.8_w2GFP.TIF]

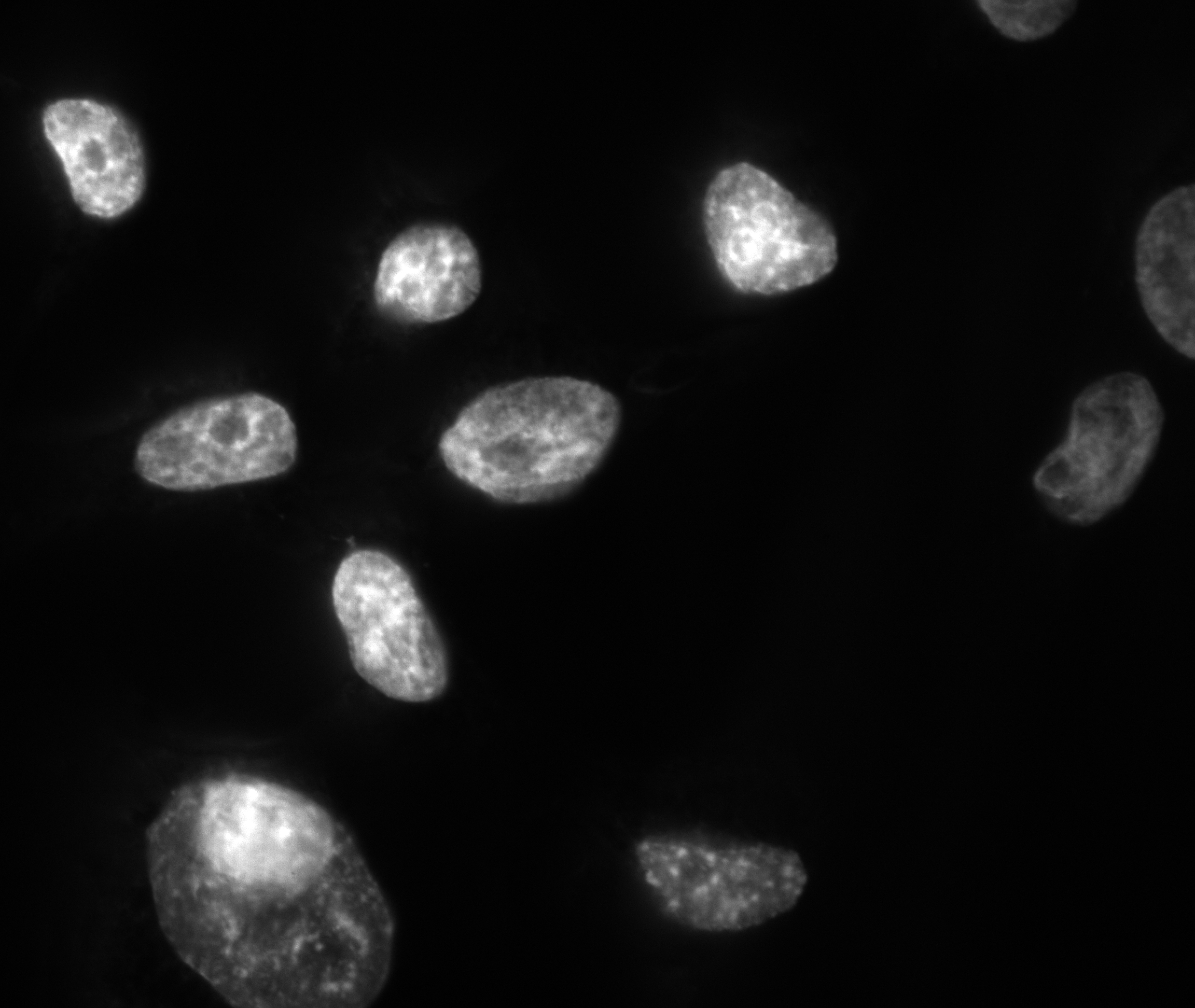

Supplement: Supplementary file 11 — Source data Fig. 3 [file 44318_2024_333_MOESM11_ESM.zip › Figure 3/Figure 3D/Recovery/H12 GFP RPL11 recovery 24h 0.8_w3DAPI (1).TIF]

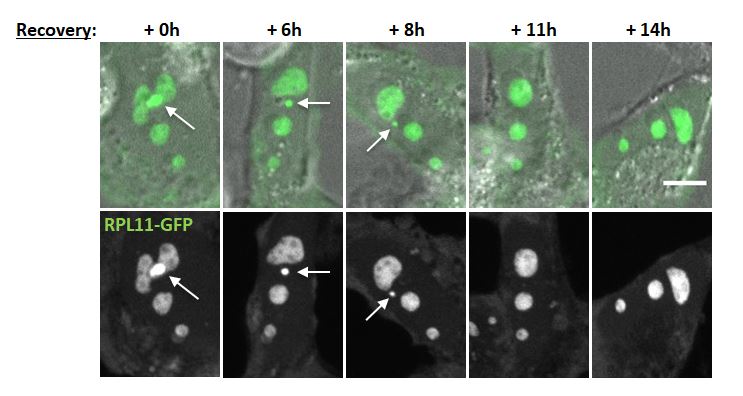

Supplement: Supplementary file 11 — Source data Fig. 3 [file 44318_2024_333_MOESM11_ESM.zip › Figure 3/Figure 3F /Live Imaging_GFP DIC_Recovery.JPG]

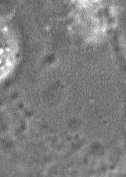

Supplement: Supplementary file 11 — Source data Fig. 3 [file 44318_2024_333_MOESM11_ESM.zip › Figure 3/Figure 3F /Position 1/Cell-04_arrows.jpg]

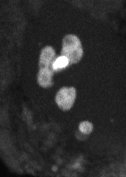

Supplement: Supplementary file 11 — Source data Fig. 3 [file 44318_2024_333_MOESM11_ESM.zip › Figure 3/Figure 3F /Position 1/Cell-04_arrows___.jpg]

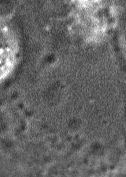

Supplement: Supplementary file 11 — Source data Fig. 3 [file 44318_2024_333_MOESM11_ESM.zip › Figure 3/Figure 3F /Position 1/Cell-04_arrows_contrast.jpg]

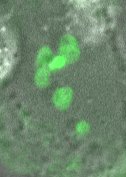

Supplement: Supplementary file 11 — Source data Fig. 3 [file 44318_2024_333_MOESM11_ESM.zip › Figure 3/Figure 3F /Position 1/Cell-04_arrowsdf.jpg]

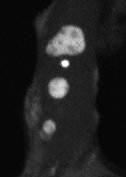

Supplement: Supplementary file 11 — Source data Fig. 3 [file 44318_2024_333_MOESM11_ESM.zip › Figure 3/Figure 3F /Position 2/Cell-04_arrows.jpg]

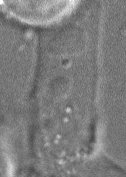

Supplement: Supplementary file 11 — Source data Fig. 3 [file 44318_2024_333_MOESM11_ESM.zip › Figure 3/Figure 3F /Position 2/Cell-04_arrows_.jpg]

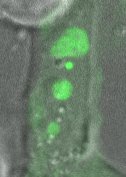

Supplement: Supplementary file 11 — Source data Fig. 3 [file 44318_2024_333_MOESM11_ESM.zip › Figure 3/Figure 3F /Position 2/Cell-04_arrows__.jpg]

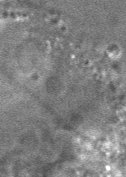

Supplement: Supplementary file 11 — Source data Fig. 3 [file 44318_2024_333_MOESM11_ESM.zip › Figure 3/Figure 3F /Position 3/Cell-04_arrows17.jpg]

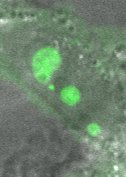

Supplement: Supplementary file 11 — Source data Fig. 3 [file 44318_2024_333_MOESM11_ESM.zip › Figure 3/Figure 3F /Position 3/Cell-04_arrows_17-2.jpg]

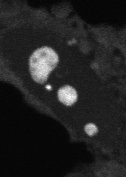

Supplement: Supplementary file 11 — Source data Fig. 3 [file 44318_2024_333_MOESM11_ESM.zip › Figure 3/Figure 3F /Position 3/Cell-04_arrows_17-3.jpg]

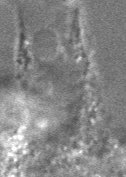

Supplement: Supplementary file 11 — Source data Fig. 3 [file 44318_2024_333_MOESM11_ESM.zip › Figure 3/Figure 3F /Position 4/Cell-04_arrows-22.jpg]

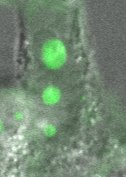

Supplement: Supplementary file 11 — Source data Fig. 3 [file 44318_2024_333_MOESM11_ESM.zip › Figure 3/Figure 3F /Position 4/Cell-04_arrows_222.jpg]

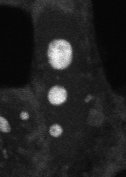

Supplement: Supplementary file 11 — Source data Fig. 3 [file 44318_2024_333_MOESM11_ESM.zip › Figure 3/Figure 3F /Position 4/Cell-04_arrows_position 22.jpg]

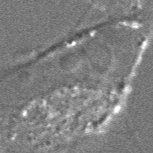

Supplement: Supplementary file 11 — Source data Fig. 3 [file 44318_2024_333_MOESM11_ESM.zip › Figure 3/Figure 3F /Position 5/Cell-04_arrows.jpg]

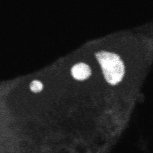

Supplement: Supplementary file 11 — Source data Fig. 3 [file 44318_2024_333_MOESM11_ESM.zip › Figure 3/Figure 3F /Position 5/Cell-04_arrows_position 28-2.jpg]

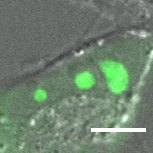

Supplement: Supplementary file 11 — Source data Fig. 3 [file 44318_2024_333_MOESM11_ESM.zip › Figure 3/Figure 3F /Position 5/Cell-04_arrows_position 28.jpg]

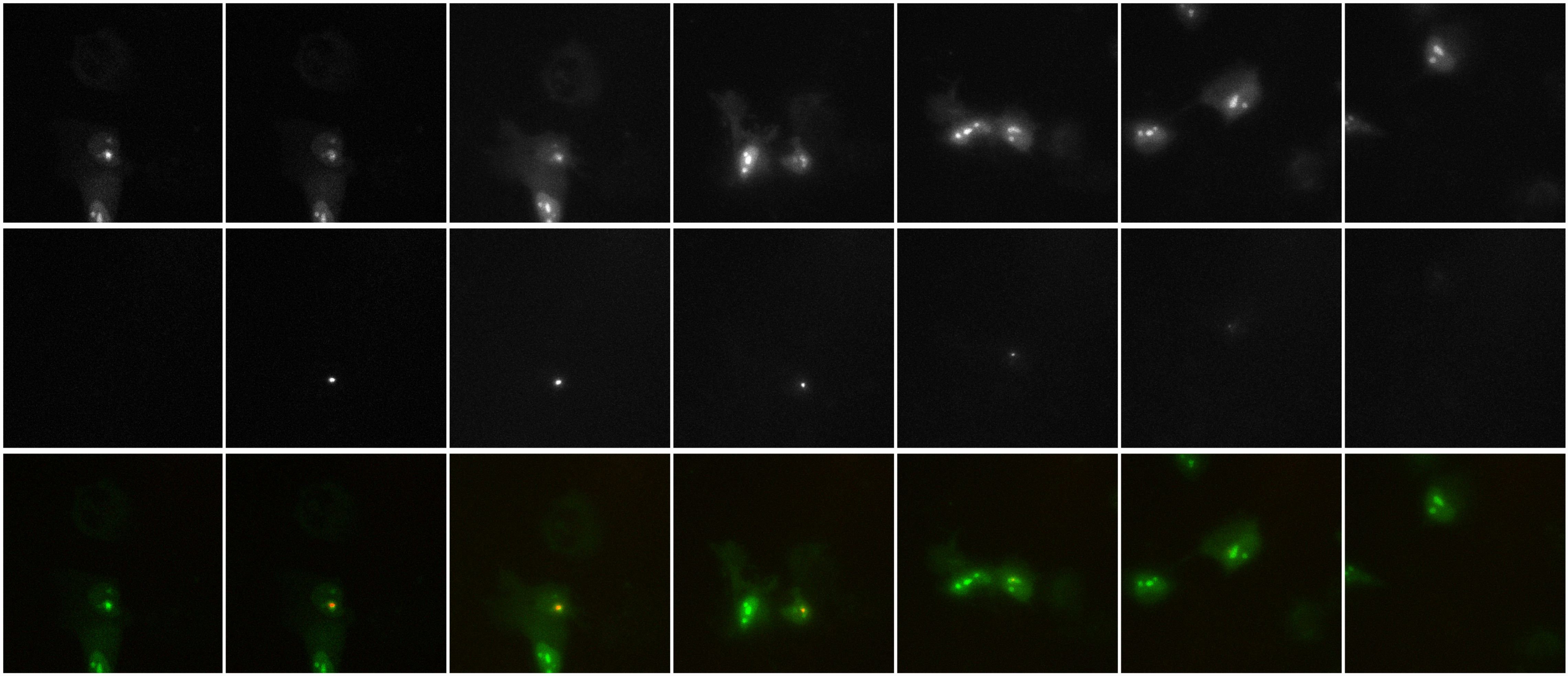

Supplement: Supplementary file 11 — Source data Fig. 3 [file 44318_2024_333_MOESM11_ESM.zip › Figure 3/Figure 3G /Fig. 3G_Movie EV4.tif]

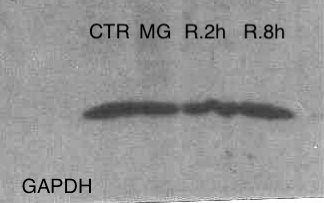

Supplement: Supplementary file 11 — Source data Fig. 3 [file 44318_2024_333_MOESM11_ESM.zip › Figure 3/Western blots_FIg 3/Fig. 3A_GAPDH.jpg]

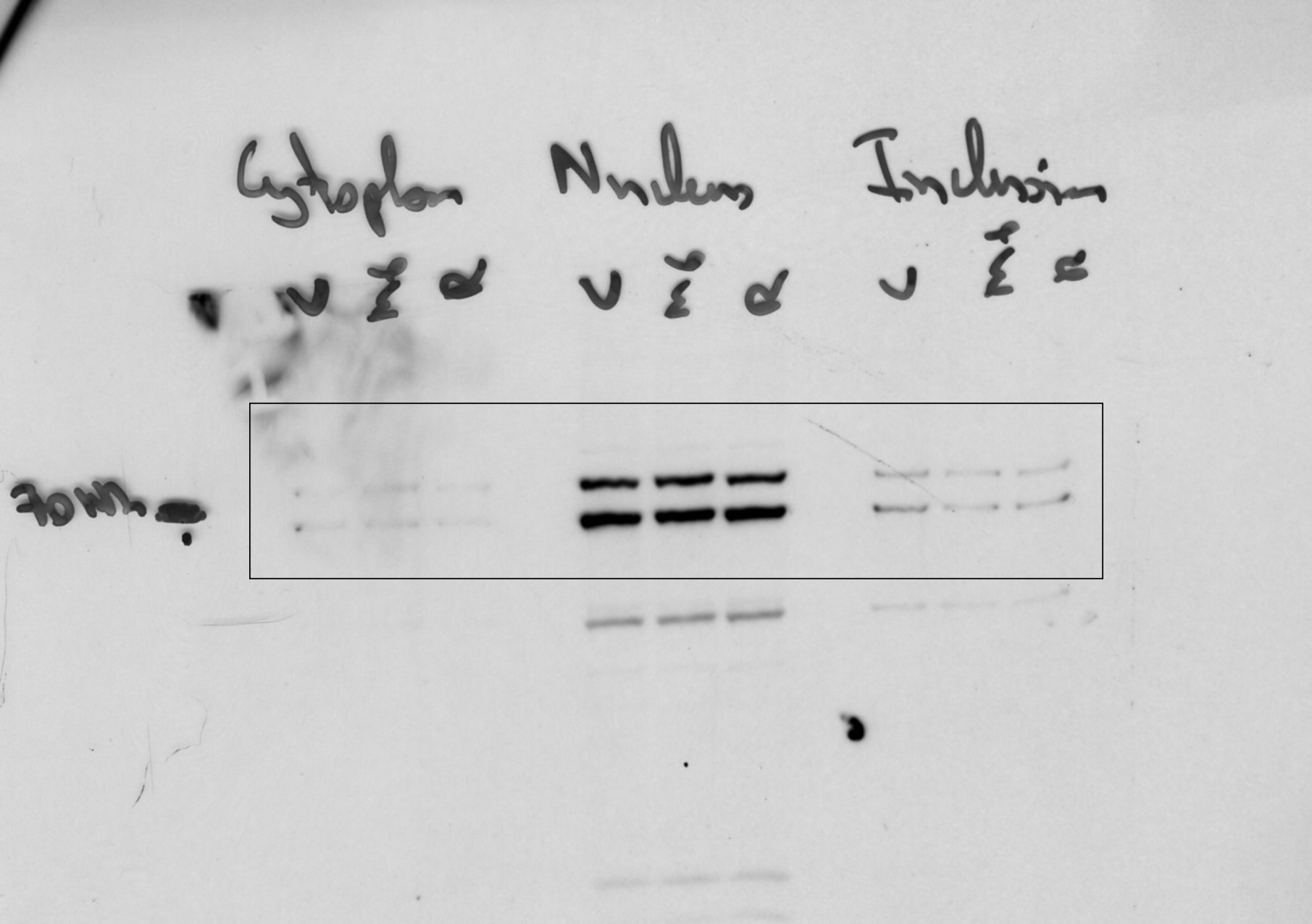

Supplement: Supplementary file 11 — Source data Fig. 3 [file 44318_2024_333_MOESM11_ESM.zip › Figure 3/Western blots_FIg 3/Lamin A_C_fraction.jpg]

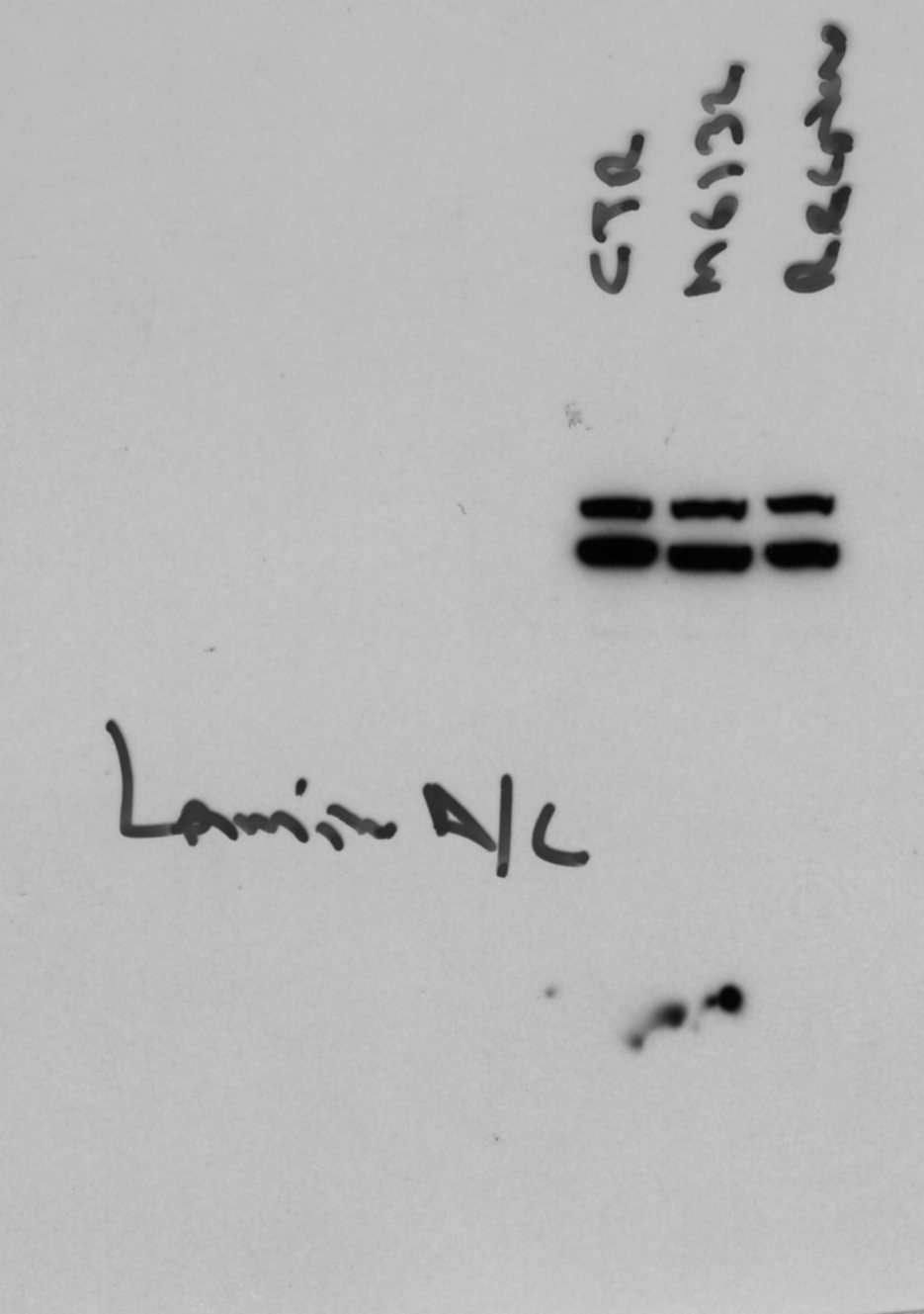

Supplement: Supplementary file 11 — Source data Fig. 3 [file 44318_2024_333_MOESM11_ESM.zip › Figure 3/Western blots_FIg 3/Lamin A_C_input.jpg]

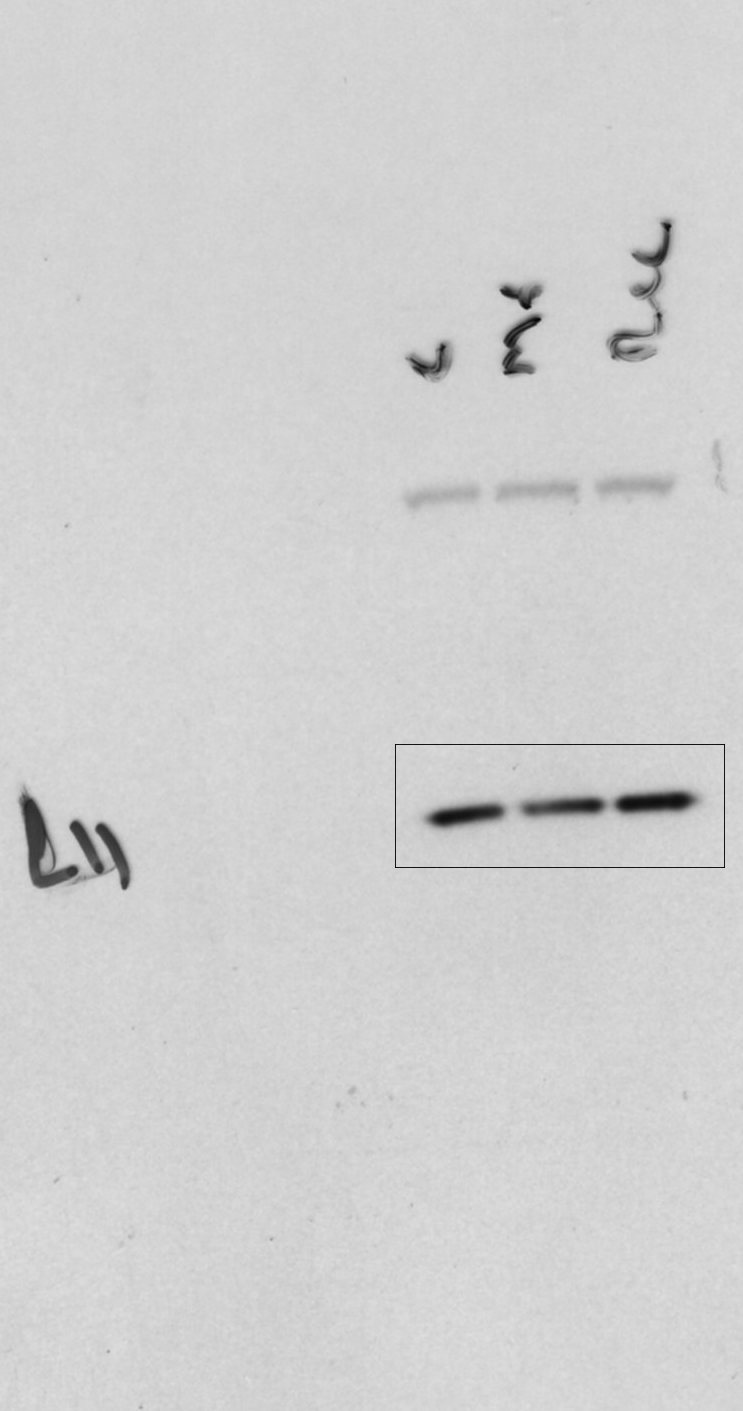

Supplement: Supplementary file 11 — Source data Fig. 3 [file 44318_2024_333_MOESM11_ESM.zip › Figure 3/Western blots_FIg 3/RPL11_cytoplasm.jpg]

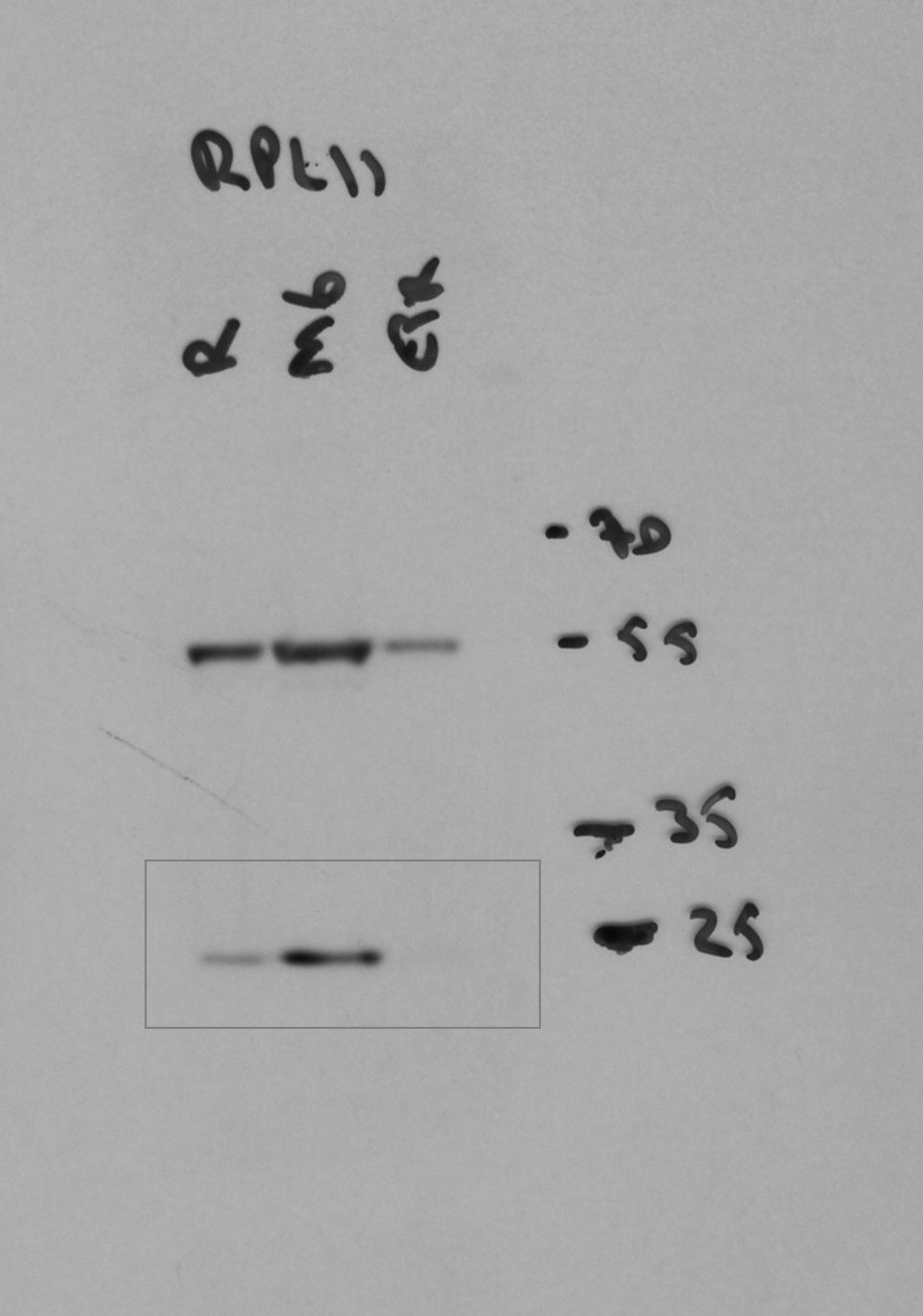

Supplement: Supplementary file 11 — Source data Fig. 3 [file 44318_2024_333_MOESM11_ESM.zip › Figure 3/Western blots_FIg 3/RPL11_inclusions.jpg]

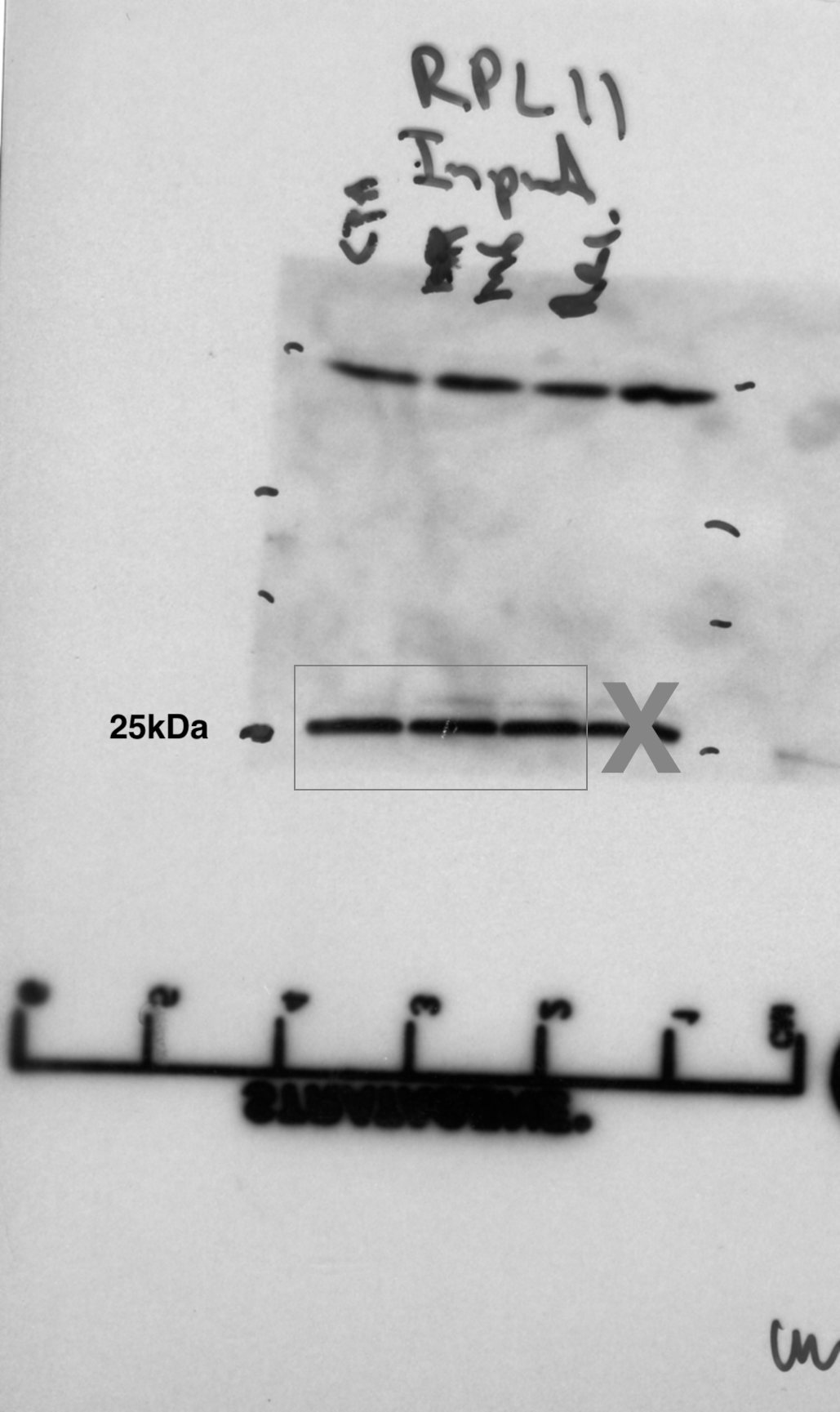

Supplement: Supplementary file 11 — Source data Fig. 3 [file 44318_2024_333_MOESM11_ESM.zip › Figure 3/Western blots_FIg 3/RPL11_input.jpg]

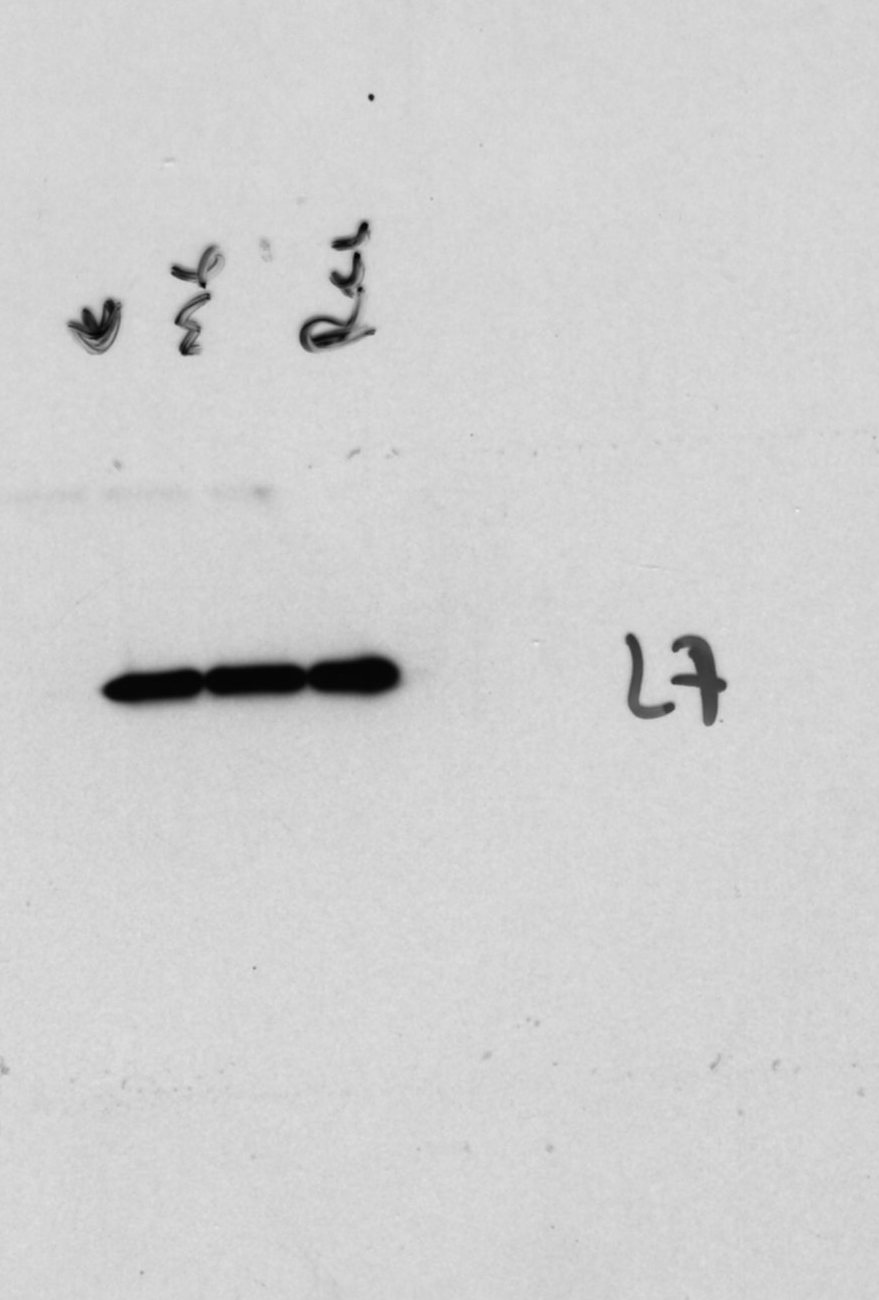

Supplement: Supplementary file 11 — Source data Fig. 3 [file 44318_2024_333_MOESM11_ESM.zip › Figure 3/Western blots_FIg 3/RPL7_cytoplasm.jpg]
