# Supplementary figures and images for: A nuclear protein quality control system for elimination of nucleolus-related inclusions (part 2 of 4)
Source: EMBO J. 2024 Dec 17;44(3):801–23. doi: 10.1038/s44318-024-00333-9 (PMC11791210; doi:10.1038/s44318-024-00333-9)

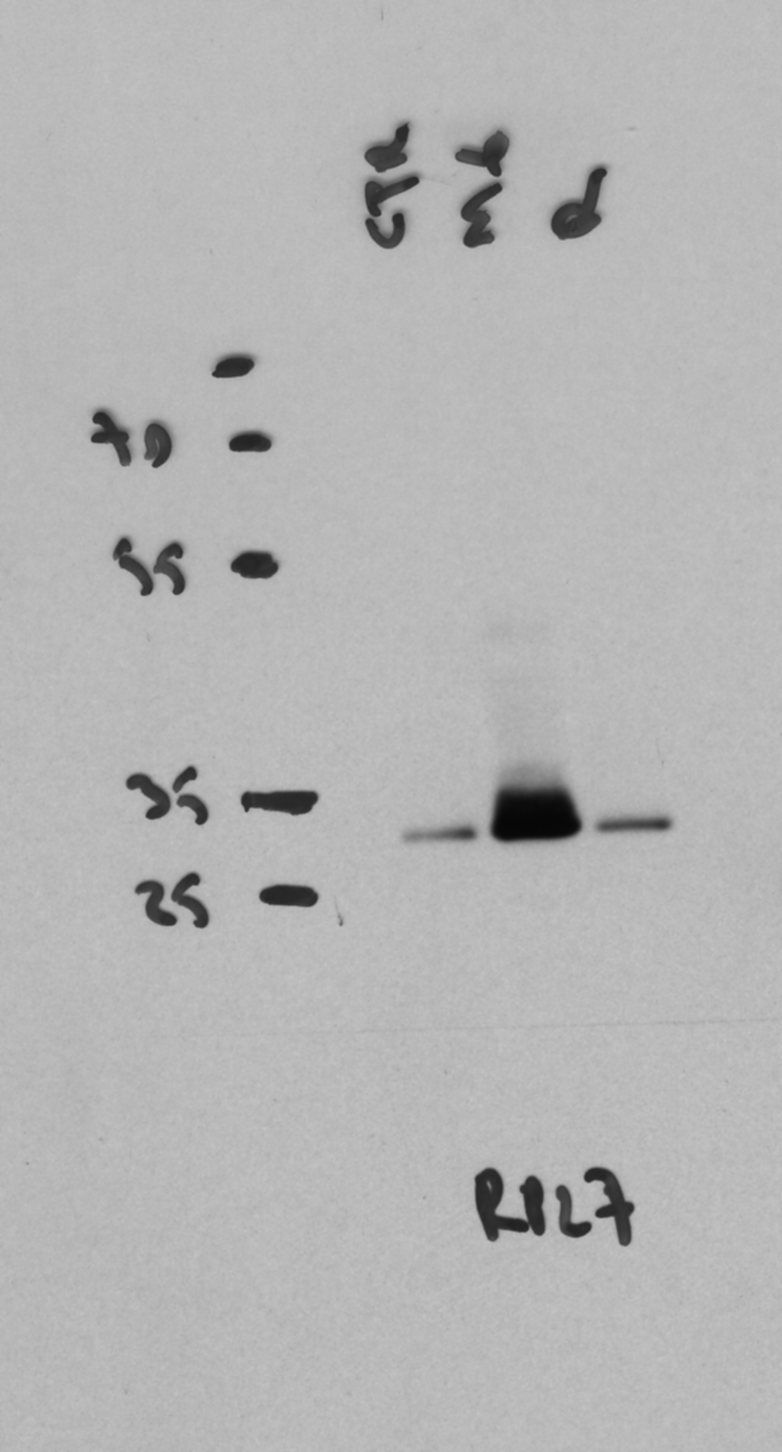

Supplement: Supplementary file 11 — Source data Fig. 3 [file 44318_2024_333_MOESM11_ESM.zip › Figure 3/Western blots_FIg 3/RPL7_inclusions.jpg]

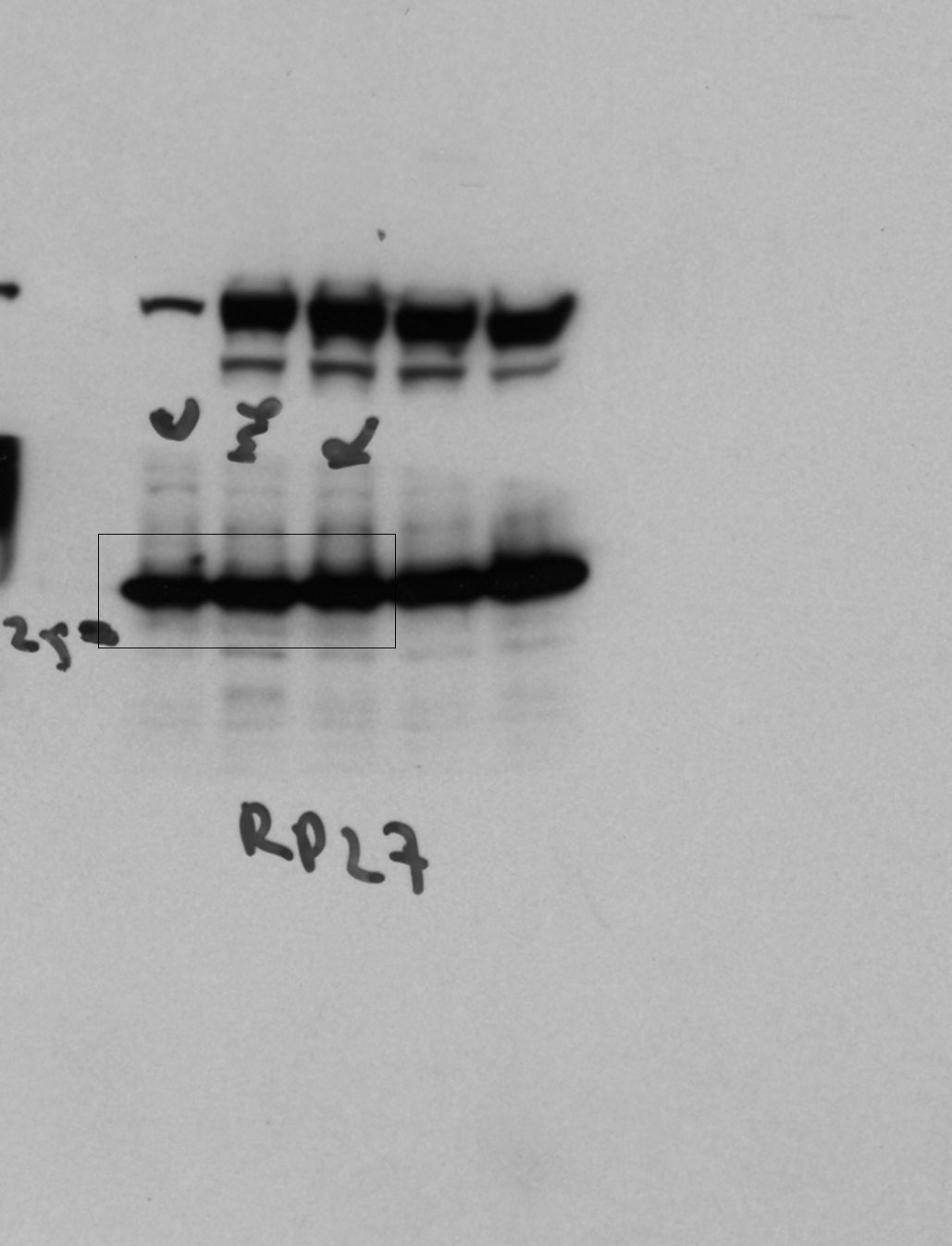

Supplement: Supplementary file 11 — Source data Fig. 3 [file 44318_2024_333_MOESM11_ESM.zip › Figure 3/Western blots_FIg 3/RPL7_input.jpg]

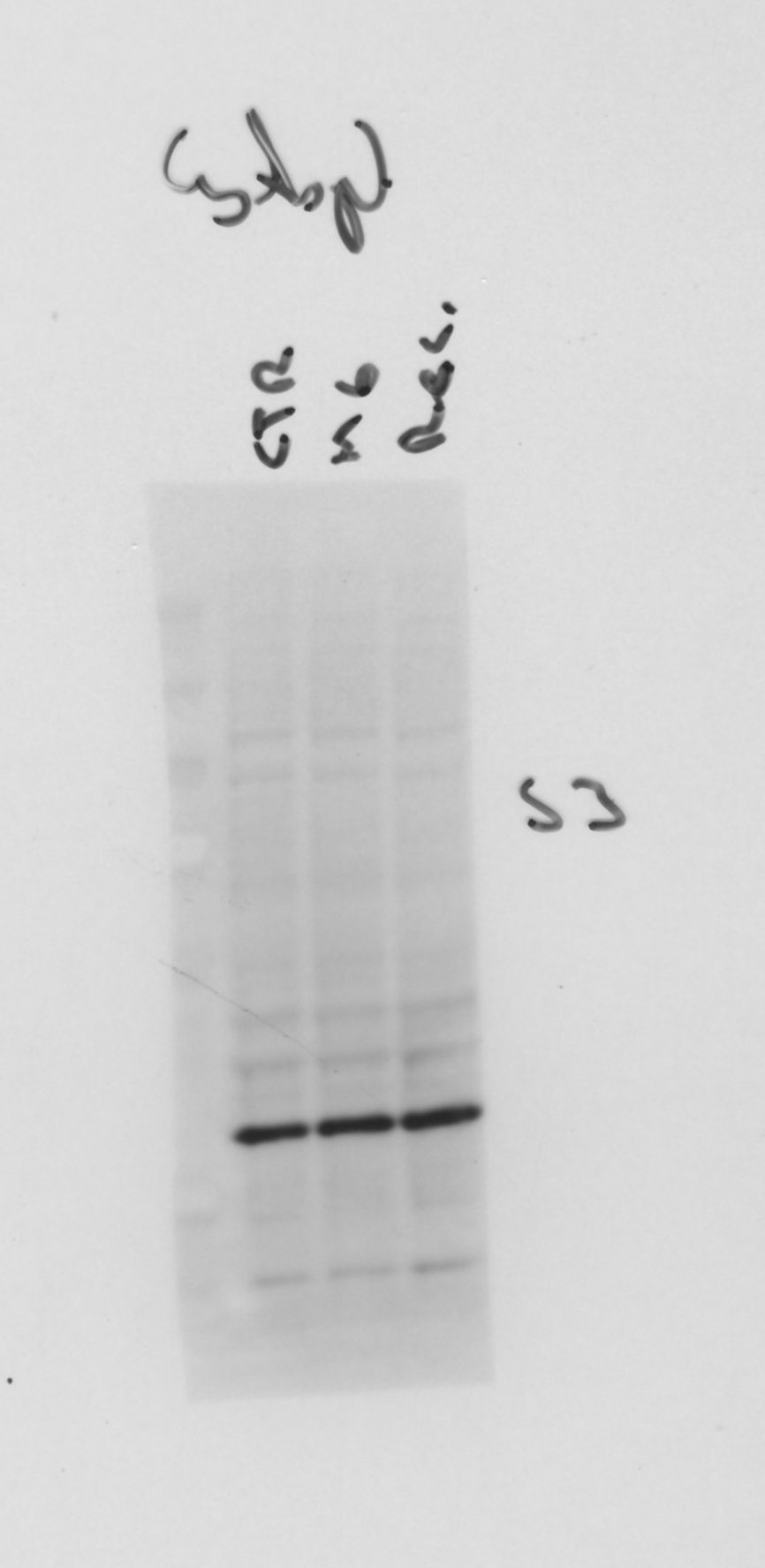

Supplement: Supplementary file 11 — Source data Fig. 3 [file 44318_2024_333_MOESM11_ESM.zip › Figure 3/Western blots_FIg 3/RPS3_cytoplasm.jpg]

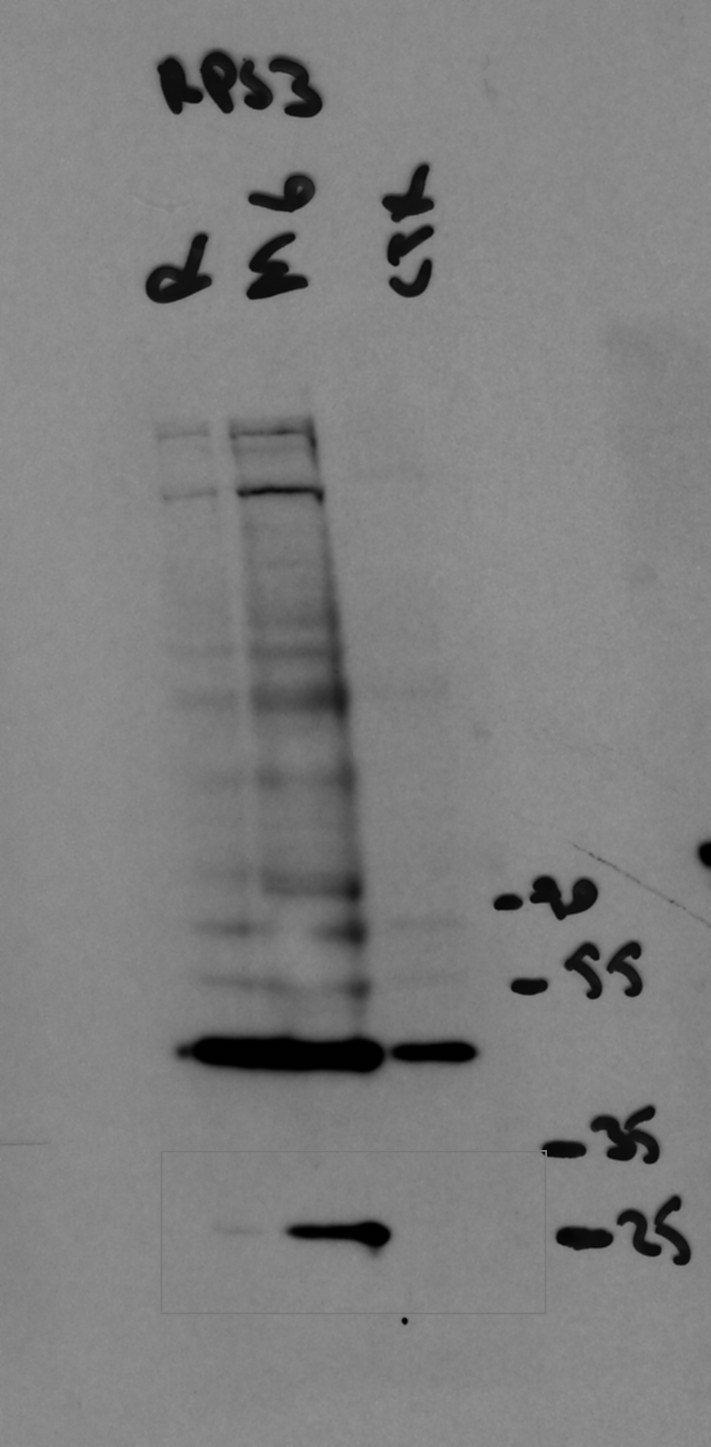

Supplement: Supplementary file 11 — Source data Fig. 3 [file 44318_2024_333_MOESM11_ESM.zip › Figure 3/Western blots_FIg 3/RPS3_inclusions.jpg]

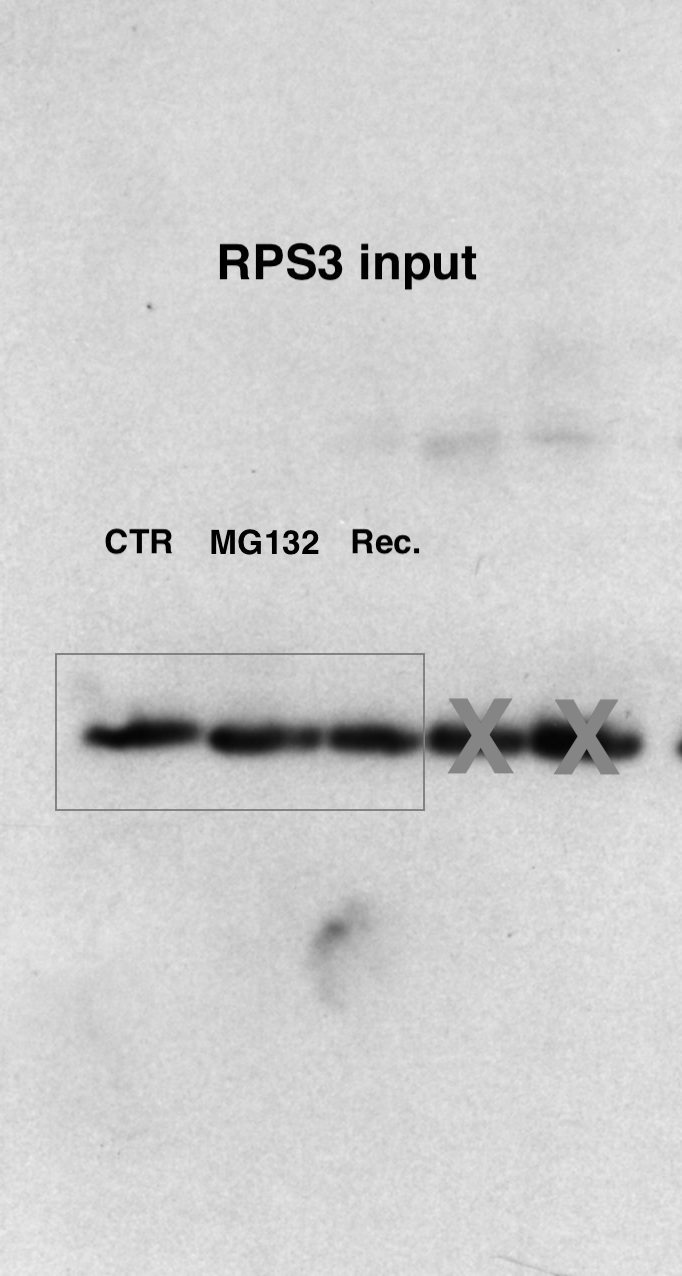

Supplement: Supplementary file 11 — Source data Fig. 3 [file 44318_2024_333_MOESM11_ESM.zip › Figure 3/Western blots_FIg 3/RPS3_Input.jpg]

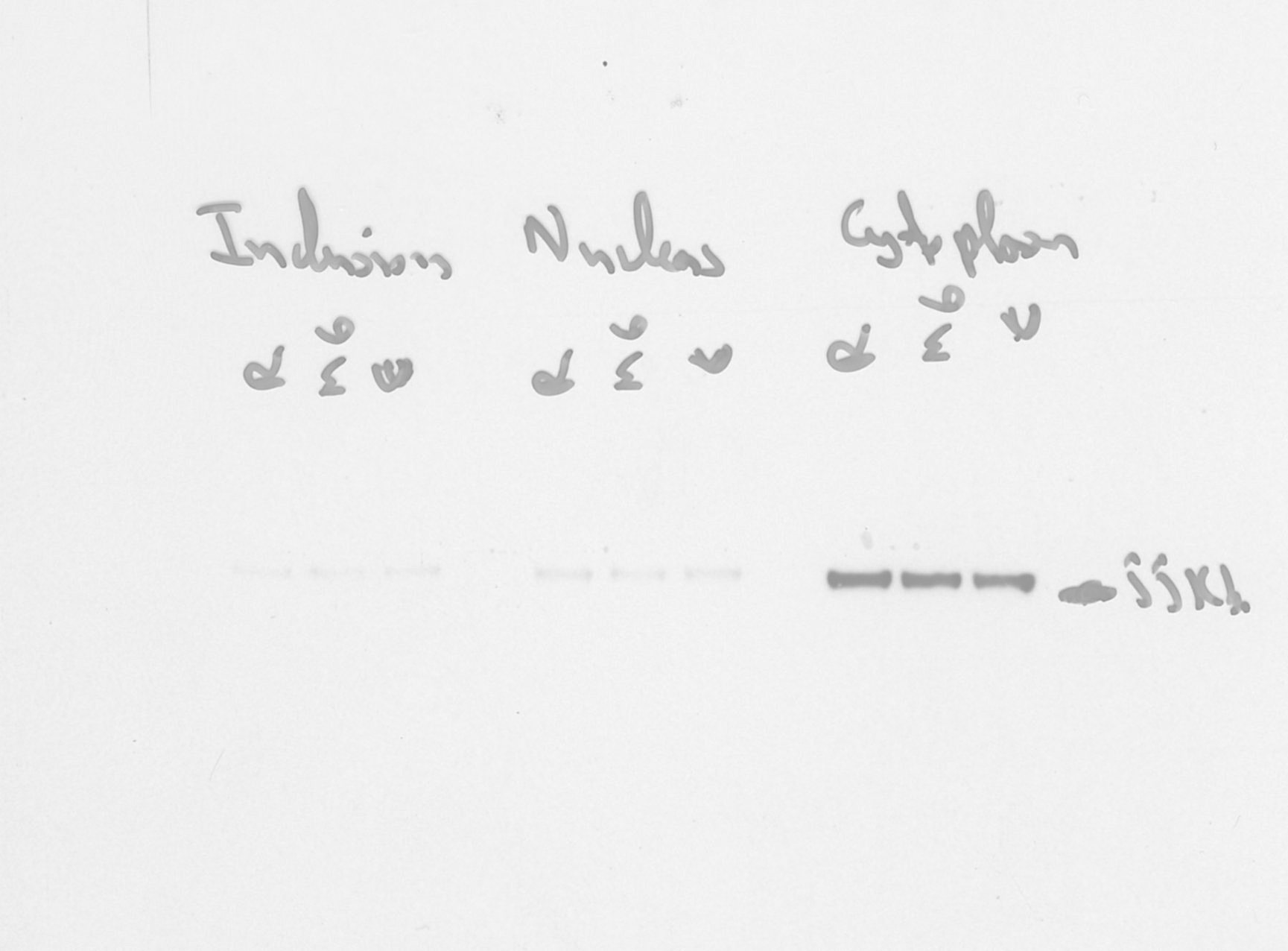

Supplement: Supplementary file 11 — Source data Fig. 3 [file 44318_2024_333_MOESM11_ESM.zip › Figure 3/Western blots_FIg 3/Tubulin_fraction.jpg]

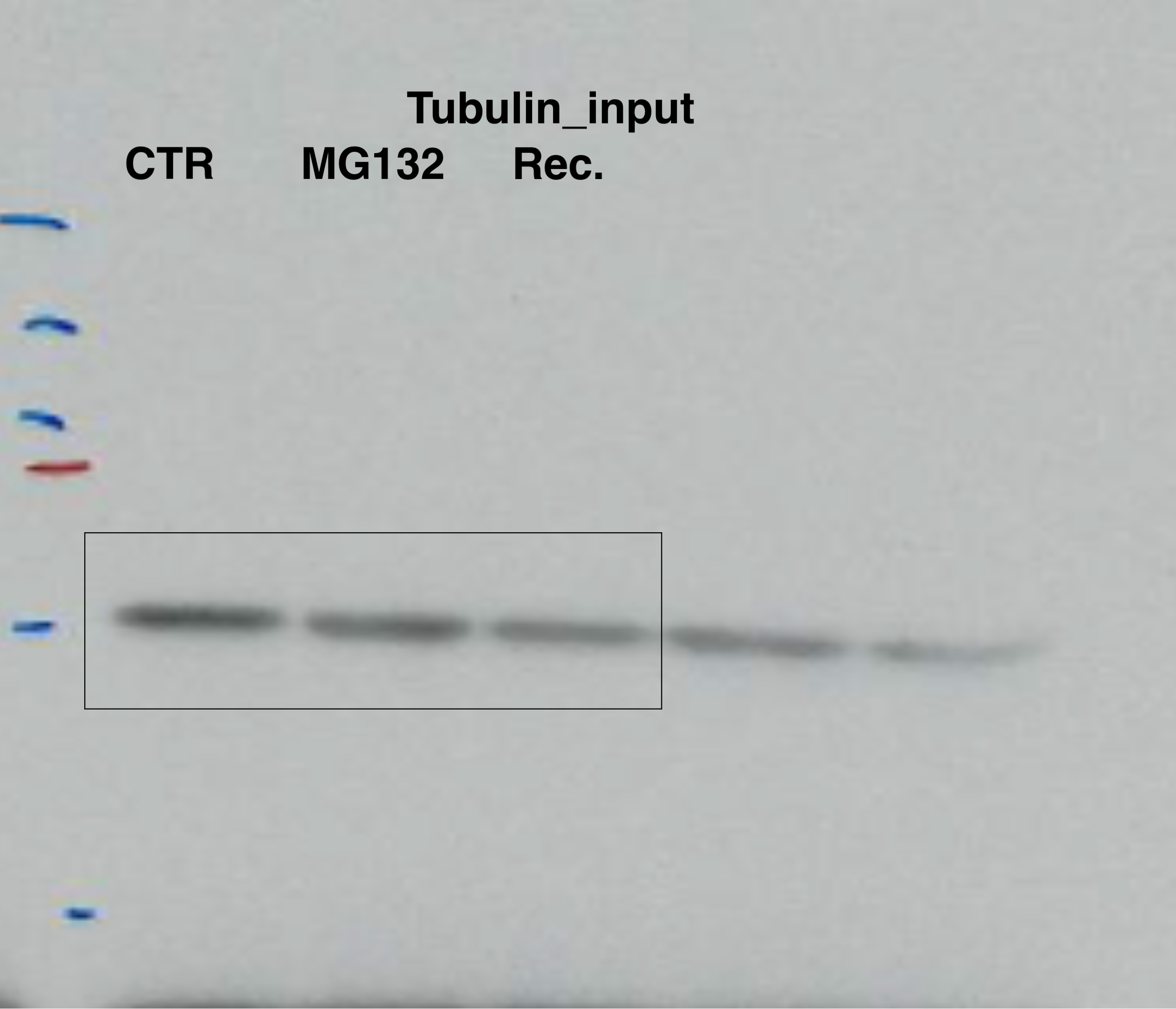

Supplement: Supplementary file 11 — Source data Fig. 3 [file 44318_2024_333_MOESM11_ESM.zip › Figure 3/Western blots_FIg 3/Tubulin_input.jpg]

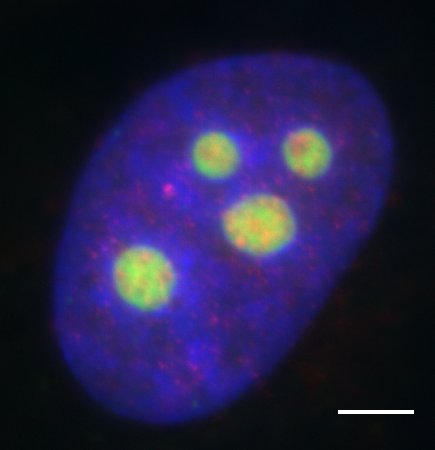

Supplement: Supplementary file 12 — Source data Fig. 4 [file 44318_2024_333_MOESM12_ESM.zip › Figure 4/Figure 4A/CTR/Composite.jpg]

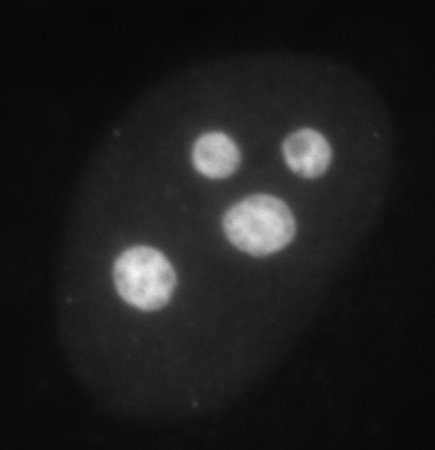

Supplement: Supplementary file 12 — Source data Fig. 4 [file 44318_2024_333_MOESM12_ESM.zip › Figure 4/Figure 4A/CTR/H12L11-GFP_inhibit proteasome during R n┬░4_DMSO_1_w1GFP.jpg]

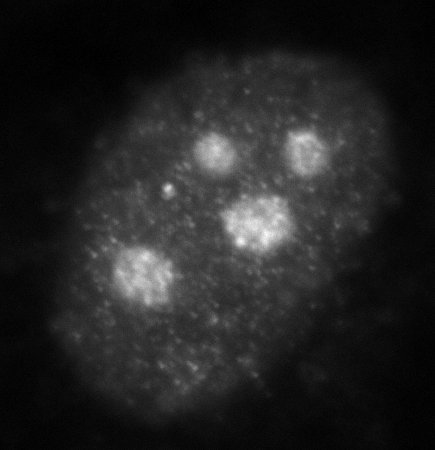

Supplement: Supplementary file 12 — Source data Fig. 4 [file 44318_2024_333_MOESM12_ESM.zip › Figure 4/Figure 4A/CTR/H12L11-GFP_inhibit proteasome during R n┬░4_DMSO_1_w2TexasRed.jpg]

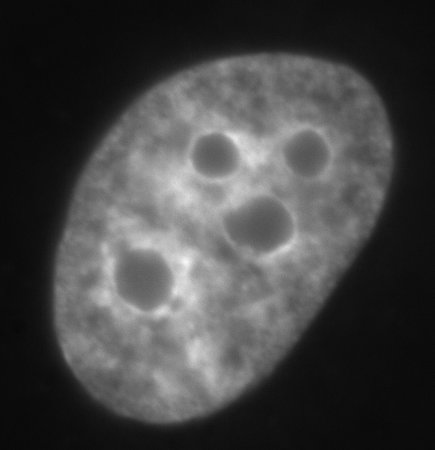

Supplement: Supplementary file 12 — Source data Fig. 4 [file 44318_2024_333_MOESM12_ESM.zip › Figure 4/Figure 4A/CTR/H12L11-GFP_inhibit proteasome during R n┬░4_DMSO_1_w3DAPI.jpg]

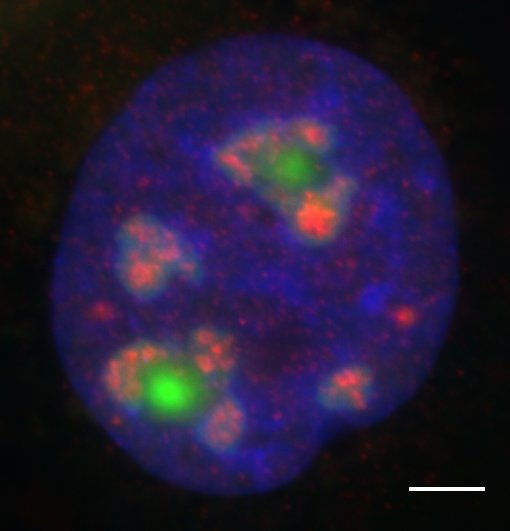

Supplement: Supplementary file 12 — Source data Fig. 4 [file 44318_2024_333_MOESM12_ESM.zip › Figure 4/Figure 4A/MG132/Composite.jpg]

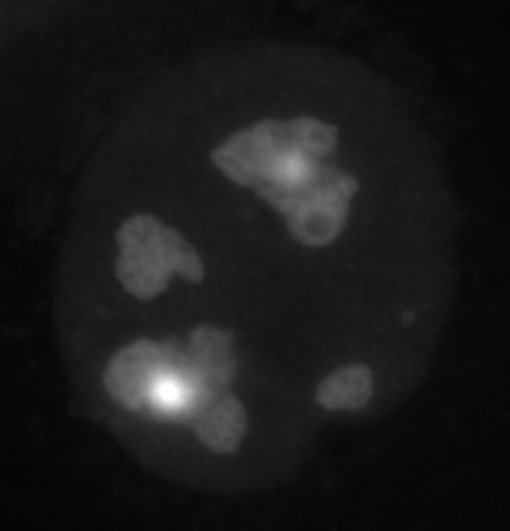

Supplement: Supplementary file 12 — Source data Fig. 4 [file 44318_2024_333_MOESM12_ESM.zip › Figure 4/Figure 4A/MG132/H12L11-GFP_inhibit proteasome during R n┬░4_mg132 ovn_1_w1GFP.jpg]

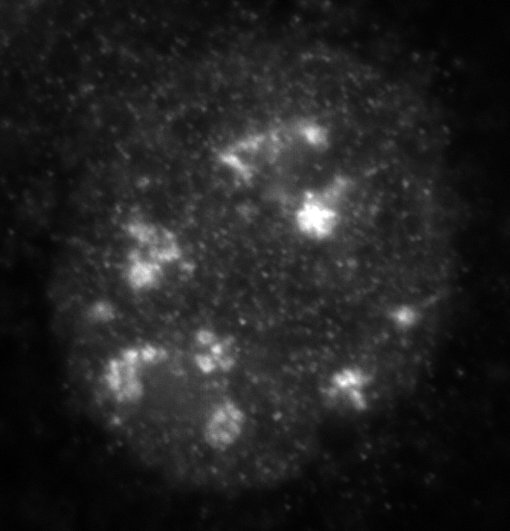

Supplement: Supplementary file 12 — Source data Fig. 4 [file 44318_2024_333_MOESM12_ESM.zip › Figure 4/Figure 4A/MG132/H12L11-GFP_inhibit proteasome during R n┬░4_mg132 ovn_1_w2TexasRed.jpg]

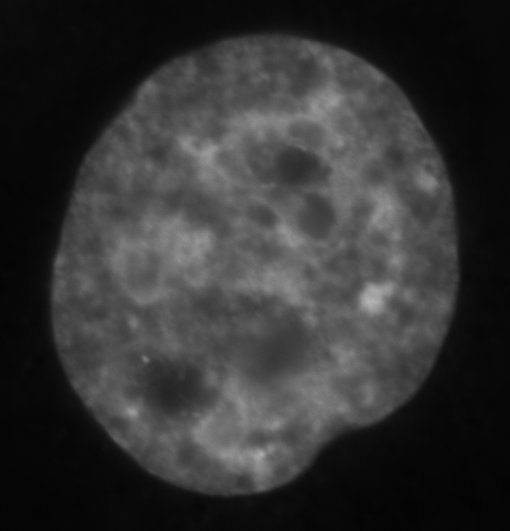

Supplement: Supplementary file 12 — Source data Fig. 4 [file 44318_2024_333_MOESM12_ESM.zip › Figure 4/Figure 4A/MG132/H12L11-GFP_inhibit proteasome during R n┬░4_mg132 ovn_1_w3DAPI.jpg]

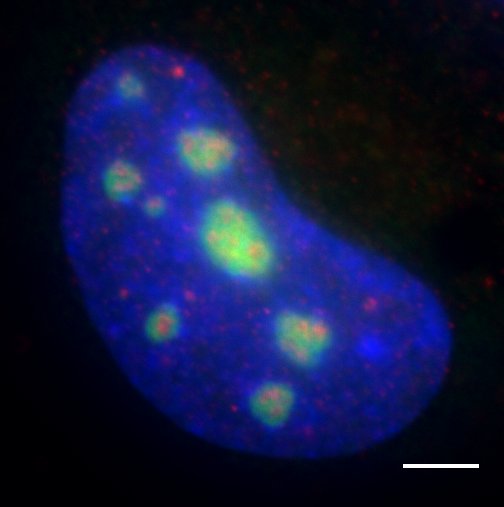

Supplement: Supplementary file 12 — Source data Fig. 4 [file 44318_2024_333_MOESM12_ESM.zip › Figure 4/Figure 4A/MG132 low doses/Composite.jpg]

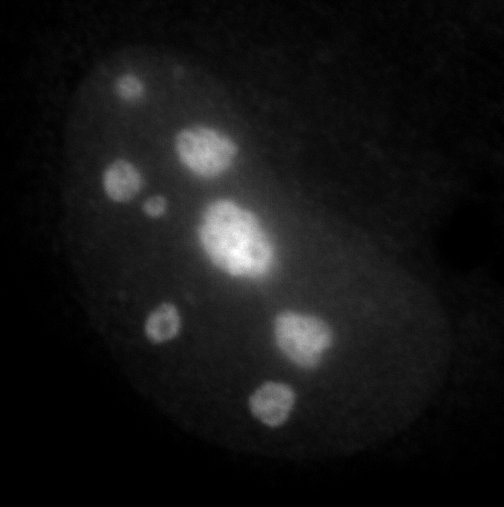

Supplement: Supplementary file 12 — Source data Fig. 4 [file 44318_2024_333_MOESM12_ESM.zip › Figure 4/Figure 4A/MG132 low doses/H12L11-GFP_inhibit proteasome during R n┬░4_mg132 low 8h_2_w1GFP.jpg]

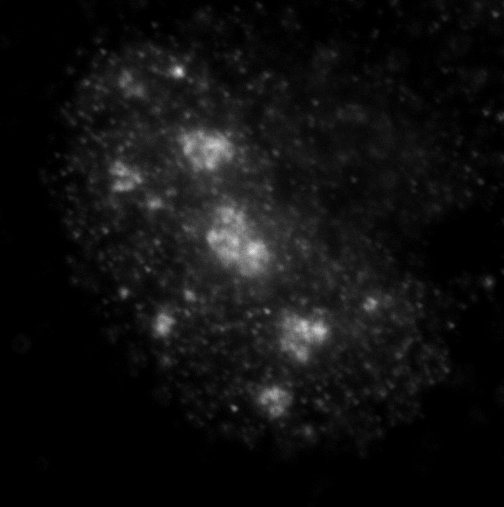

Supplement: Supplementary file 12 — Source data Fig. 4 [file 44318_2024_333_MOESM12_ESM.zip › Figure 4/Figure 4A/MG132 low doses/H12L11-GFP_inhibit proteasome during R n┬░4_mg132 low 8h_2_w2TexasRed.jpg]

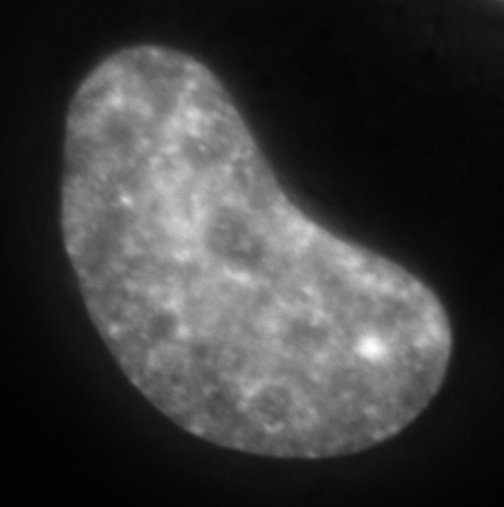

Supplement: Supplementary file 12 — Source data Fig. 4 [file 44318_2024_333_MOESM12_ESM.zip › Figure 4/Figure 4A/MG132 low doses/H12L11-GFP_inhibit proteasome during R n┬░4_mg132 low 8h_2_w3DAPI.jpg]

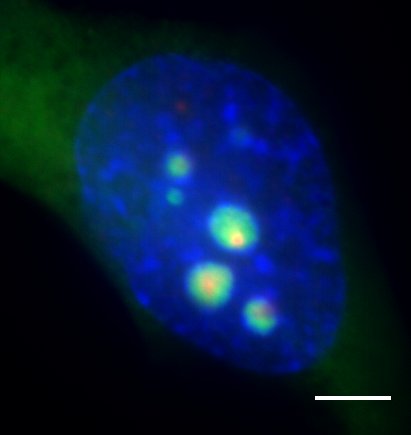

Supplement: Supplementary file 12 — Source data Fig. 4 [file 44318_2024_333_MOESM12_ESM.zip › Figure 4/Figure 4A/Recovery/Composite1.jpg]

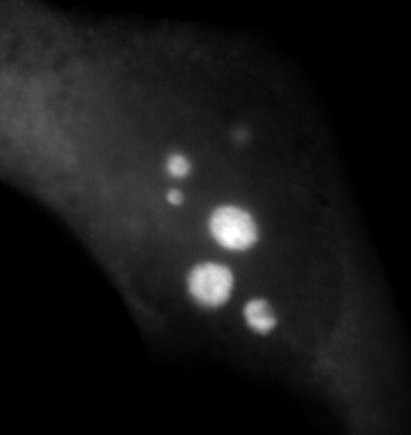

Supplement: Supplementary file 12 — Source data Fig. 4 [file 44318_2024_333_MOESM12_ESM.zip › Figure 4/Figure 4A/Recovery/H12L11-GFP_inhibit proteasome during R n┬░4_r8h_13_w1GFP.jpg]

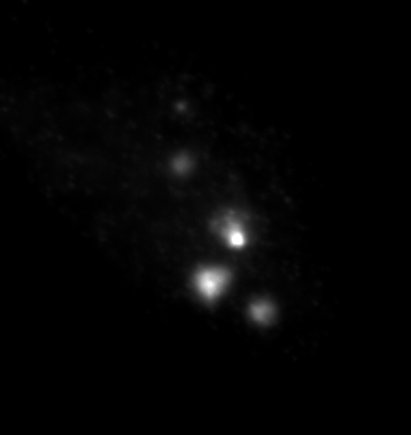

Supplement: Supplementary file 12 — Source data Fig. 4 [file 44318_2024_333_MOESM12_ESM.zip › Figure 4/Figure 4A/Recovery/H12L11-GFP_inhibit proteasome during R n┬░4_r8h_13_w2TexasRed.jpg]

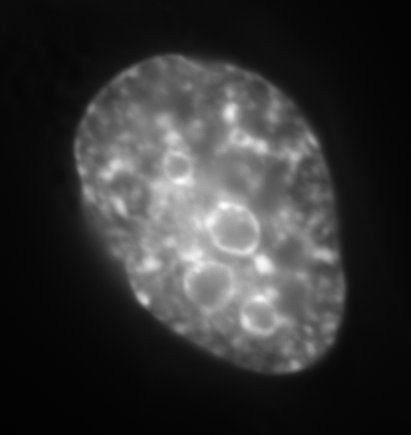

Supplement: Supplementary file 12 — Source data Fig. 4 [file 44318_2024_333_MOESM12_ESM.zip › Figure 4/Figure 4A/Recovery/H12L11-GFP_inhibit proteasome during R n┬░4_r8h_13_w3DAPI.jpg]

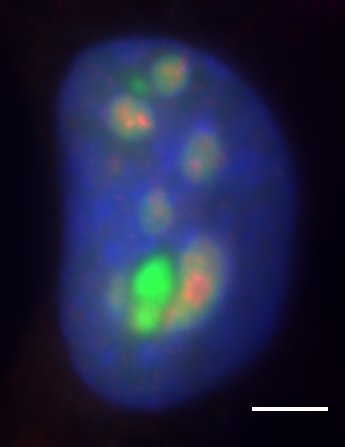

Supplement: Supplementary file 12 — Source data Fig. 4 [file 44318_2024_333_MOESM12_ESM.zip › Figure 4/Figure 4A/Recovery MG low doses/Composite1.jpg]

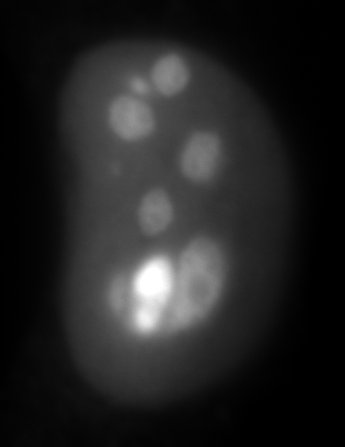

Supplement: Supplementary file 12 — Source data Fig. 4 [file 44318_2024_333_MOESM12_ESM.zip › Figure 4/Figure 4A/Recovery MG low doses/H12L11-GFP_inhibit proteasome during R n┬░4_r8h mg low_13_w1GFP.jpg]

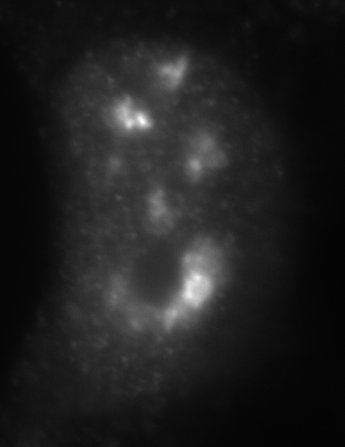

Supplement: Supplementary file 12 — Source data Fig. 4 [file 44318_2024_333_MOESM12_ESM.zip › Figure 4/Figure 4A/Recovery MG low doses/H12L11-GFP_inhibit proteasome during R n┬░4_r8h mg low_13_w2TexasRed.jpg]

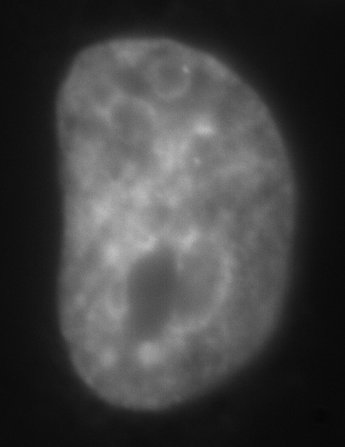

Supplement: Supplementary file 12 — Source data Fig. 4 [file 44318_2024_333_MOESM12_ESM.zip › Figure 4/Figure 4A/Recovery MG low doses/H12L11-GFP_inhibit proteasome during R n┬░4_r8h mg low_13_w3DAPI.jpg]

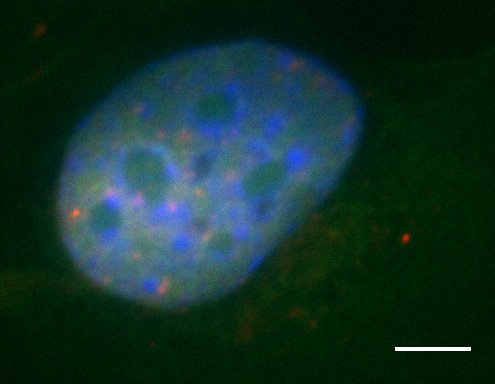

Supplement: Supplementary file 12 — Source data Fig. 4 [file 44318_2024_333_MOESM12_ESM.zip › Figure 4/Figure 4C/CTR/Composite_unt.jpg]

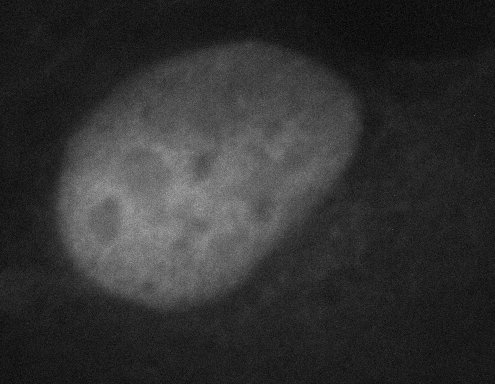

Supplement: Supplementary file 12 — Source data Fig. 4 [file 44318_2024_333_MOESM12_ESM.zip › Figure 4/Figure 4C/CTR/FL + ub_HS 2h exp_12022020_unt_1_w1GFP.jpg]

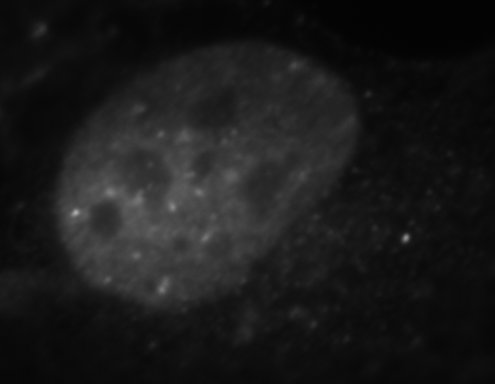

Supplement: Supplementary file 12 — Source data Fig. 4 [file 44318_2024_333_MOESM12_ESM.zip › Figure 4/Figure 4C/CTR/FL + ub_HS 2h exp_12022020_unt_1_w2Texas Red.jpg]

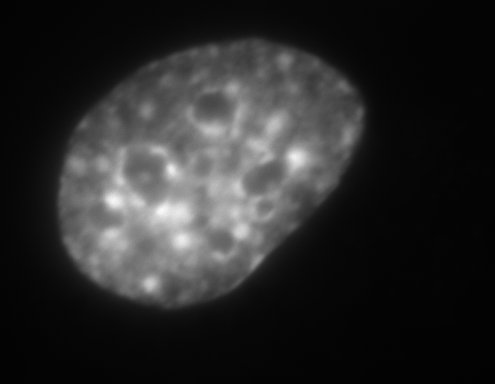

Supplement: Supplementary file 12 — Source data Fig. 4 [file 44318_2024_333_MOESM12_ESM.zip › Figure 4/Figure 4C/CTR/FL + ub_HS 2h exp_12022020_unt_1_w3Hoechst.jpg]

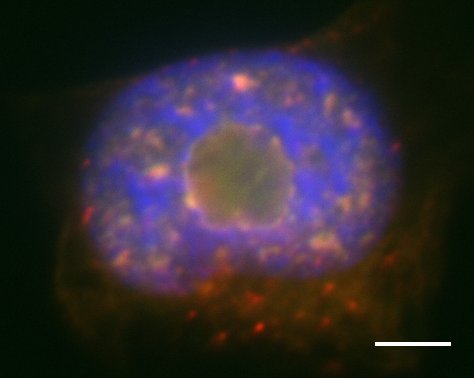

Supplement: Supplementary file 12 — Source data Fig. 4 [file 44318_2024_333_MOESM12_ESM.zip › Figure 4/Figure 4C/HS/Composite.jpg]

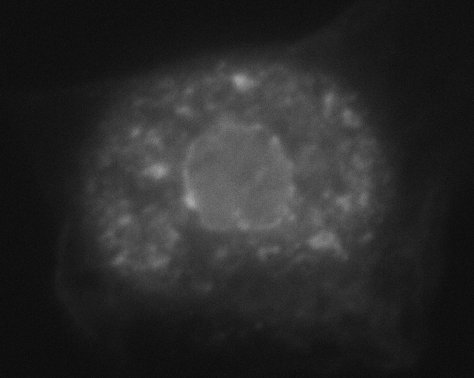

Supplement: Supplementary file 12 — Source data Fig. 4 [file 44318_2024_333_MOESM12_ESM.zip › Figure 4/Figure 4C/HS/FL + ub_HS 2h exp_12022020_hs 2h_1_w1GFP.jpg]

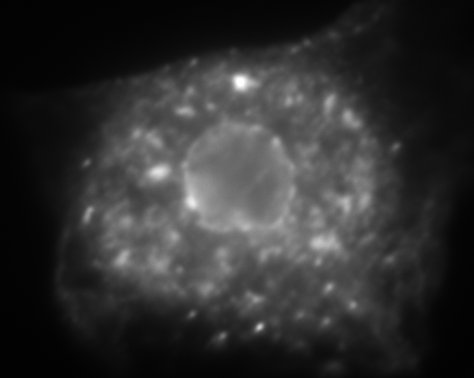

Supplement: Supplementary file 12 — Source data Fig. 4 [file 44318_2024_333_MOESM12_ESM.zip › Figure 4/Figure 4C/HS/FL + ub_HS 2h exp_12022020_hs 2h_1_w2Texas Red.jpg]

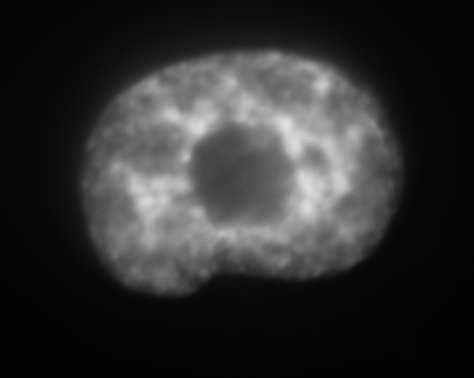

Supplement: Supplementary file 12 — Source data Fig. 4 [file 44318_2024_333_MOESM12_ESM.zip › Figure 4/Figure 4C/HS/FL + ub_HS 2h exp_12022020_hs 2h_1_w3Hoechst.jpg]

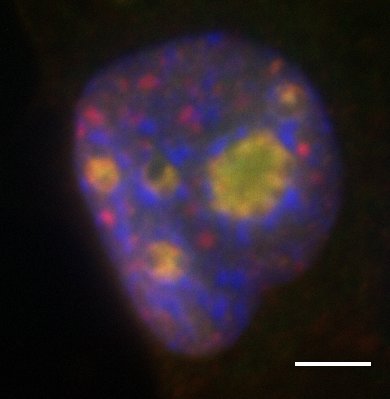

Supplement: Supplementary file 12 — Source data Fig. 4 [file 44318_2024_333_MOESM12_ESM.zip › Figure 4/Figure 4C/Recovery 2h/Composite.jpg]

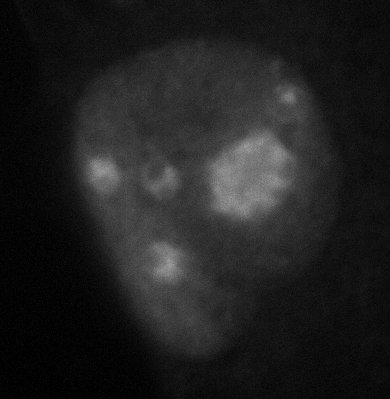

Supplement: Supplementary file 12 — Source data Fig. 4 [file 44318_2024_333_MOESM12_ESM.zip › Figure 4/Figure 4C/Recovery 2h/FL + ub_HS 2h exp_12022020_reco 2h__w1GFP.jpg]

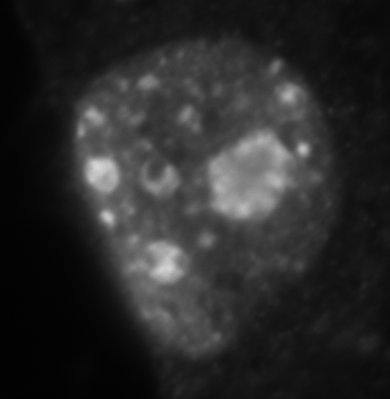

Supplement: Supplementary file 12 — Source data Fig. 4 [file 44318_2024_333_MOESM12_ESM.zip › Figure 4/Figure 4C/Recovery 2h/FL + ub_HS 2h exp_12022020_reco 2h__w2Texas Red.jpg]

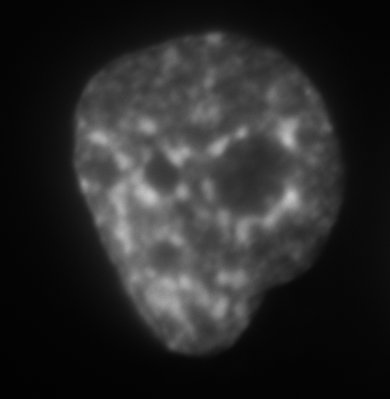

Supplement: Supplementary file 12 — Source data Fig. 4 [file 44318_2024_333_MOESM12_ESM.zip › Figure 4/Figure 4C/Recovery 2h/FL + ub_HS 2h exp_12022020_reco 2h__w3Hoechst.jpg]

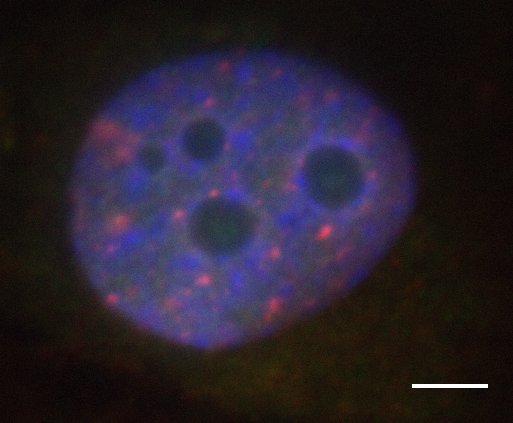

Supplement: Supplementary file 12 — Source data Fig. 4 [file 44318_2024_333_MOESM12_ESM.zip › Figure 4/Figure 4C/Recovery 6h/Composite.jpg]

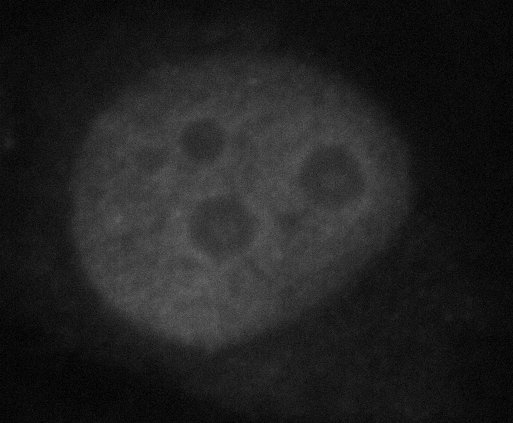

Supplement: Supplementary file 12 — Source data Fig. 4 [file 44318_2024_333_MOESM12_ESM.zip › Figure 4/Figure 4C/Recovery 6h/FL + ub_HS 2h exp_12022020_reco 6h_5_w1GFP.jpg]

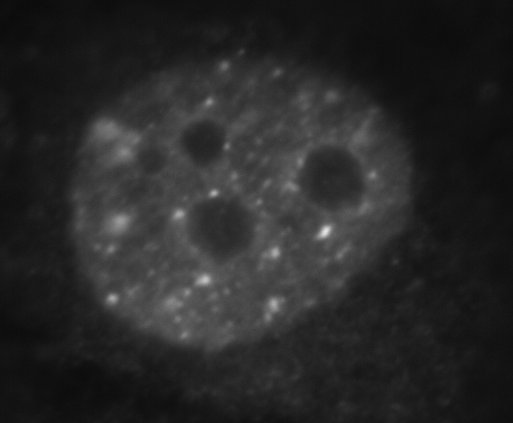

Supplement: Supplementary file 12 — Source data Fig. 4 [file 44318_2024_333_MOESM12_ESM.zip › Figure 4/Figure 4C/Recovery 6h/FL + ub_HS 2h exp_12022020_reco 6h_5_w2Texas Red.jpg]

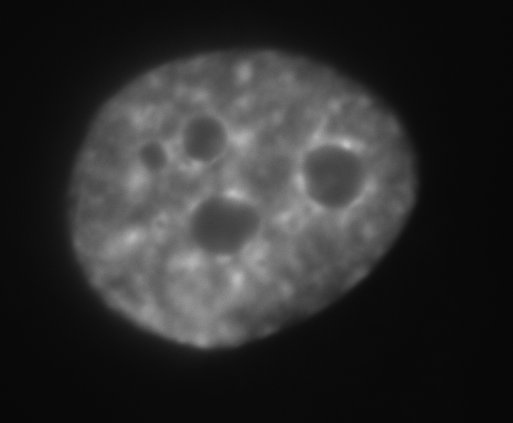

Supplement: Supplementary file 12 — Source data Fig. 4 [file 44318_2024_333_MOESM12_ESM.zip › Figure 4/Figure 4C/Recovery 6h/FL + ub_HS 2h exp_12022020_reco 6h_5_w3Hoechst.jpg]

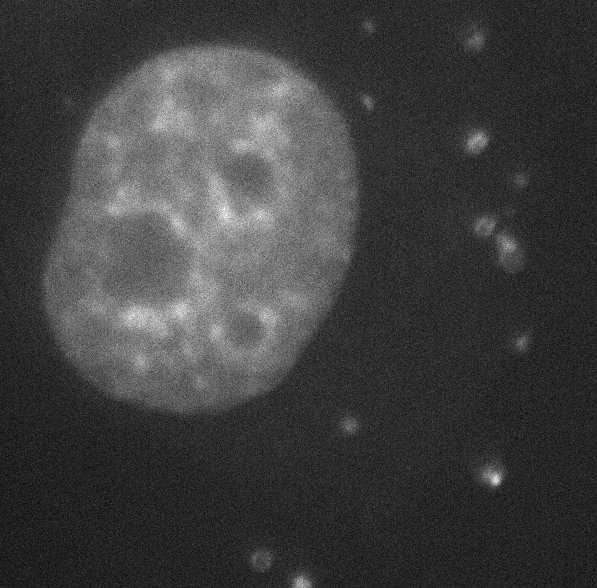

Supplement: Supplementary file 12 — Source data Fig. 4 [file 44318_2024_333_MOESM12_ESM.zip › Figure 4/Figure 4D/Lactac. Recovery HS/Inhibit Prot_FL_310720_R2h beta lactac.r_8_w2Hoechst.jpg]

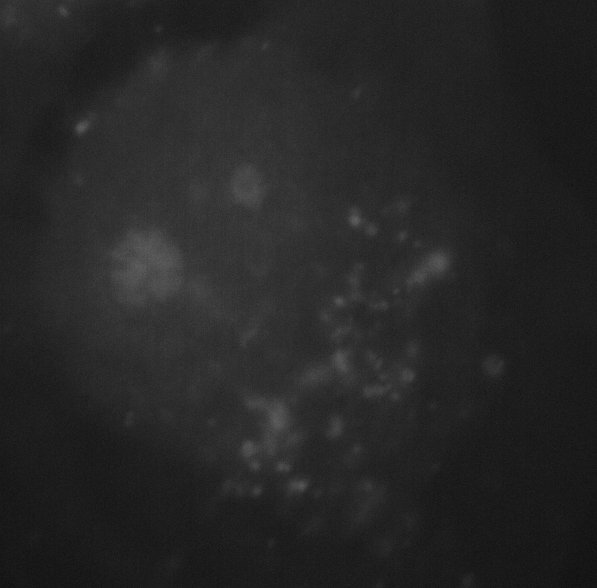

Supplement: Supplementary file 12 — Source data Fig. 4 [file 44318_2024_333_MOESM12_ESM.zip › Figure 4/Figure 4D/Lactac. Recovery HS/Inhibit Prot_FL_310720_R2h beta lactacr_8_w1GFP.jpg]

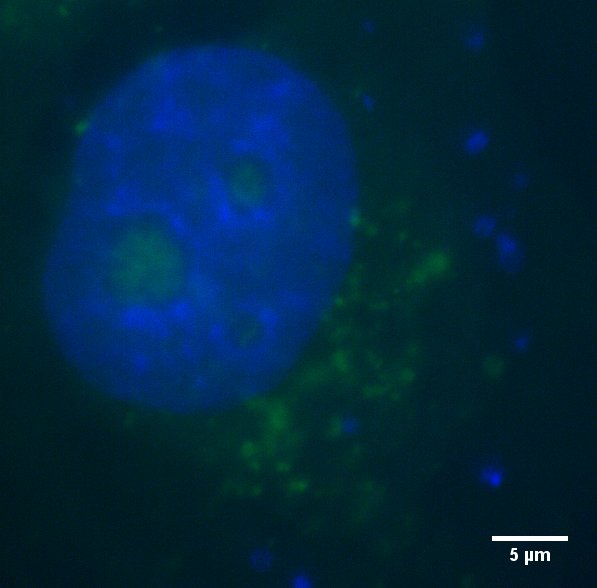

Supplement: Supplementary file 12 — Source data Fig. 4 [file 44318_2024_333_MOESM12_ESM.zip › Figure 4/Figure 4D/Lactac. Recovery HS/R2h beta lact. scale.jpg]

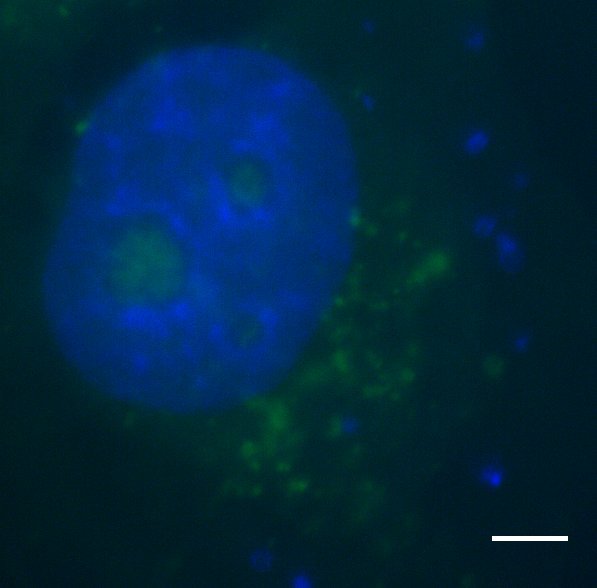

Supplement: Supplementary file 12 — Source data Fig. 4 [file 44318_2024_333_MOESM12_ESM.zip › Figure 4/Figure 4D/Lactac. Recovery HS/R2h beta lact..jpg]

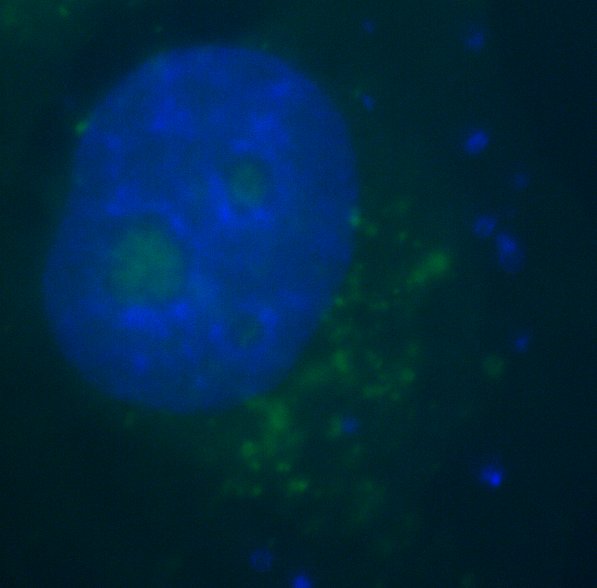

Supplement: Supplementary file 12 — Source data Fig. 4 [file 44318_2024_333_MOESM12_ESM.zip › Figure 4/Figure 4D/Lactac. Recovery HS/R2h beta lact.jpg]

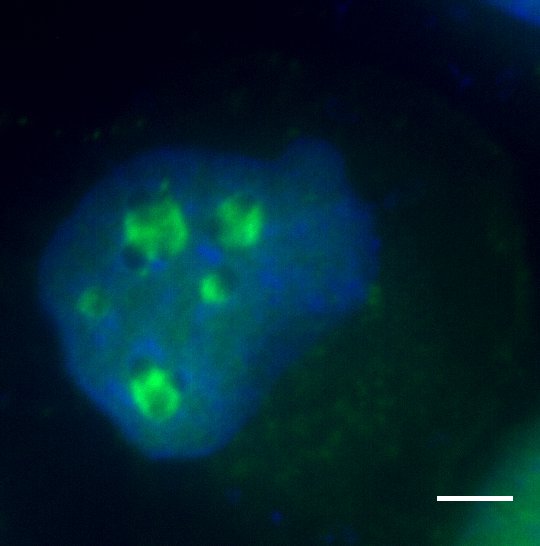

Supplement: Supplementary file 12 — Source data Fig. 4 [file 44318_2024_333_MOESM12_ESM.zip › Figure 4/Figure 4D/Recovery HS/Composite_R2h eee.jpg]

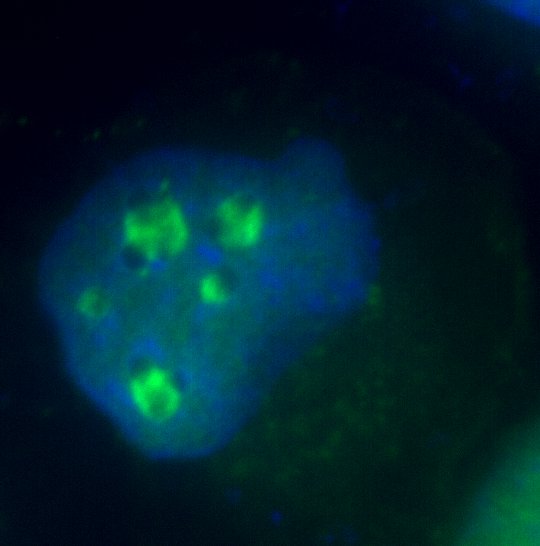

Supplement: Supplementary file 12 — Source data Fig. 4 [file 44318_2024_333_MOESM12_ESM.zip › Figure 4/Figure 4D/Recovery HS/Composite_R2h.jpg]

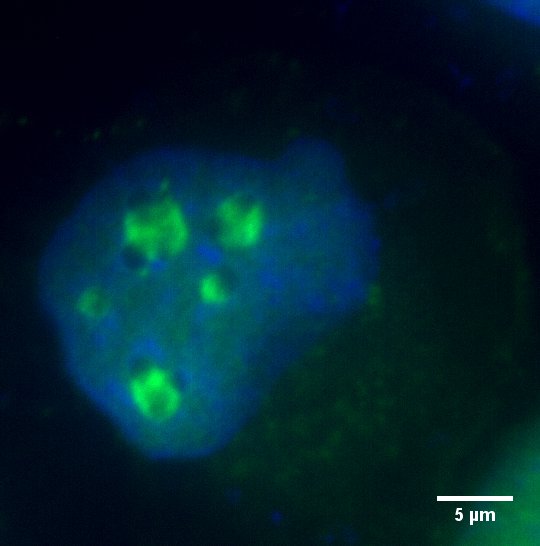

Supplement: Supplementary file 12 — Source data Fig. 4 [file 44318_2024_333_MOESM12_ESM.zip › Figure 4/Figure 4D/Recovery HS/Composite_R2h_scale.jpg]

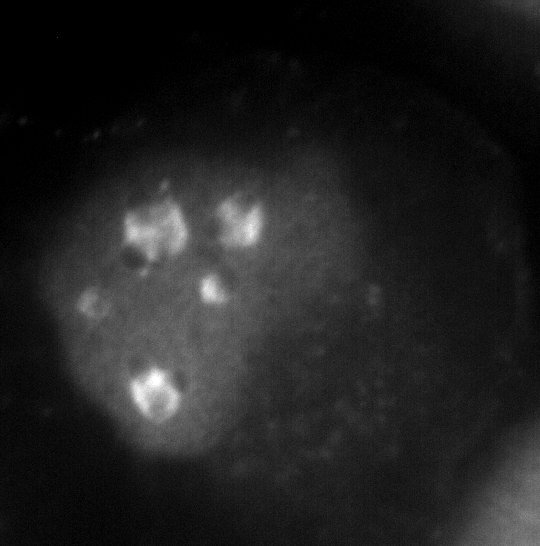

Supplement: Supplementary file 12 — Source data Fig. 4 [file 44318_2024_333_MOESM12_ESM.zip › Figure 4/Figure 4D/Recovery HS/Inhibit Prot_FL_310720_R2h_7_w1GFP.jpg]

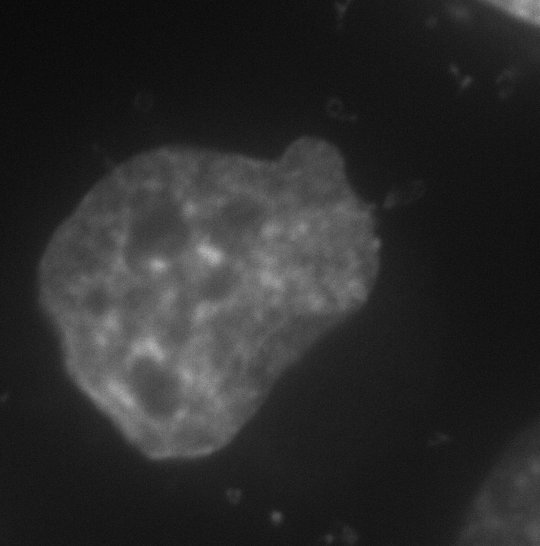

Supplement: Supplementary file 12 — Source data Fig. 4 [file 44318_2024_333_MOESM12_ESM.zip › Figure 4/Figure 4D/Recovery HS/Inhibit Prot_FL_310720_R2h_7_w2Hoechst.jpg]

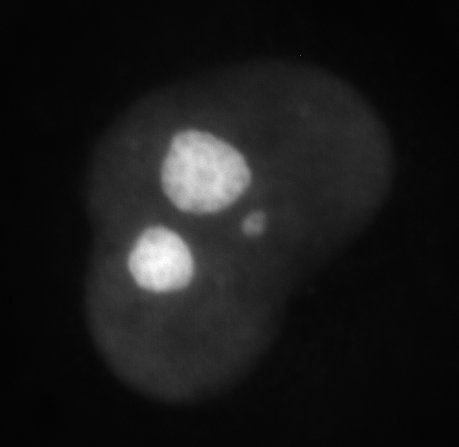

Supplement: Supplementary file 13 — Source data Fig. 5 [file 44318_2024_333_MOESM13_ESM.zip › Figure 5/Figure 5C/CTR/181219_RPL11 GFP H12_nedd8 exp_no stress_2_w1GFP.jpg]

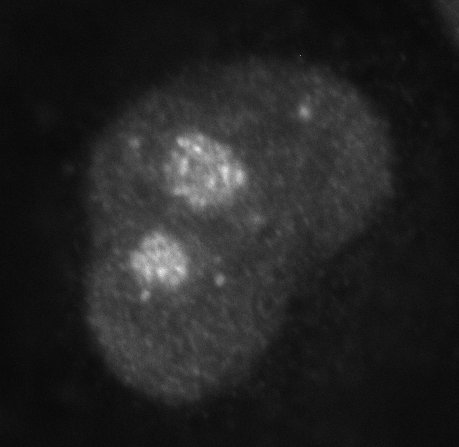

Supplement: Supplementary file 13 — Source data Fig. 5 [file 44318_2024_333_MOESM13_ESM.zip › Figure 5/Figure 5C/CTR/181219_RPL11 GFP H12_nedd8 exp_no stress_2_w2TexasRed.jpg]

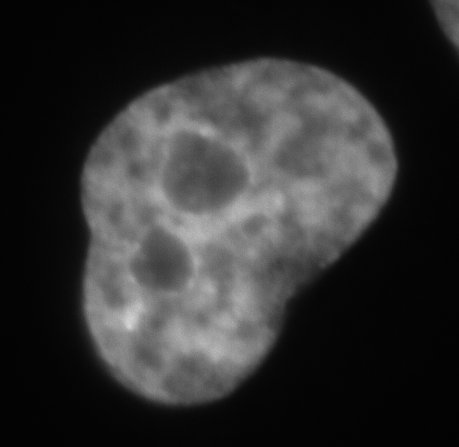

Supplement: Supplementary file 13 — Source data Fig. 5 [file 44318_2024_333_MOESM13_ESM.zip › Figure 5/Figure 5C/CTR/181219_RPL11 GFP H12_nedd8 exp_no stress_2_w3DAPI.jpg]

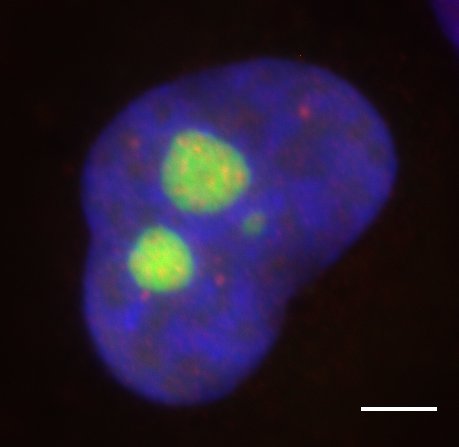

Supplement: Supplementary file 13 — Source data Fig. 5 [file 44318_2024_333_MOESM13_ESM.zip › Figure 5/Figure 5C/CTR/Composite.jpg]

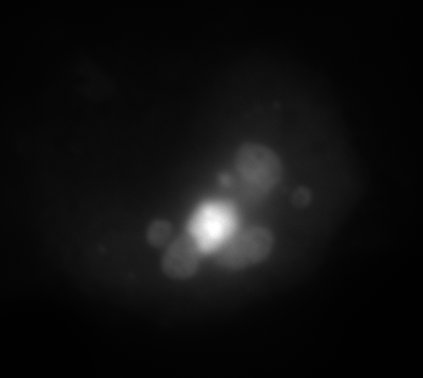

Supplement: Supplementary file 13 — Source data Fig. 5 [file 44318_2024_333_MOESM13_ESM.zip › Figure 5/Figure 5C/MG132/181219_RPL11 GFP H12_ubai exp_MG132_3_w1GFP.jpg]

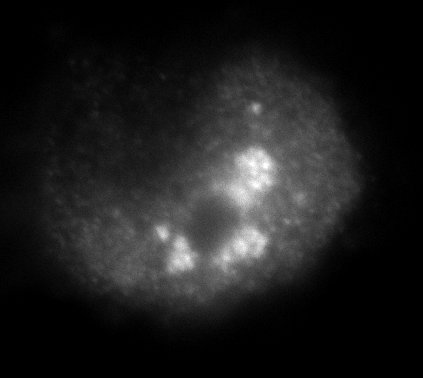

Supplement: Supplementary file 13 — Source data Fig. 5 [file 44318_2024_333_MOESM13_ESM.zip › Figure 5/Figure 5C/MG132/181219_RPL11 GFP H12_ubai exp_MG132_3_w2TexasRed.jpg]

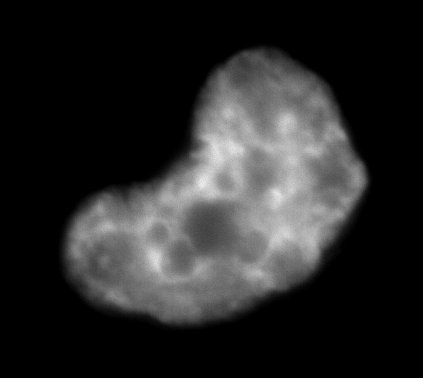

Supplement: Supplementary file 13 — Source data Fig. 5 [file 44318_2024_333_MOESM13_ESM.zip › Figure 5/Figure 5C/MG132/181219_RPL11 GFP H12_ubai exp_MG132_3_w3DAPI.jpg]

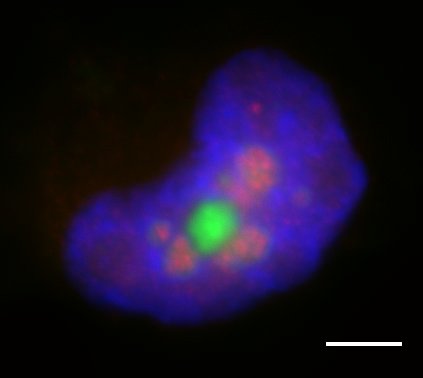

Supplement: Supplementary file 13 — Source data Fig. 5 [file 44318_2024_333_MOESM13_ESM.zip › Figure 5/Figure 5C/MG132/Composite.jpg]

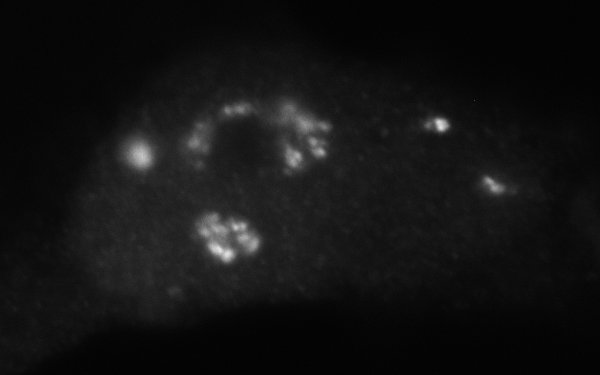

Supplement: Supplementary file 13 — Source data Fig. 5 [file 44318_2024_333_MOESM13_ESM.zip › Figure 5/Figure 5C/MG132 MLN ubaie/(mln ubai) 190111_mln mg132_6_w1TexasRed-1.jpg]

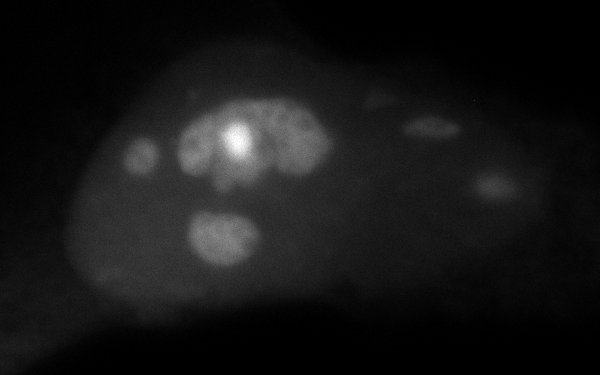

Supplement: Supplementary file 13 — Source data Fig. 5 [file 44318_2024_333_MOESM13_ESM.zip › Figure 5/Figure 5C/MG132 MLN ubaie/(mln ubai) 190111_mln mg132_6_w2GFP.jpg]

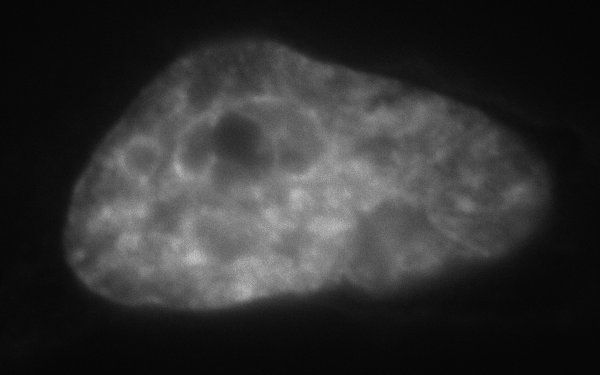

Supplement: Supplementary file 13 — Source data Fig. 5 [file 44318_2024_333_MOESM13_ESM.zip › Figure 5/Figure 5C/MG132 MLN ubaie/(mln ubai) 190111_mln mg132_6_w3DAPI.jpg]

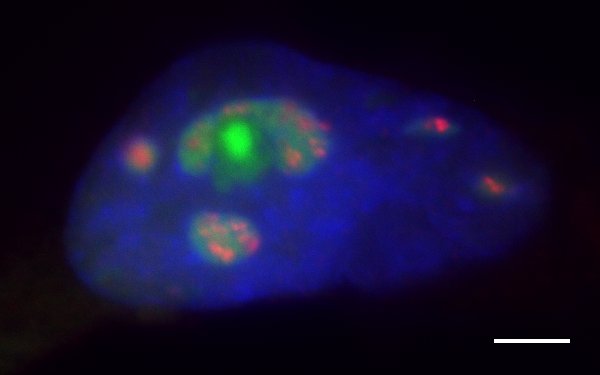

Supplement: Supplementary file 13 — Source data Fig. 5 [file 44318_2024_333_MOESM13_ESM.zip › Figure 5/Figure 5C/MG132 MLN ubaie/Composite.jpg]

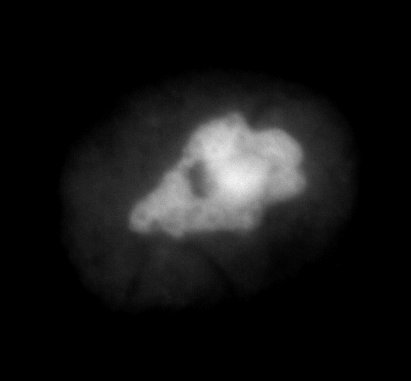

Supplement: Supplementary file 13 — Source data Fig. 5 [file 44318_2024_333_MOESM13_ESM.zip › Figure 5/Figure 5C/MLN NAEi MG132/181219_RPL11 GFP H12_nedd8 exp_MG132 with mln nedd8_21_w1GFP.jpg]

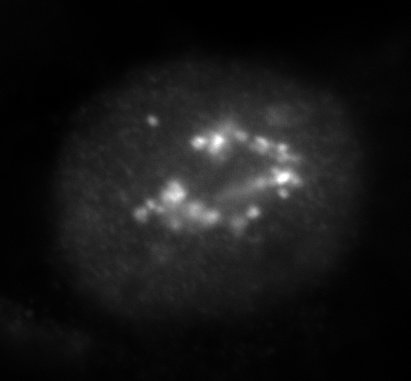

Supplement: Supplementary file 13 — Source data Fig. 5 [file 44318_2024_333_MOESM13_ESM.zip › Figure 5/Figure 5C/MLN NAEi MG132/181219_RPL11 GFP H12_nedd8 exp_MG132 with mln nedd8_21_w2TexasRed.jpg]

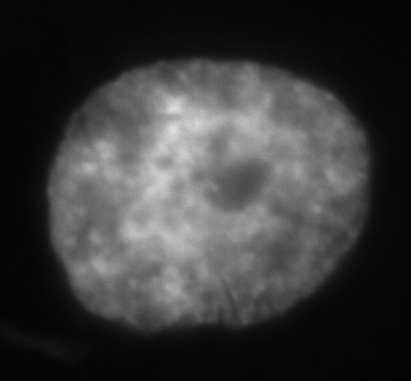

Supplement: Supplementary file 13 — Source data Fig. 5 [file 44318_2024_333_MOESM13_ESM.zip › Figure 5/Figure 5C/MLN NAEi MG132/181219_RPL11 GFP H12_nedd8 exp_MG132 with mln nedd8_21_w3DAPI.jpg]

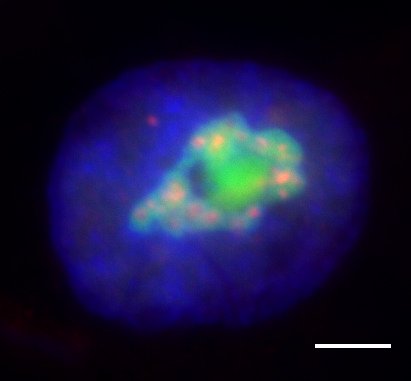

Supplement: Supplementary file 13 — Source data Fig. 5 [file 44318_2024_333_MOESM13_ESM.zip › Figure 5/Figure 5C/MLN NAEi MG132/Composite.jpg]

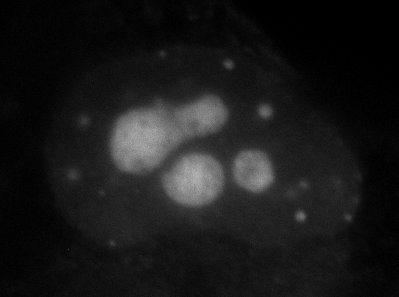

Supplement: Supplementary file 13 — Source data Fig. 5 [file 44318_2024_333_MOESM13_ESM.zip › Figure 5/Figure 5C/NAEi/181219_RPL11 GFP H12_nedd8 exp_no stress nedd8 mln_14_w1GFP.jpg]

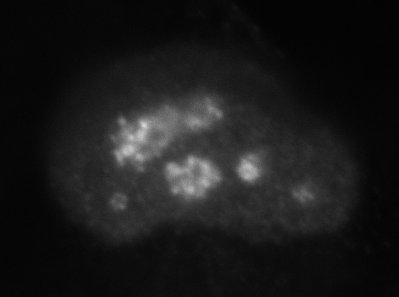

Supplement: Supplementary file 13 — Source data Fig. 5 [file 44318_2024_333_MOESM13_ESM.zip › Figure 5/Figure 5C/NAEi/181219_RPL11 GFP H12_nedd8 exp_no stress nedd8 mln_14_w2TexasRed.jpg]

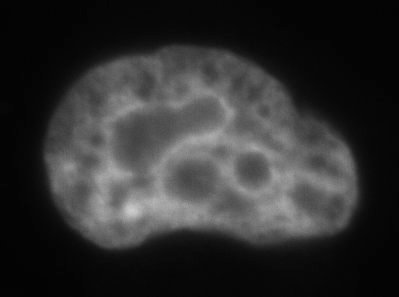

Supplement: Supplementary file 13 — Source data Fig. 5 [file 44318_2024_333_MOESM13_ESM.zip › Figure 5/Figure 5C/NAEi/181219_RPL11 GFP H12_nedd8 exp_no stress nedd8 mln_14_w3DAPI.jpg]

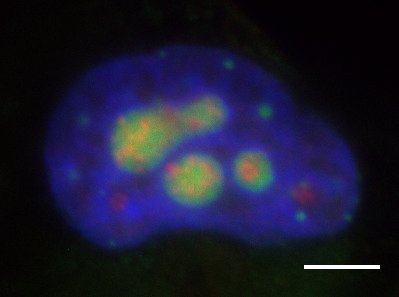

Supplement: Supplementary file 13 — Source data Fig. 5 [file 44318_2024_333_MOESM13_ESM.zip › Figure 5/Figure 5C/NAEi/Composite.jpg]

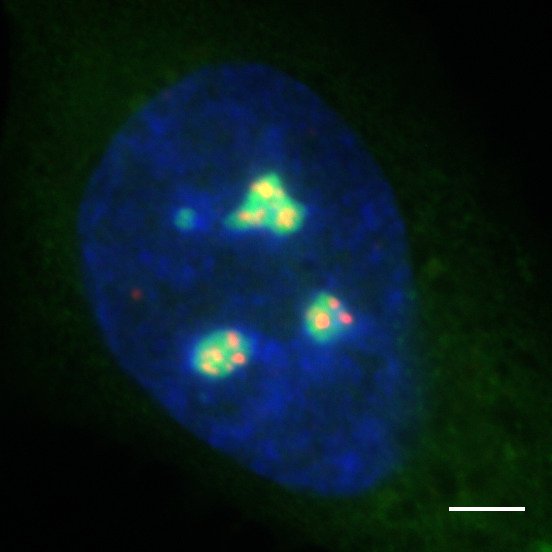

Supplement: Supplementary file 13 — Source data Fig. 5 [file 44318_2024_333_MOESM13_ESM.zip › Figure 5/Figure 5C/NAEi R8h/Composite.jpg]

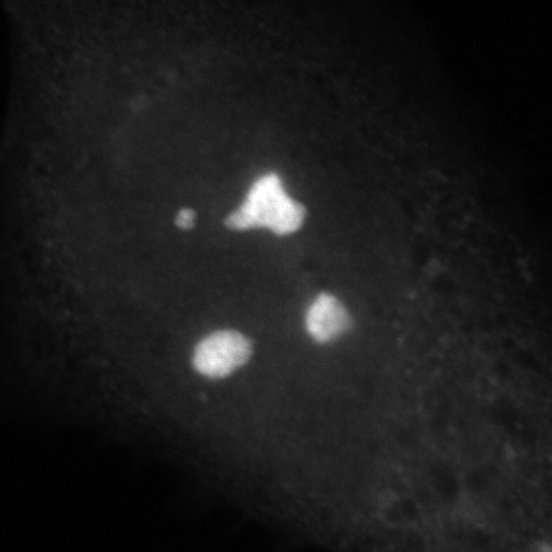

Supplement: Supplementary file 13 — Source data Fig. 5 [file 44318_2024_333_MOESM13_ESM.zip › Figure 5/Figure 5C/NAEi R8h/H12RPL11-GFP_MLN nedd8 recovery8h_1_w1GFP.jpg]

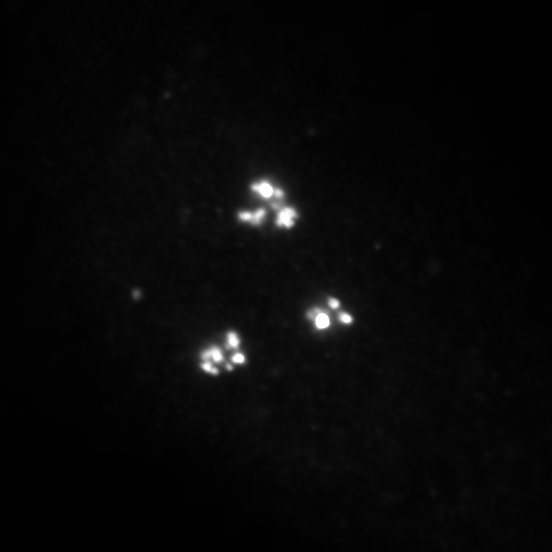

Supplement: Supplementary file 13 — Source data Fig. 5 [file 44318_2024_333_MOESM13_ESM.zip › Figure 5/Figure 5C/NAEi R8h/H12RPL11-GFP_MLN nedd8 recovery8h_1_w2TexasRed.jpg]

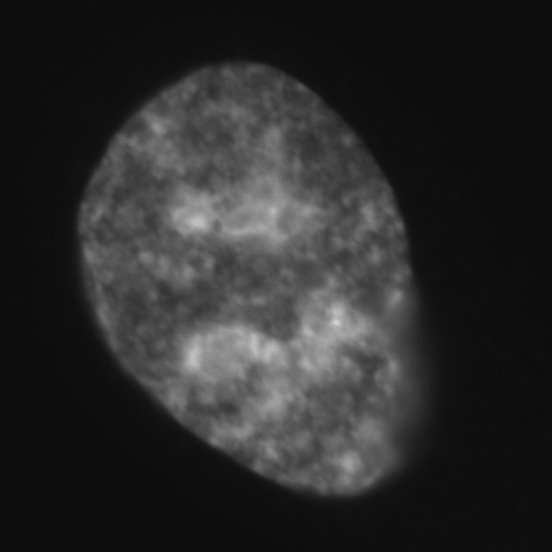

Supplement: Supplementary file 13 — Source data Fig. 5 [file 44318_2024_333_MOESM13_ESM.zip › Figure 5/Figure 5C/NAEi R8h/H12RPL11-GFP_MLN nedd8 recovery8h_1_w3DAPI.jpg]

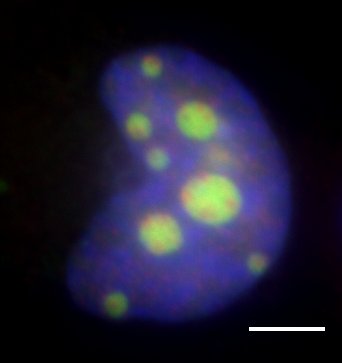

Supplement: Supplementary file 13 — Source data Fig. 5 [file 44318_2024_333_MOESM13_ESM.zip › Figure 5/Figure 5C/Recovery 8h/Composite.jpg]

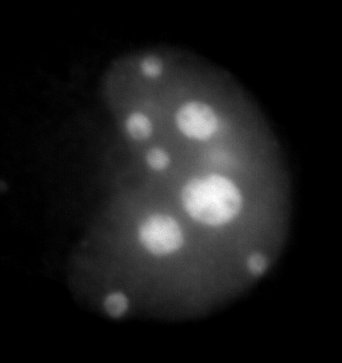

Supplement: Supplementary file 13 — Source data Fig. 5 [file 44318_2024_333_MOESM13_ESM.zip › Figure 5/Figure 5C/Recovery 8h/H12L11GFP_MLN n┬░1_fibrillarin staining_mg132 reco 8h_1_w1GFP.jpg]

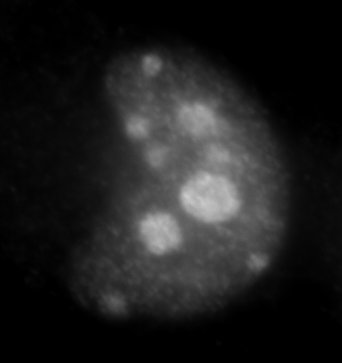

Supplement: Supplementary file 13 — Source data Fig. 5 [file 44318_2024_333_MOESM13_ESM.zip › Figure 5/Figure 5C/Recovery 8h/H12L11GFP_MLN n┬░1_fibrillarin staining_mg132 reco 8h_1_w2TexasRed.jpg]

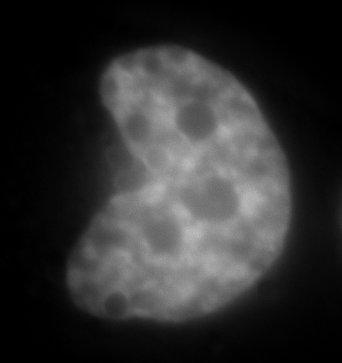

Supplement: Supplementary file 13 — Source data Fig. 5 [file 44318_2024_333_MOESM13_ESM.zip › Figure 5/Figure 5C/Recovery 8h/H12L11GFP_MLN n┬░1_fibrillarin staining_mg132 reco 8h_1_w3DAPI.jpg]

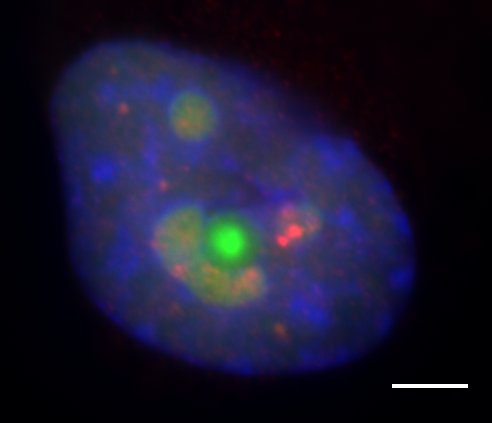

Supplement: Supplementary file 13 — Source data Fig. 5 [file 44318_2024_333_MOESM13_ESM.zip › Figure 5/Figure 5C/Recovery 8h Ubai/Composite.jpg]

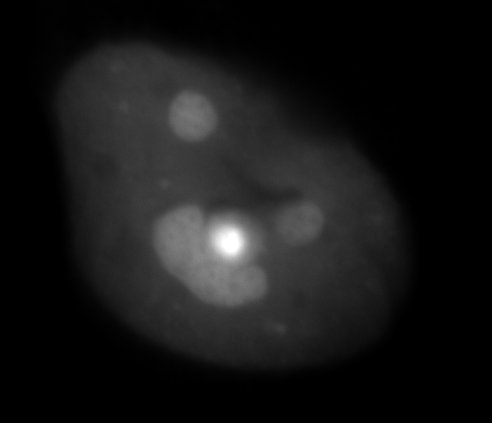

Supplement: Supplementary file 13 — Source data Fig. 5 [file 44318_2024_333_MOESM13_ESM.zip › Figure 5/Figure 5C/Recovery 8h Ubai/H12L11GFP_MLN n┬░1_fibrillarin staining_mg132 reco 8h mln ubai_34_w1GFP.jpg]

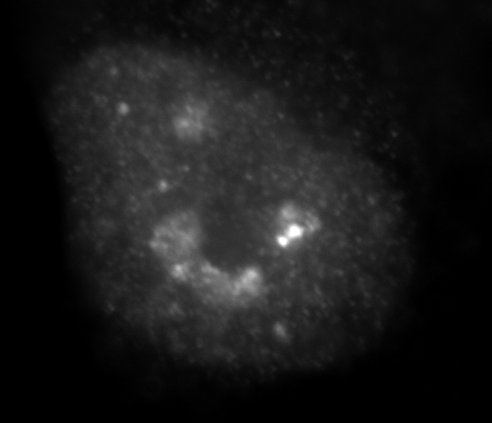

Supplement: Supplementary file 13 — Source data Fig. 5 [file 44318_2024_333_MOESM13_ESM.zip › Figure 5/Figure 5C/Recovery 8h Ubai/H12L11GFP_MLN n┬░1_fibrillarin staining_mg132 reco 8h mln ubai_34_w2TexasRed.jpg]

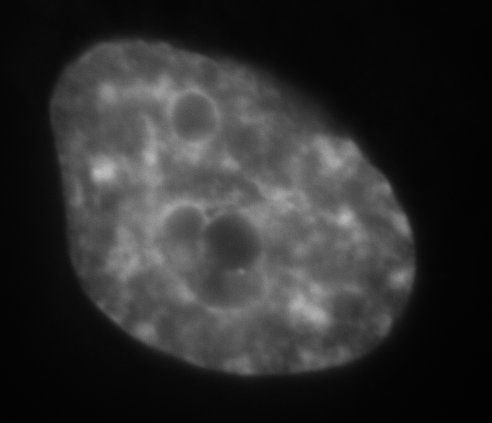

Supplement: Supplementary file 13 — Source data Fig. 5 [file 44318_2024_333_MOESM13_ESM.zip › Figure 5/Figure 5C/Recovery 8h Ubai/H12L11GFP_MLN n┬░1_fibrillarin staining_mg132 reco 8h mln ubai_34_w3DAPI.jpg]

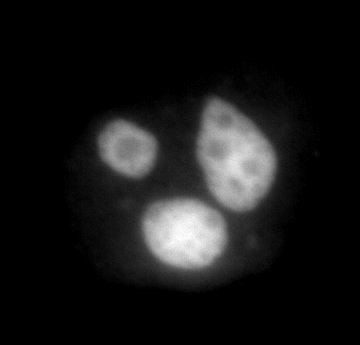

Supplement: Supplementary file 13 — Source data Fig. 5 [file 44318_2024_333_MOESM13_ESM.zip › Figure 5/Figure 5C/Ubai/181219_RPL11 GFP H12_ubai exp_no stress mln_10_w1GFP.jpg]

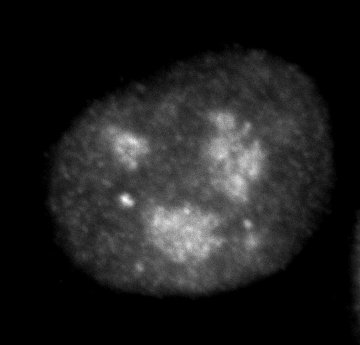

Supplement: Supplementary file 13 — Source data Fig. 5 [file 44318_2024_333_MOESM13_ESM.zip › Figure 5/Figure 5C/Ubai/181219_RPL11 GFP H12_ubai exp_no stress mln_10_w2TexasRed.jpg]

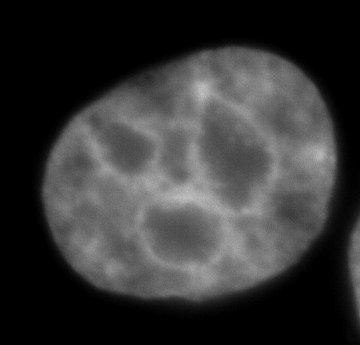

Supplement: Supplementary file 13 — Source data Fig. 5 [file 44318_2024_333_MOESM13_ESM.zip › Figure 5/Figure 5C/Ubai/181219_RPL11 GFP H12_ubai exp_no stress mln_10_w3DAPI.jpg]

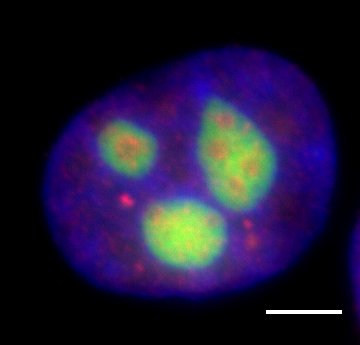

Supplement: Supplementary file 13 — Source data Fig. 5 [file 44318_2024_333_MOESM13_ESM.zip › Figure 5/Figure 5C/Ubai/Composite.jpg]

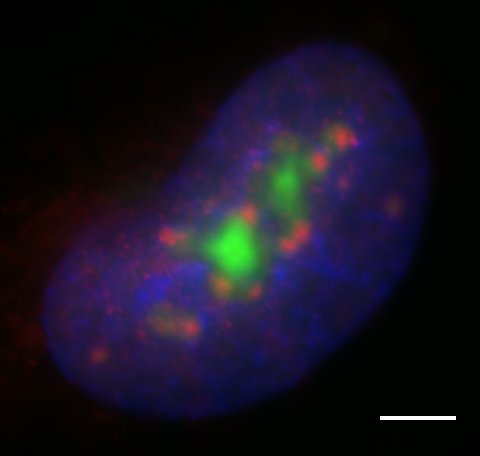

Supplement: Supplementary file 13 — Source data Fig. 5 [file 44318_2024_333_MOESM13_ESM.zip › Figure 5/Figure 5E/MG-siHUWE1/Composite.jpg]

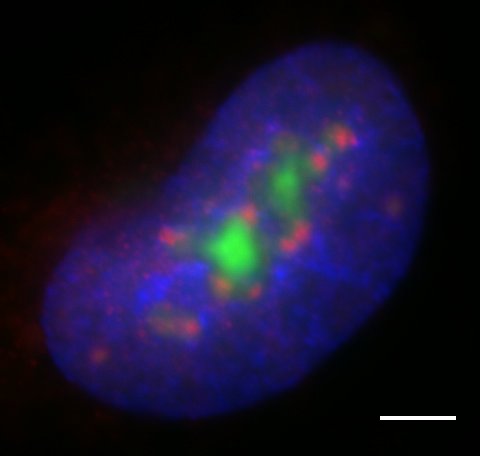

Supplement: Supplementary file 13 — Source data Fig. 5 [file 44318_2024_333_MOESM13_ESM.zip › Figure 5/Figure 5E/MG-siHUWE1/Composite1.jpg]

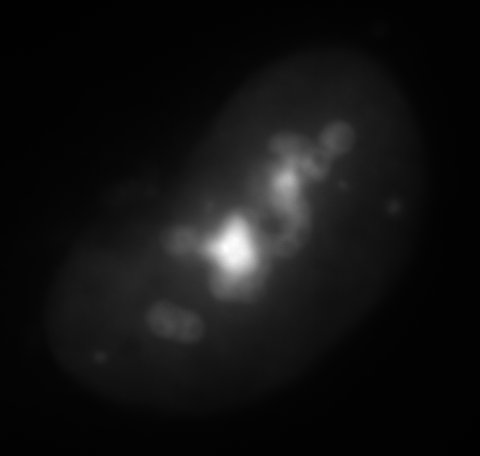

Supplement: Supplementary file 13 — Source data Fig. 5 [file 44318_2024_333_MOESM13_ESM.zip › Figure 5/Figure 5E/MG-siHUWE1/siHUWE1_240919_A_MG si_2_w1GFP.jpg]

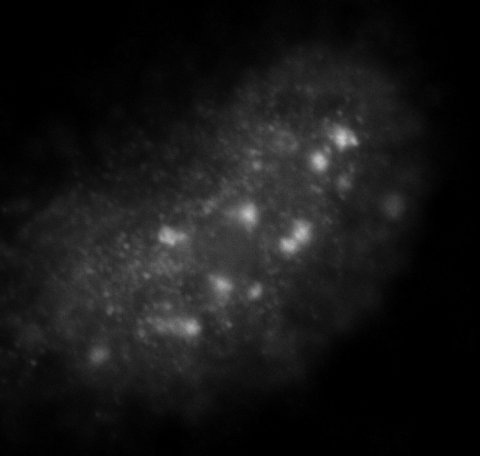

Supplement: Supplementary file 13 — Source data Fig. 5 [file 44318_2024_333_MOESM13_ESM.zip › Figure 5/Figure 5E/MG-siHUWE1/siHUWE1_240919_A_MG si_2_w2TexasRed.jpg]

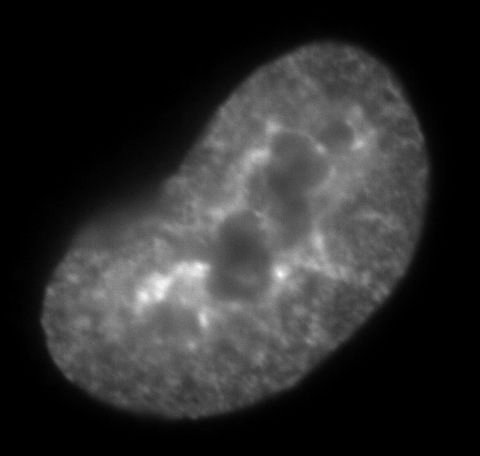

Supplement: Supplementary file 13 — Source data Fig. 5 [file 44318_2024_333_MOESM13_ESM.zip › Figure 5/Figure 5E/MG-siHUWE1/siHUWE1_240919_A_MG si_2_w3DAPI.jpg]

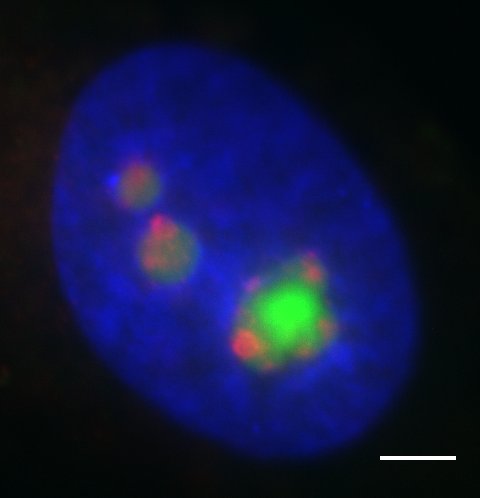

Supplement: Supplementary file 13 — Source data Fig. 5 [file 44318_2024_333_MOESM13_ESM.zip › Figure 5/Figure 5E/MG132/Composite2.jpg]

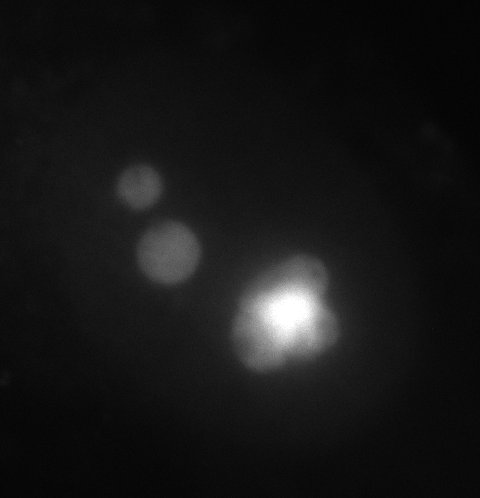

Supplement: Supplementary file 13 — Source data Fig. 5 [file 44318_2024_333_MOESM13_ESM.zip › Figure 5/Figure 5E/MG132/siHUWE1_240919_A_MG_37_w1GFP.jpg]

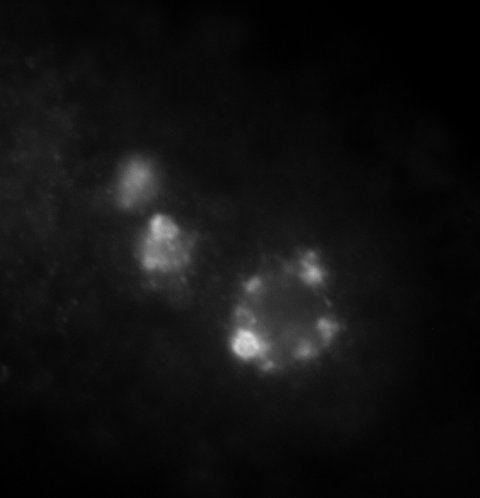

Supplement: Supplementary file 13 — Source data Fig. 5 [file 44318_2024_333_MOESM13_ESM.zip › Figure 5/Figure 5E/MG132/siHUWE1_240919_A_MG_37_w2TexasRed.jpg]

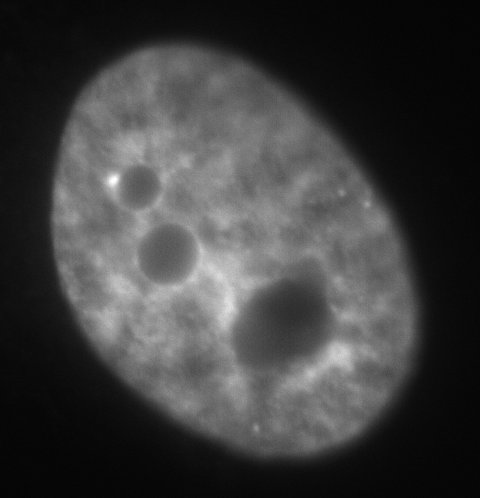

Supplement: Supplementary file 13 — Source data Fig. 5 [file 44318_2024_333_MOESM13_ESM.zip › Figure 5/Figure 5E/MG132/siHUWE1_240919_A_MG_37_w3DAPI.jpg]

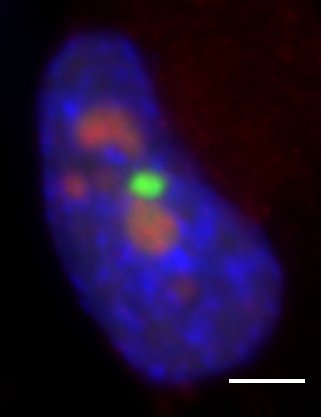

Supplement: Supplementary file 13 — Source data Fig. 5 [file 44318_2024_333_MOESM13_ESM.zip › Figure 5/Figure 5E/R8h siHUWE1/Composite.jpg]

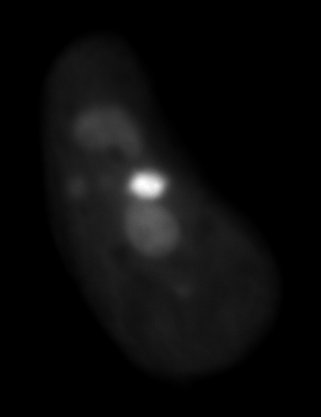

Supplement: Supplementary file 13 — Source data Fig. 5 [file 44318_2024_333_MOESM13_ESM.zip › Figure 5/Figure 5E/R8h siHUWE1/siHUWE1_240919_b_R8h si__w1GFP.jpg]
