# Supplementary figures and images for: A nuclear protein quality control system for elimination of nucleolus-related inclusions (part 3 of 4)
Source: EMBO J. 2024 Dec 17;44(3):801–23. doi: 10.1038/s44318-024-00333-9 (PMC11791210; doi:10.1038/s44318-024-00333-9)

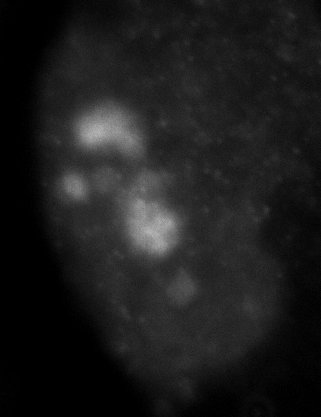

Supplement: Supplementary file 13 — Source data Fig. 5 [file 44318_2024_333_MOESM13_ESM.zip › Figure 5/Figure 5E/R8h siHUWE1/siHUWE1_240919_b_R8h si__w2TexasRed.jpg]

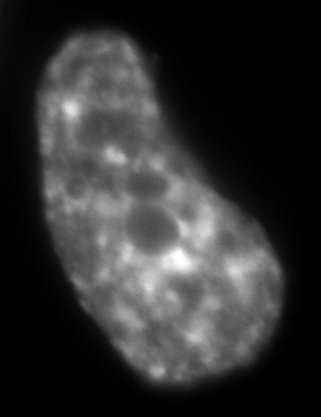

Supplement: Supplementary file 13 — Source data Fig. 5 [file 44318_2024_333_MOESM13_ESM.zip › Figure 5/Figure 5E/R8h siHUWE1/siHUWE1_240919_b_R8h si__w3DAPI.jpg]

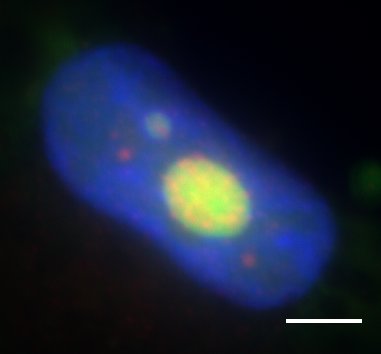

Supplement: Supplementary file 13 — Source data Fig. 5 [file 44318_2024_333_MOESM13_ESM.zip › Figure 5/Figure 5E/Recovery/Composite.jpg]

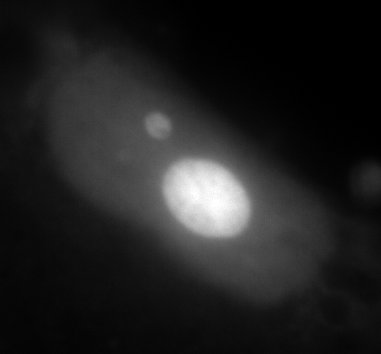

Supplement: Supplementary file 13 — Source data Fig. 5 [file 44318_2024_333_MOESM13_ESM.zip › Figure 5/Figure 5E/Recovery/siHUWE1_240919_A_R8h_2_w1GFP.jpg]

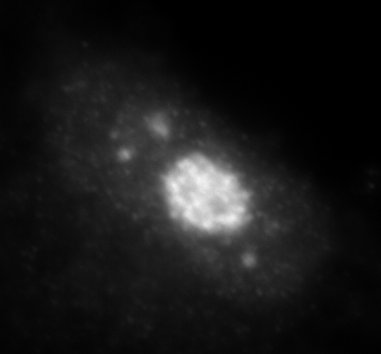

Supplement: Supplementary file 13 — Source data Fig. 5 [file 44318_2024_333_MOESM13_ESM.zip › Figure 5/Figure 5E/Recovery/siHUWE1_240919_A_R8h_2_w2TexasRed.jpg]

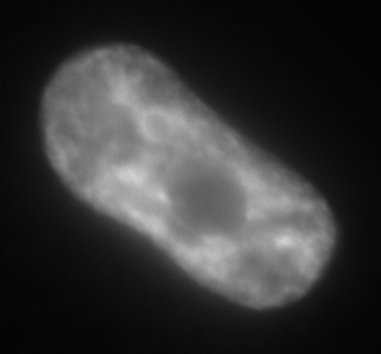

Supplement: Supplementary file 13 — Source data Fig. 5 [file 44318_2024_333_MOESM13_ESM.zip › Figure 5/Figure 5E/Recovery/siHUWE1_240919_A_R8h_2_w3DAPI.jpg]

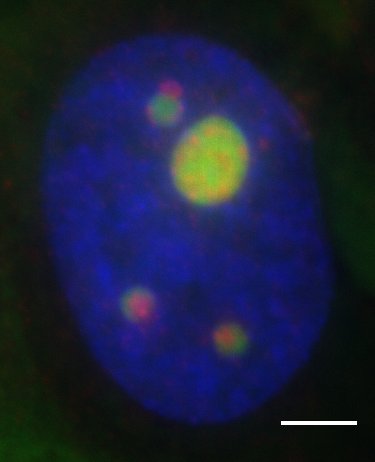

Supplement: Supplementary file 13 — Source data Fig. 5 [file 44318_2024_333_MOESM13_ESM.zip › Figure 5/Figure 5E/ctr/Composite1.jpg]

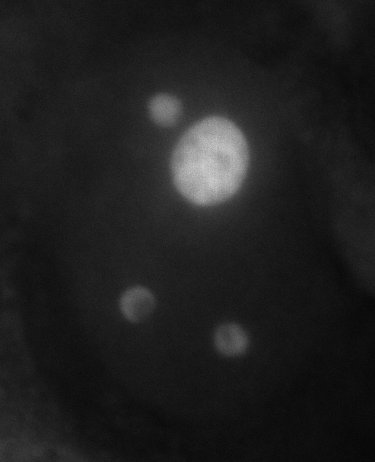

Supplement: Supplementary file 13 — Source data Fig. 5 [file 44318_2024_333_MOESM13_ESM.zip › Figure 5/Figure 5E/ctr/siHUWE1_240919_b_unt_12_w1GFP.jpg]

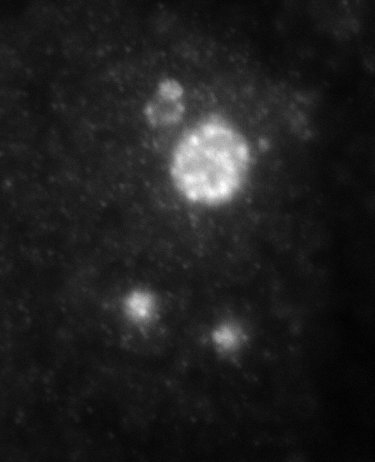

Supplement: Supplementary file 13 — Source data Fig. 5 [file 44318_2024_333_MOESM13_ESM.zip › Figure 5/Figure 5E/ctr/siHUWE1_240919_b_unt_12_w2TexasRed.jpg]

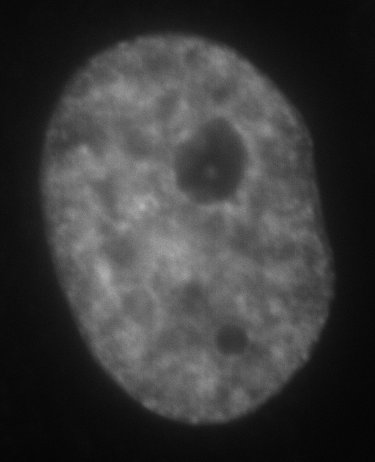

Supplement: Supplementary file 13 — Source data Fig. 5 [file 44318_2024_333_MOESM13_ESM.zip › Figure 5/Figure 5E/ctr/siHUWE1_240919_b_unt_12_w3DAPI.jpg]

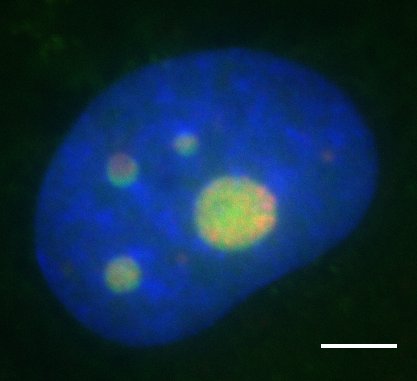

Supplement: Supplementary file 13 — Source data Fig. 5 [file 44318_2024_333_MOESM13_ESM.zip › Figure 5/Figure 5E/ctr - sihuwe1/Composite.jpg]

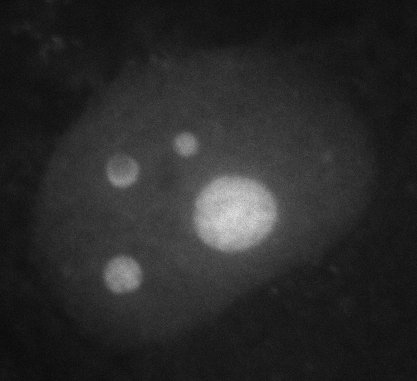

Supplement: Supplementary file 13 — Source data Fig. 5 [file 44318_2024_333_MOESM13_ESM.zip › Figure 5/Figure 5E/ctr - sihuwe1/siHUWE1_240919_b_si__w1GFP.jpg]

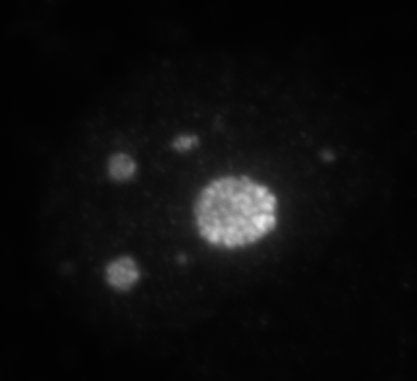

Supplement: Supplementary file 13 — Source data Fig. 5 [file 44318_2024_333_MOESM13_ESM.zip › Figure 5/Figure 5E/ctr - sihuwe1/siHUWE1_240919_b_si__w2TexasRed.jpg]

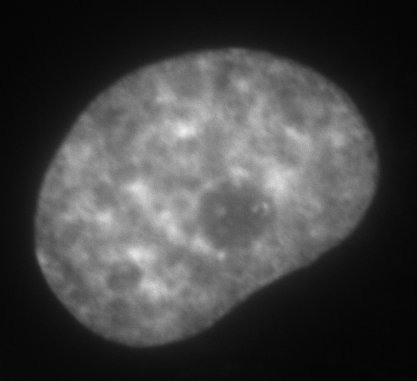

Supplement: Supplementary file 13 — Source data Fig. 5 [file 44318_2024_333_MOESM13_ESM.zip › Figure 5/Figure 5E/ctr - sihuwe1/siHUWE1_240919_b_si__w3DAPI.jpg]

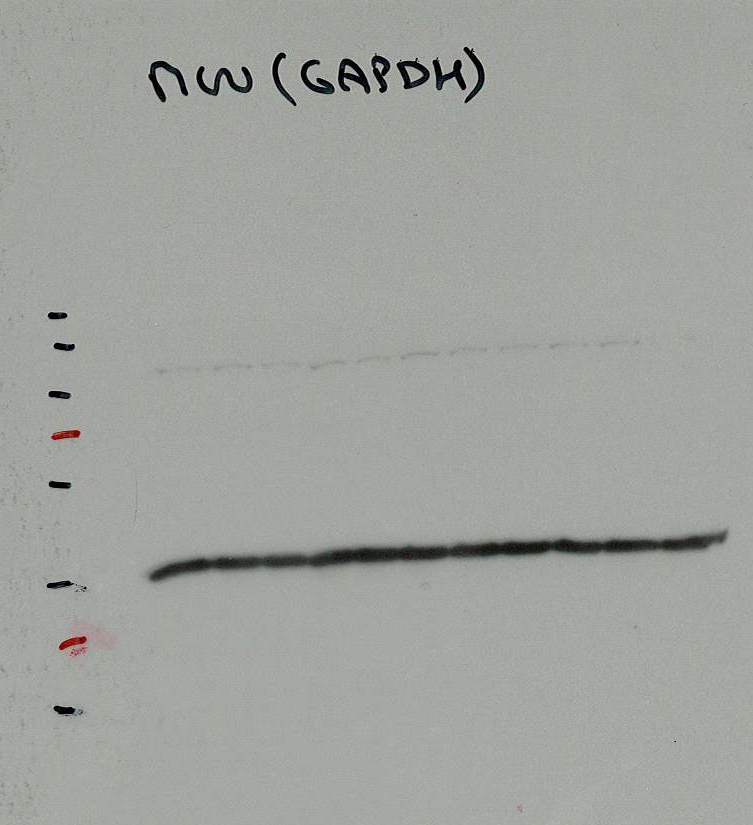

Supplement: Supplementary file 13 — Source data Fig. 5 [file 44318_2024_333_MOESM13_ESM.zip › Figure 5/Western blots_Fig 5B and 5F/Fig. 5B_GAPDH.jpg]

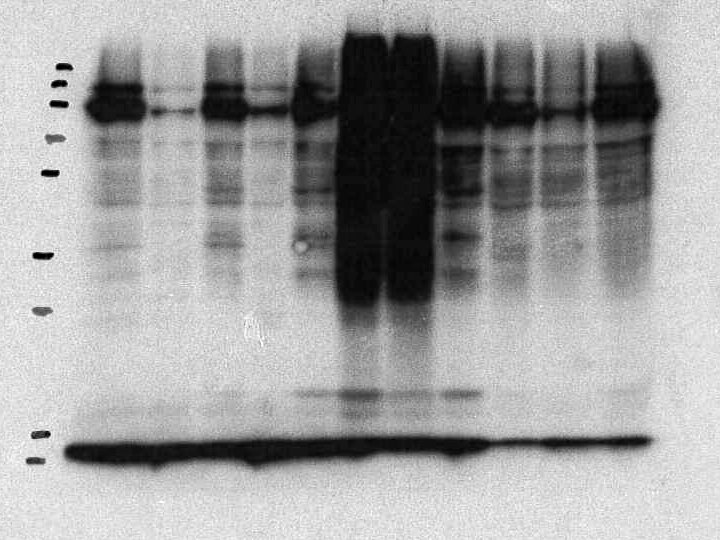

Supplement: Supplementary file 13 — Source data Fig. 5 [file 44318_2024_333_MOESM13_ESM.zip › Figure 5/Western blots_Fig 5B and 5F/Fig. 5B_NEDD8.jpg]

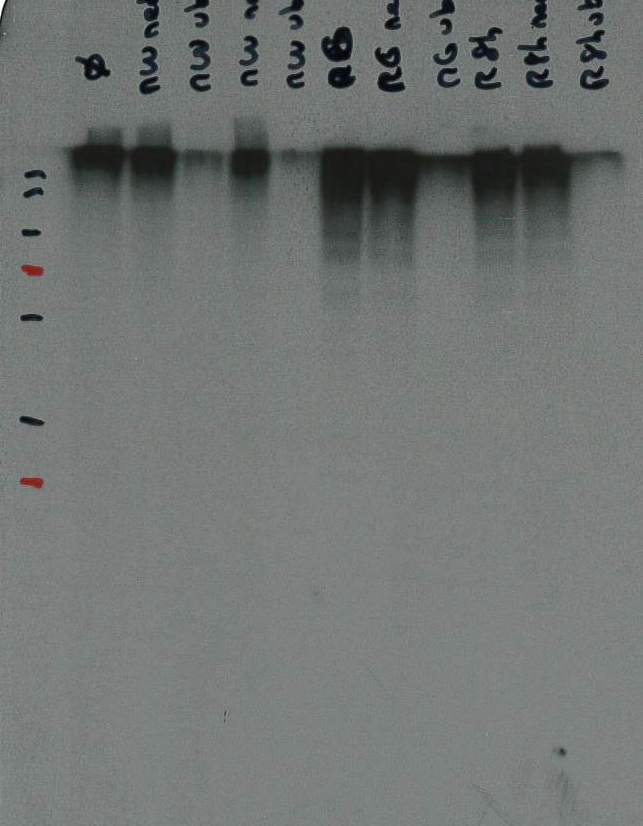

Supplement: Supplementary file 13 — Source data Fig. 5 [file 44318_2024_333_MOESM13_ESM.zip › Figure 5/Western blots_Fig 5B and 5F/Fig. 5B_ubiquitin.jpg]

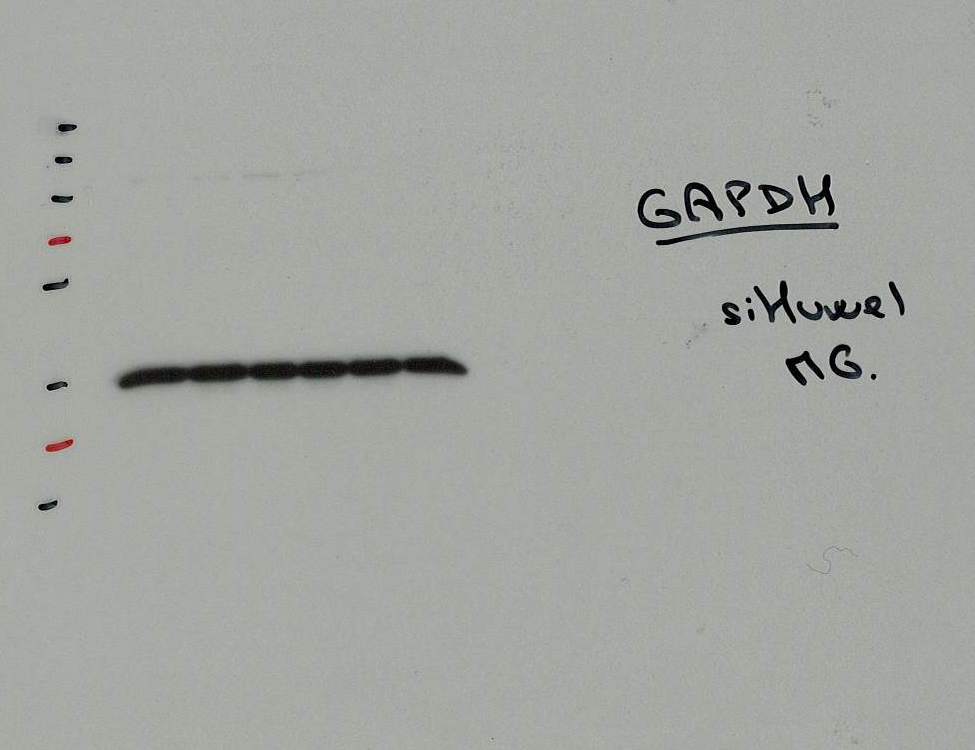

Supplement: Supplementary file 13 — Source data Fig. 5 [file 44318_2024_333_MOESM13_ESM.zip › Figure 5/Western blots_Fig 5B and 5F/Fig. 5F_GAPDH.jpg]

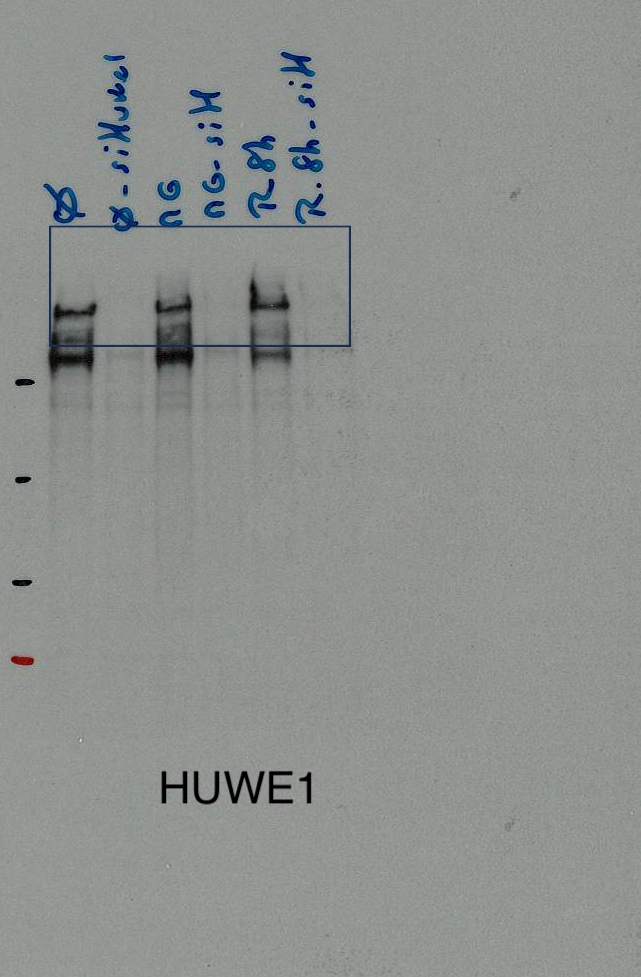

Supplement: Supplementary file 13 — Source data Fig. 5 [file 44318_2024_333_MOESM13_ESM.zip › Figure 5/Western blots_Fig 5B and 5F/Fig. 5F_HUWE1.jpg]

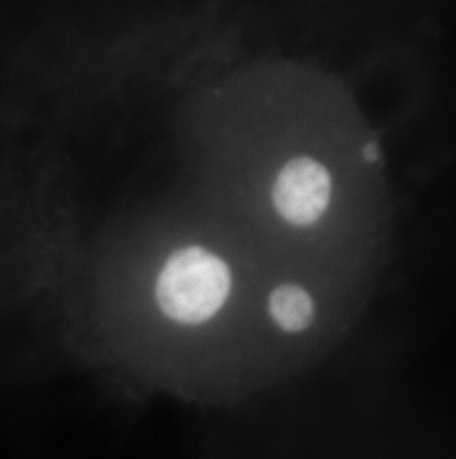

Supplement: Supplementary file 14 — Source data Fig. 6 [file 44318_2024_333_MOESM14_ESM.zip › Figure 6/Figure 6A/ActD 1h/050221_L11 GFP_Fibrillarin_ActD 1h_2_w1GFP.jpg]

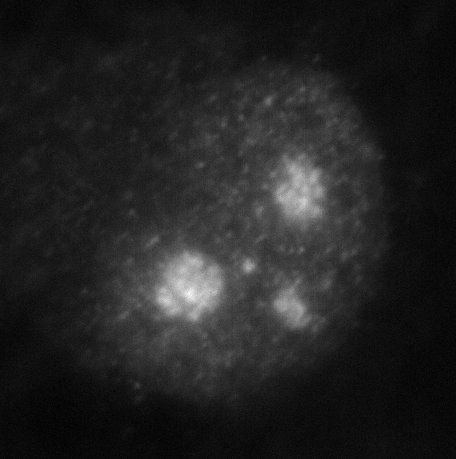

Supplement: Supplementary file 14 — Source data Fig. 6 [file 44318_2024_333_MOESM14_ESM.zip › Figure 6/Figure 6A/ActD 1h/050221_L11 GFP_Fibrillarin_ActD 1h_2_w2Texas Red.jpg]

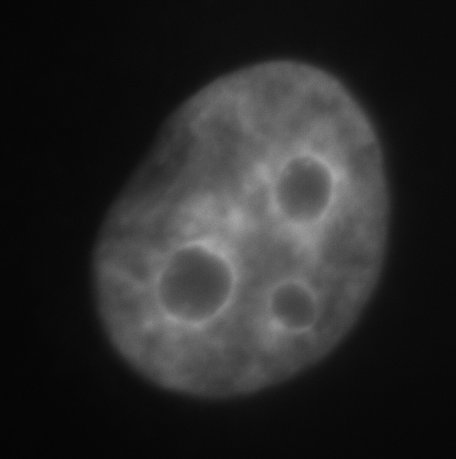

Supplement: Supplementary file 14 — Source data Fig. 6 [file 44318_2024_333_MOESM14_ESM.zip › Figure 6/Figure 6A/ActD 1h/050221_L11 GFP_Fibrillarin_ActD 1h_2_w3Hoechst.jpg]

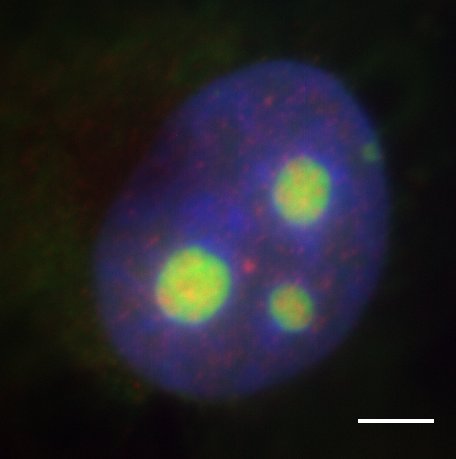

Supplement: Supplementary file 14 — Source data Fig. 6 [file 44318_2024_333_MOESM14_ESM.zip › Figure 6/Figure 6A/ActD 1h/Composite.jpg]

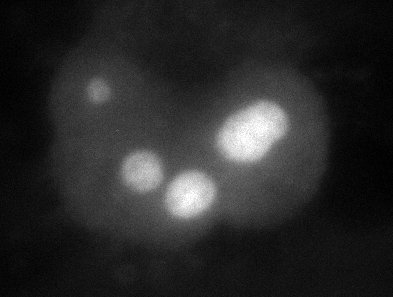

Supplement: Supplementary file 14 — Source data Fig. 6 [file 44318_2024_333_MOESM14_ESM.zip › Figure 6/Figure 6A/ActD 1h then on/220121_Fibrillarin_ActD 1h then on_4_w1GFP.jpg]

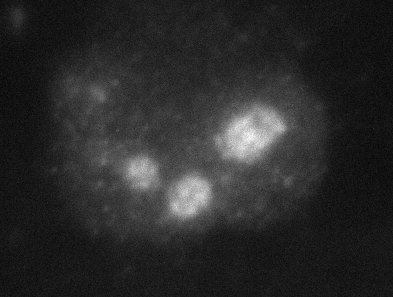

Supplement: Supplementary file 14 — Source data Fig. 6 [file 44318_2024_333_MOESM14_ESM.zip › Figure 6/Figure 6A/ActD 1h then on/220121_Fibrillarin_ActD 1h then on_4_w2Texas Red.jpg]

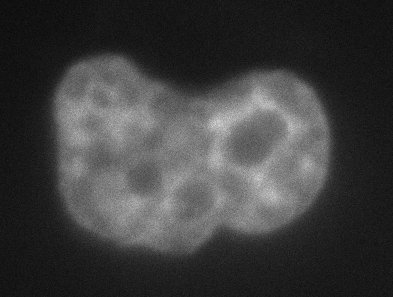

Supplement: Supplementary file 14 — Source data Fig. 6 [file 44318_2024_333_MOESM14_ESM.zip › Figure 6/Figure 6A/ActD 1h then on/220121_Fibrillarin_ActD 1h then on_4_w3Hoechst.jpg]

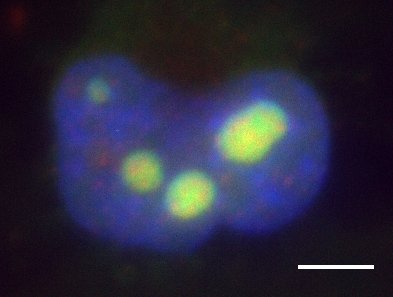

Supplement: Supplementary file 14 — Source data Fig. 6 [file 44318_2024_333_MOESM14_ESM.zip › Figure 6/Figure 6A/ActD 1h then on/Composite1.jpg]

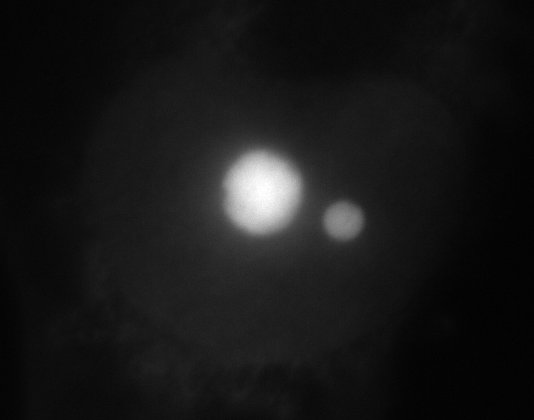

Supplement: Supplementary file 14 — Source data Fig. 6 [file 44318_2024_333_MOESM14_ESM.zip › Figure 6/Figure 6A/ActD 2h/050221_L11 GFP_Fibrillarin_ActD 2h_2_w1GFP.jpg]

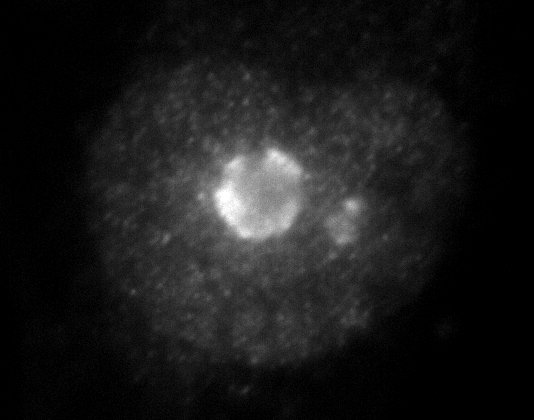

Supplement: Supplementary file 14 — Source data Fig. 6 [file 44318_2024_333_MOESM14_ESM.zip › Figure 6/Figure 6A/ActD 2h/050221_L11 GFP_Fibrillarin_ActD 2h_2_w2Texas Red.jpg]

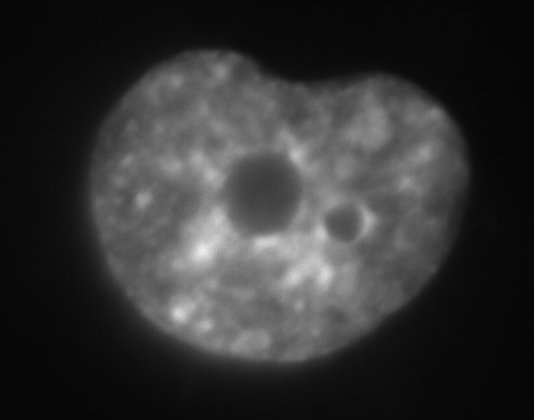

Supplement: Supplementary file 14 — Source data Fig. 6 [file 44318_2024_333_MOESM14_ESM.zip › Figure 6/Figure 6A/ActD 2h/050221_L11 GFP_Fibrillarin_ActD 2h_2_w3Hoechst.jpg]

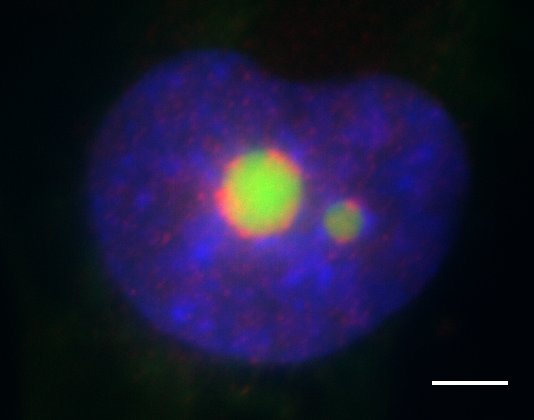

Supplement: Supplementary file 14 — Source data Fig. 6 [file 44318_2024_333_MOESM14_ESM.zip › Figure 6/Figure 6A/ActD 2h/Composite.jpg]

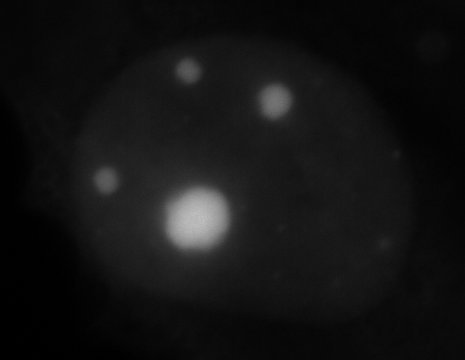

Supplement: Supplementary file 14 — Source data Fig. 6 [file 44318_2024_333_MOESM14_ESM.zip › Figure 6/Figure 6A/ActD 4h/050221_L11 GFP_Fibrillarin_ActD 4h_1_w1GFP.jpg]

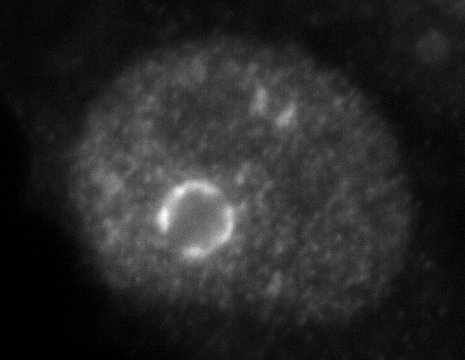

Supplement: Supplementary file 14 — Source data Fig. 6 [file 44318_2024_333_MOESM14_ESM.zip › Figure 6/Figure 6A/ActD 4h/050221_L11 GFP_Fibrillarin_ActD 4h_1_w2Texas Red.jpg]

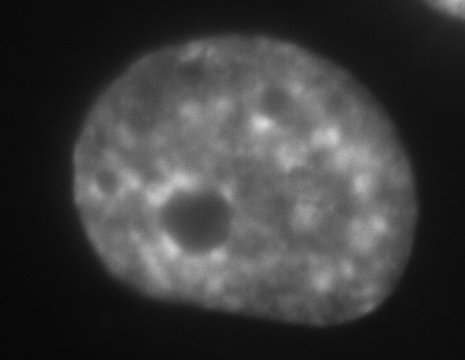

Supplement: Supplementary file 14 — Source data Fig. 6 [file 44318_2024_333_MOESM14_ESM.zip › Figure 6/Figure 6A/ActD 4h/050221_L11 GFP_Fibrillarin_ActD 4h_1_w3Hoechst.jpg]

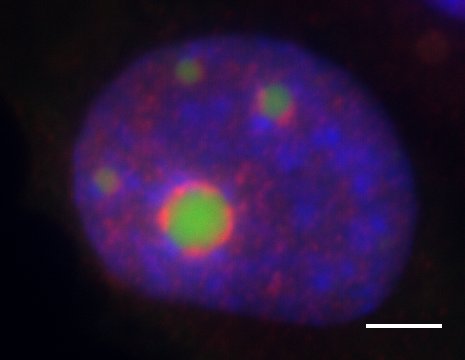

Supplement: Supplementary file 14 — Source data Fig. 6 [file 44318_2024_333_MOESM14_ESM.zip › Figure 6/Figure 6A/ActD 4h/Composite.jpg]

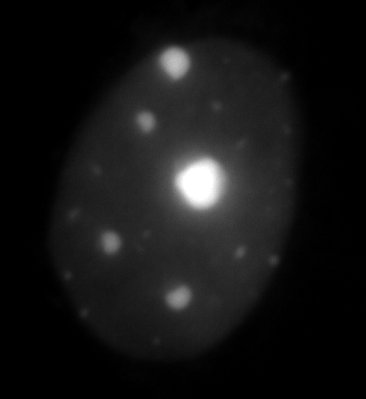

Supplement: Supplementary file 14 — Source data Fig. 6 [file 44318_2024_333_MOESM14_ESM.zip › Figure 6/Figure 6A/ActD 8h/050221_L11 GFP_Fibrillarin_ActD 8h_18_w1GFP.jpg]

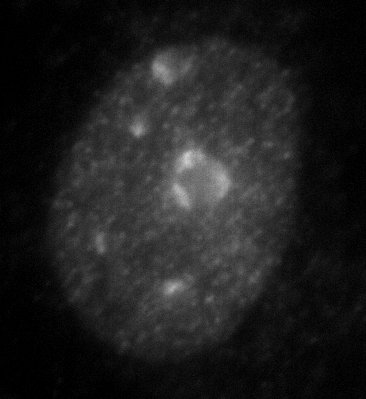

Supplement: Supplementary file 14 — Source data Fig. 6 [file 44318_2024_333_MOESM14_ESM.zip › Figure 6/Figure 6A/ActD 8h/050221_L11 GFP_Fibrillarin_ActD 8h_18_w2Texas Red.jpg]

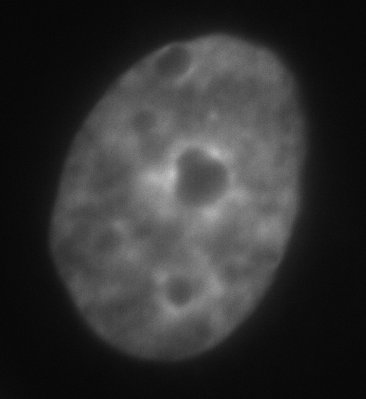

Supplement: Supplementary file 14 — Source data Fig. 6 [file 44318_2024_333_MOESM14_ESM.zip › Figure 6/Figure 6A/ActD 8h/050221_L11 GFP_Fibrillarin_ActD 8h_18_w3Hoechst.jpg]

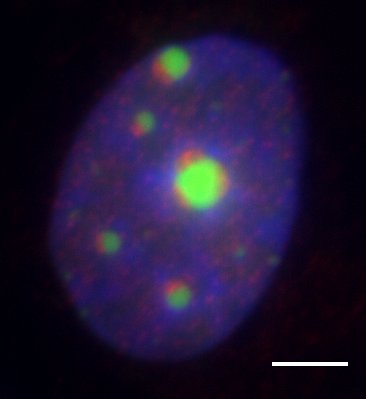

Supplement: Supplementary file 14 — Source data Fig. 6 [file 44318_2024_333_MOESM14_ESM.zip › Figure 6/Figure 6A/ActD 8h/Composite.jpg]

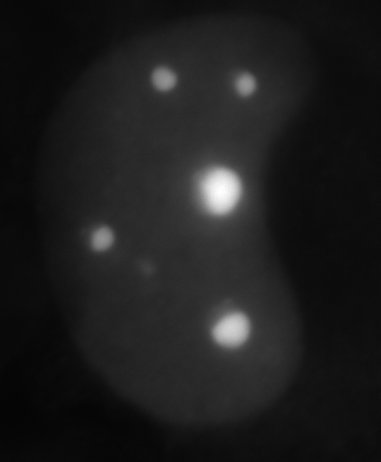

Supplement: Supplementary file 14 — Source data Fig. 6 [file 44318_2024_333_MOESM14_ESM.zip › Figure 6/Figure 6A/ActD on/050221_L11 GFP_Fibrillarin_ActD on_9_w1GFP.jpg]

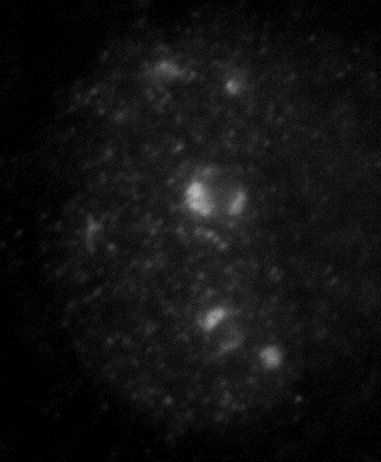

Supplement: Supplementary file 14 — Source data Fig. 6 [file 44318_2024_333_MOESM14_ESM.zip › Figure 6/Figure 6A/ActD on/050221_L11 GFP_Fibrillarin_ActD on_9_w2Texas Red.jpg]

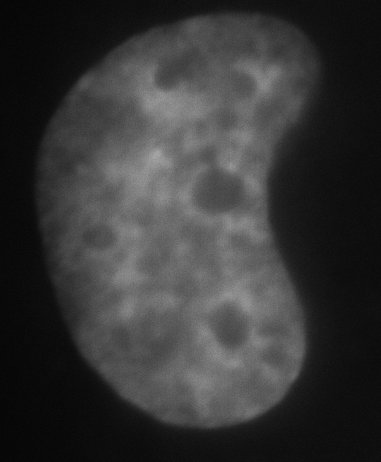

Supplement: Supplementary file 14 — Source data Fig. 6 [file 44318_2024_333_MOESM14_ESM.zip › Figure 6/Figure 6A/ActD on/050221_L11 GFP_Fibrillarin_ActD on_9_w3Hoechst.jpg]

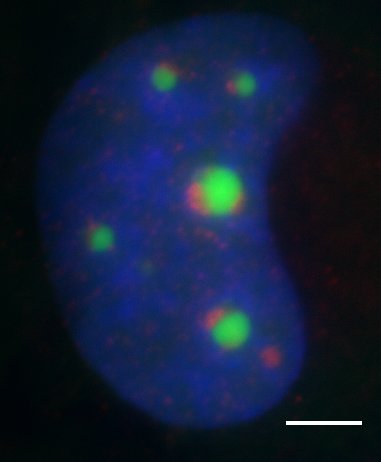

Supplement: Supplementary file 14 — Source data Fig. 6 [file 44318_2024_333_MOESM14_ESM.zip › Figure 6/Figure 6A/ActD on/Composite.jpg]

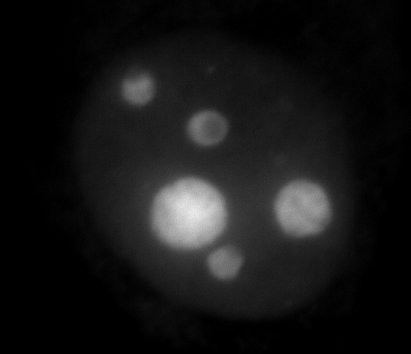

Supplement: Supplementary file 14 — Source data Fig. 6 [file 44318_2024_333_MOESM14_ESM.zip › Figure 6/Figure 6A/CTR/050221_L11 GFP_Fibrillarin_unt_12_w1GFP.jpg]

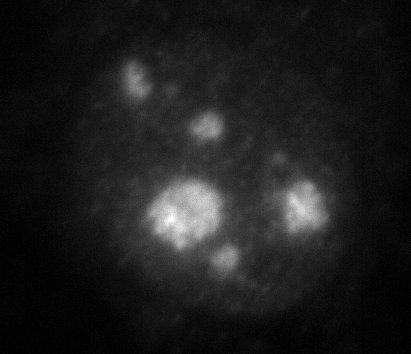

Supplement: Supplementary file 14 — Source data Fig. 6 [file 44318_2024_333_MOESM14_ESM.zip › Figure 6/Figure 6A/CTR/050221_L11 GFP_Fibrillarin_unt_12_w2Texas Red.jpg]

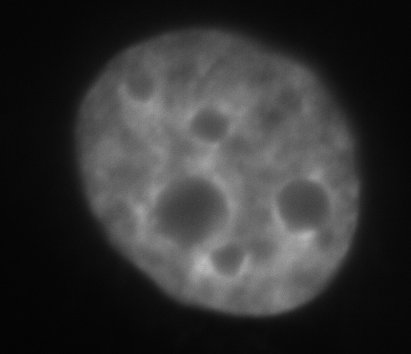

Supplement: Supplementary file 14 — Source data Fig. 6 [file 44318_2024_333_MOESM14_ESM.zip › Figure 6/Figure 6A/CTR/050221_L11 GFP_Fibrillarin_unt_12_w3Hoechst.jpg]

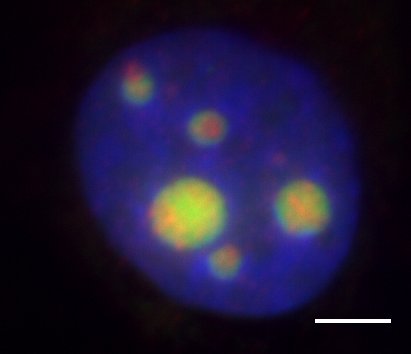

Supplement: Supplementary file 14 — Source data Fig. 6 [file 44318_2024_333_MOESM14_ESM.zip › Figure 6/Figure 6A/CTR/Composite.jpg]

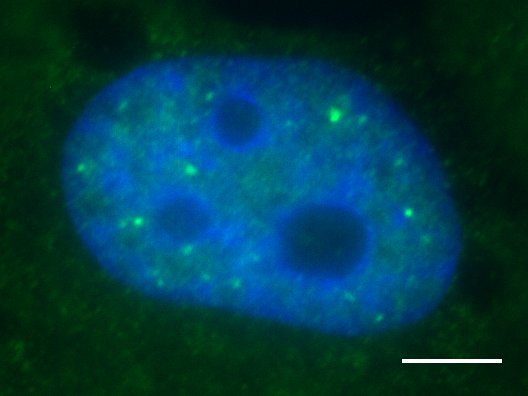

Supplement: Supplementary file 14 — Source data Fig. 6 [file 44318_2024_333_MOESM14_ESM.zip › Figure 6/Figure 6C/ActD 1h/actd 1h.jpg]

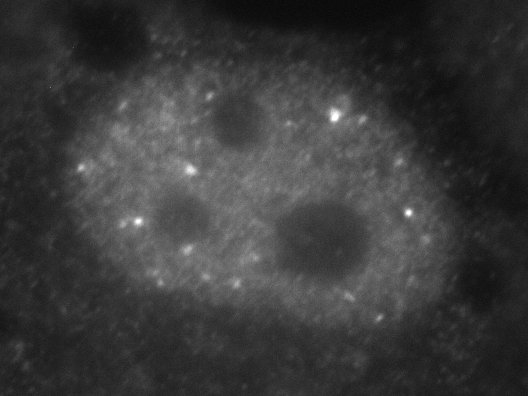

Supplement: Supplementary file 14 — Source data Fig. 6 [file 44318_2024_333_MOESM14_ESM.zip › Figure 6/Figure 6C/ActD 1h/ActD 1h__w1GFP.jpg]

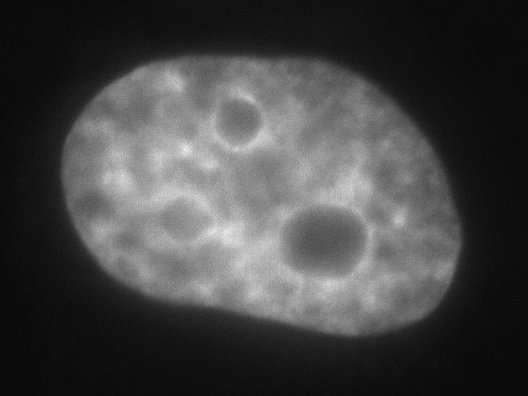

Supplement: Supplementary file 14 — Source data Fig. 6 [file 44318_2024_333_MOESM14_ESM.zip › Figure 6/Figure 6C/ActD 1h/ActD 1h__w2Hoechst.jpg]

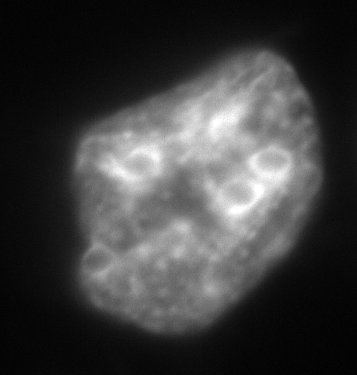

Supplement: Supplementary file 14 — Source data Fig. 6 [file 44318_2024_333_MOESM14_ESM.zip › Figure 6/Figure 6C/ActD R/121120_ub_ActD before MG_R8h actd_9_w1Hoechst.jpg]

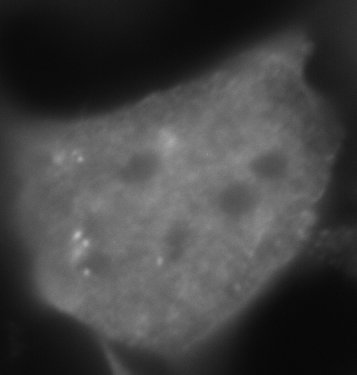

Supplement: Supplementary file 14 — Source data Fig. 6 [file 44318_2024_333_MOESM14_ESM.zip › Figure 6/Figure 6C/ActD R/121120_ub_ActD before MG_R8h actd_9_w2GFP.jpg]

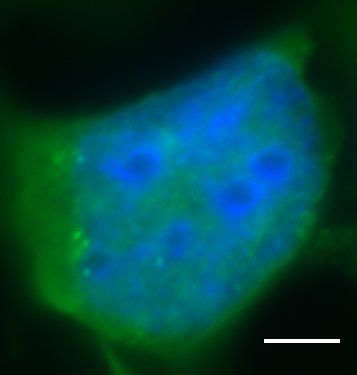

Supplement: Supplementary file 14 — Source data Fig. 6 [file 44318_2024_333_MOESM14_ESM.zip › Figure 6/Figure 6C/ActD R/Composite.jpg]

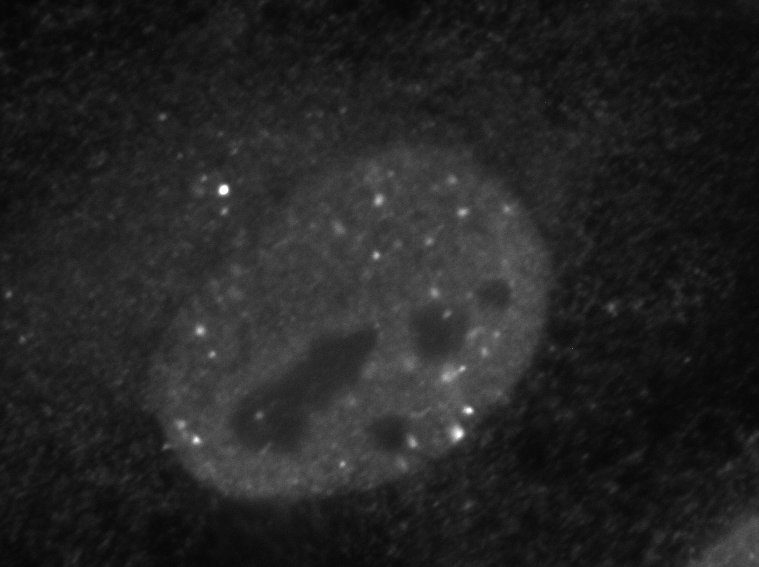

Supplement: Supplementary file 14 — Source data Fig. 6 [file 44318_2024_333_MOESM14_ESM.zip › Figure 6/Figure 6C/CTR/30.05.20_ActD_ubiquitin_unt_4_w1GFP.jpg]

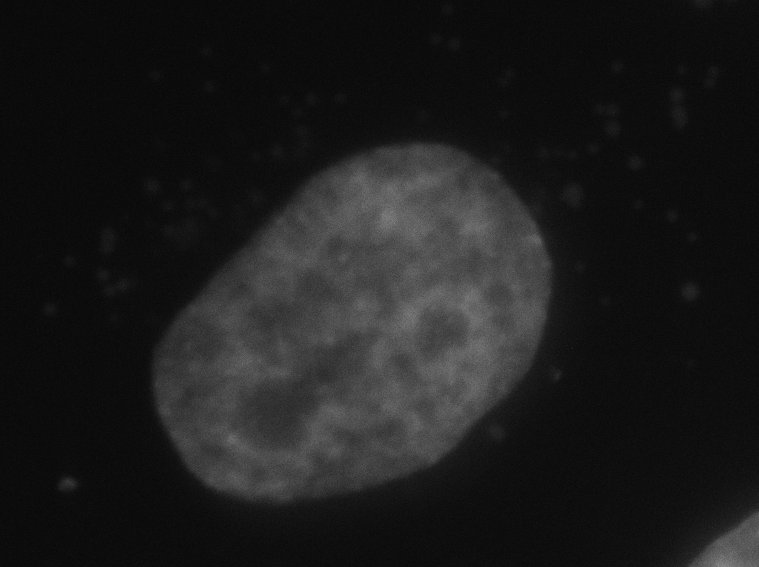

Supplement: Supplementary file 14 — Source data Fig. 6 [file 44318_2024_333_MOESM14_ESM.zip › Figure 6/Figure 6C/CTR/30.05.20_ActD_ubiquitin_unt_4_w2Hoechst.jpg]

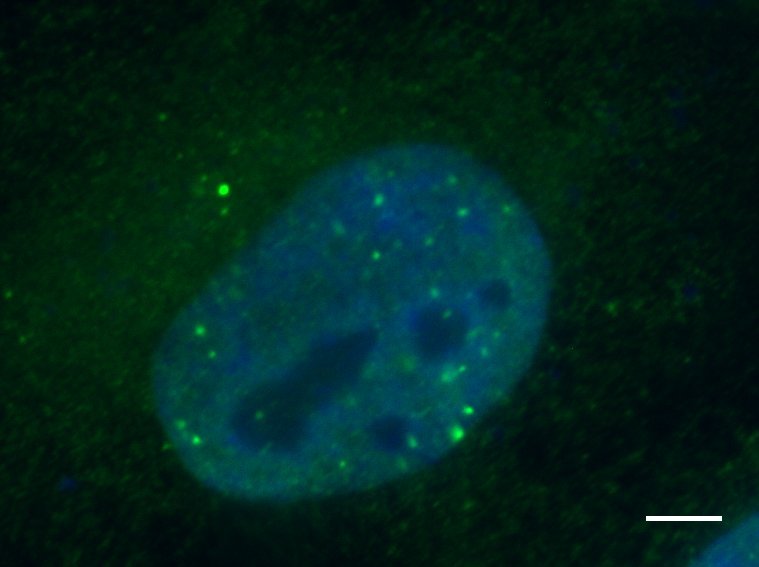

Supplement: Supplementary file 14 — Source data Fig. 6 [file 44318_2024_333_MOESM14_ESM.zip › Figure 6/Figure 6C/CTR/Composite_UNT bar.jpg]

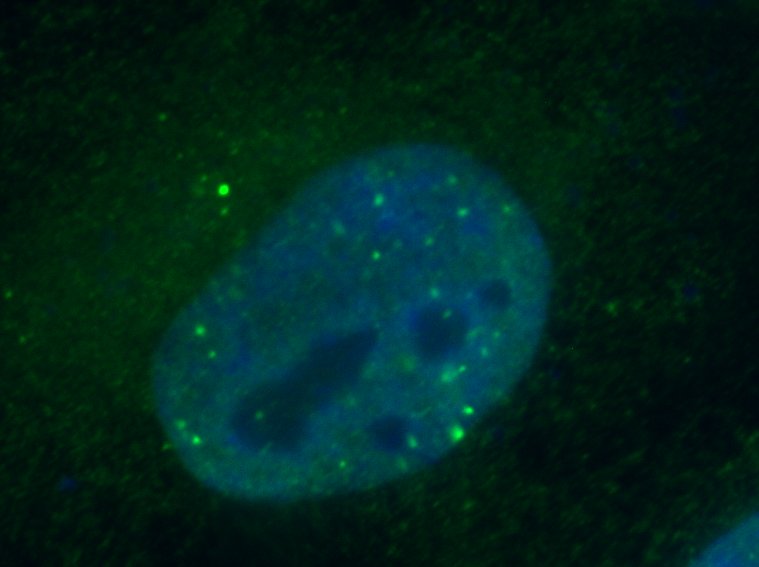

Supplement: Supplementary file 14 — Source data Fig. 6 [file 44318_2024_333_MOESM14_ESM.zip › Figure 6/Figure 6C/CTR/Composite_UNT.jpg]

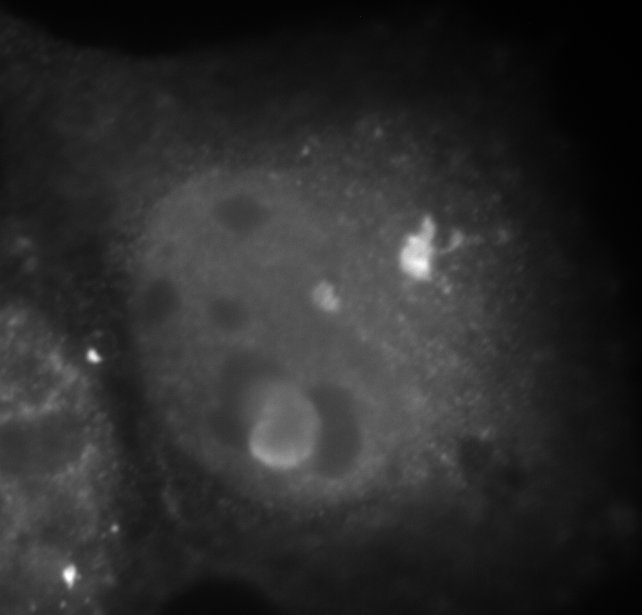

Supplement: Supplementary file 14 — Source data Fig. 6 [file 44318_2024_333_MOESM14_ESM.zip › Figure 6/Figure 6C/MG132/30.05.20_ActD_ubiquitin_MG132_17_w1GFP.jpg]

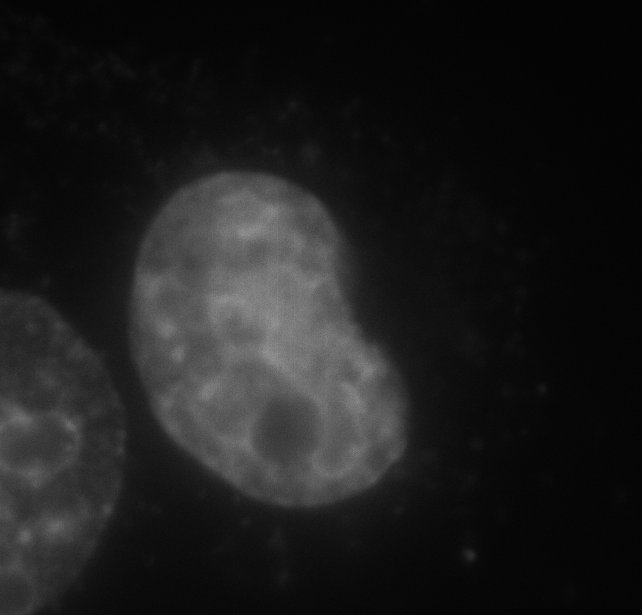

Supplement: Supplementary file 14 — Source data Fig. 6 [file 44318_2024_333_MOESM14_ESM.zip › Figure 6/Figure 6C/MG132/30.05.20_ActD_ubiquitin_MG132_17_w2Hoechst.jpg]

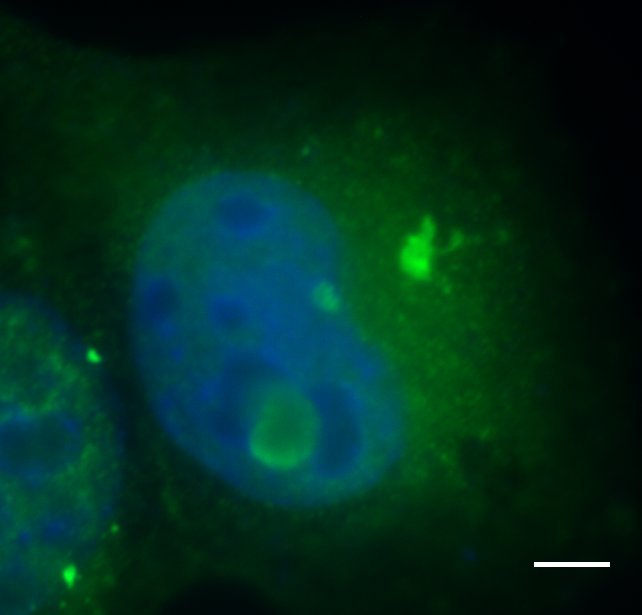

Supplement: Supplementary file 14 — Source data Fig. 6 [file 44318_2024_333_MOESM14_ESM.zip › Figure 6/Figure 6C/MG132/Composite_MG132 bar.jpg]

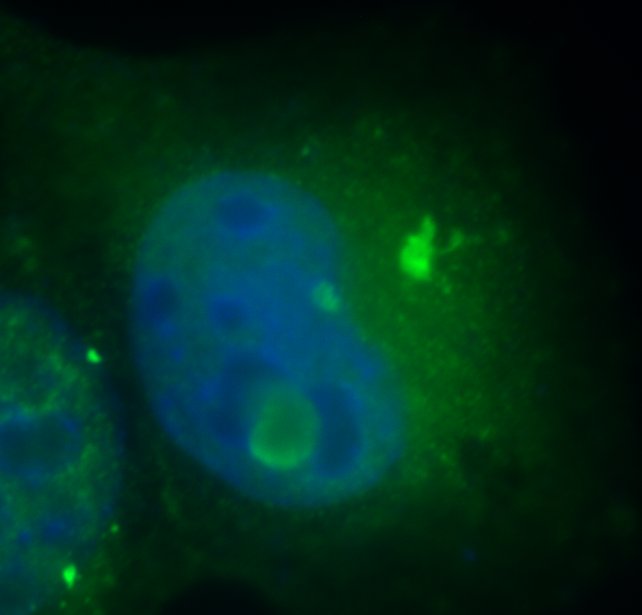

Supplement: Supplementary file 14 — Source data Fig. 6 [file 44318_2024_333_MOESM14_ESM.zip › Figure 6/Figure 6C/MG132/Composite_MG132.jpg]

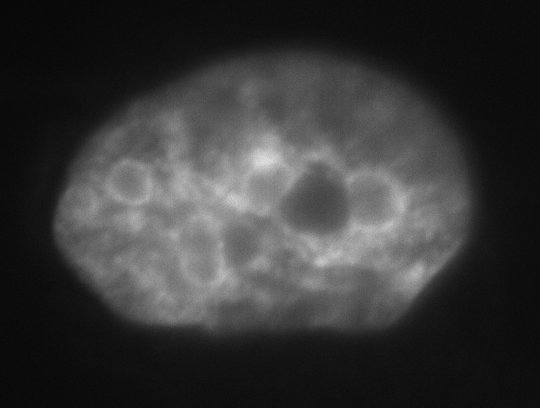

Supplement: Supplementary file 14 — Source data Fig. 6 [file 44318_2024_333_MOESM14_ESM.zip › Figure 6/Figure 6C/MG132_ActD 1h before/121120_ub_ActD before MG_MG actd1h before_12_w1Hoechst.jpg]

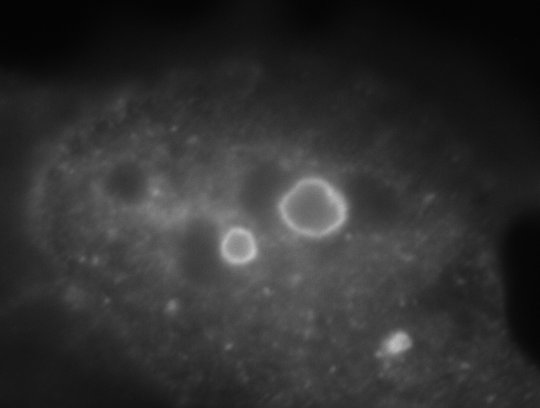

Supplement: Supplementary file 14 — Source data Fig. 6 [file 44318_2024_333_MOESM14_ESM.zip › Figure 6/Figure 6C/MG132_ActD 1h before/121120_ub_ActD before MG_MG actd1h before_12_w2GFP.jpg]

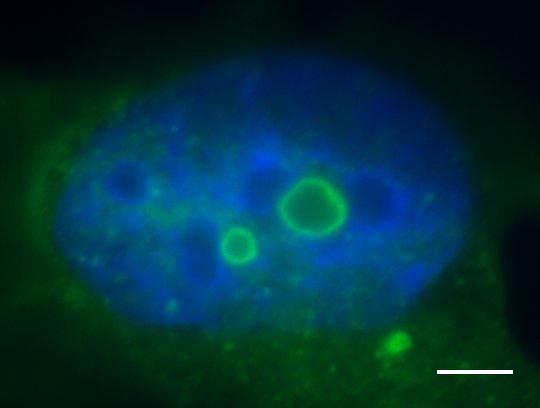

Supplement: Supplementary file 14 — Source data Fig. 6 [file 44318_2024_333_MOESM14_ESM.zip › Figure 6/Figure 6C/MG132_ActD 1h before/Composite.jpg]

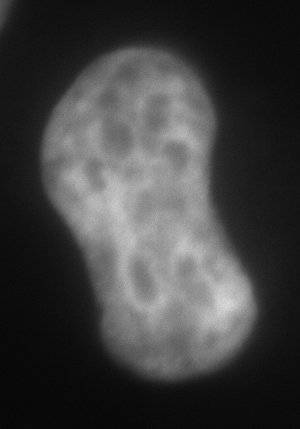

Supplement: Supplementary file 14 — Source data Fig. 6 [file 44318_2024_333_MOESM14_ESM.zip › Figure 6/Figure 6C/R8h/121120_ub_ActD before MG_R8h_4_w1Hoechst.jpg]

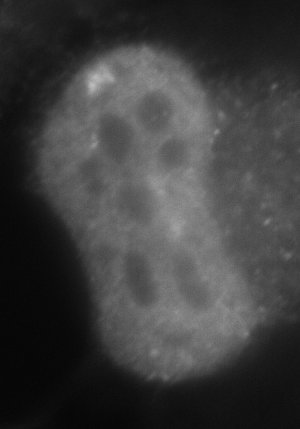

Supplement: Supplementary file 14 — Source data Fig. 6 [file 44318_2024_333_MOESM14_ESM.zip › Figure 6/Figure 6C/R8h/121120_ub_ActD before MG_R8h_4_w2GFP.jpg]

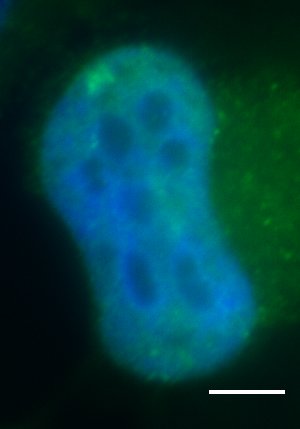

Supplement: Supplementary file 14 — Source data Fig. 6 [file 44318_2024_333_MOESM14_ESM.zip › Figure 6/Figure 6C/R8h/Composite.jpg]

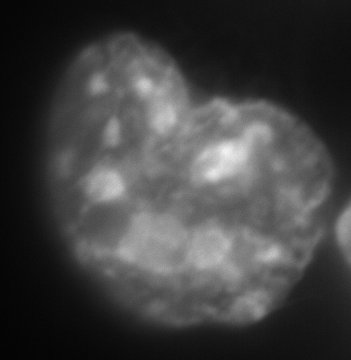

Supplement: Supplementary file 14 — Source data Fig. 6 [file 44318_2024_333_MOESM14_ESM.zip › Figure 6/Figure 6C/R8h ActD 1h before/121120_ub_ActD before MG_R8h actd 1h before mg__w1Hoechst.jpg]

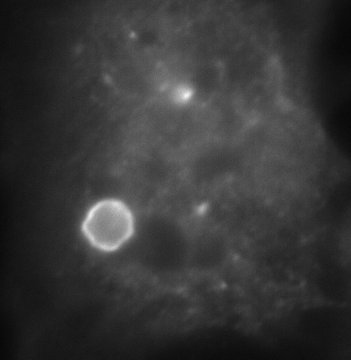

Supplement: Supplementary file 14 — Source data Fig. 6 [file 44318_2024_333_MOESM14_ESM.zip › Figure 6/Figure 6C/R8h ActD 1h before/121120_ub_ActD before MG_R8h actd 1h before mg__w2GFP.jpg]

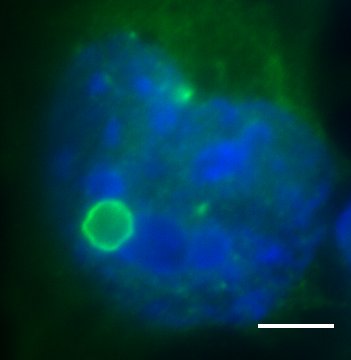

Supplement: Supplementary file 14 — Source data Fig. 6 [file 44318_2024_333_MOESM14_ESM.zip › Figure 6/Figure 6C/R8h ActD 1h before/Composite.jpg]

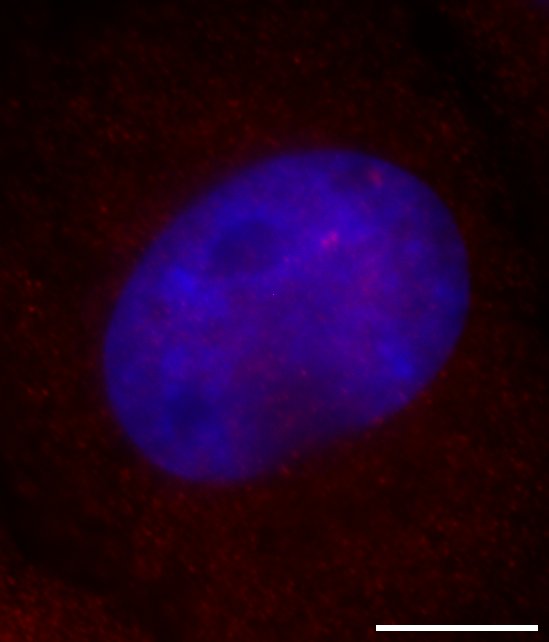

Supplement: Supplementary file 16 — Source data Fig. 8 [file 44318_2024_333_MOESM16_ESM.zip › Figure 8/Figure 8A/CTR siRNA/CTR/Composite.jpg]

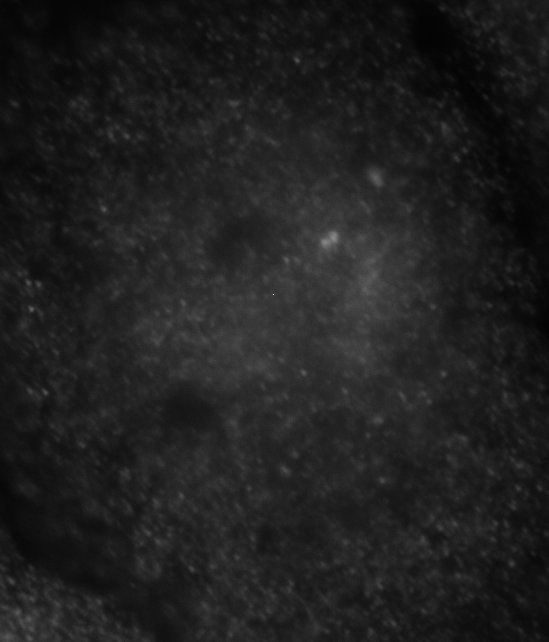

Supplement: Supplementary file 16 — Source data Fig. 8 [file 44318_2024_333_MOESM16_ESM.zip › Figure 8/Figure 8A/CTR siRNA/CTR/series1_control_untreated_2_w1AF-546.jpg]

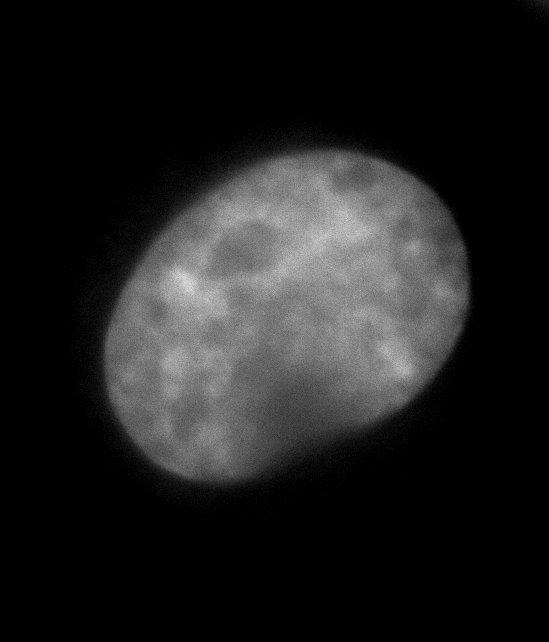

Supplement: Supplementary file 16 — Source data Fig. 8 [file 44318_2024_333_MOESM16_ESM.zip › Figure 8/Figure 8A/CTR siRNA/CTR/series1_control_untreated_2_w3DAPI.jpg]

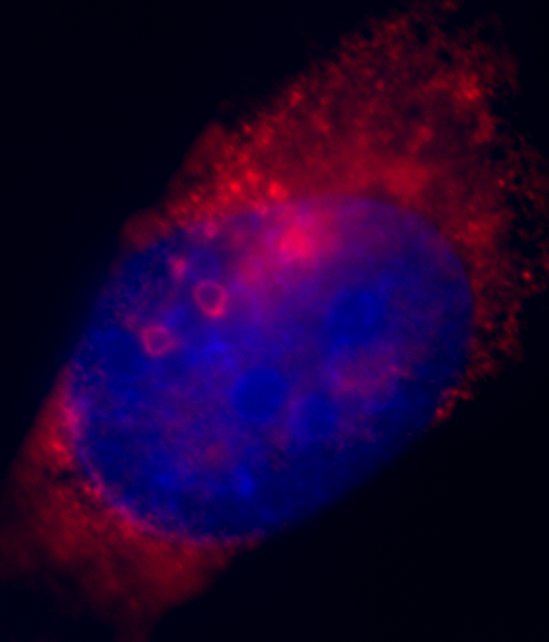

Supplement: Supplementary file 16 — Source data Fig. 8 [file 44318_2024_333_MOESM16_ESM.zip › Figure 8/Figure 8A/CTR siRNA/MG132/Composite.jpg]

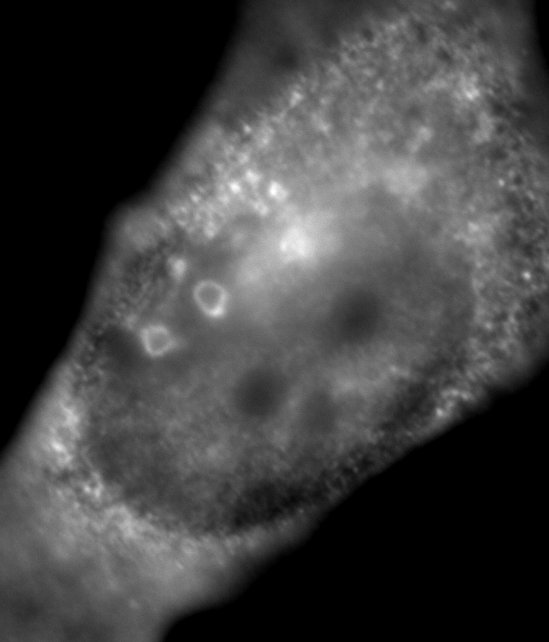

Supplement: Supplementary file 16 — Source data Fig. 8 [file 44318_2024_333_MOESM16_ESM.zip › Figure 8/Figure 8A/CTR siRNA/MG132/series1_control_MG_ON__w1AF-546.jpg]

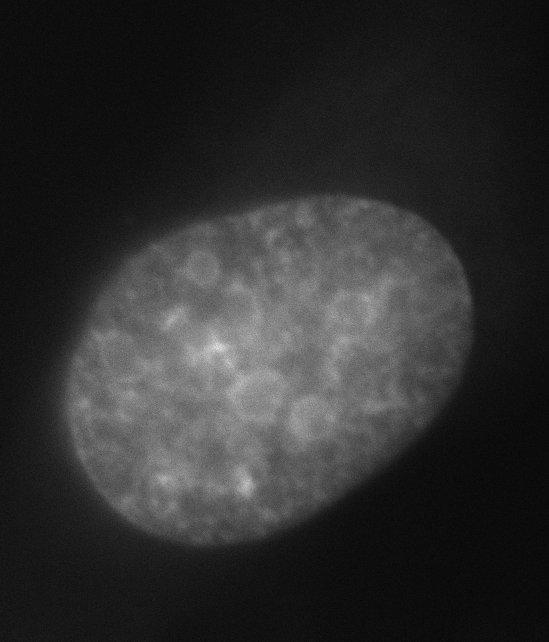

Supplement: Supplementary file 16 — Source data Fig. 8 [file 44318_2024_333_MOESM16_ESM.zip › Figure 8/Figure 8A/CTR siRNA/MG132/series1_control_MG_ON__w3DAPI.jpg]

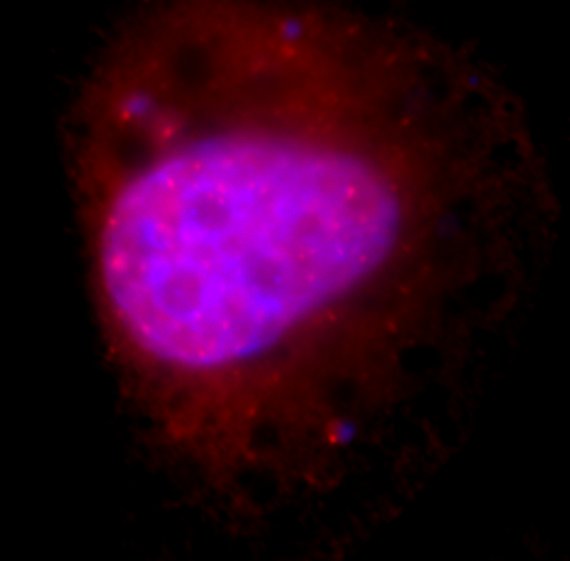

Supplement: Supplementary file 16 — Source data Fig. 8 [file 44318_2024_333_MOESM16_ESM.zip › Figure 8/Figure 8A/CTR siRNA/R.8h/Composite.jpg]

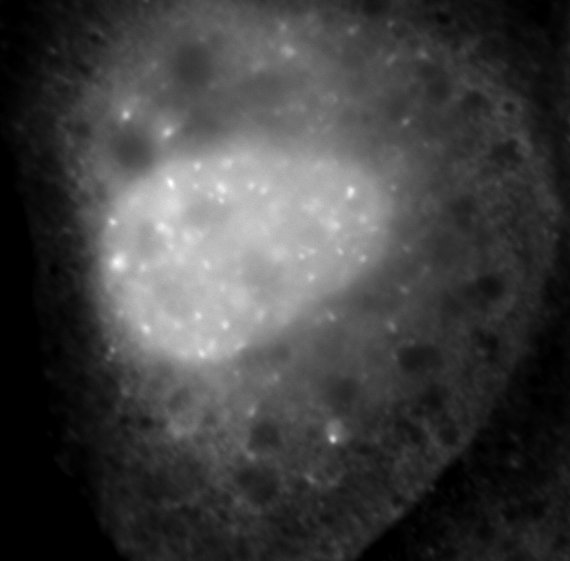

Supplement: Supplementary file 16 — Source data Fig. 8 [file 44318_2024_333_MOESM16_ESM.zip › Figure 8/Figure 8A/CTR siRNA/R.8h/series1_control_recovery__w1AF-546.jpg]

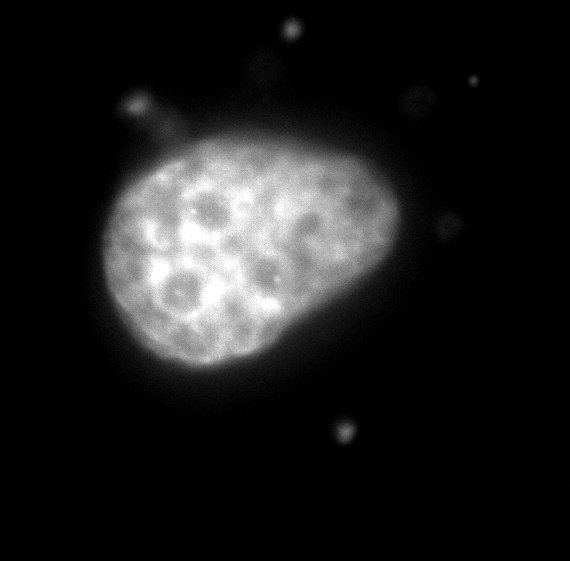

Supplement: Supplementary file 16 — Source data Fig. 8 [file 44318_2024_333_MOESM16_ESM.zip › Figure 8/Figure 8A/CTR siRNA/R.8h/series1_control_recovery__w3DAPI.jpg]

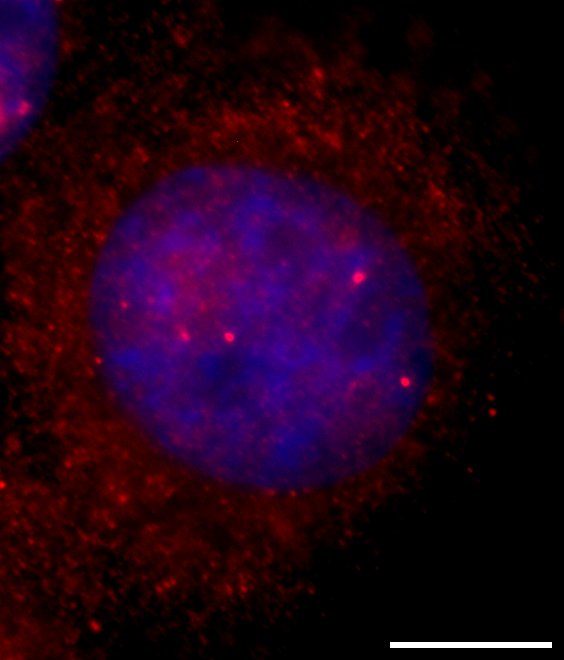

Supplement: Supplementary file 16 — Source data Fig. 8 [file 44318_2024_333_MOESM16_ESM.zip › Figure 8/Figure 8A/p42 siRNA/CTR/Composite.jpg]

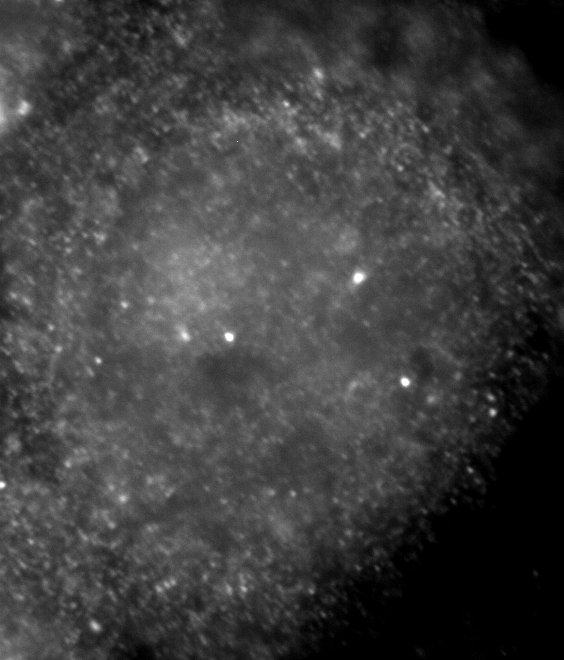

Supplement: Supplementary file 16 — Source data Fig. 8 [file 44318_2024_333_MOESM16_ESM.zip › Figure 8/Figure 8A/p42 siRNA/CTR/series1_si42_untreated_5_w1AF-546.jpg]

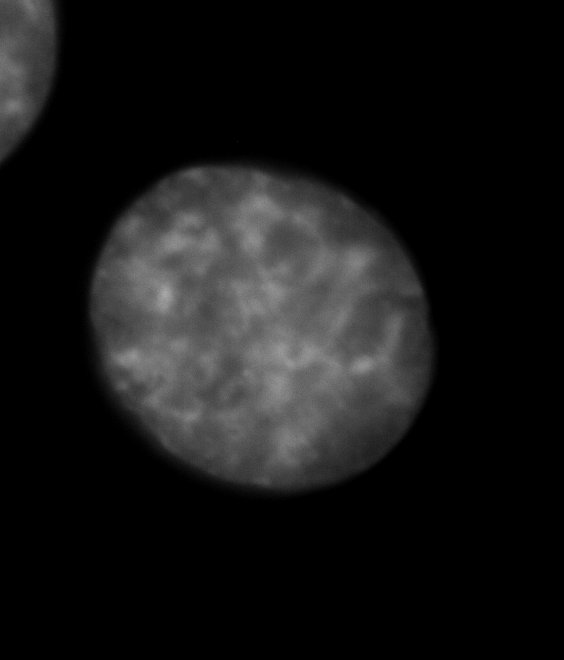

Supplement: Supplementary file 16 — Source data Fig. 8 [file 44318_2024_333_MOESM16_ESM.zip › Figure 8/Figure 8A/p42 siRNA/CTR/series1_si42_untreated_5_w2DAPI.jpg]

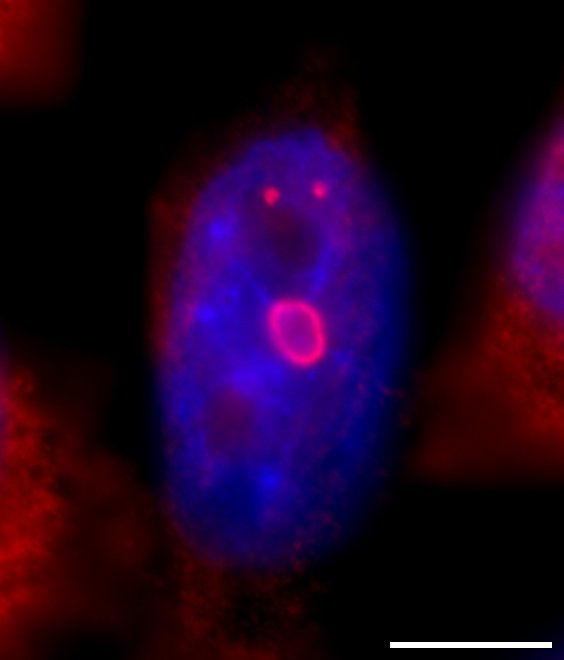

Supplement: Supplementary file 16 — Source data Fig. 8 [file 44318_2024_333_MOESM16_ESM.zip › Figure 8/Figure 8A/p42 siRNA/MG132/Composite.jpg]

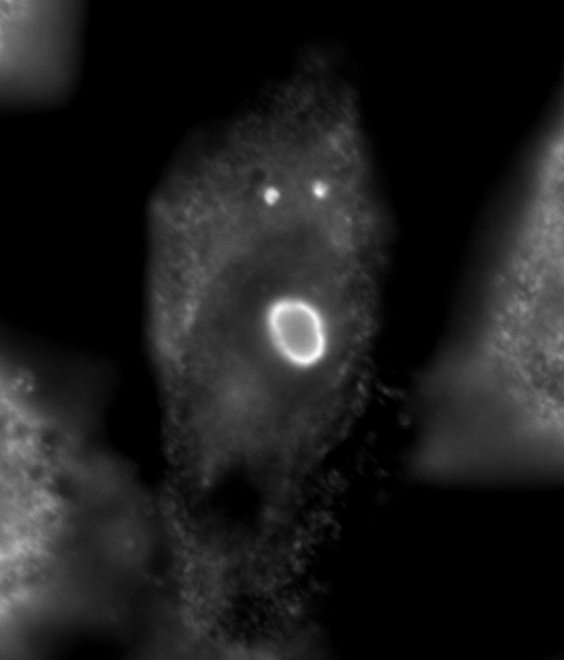

Supplement: Supplementary file 16 — Source data Fig. 8 [file 44318_2024_333_MOESM16_ESM.zip › Figure 8/Figure 8A/p42 siRNA/MG132/series1_si42_MG_ON_2_w1AF-546.jpg]

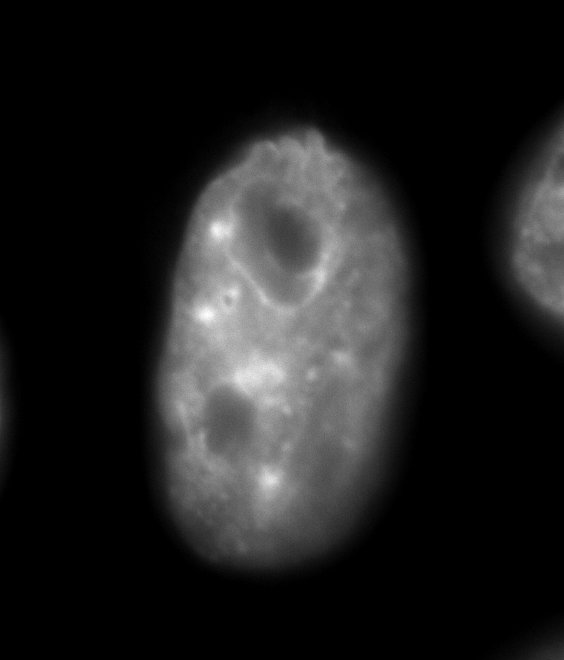

Supplement: Supplementary file 16 — Source data Fig. 8 [file 44318_2024_333_MOESM16_ESM.zip › Figure 8/Figure 8A/p42 siRNA/MG132/series1_si42_MG_ON_2_w2DAPI.jpg]

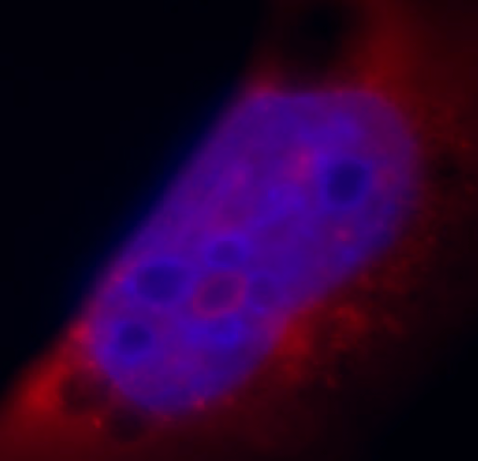

Supplement: Supplementary file 16 — Source data Fig. 8 [file 44318_2024_333_MOESM16_ESM.zip › Figure 8/Figure 8A/p42 siRNA/R.8h/composite.tif]

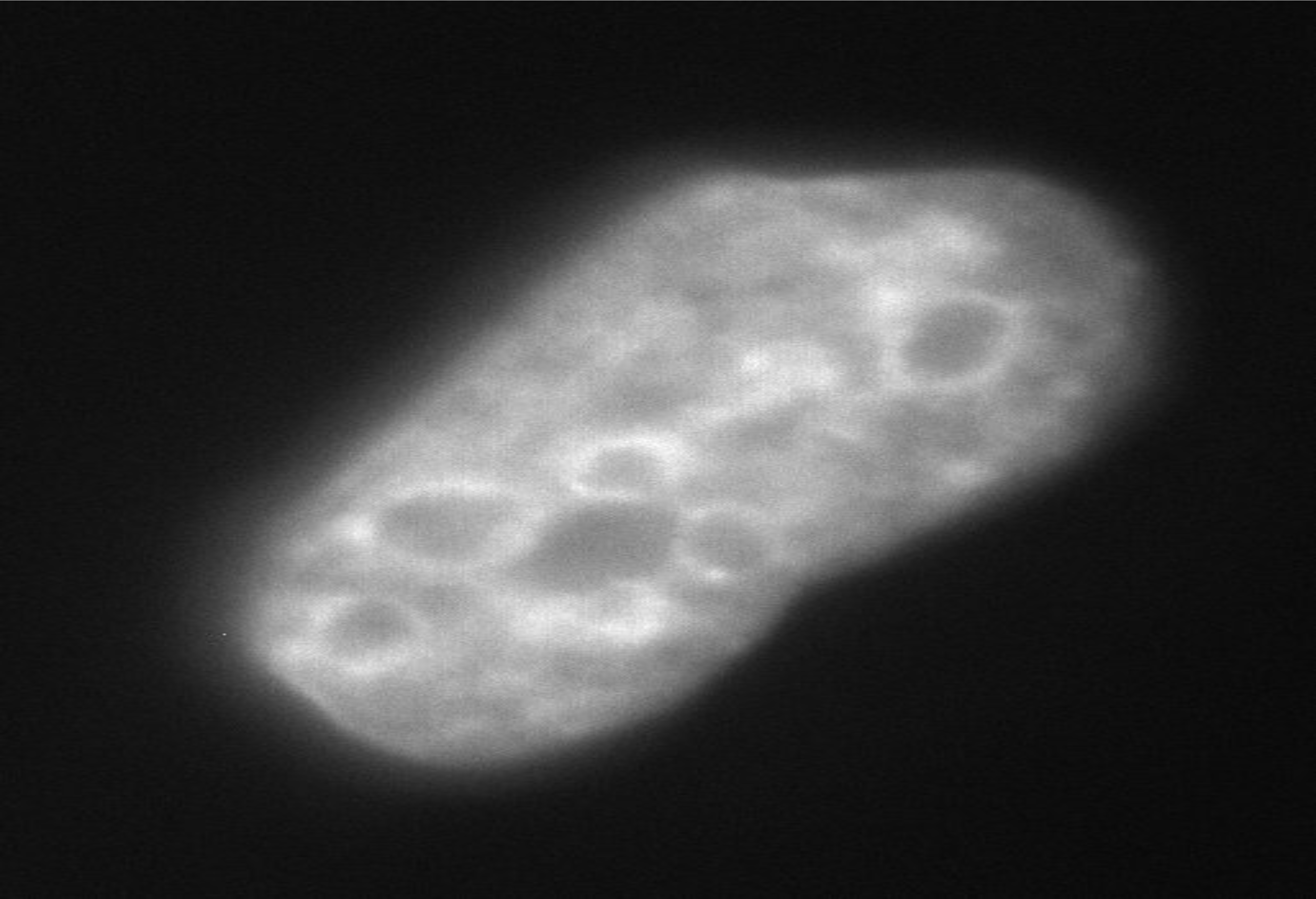

Supplement: Supplementary file 16 — Source data Fig. 8 [file 44318_2024_333_MOESM16_ESM.zip › Figure 8/Figure 8A/p42 siRNA/R.8h/si42_recov8h_DAPI.tif]

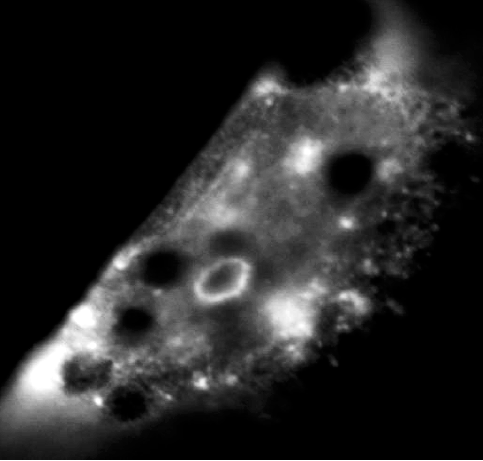

Supplement: Supplementary file 16 — Source data Fig. 8 [file 44318_2024_333_MOESM16_ESM.zip › Figure 8/Figure 8A/p42 siRNA/R.8h/si4_recov8h_Ub.tif]

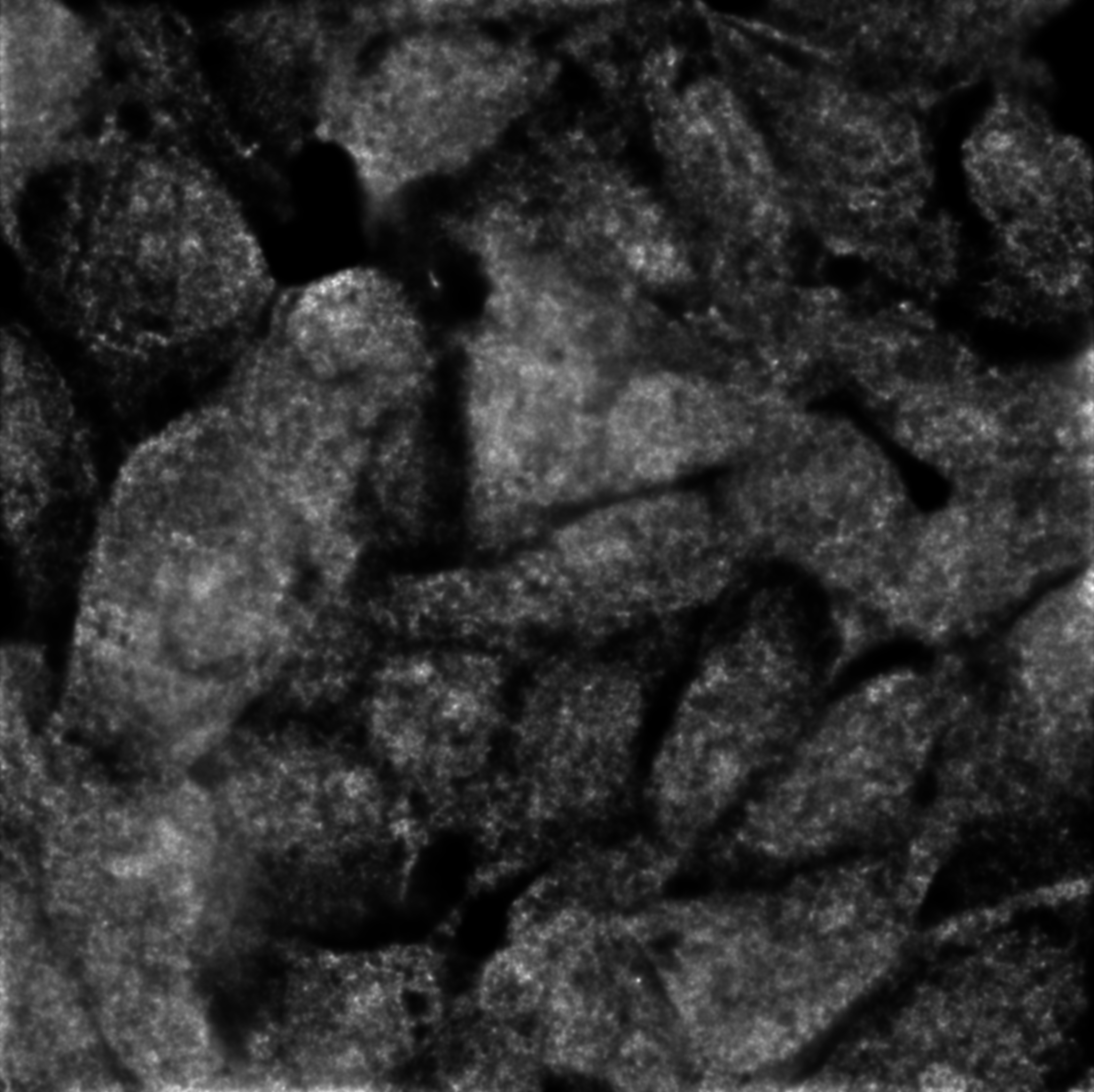

Supplement: Supplementary file 17 — Source data Fig. 9 [file 44318_2024_333_MOESM17_ESM.zip › Figure 9/9A/Cy3_IGS42_CTR.8-bit.tif]

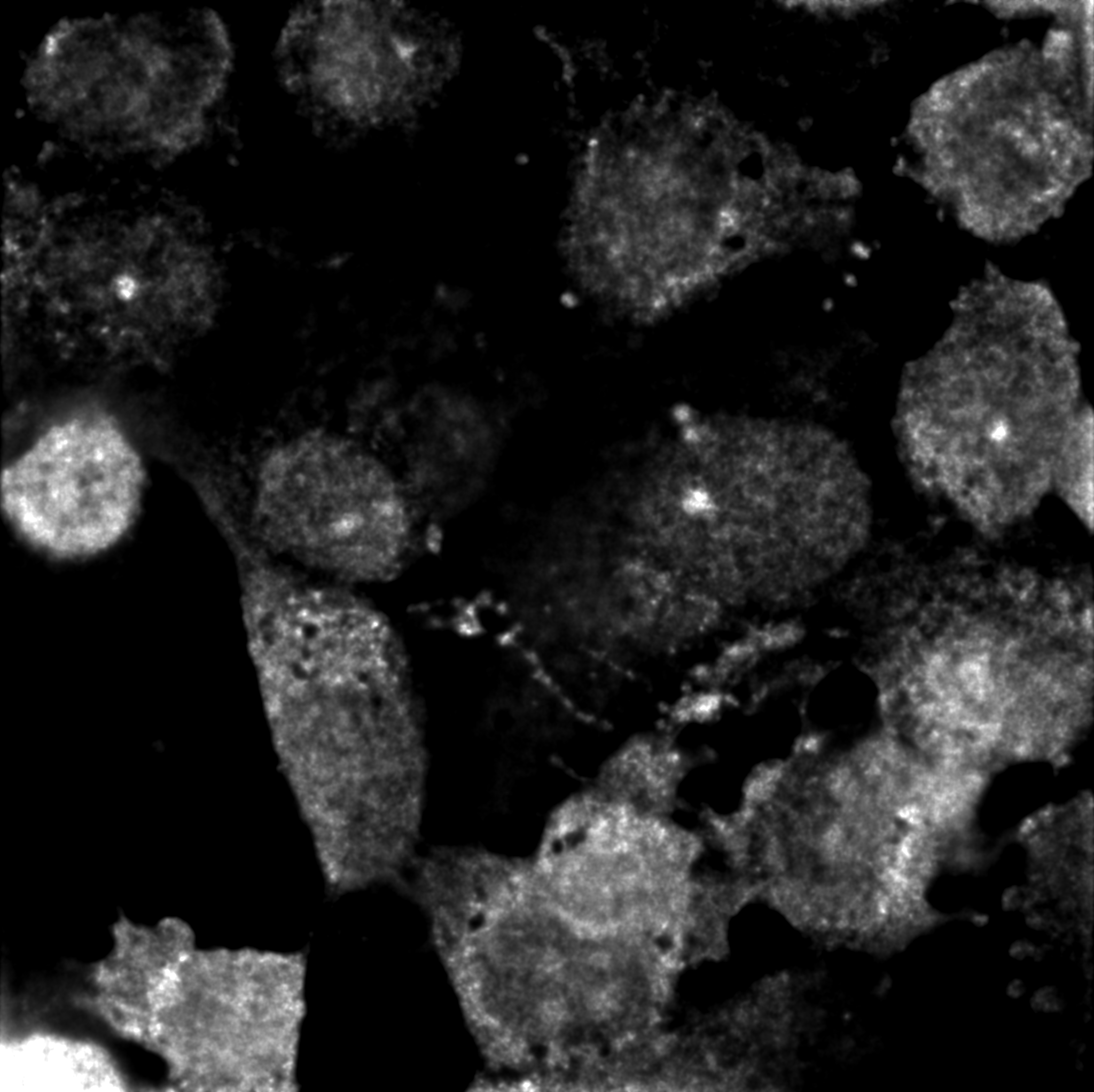

Supplement: Supplementary file 17 — Source data Fig. 9 [file 44318_2024_333_MOESM17_ESM.zip › Figure 9/9A/Cy3_IGS42_MG132.8-bit.tif]

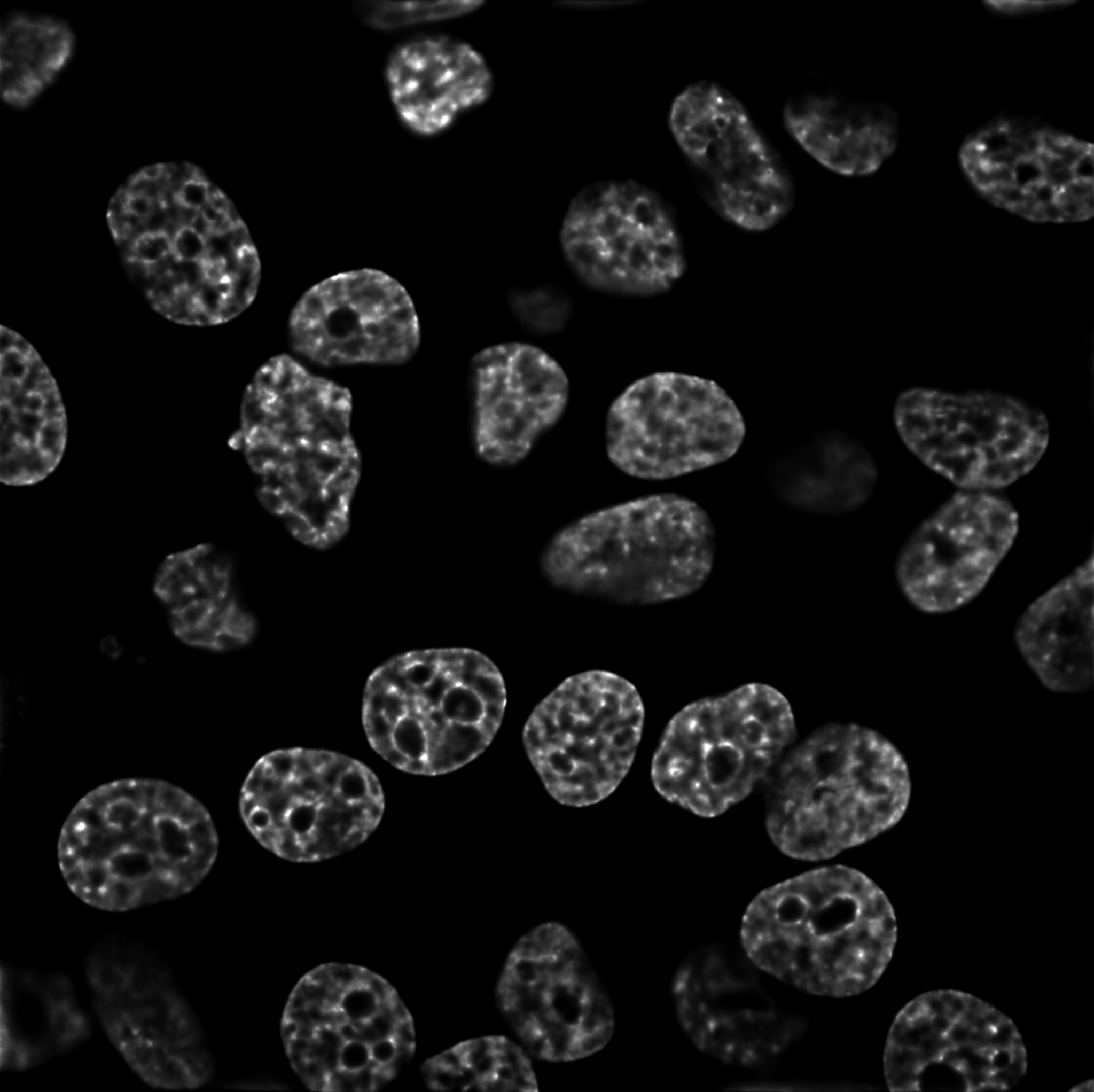

Supplement: Supplementary file 17 — Source data Fig. 9 [file 44318_2024_333_MOESM17_ESM.zip › Figure 9/9A/HOECHST_CTR.8-bit.tif]

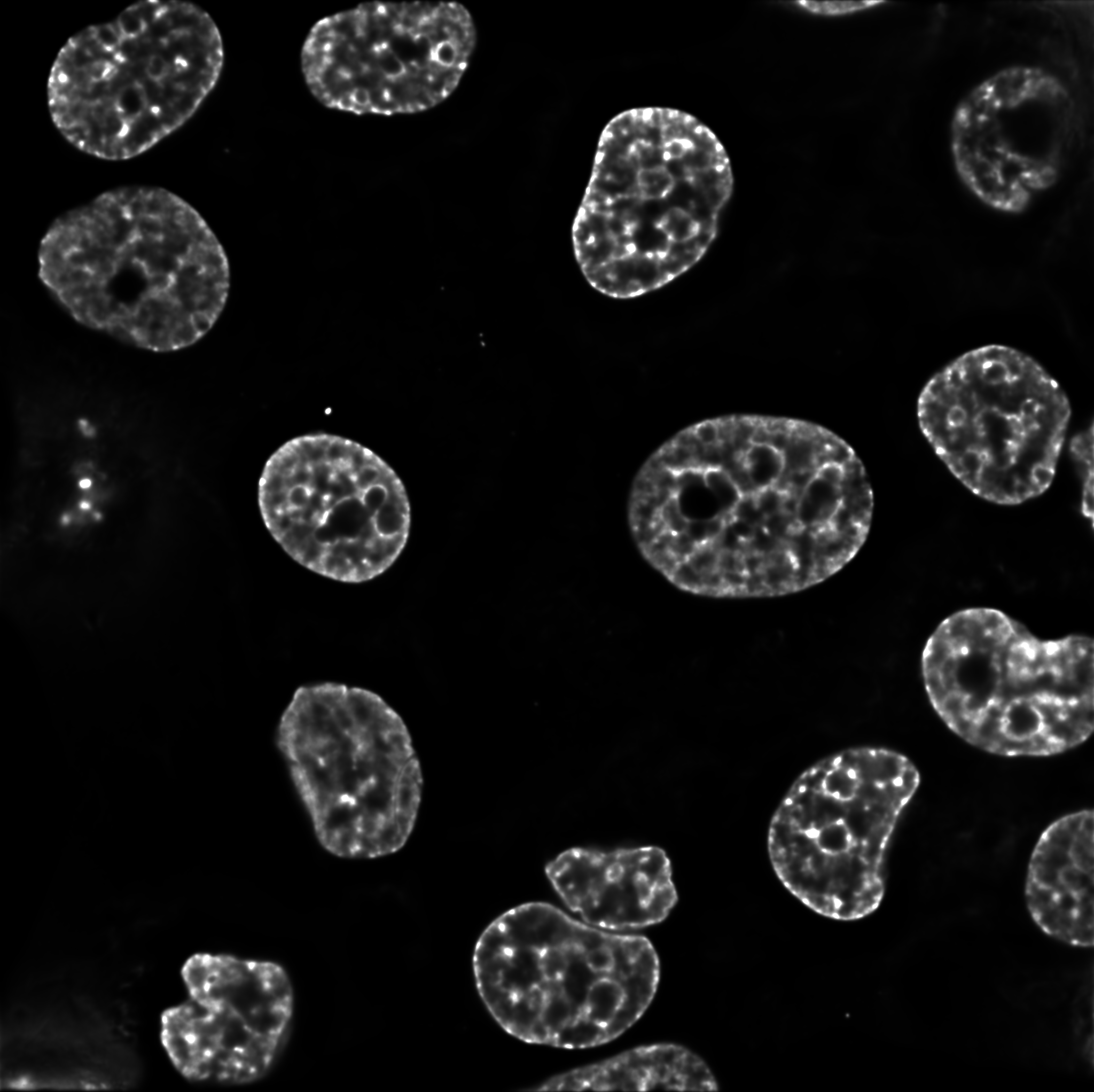

Supplement: Supplementary file 17 — Source data Fig. 9 [file 44318_2024_333_MOESM17_ESM.zip › Figure 9/9A/HOECHST_MG132.8-bit.tif]

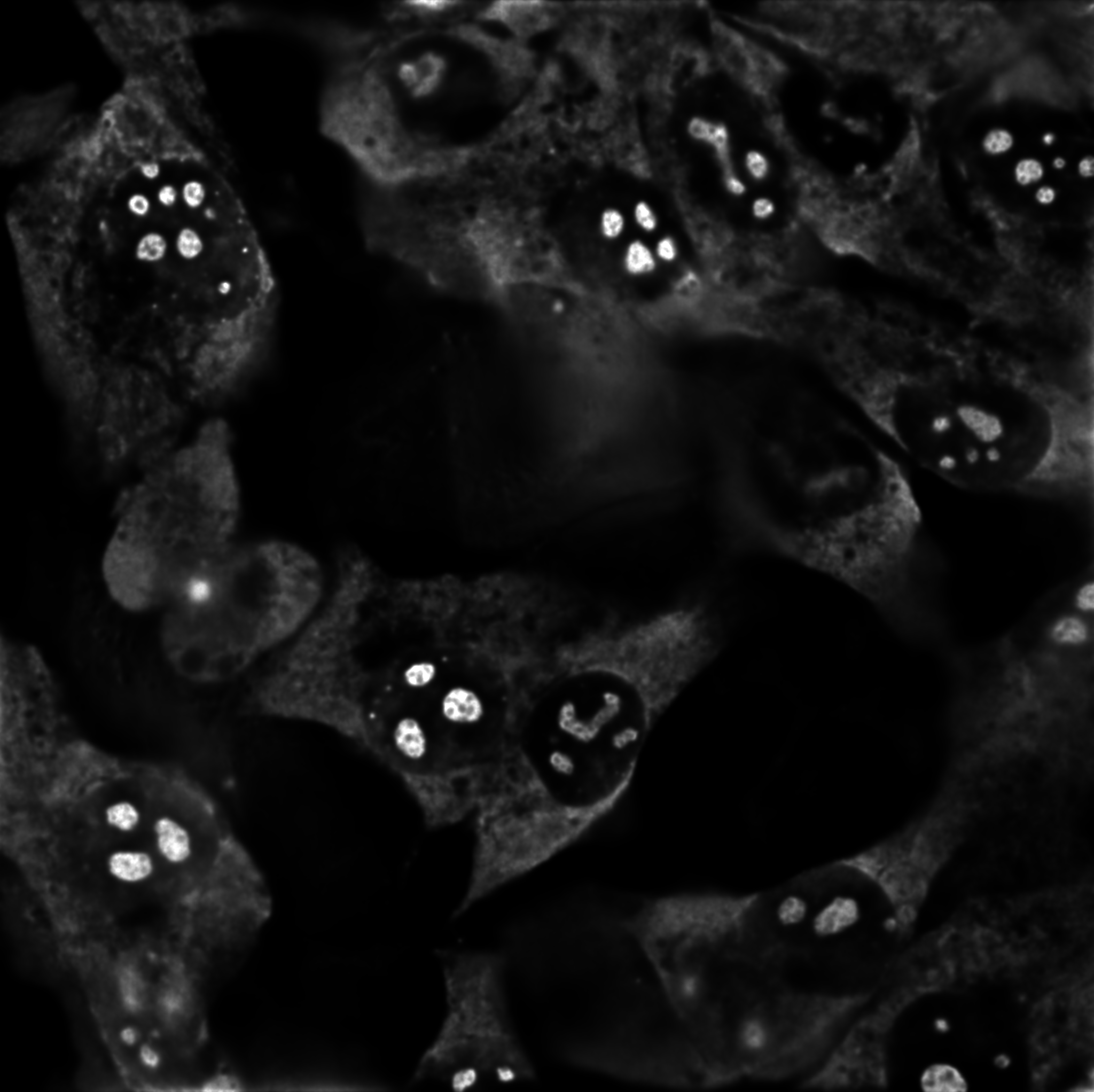

Supplement: Supplementary file 17 — Source data Fig. 9 [file 44318_2024_333_MOESM17_ESM.zip › Figure 9/9A/RPL11_CTR.8-bit.tif]

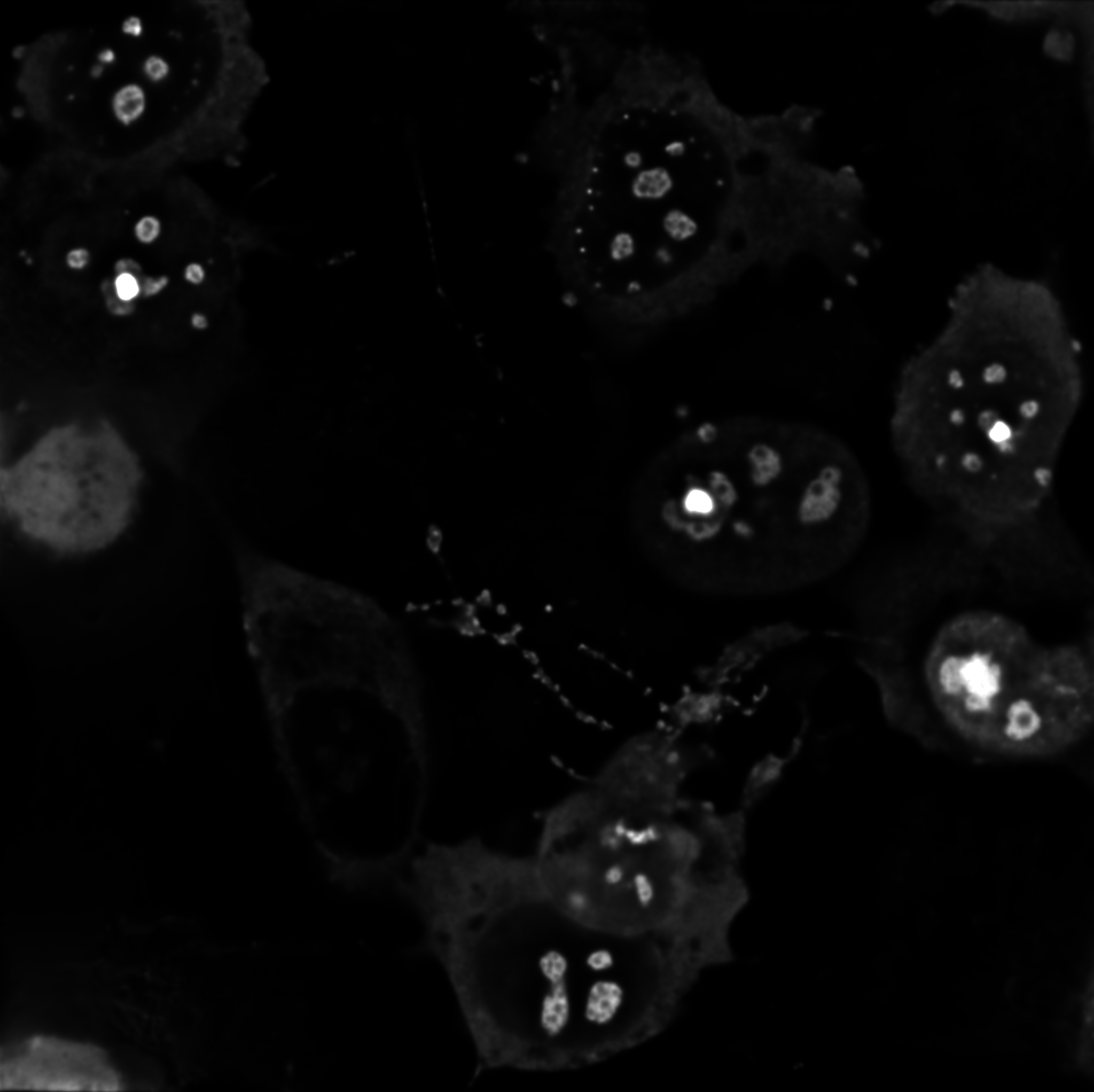

Supplement: Supplementary file 17 — Source data Fig. 9 [file 44318_2024_333_MOESM17_ESM.zip › Figure 9/9A/RPL11_MG132.8-bit.tif]

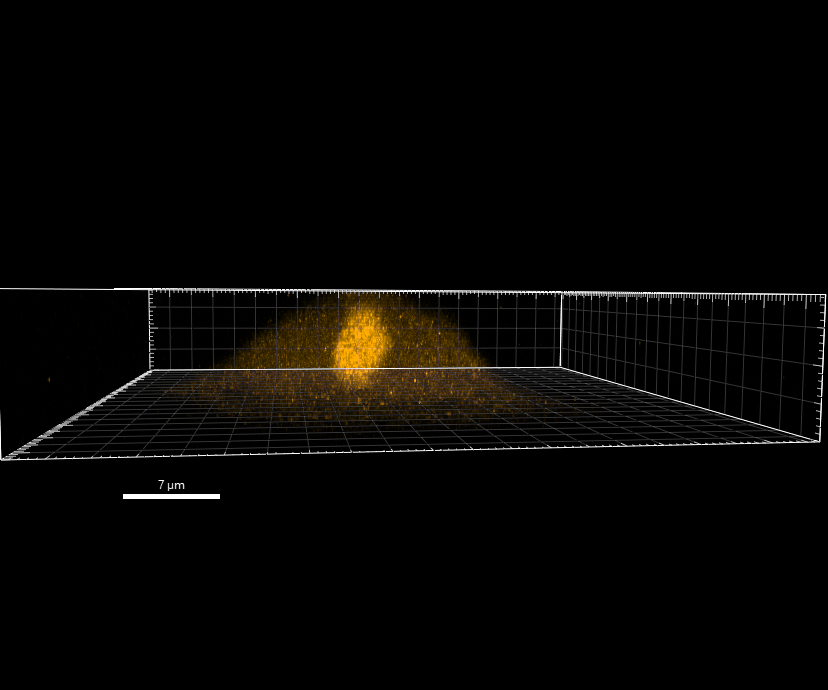

Supplement: Supplementary file 17 — Source data Fig. 9 [file 44318_2024_333_MOESM17_ESM.zip › Figure 9/9B/H12-RPL11GFP-IGS42cy3-zstack-2_2024-10-23T15-28-19.336.tif]

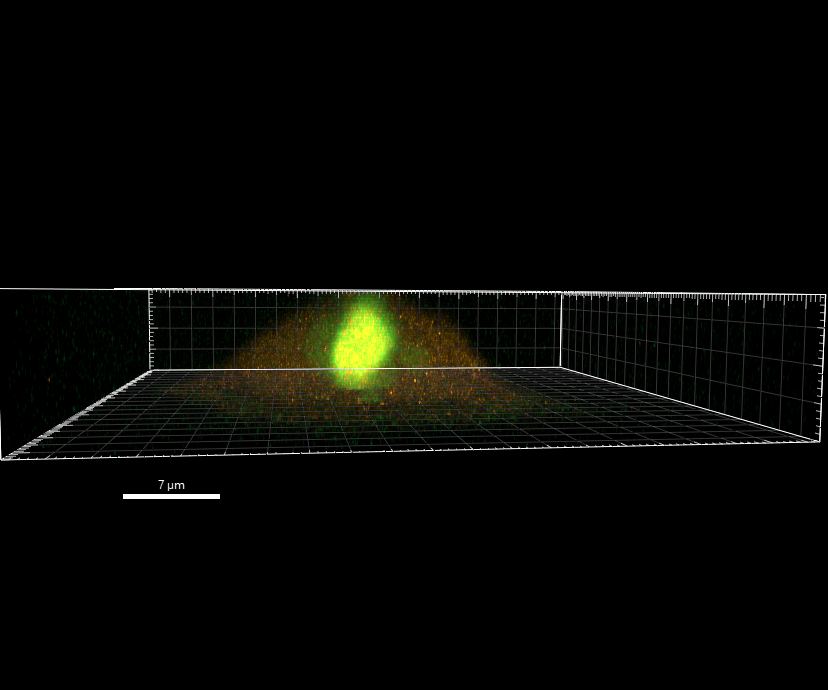

Supplement: Supplementary file 17 — Source data Fig. 9 [file 44318_2024_333_MOESM17_ESM.zip › Figure 9/9B/H12-RPL11GFP-IGS42cy3-zstack-2_2024-10-23T15-28-22.510.tif]

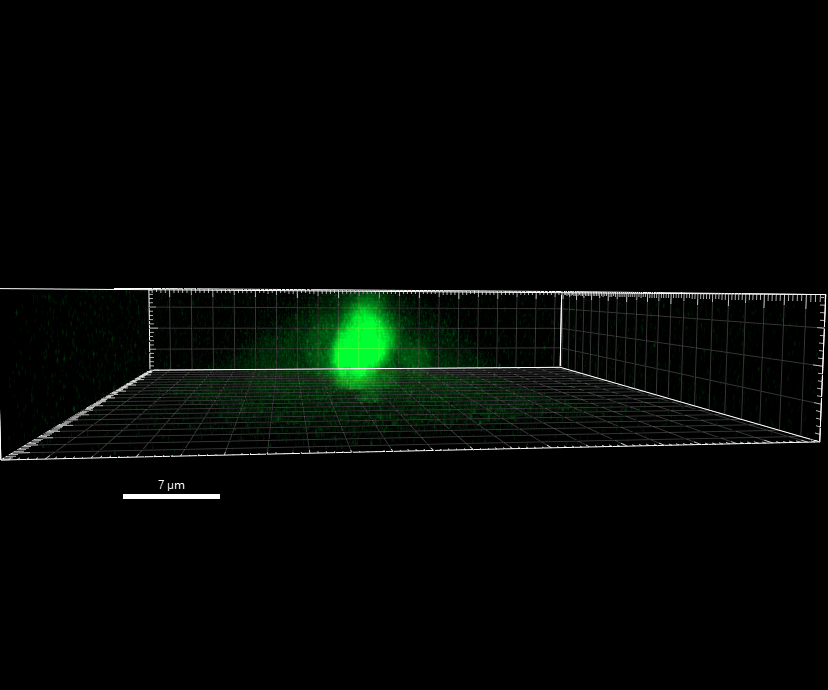

Supplement: Supplementary file 17 — Source data Fig. 9 [file 44318_2024_333_MOESM17_ESM.zip › Figure 9/9B/H12-RPL11GFP-IGS42cy3-zstack-2_2024-10-23T15-28-25.959.tif]

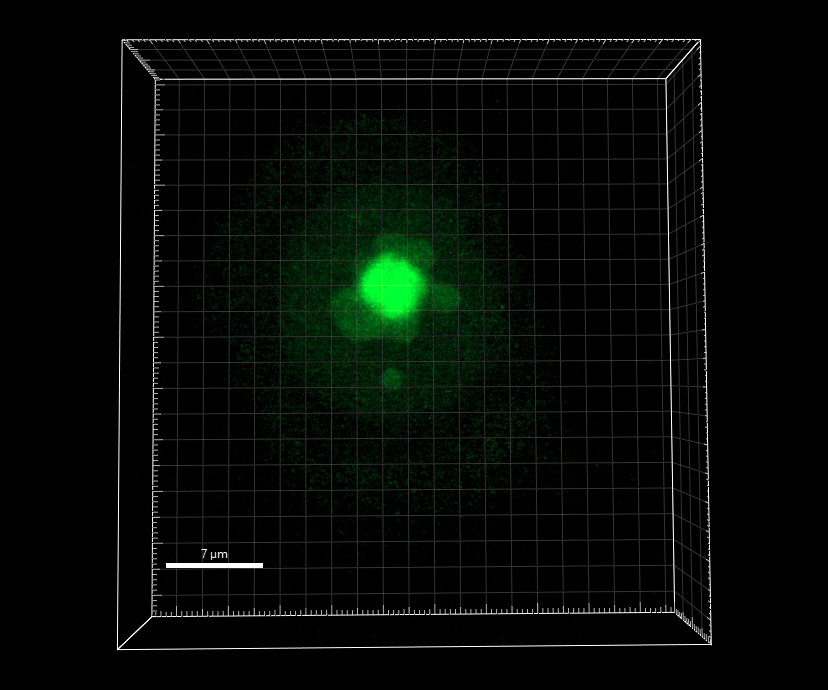

Supplement: Supplementary file 17 — Source data Fig. 9 [file 44318_2024_333_MOESM17_ESM.zip › Figure 9/9B/H12-RPL11GFP-IGS42cy3-zstack-2_2024-10-23T15-30-02.259.tif]

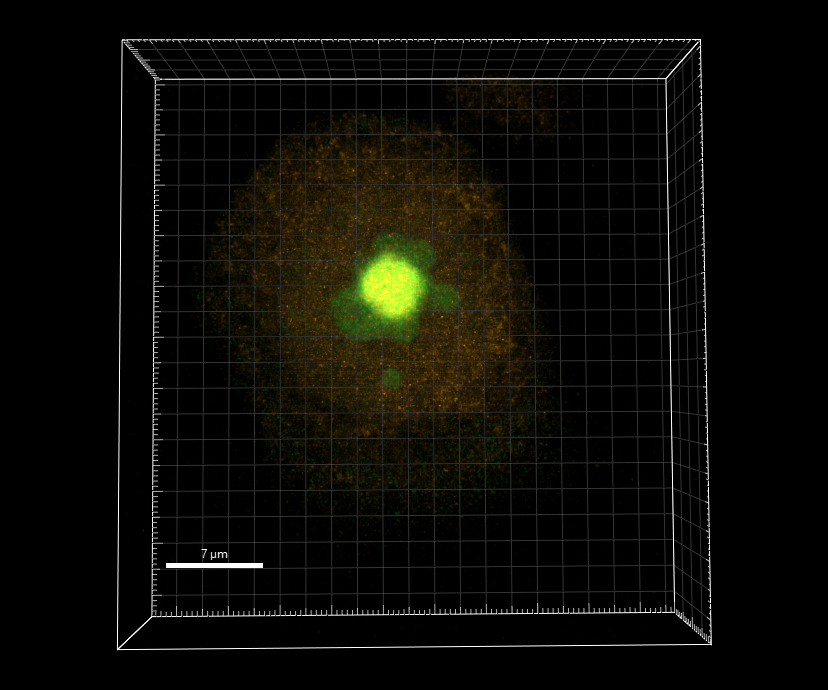

Supplement: Supplementary file 17 — Source data Fig. 9 [file 44318_2024_333_MOESM17_ESM.zip › Figure 9/9B/H12-RPL11GFP-IGS42cy3-zstack-2_2024-10-23T15-30-10.849.tif]

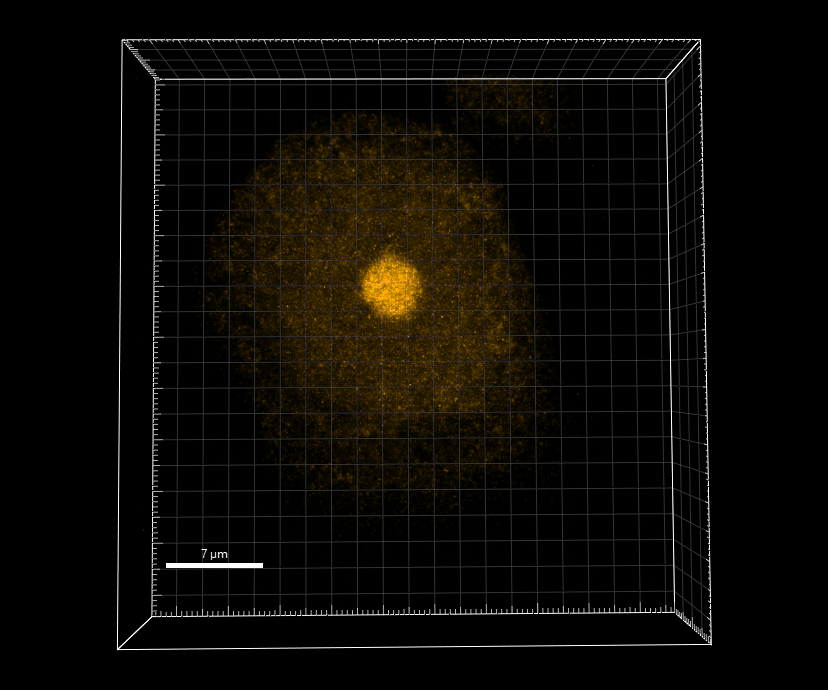

Supplement: Supplementary file 17 — Source data Fig. 9 [file 44318_2024_333_MOESM17_ESM.zip › Figure 9/9B/H12-RPL11GFP-IGS42cy3-zstack-2_2024-10-23T15-30-14.436.tif]
