# Supplementary figures and images for: A nuclear protein quality control system for elimination of nucleolus-related inclusions (part 4 of 4)
Source: EMBO J. 2024 Dec 17;44(3):801–23. doi: 10.1038/s44318-024-00333-9 (PMC11791210; doi:10.1038/s44318-024-00333-9)

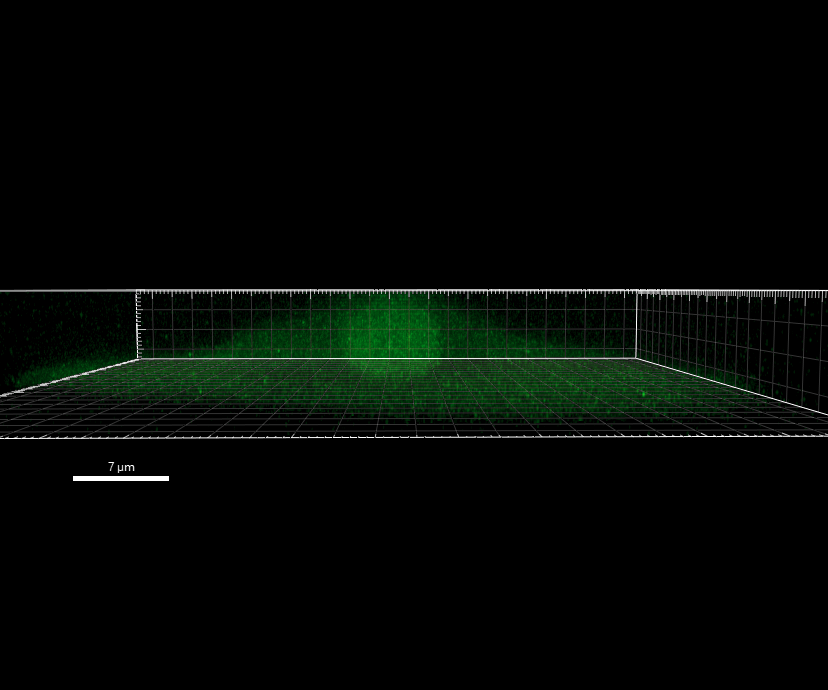

Supplement: Supplementary file 17 — Source data Fig. 9 [file 44318_2024_333_MOESM17_ESM.zip › Figure 9/9B/unstressed_2024-10-23T19-12-48.733.tif]

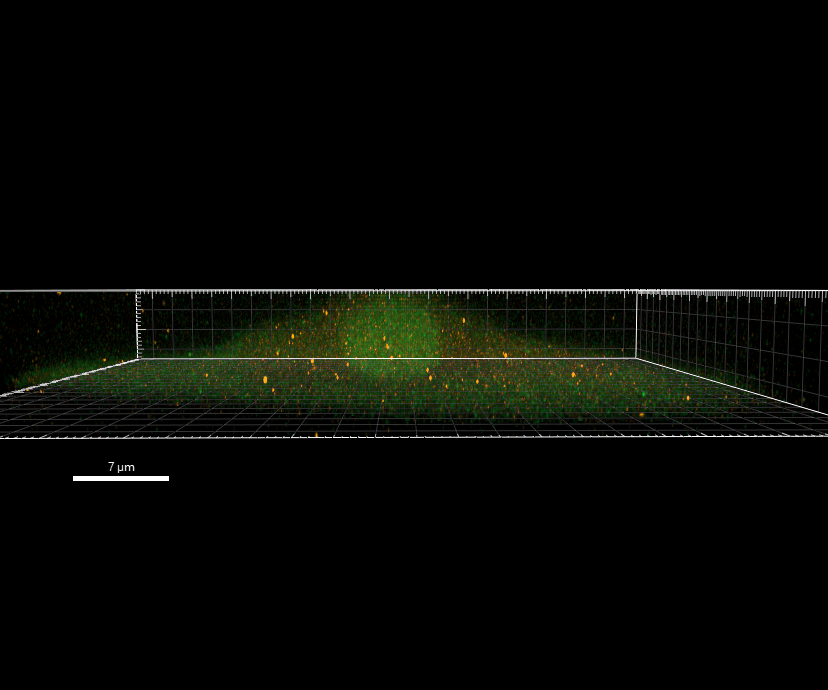

Supplement: Supplementary file 17 — Source data Fig. 9 [file 44318_2024_333_MOESM17_ESM.zip › Figure 9/9B/unstressed_2024-10-23T19-12-53.711.tif]

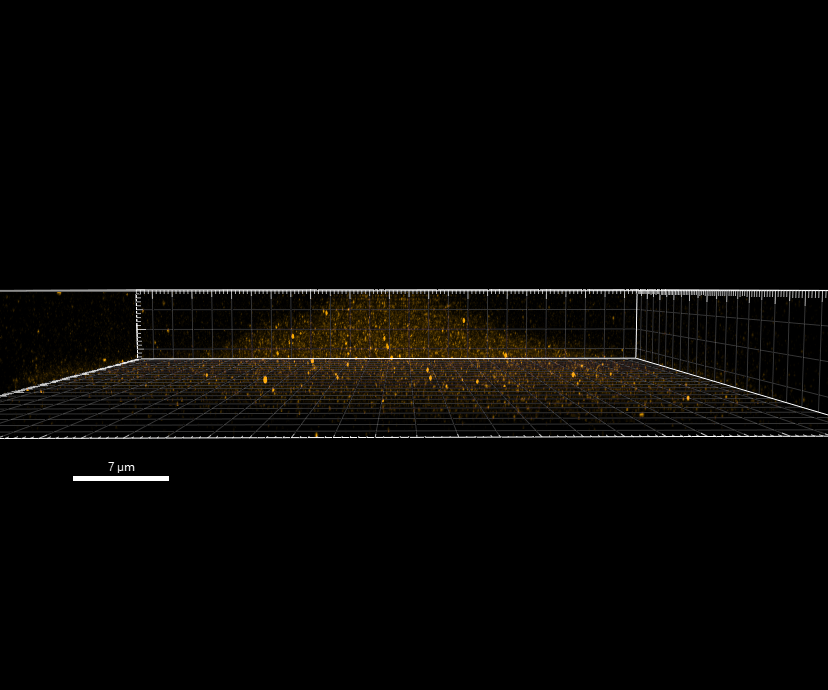

Supplement: Supplementary file 17 — Source data Fig. 9 [file 44318_2024_333_MOESM17_ESM.zip › Figure 9/9B/unstressed_2024-10-23T19-12-57.576.tif]

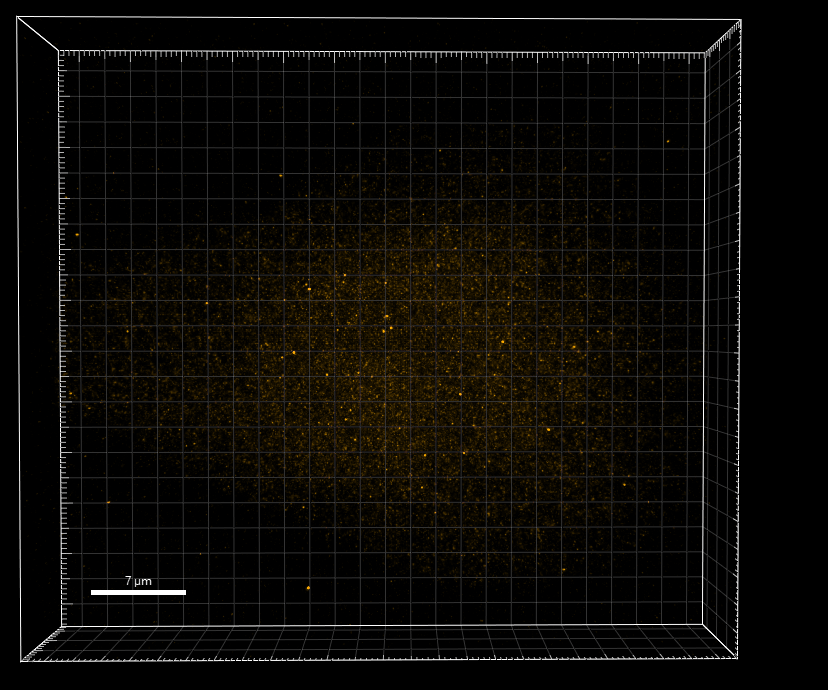

Supplement: Supplementary file 17 — Source data Fig. 9 [file 44318_2024_333_MOESM17_ESM.zip › Figure 9/9B/unstressed_2024-10-23T19-13-18.629.tif]

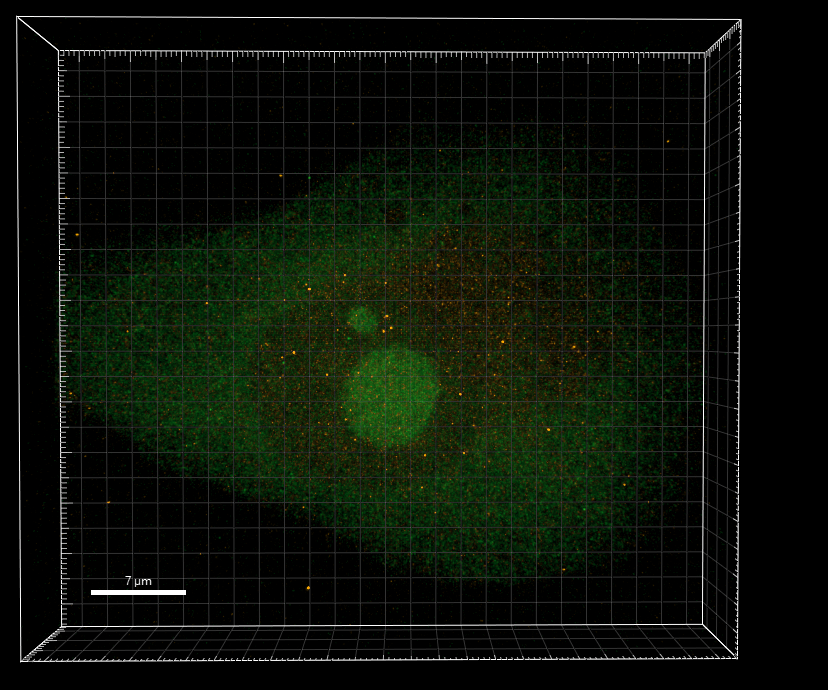

Supplement: Supplementary file 17 — Source data Fig. 9 [file 44318_2024_333_MOESM17_ESM.zip › Figure 9/9B/unstressed_2024-10-23T19-13-22.425.tif]

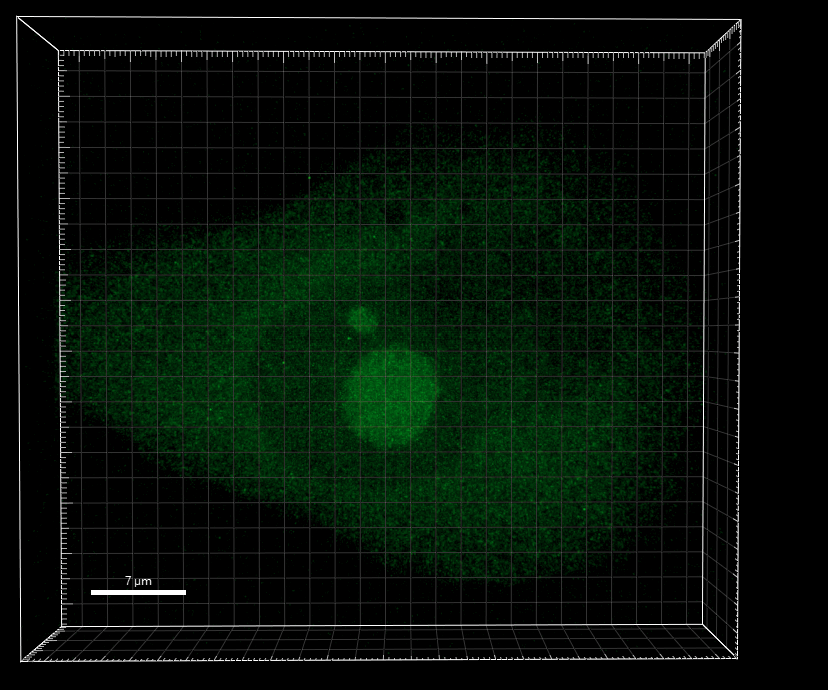

Supplement: Supplementary file 17 — Source data Fig. 9 [file 44318_2024_333_MOESM17_ESM.zip › Figure 9/9B/unstressed_2024-10-23T19-13-26.503.tif]

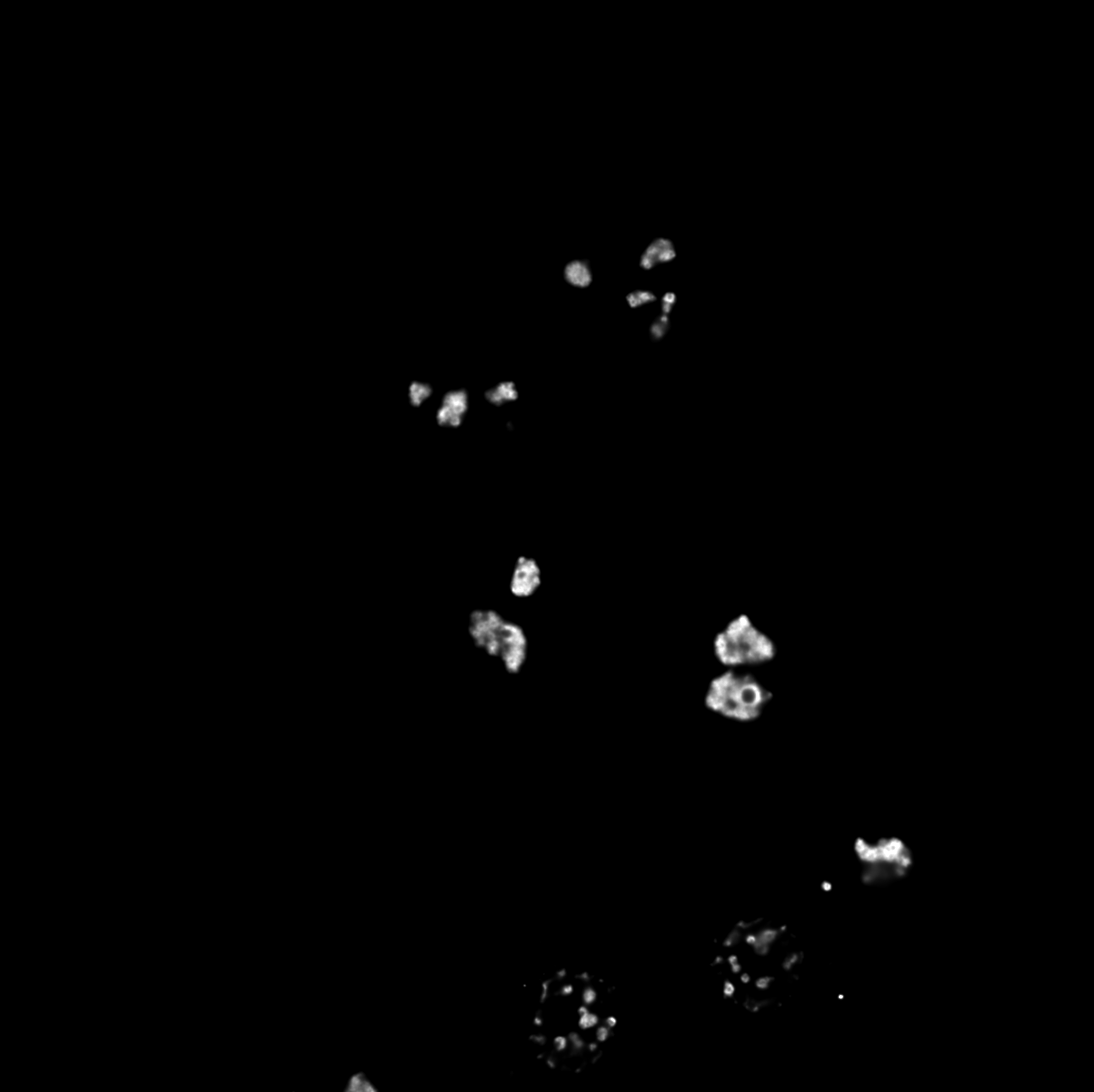

Supplement: Supplementary file 17 — Source data Fig. 9 [file 44318_2024_333_MOESM17_ESM.zip › Figure 9/9C/Cy3_ITS1_CTR.8-bit.tif]

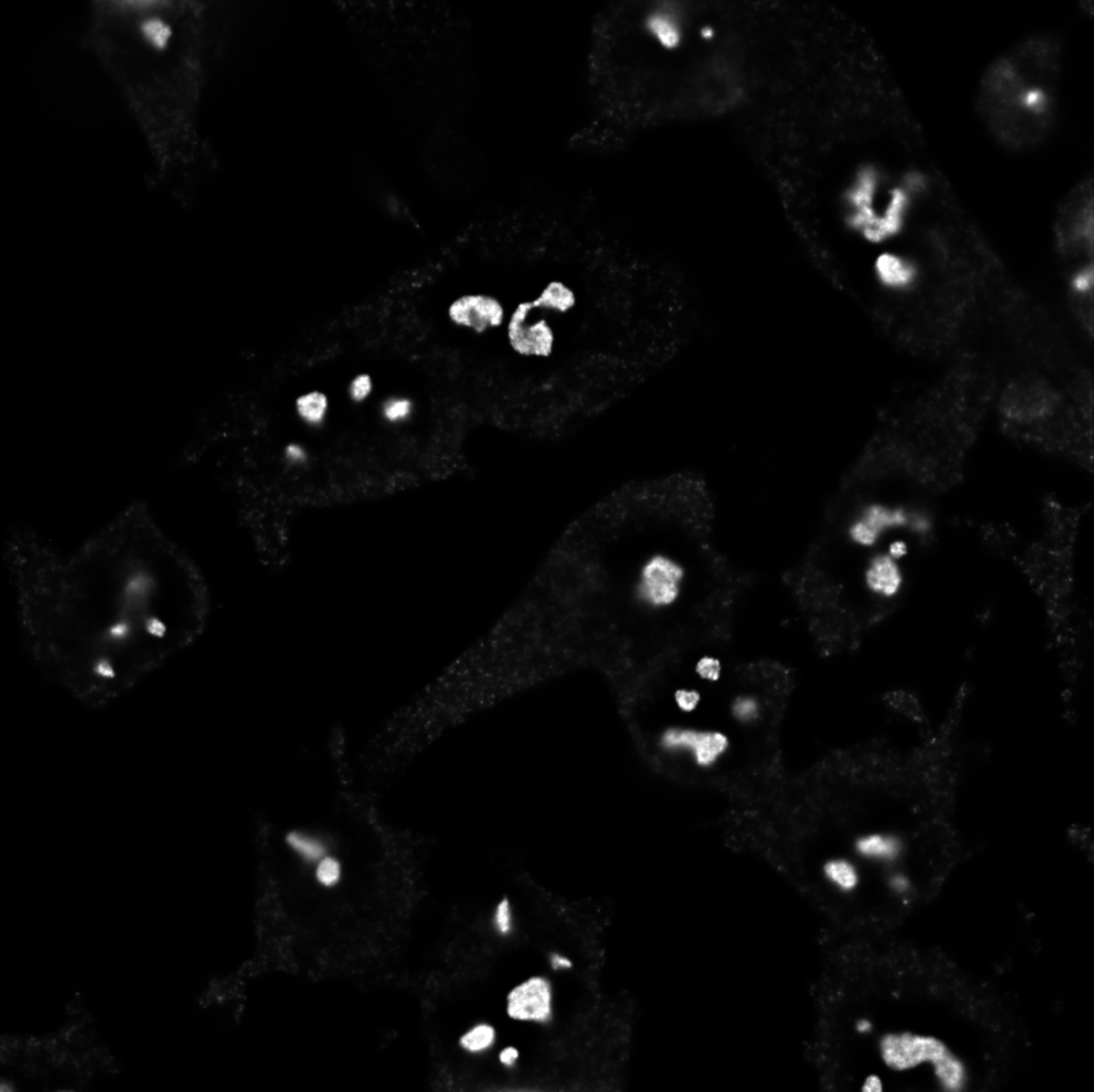

Supplement: Supplementary file 17 — Source data Fig. 9 [file 44318_2024_333_MOESM17_ESM.zip › Figure 9/9C/Cy3_ITS1_MG132.8-bit.tif]

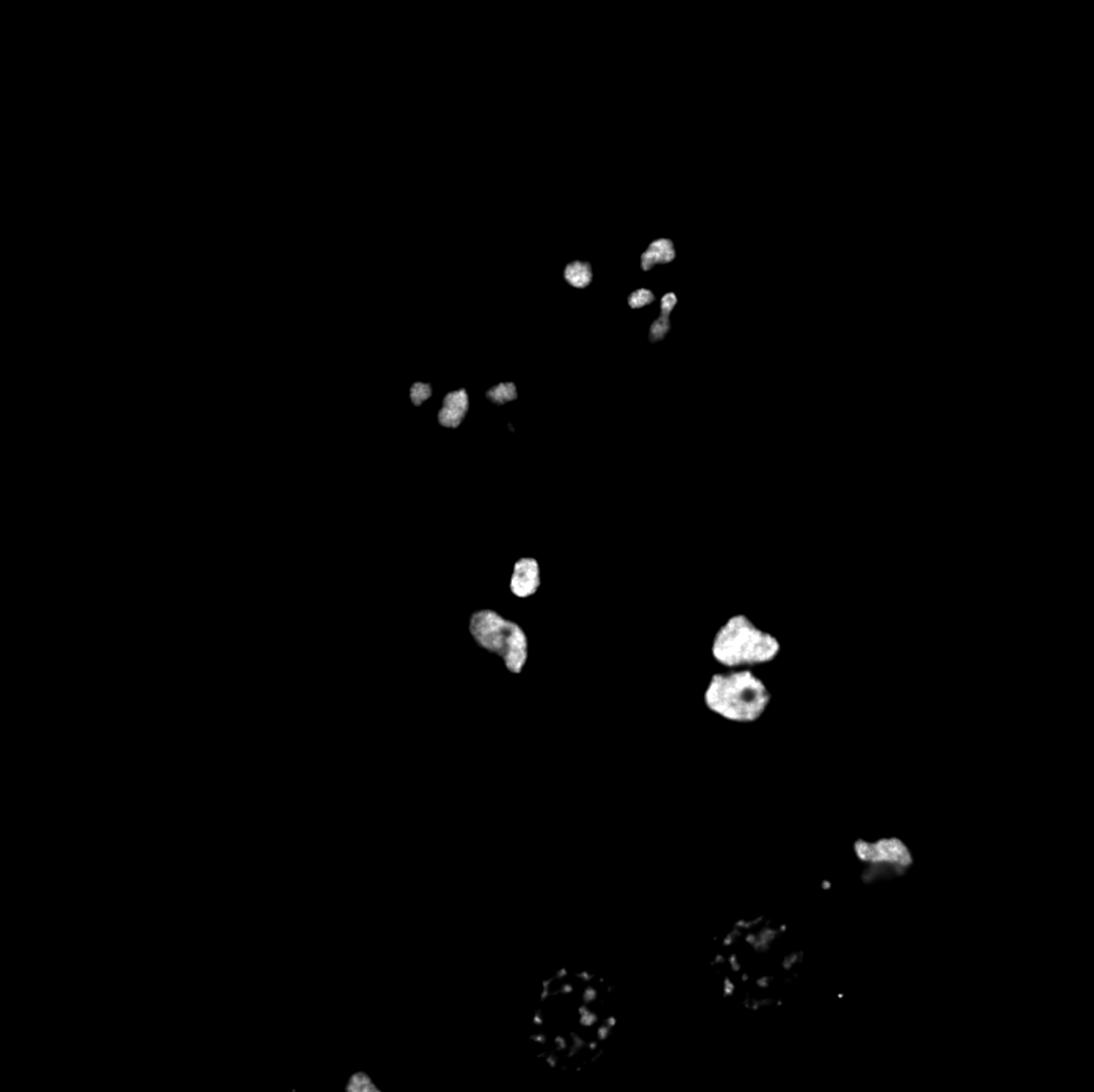

Supplement: Supplementary file 17 — Source data Fig. 9 [file 44318_2024_333_MOESM17_ESM.zip › Figure 9/9C/Cy5_ITS2_CTR.8-bit.tif]

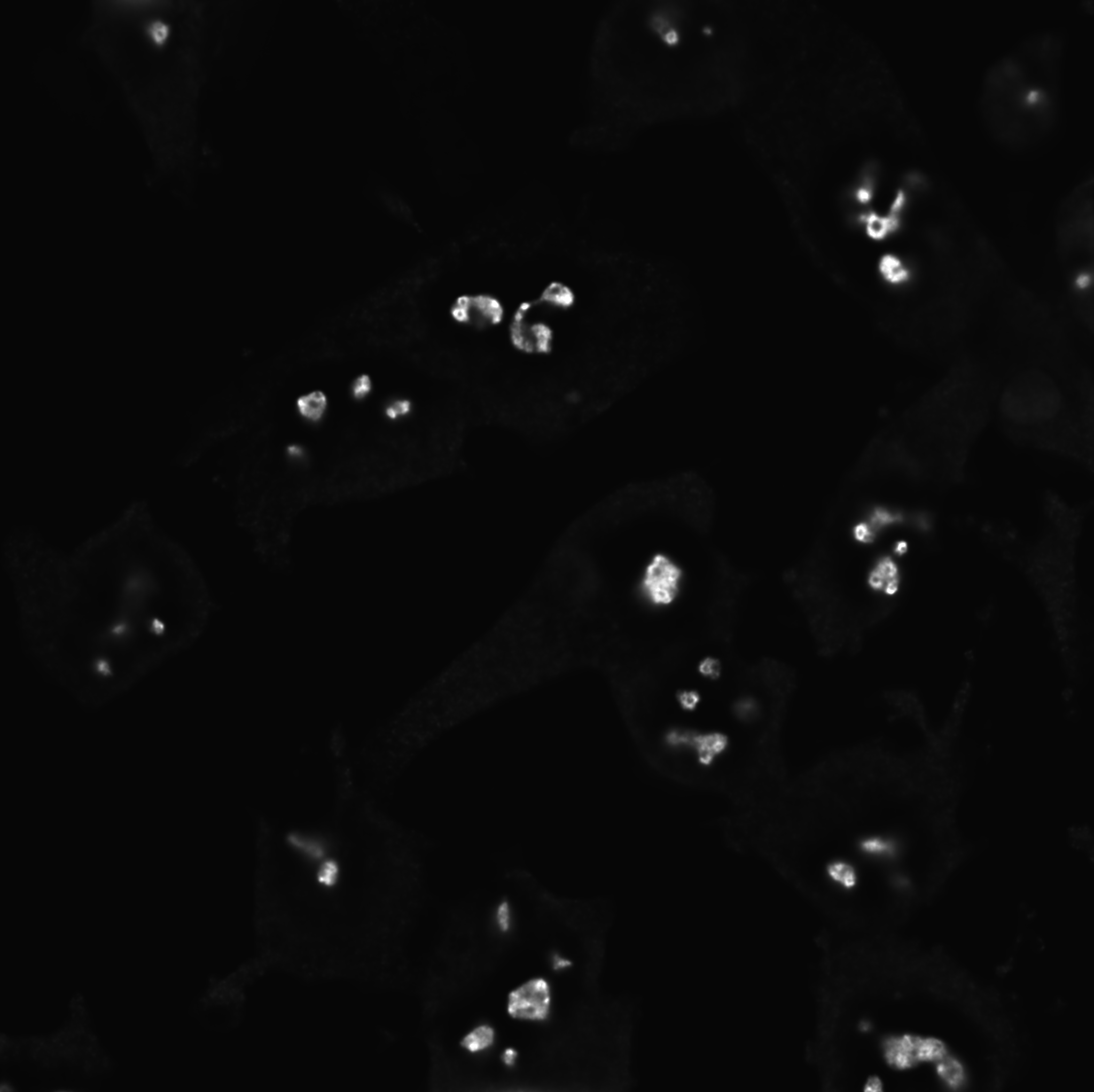

Supplement: Supplementary file 17 — Source data Fig. 9 [file 44318_2024_333_MOESM17_ESM.zip › Figure 9/9C/Cy5_ITS2_MG132.8-bit.tif]

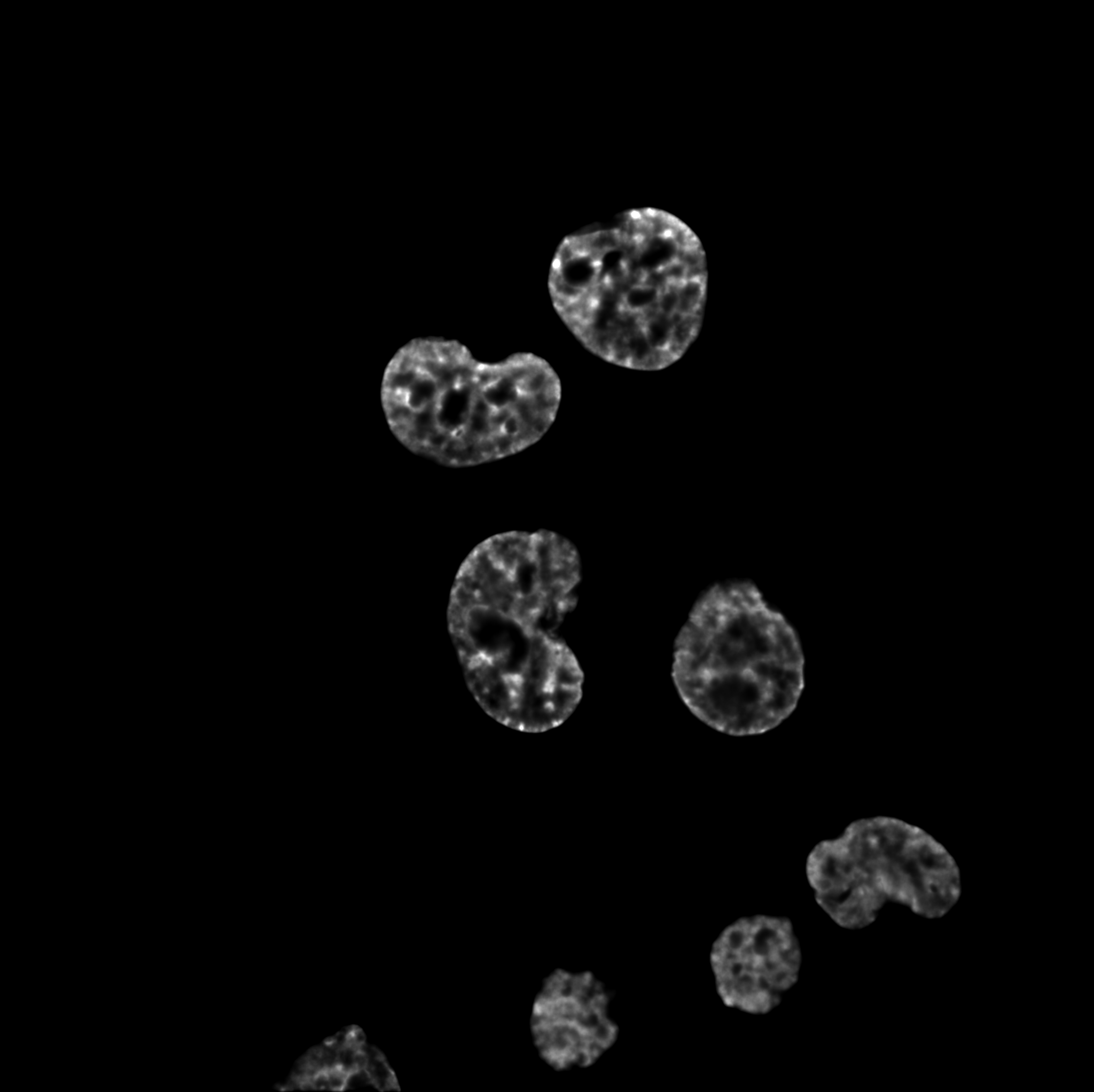

Supplement: Supplementary file 17 — Source data Fig. 9 [file 44318_2024_333_MOESM17_ESM.zip › Figure 9/9C/HOECHST_CTR.8-bit.tif]

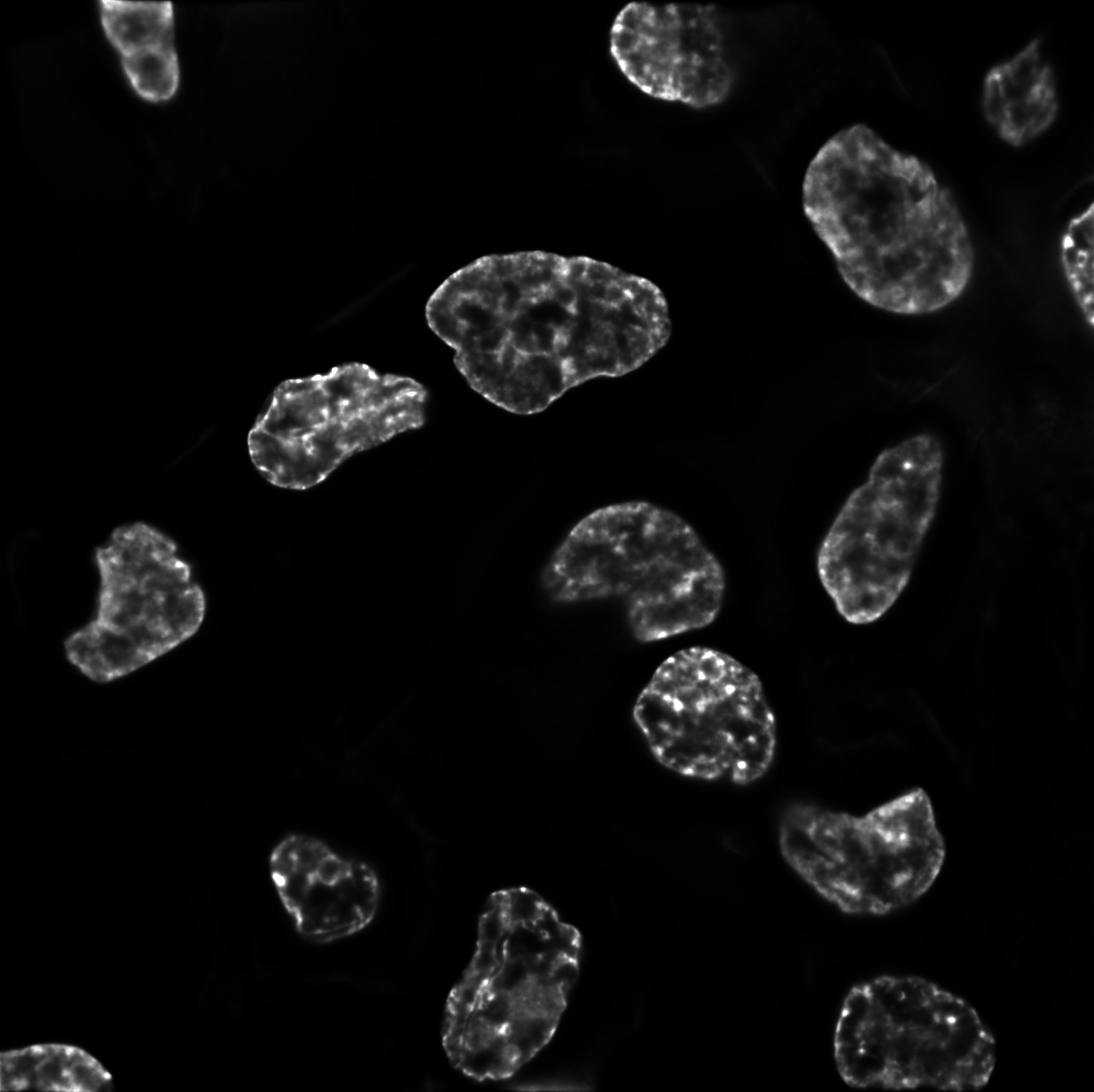

Supplement: Supplementary file 17 — Source data Fig. 9 [file 44318_2024_333_MOESM17_ESM.zip › Figure 9/9C/HOECHST_MG132.8-bit.tif]

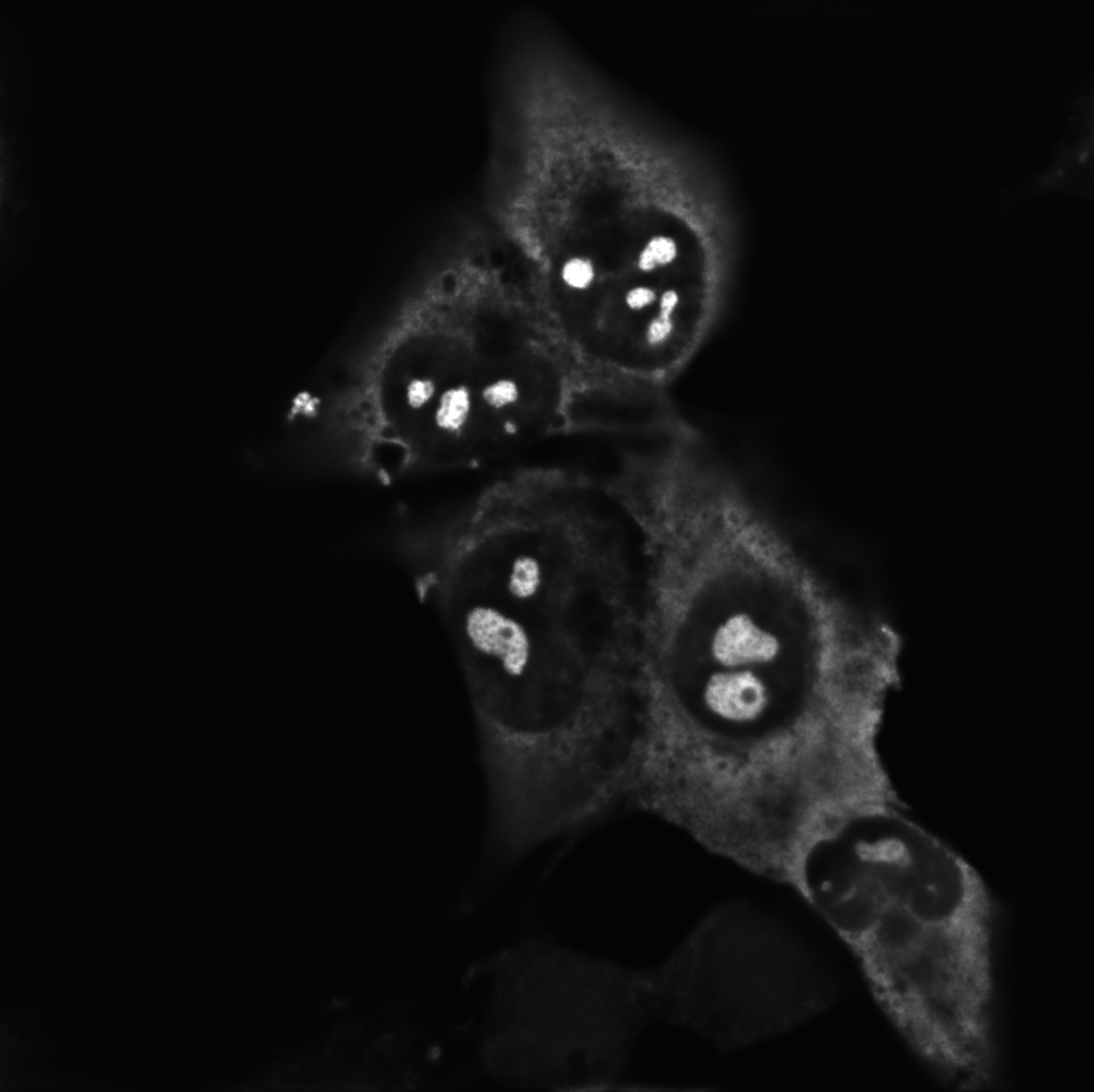

Supplement: Supplementary file 17 — Source data Fig. 9 [file 44318_2024_333_MOESM17_ESM.zip › Figure 9/9C/RPL11_CTR.8-bit.tif]

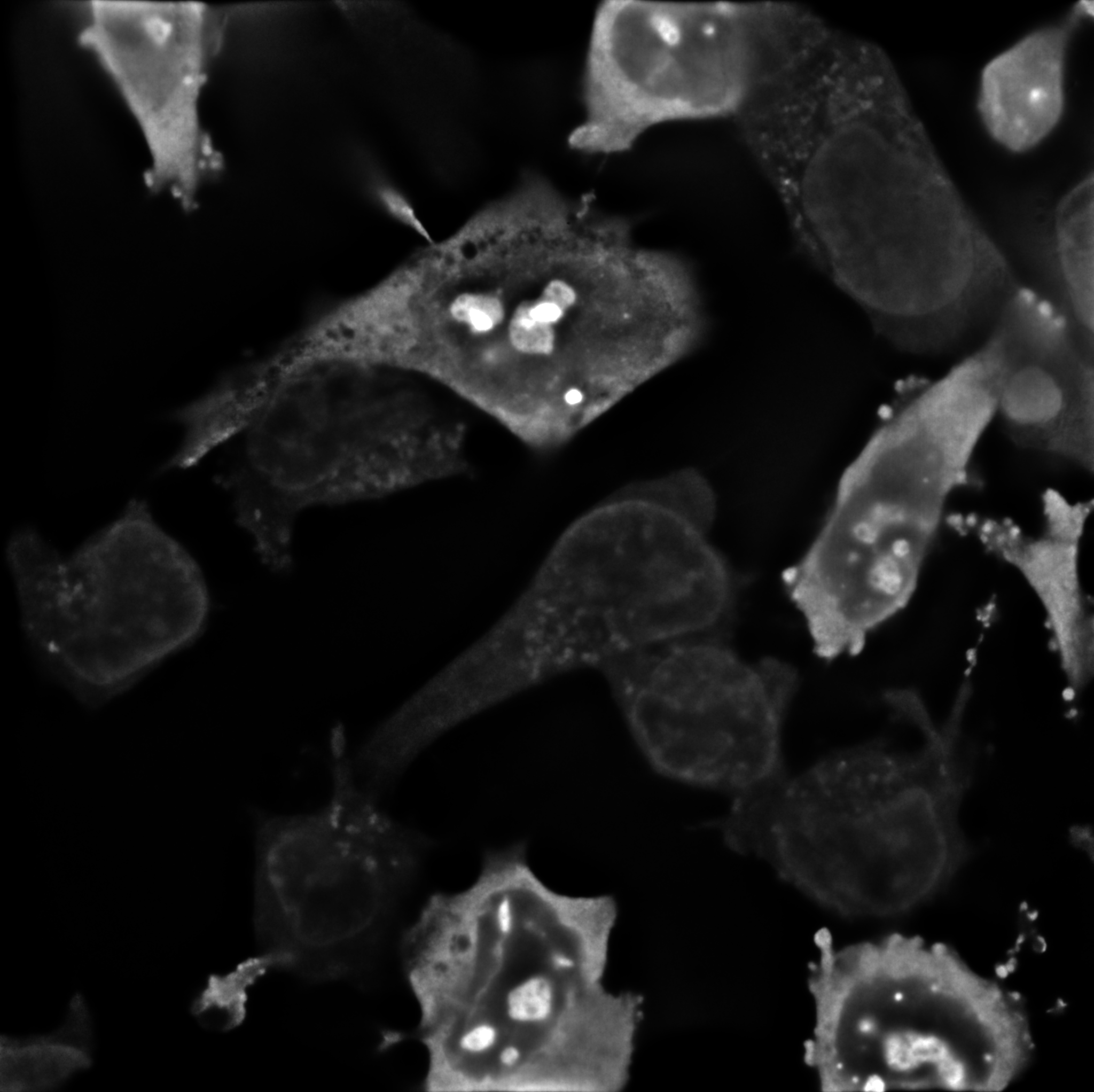

Supplement: Supplementary file 17 — Source data Fig. 9 [file 44318_2024_333_MOESM17_ESM.zip › Figure 9/9C/RPL11_MG132.8-bit.tif]

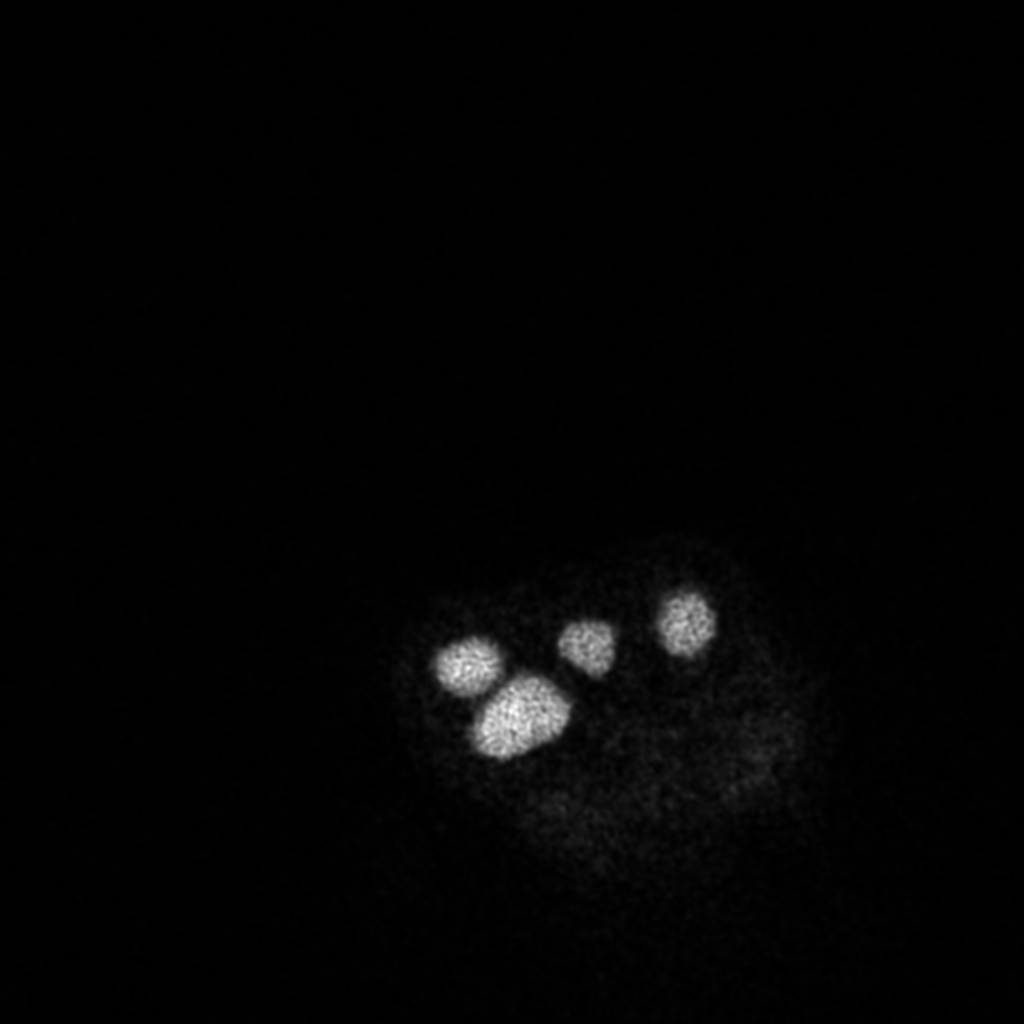

Supplement: Supplementary file 17 — Source data Fig. 9 [file 44318_2024_333_MOESM17_ESM.zip › Figure 9/9D/RIM_Cy3_ITS1_CTR.8-bit.tif]

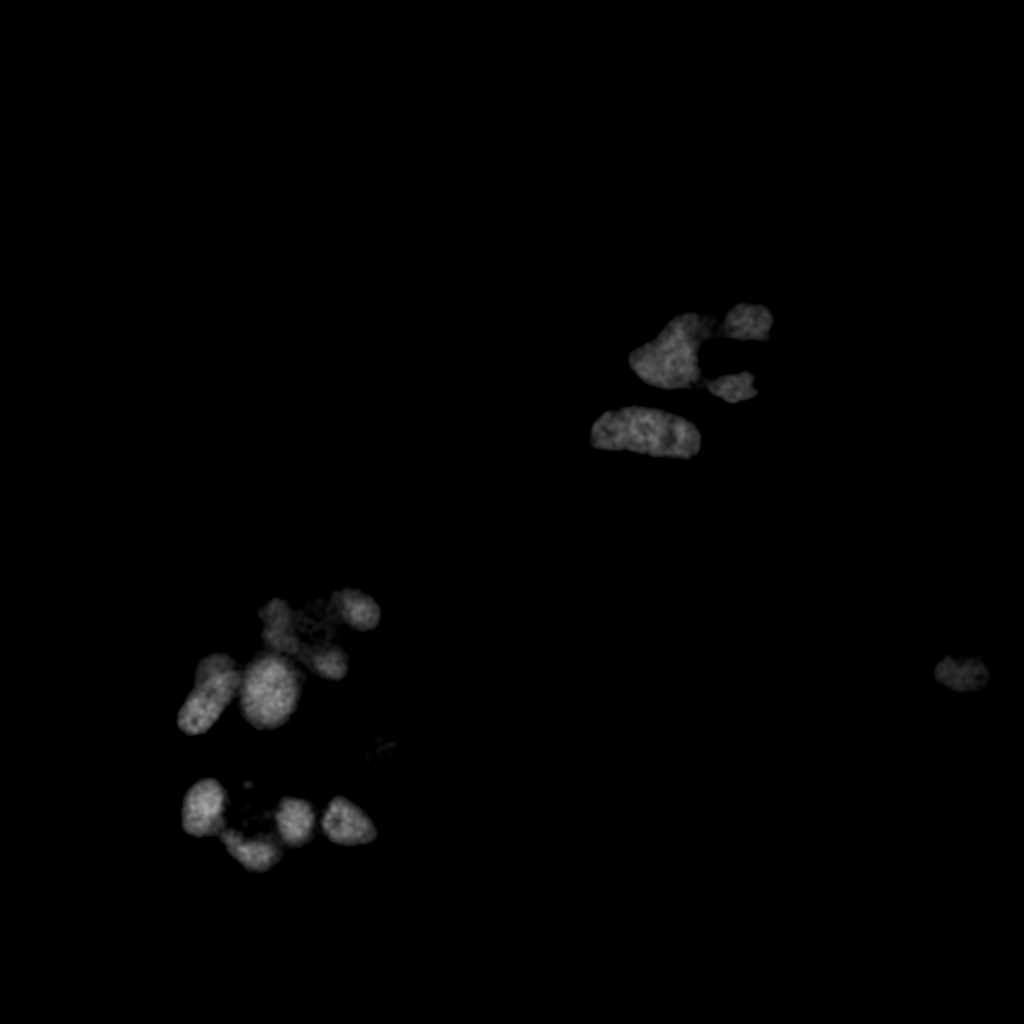

Supplement: Supplementary file 17 — Source data Fig. 9 [file 44318_2024_333_MOESM17_ESM.zip › Figure 9/9D/RIM_Cy3_ITS1_MG132_A.8-bit.tif]

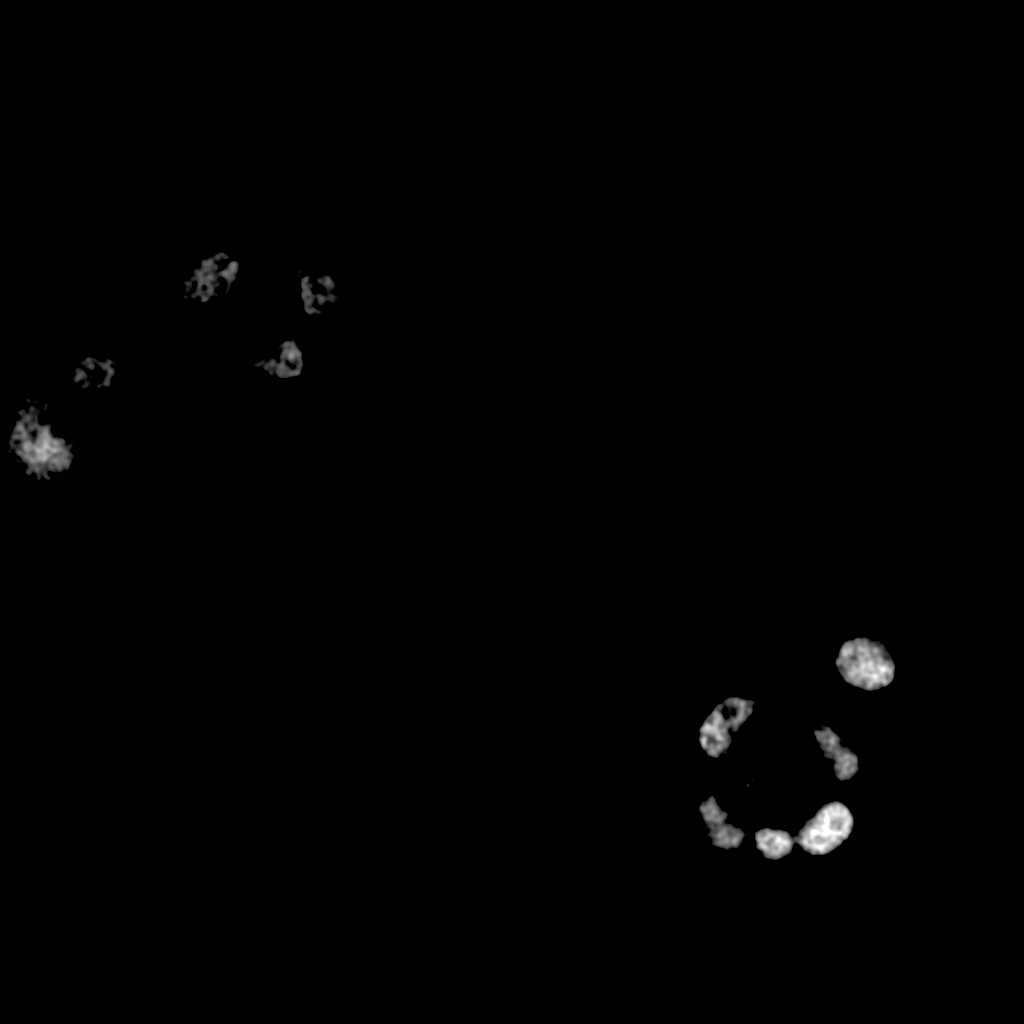

Supplement: Supplementary file 17 — Source data Fig. 9 [file 44318_2024_333_MOESM17_ESM.zip › Figure 9/9D/RIM_Cy3_ITS1_MG132_B.8-bit.tif]

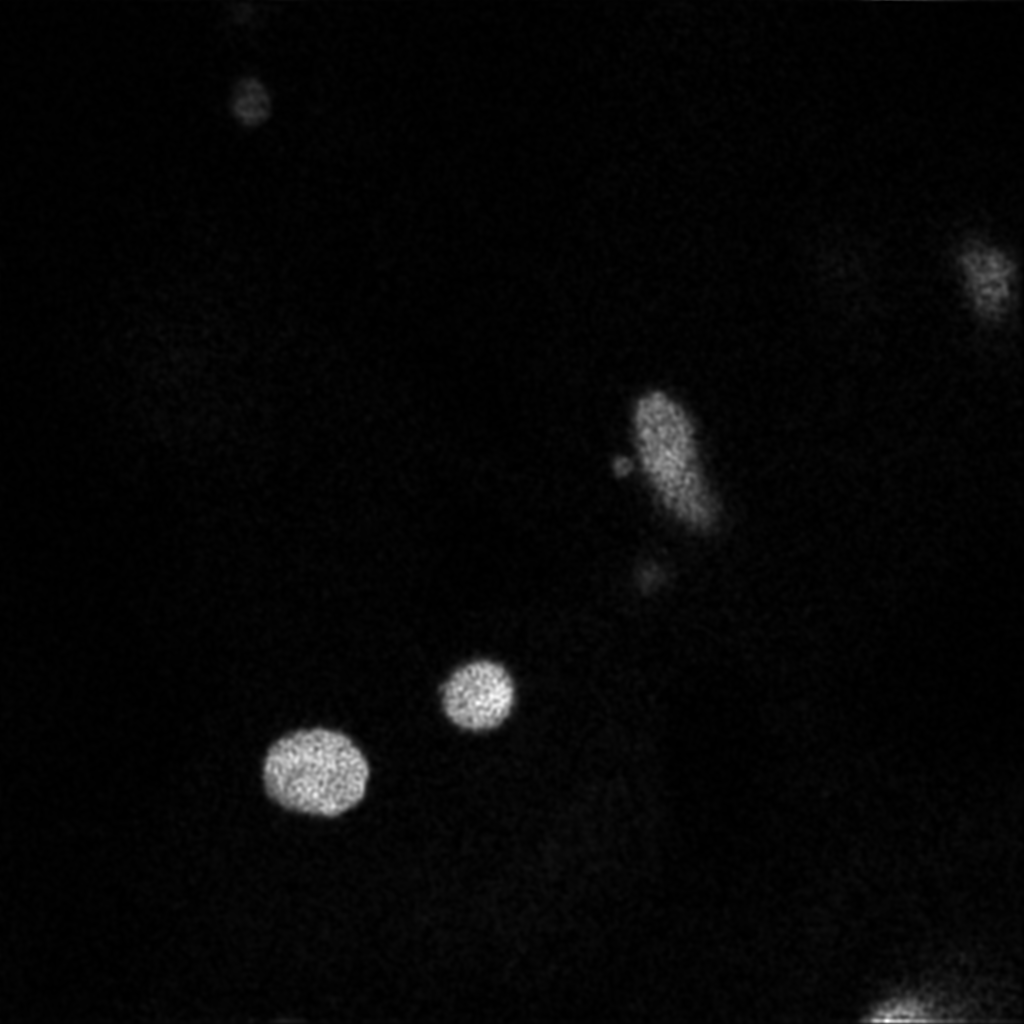

Supplement: Supplementary file 17 — Source data Fig. 9 [file 44318_2024_333_MOESM17_ESM.zip › Figure 9/9D/RIM_Cy3_ITS2_CTR.8-bit.tif]

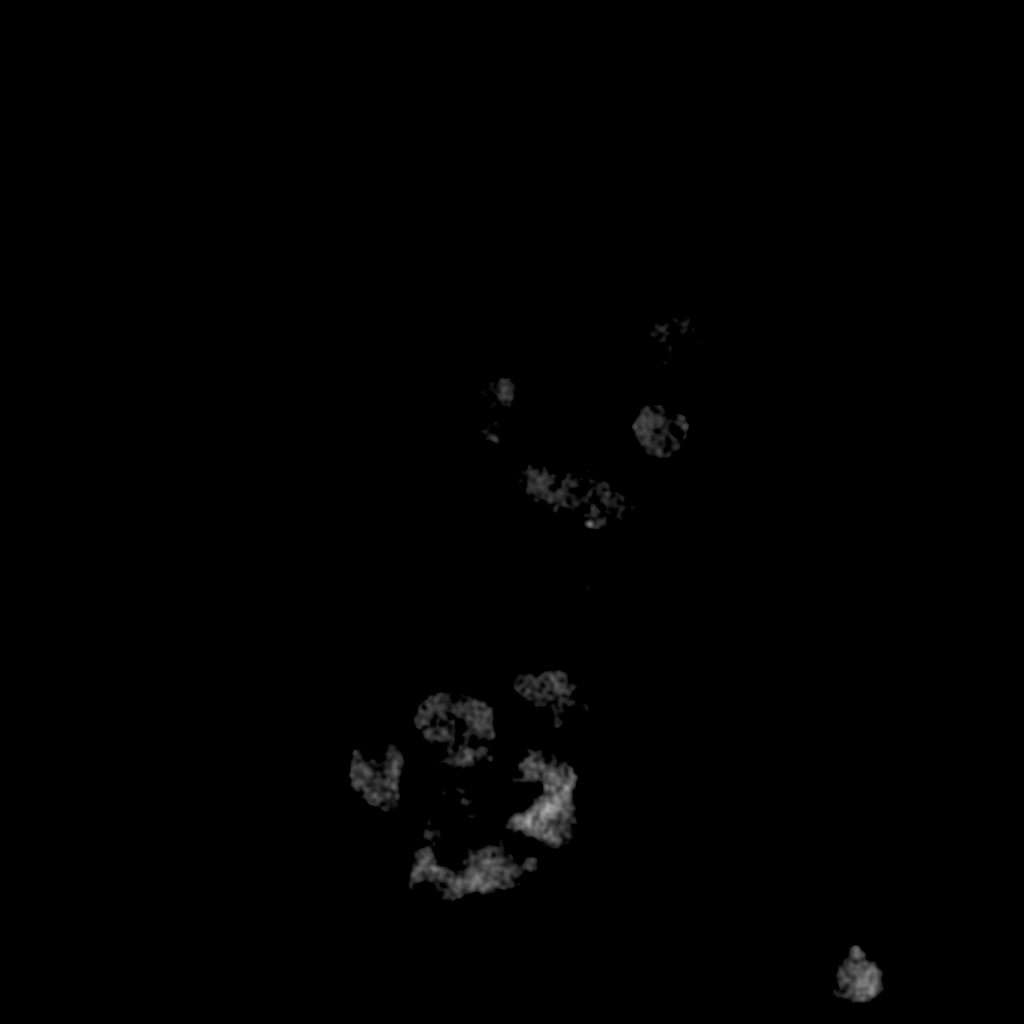

Supplement: Supplementary file 17 — Source data Fig. 9 [file 44318_2024_333_MOESM17_ESM.zip › Figure 9/9D/RIM_Cy3_ITS2_MG132_A_B.8-bit.tif]

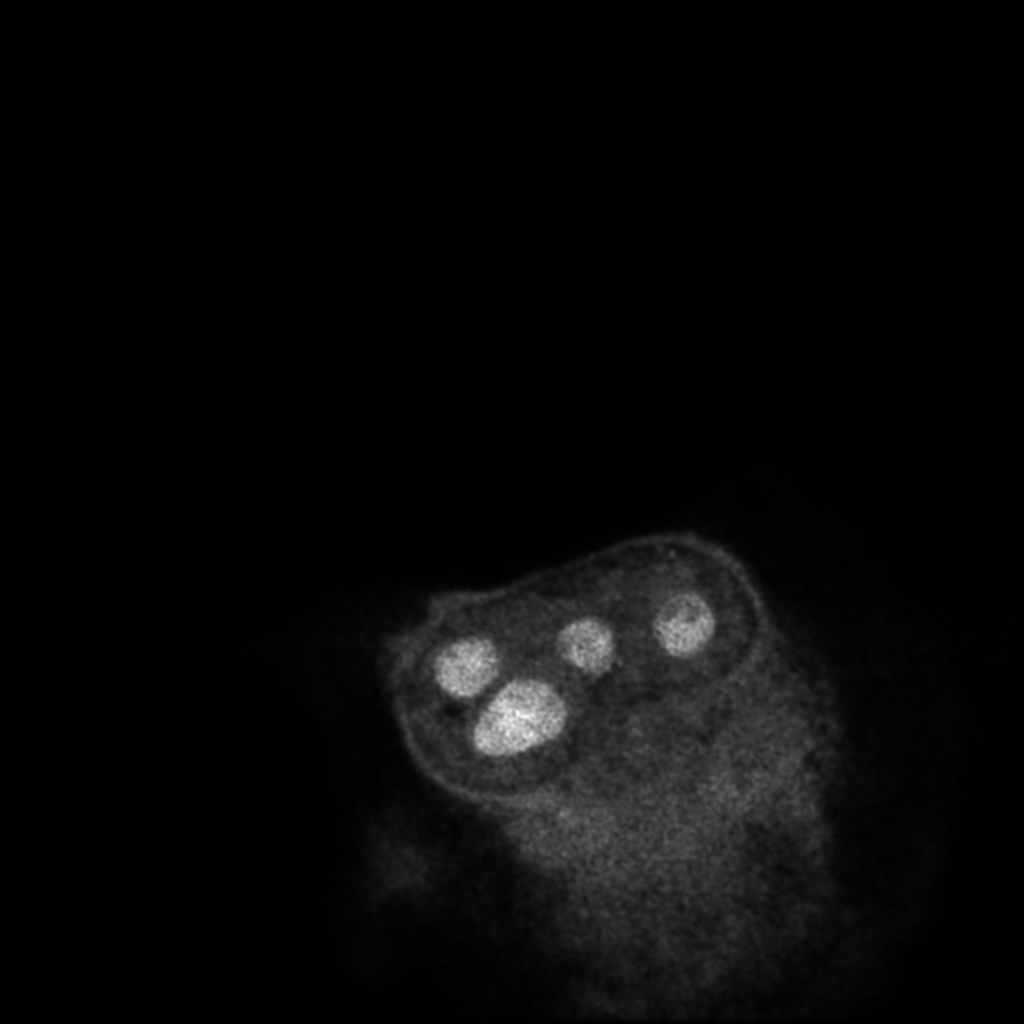

Supplement: Supplementary file 17 — Source data Fig. 9 [file 44318_2024_333_MOESM17_ESM.zip › Figure 9/9D/RIM_RPL11_ITS1_CTR.8-bit.tif]

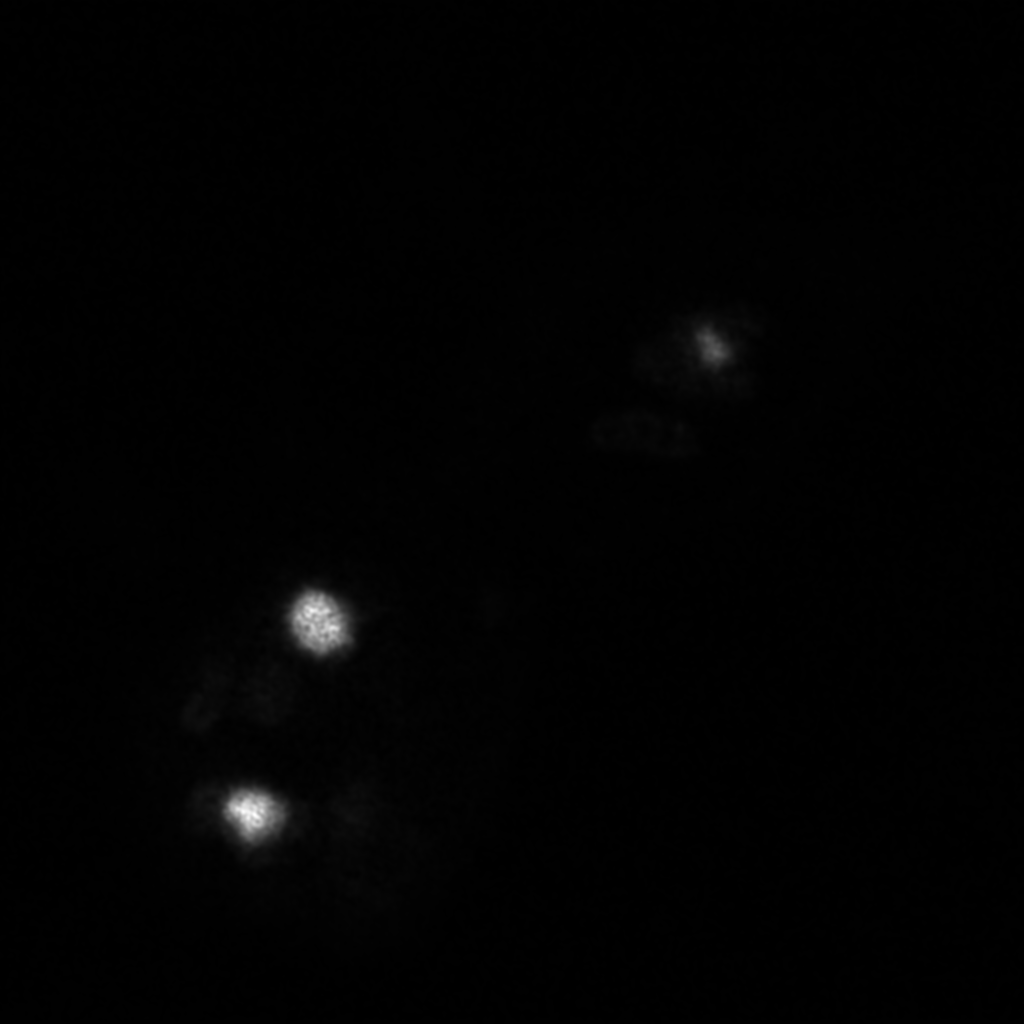

Supplement: Supplementary file 17 — Source data Fig. 9 [file 44318_2024_333_MOESM17_ESM.zip › Figure 9/9D/RIM_RPL11_ITS1_MG132_A.8-bit.tif]

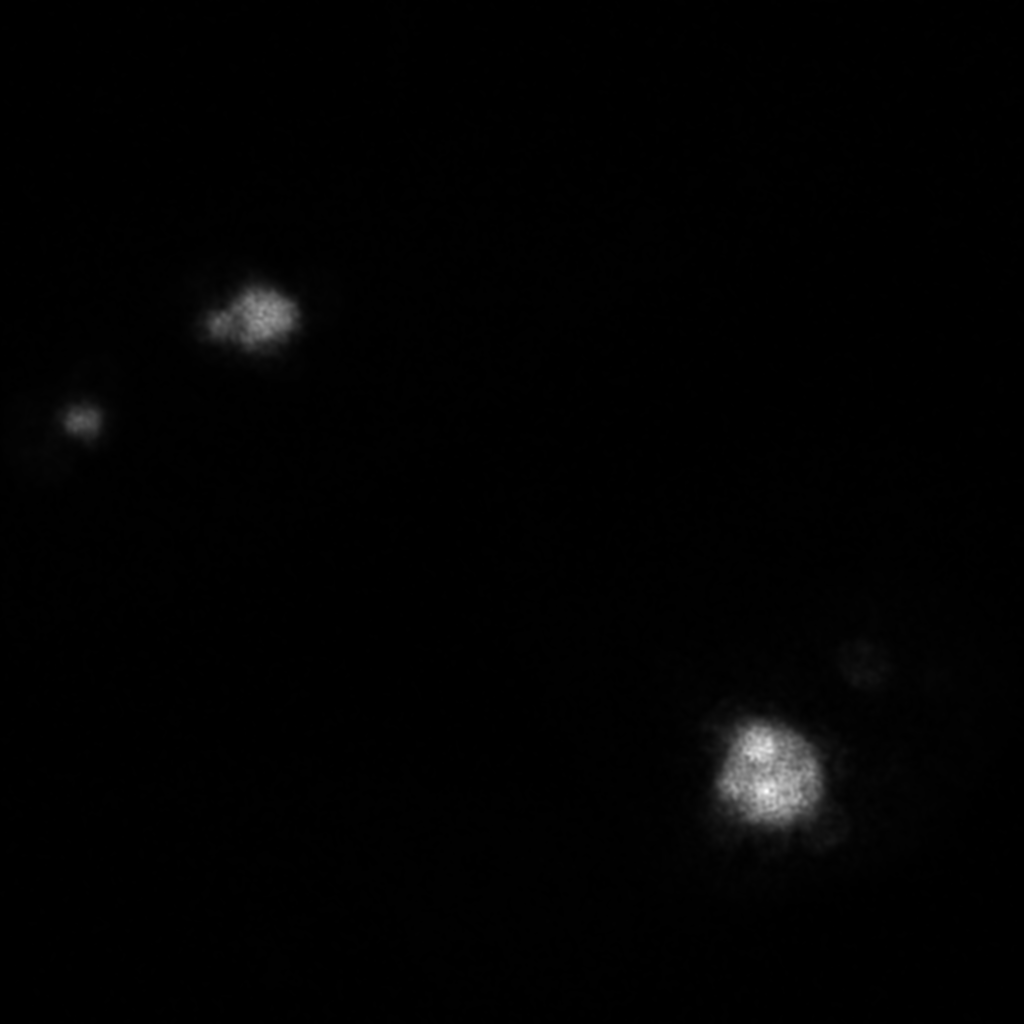

Supplement: Supplementary file 17 — Source data Fig. 9 [file 44318_2024_333_MOESM17_ESM.zip › Figure 9/9D/RIM_RPL11_ITS1_MG132_B.8-bit.tif]

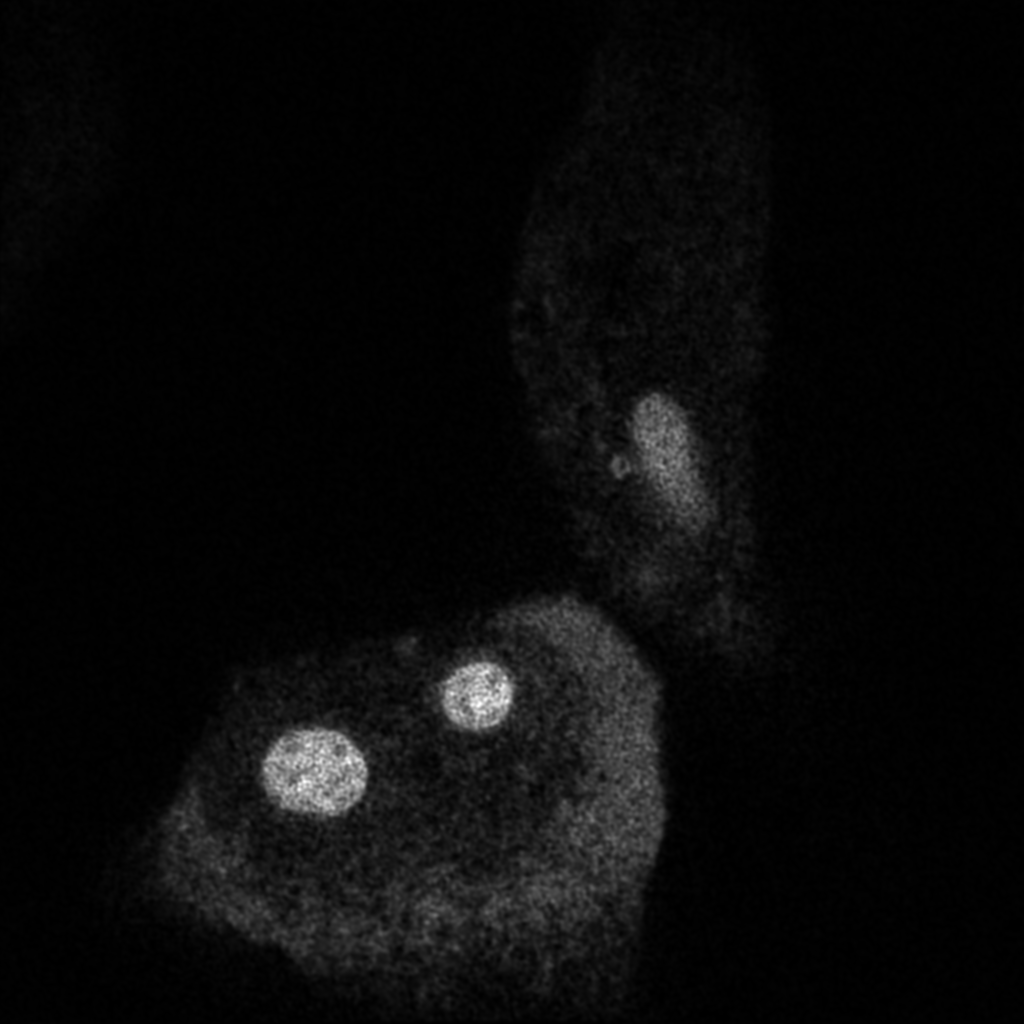

Supplement: Supplementary file 17 — Source data Fig. 9 [file 44318_2024_333_MOESM17_ESM.zip › Figure 9/9D/RIM_RPL11_ITS2_CTR.8-bit.tif]

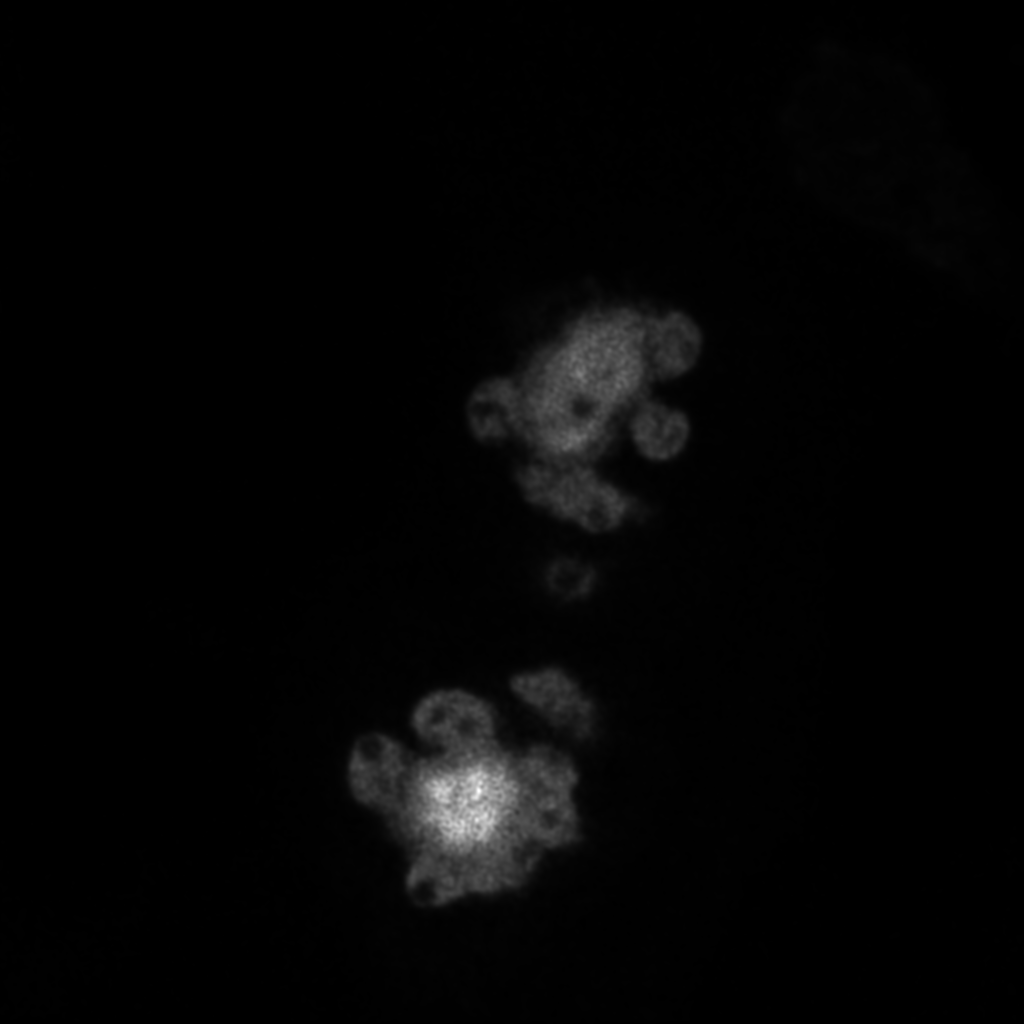

Supplement: Supplementary file 17 — Source data Fig. 9 [file 44318_2024_333_MOESM17_ESM.zip › Figure 9/9D/RIM_RPL11_ITS2_MG132_A_B.8-bit.tif]
